# Structure units oriented approach towards collective synthesis of sarpagine-ajmaline-koumine type alkaloids

**DOI:** 10.1038/s41467-022-28535-x

**Published:** 2022-02-17

**Authors:** Wen Chen, Yonghui Ma, Wenyan He, Yinxia Wu, Yuancheng Huang, Yipeng Zhang, Hongchang Tian, Kai Wei, Xiaodong Yang, Hongbin Zhang

**Affiliations:** Key Laboratory of Medicinal Chemistry for Natural Resource, Ministry of Education; Yunnan Provincial Center for Research & Development of Natural Products; School of Chemical Science and Technology, Yunnan University, Kunming, 650091 P. R. China

**Keywords:** Synthetic chemistry methodology, Natural product synthesis

## Abstract

Sarpagine-Ajmaline-Koumine type monoterpenoid indole alkaloids represent a fascinating class of natural products with polycyclic and cage-like structures, interesting biological activities, and related biosynthetic origins. Herein we report a unified approach towards the asymmetric synthesis of these three types of alkaloids, leading to a collective synthesis of 14 natural alkaloids. Among them, akuammidine, 19-*Z*-akuammidine, vincamedine, vincarine, quebrachidine, vincamajine, alstiphylianine J, and dihydrokoumine are accomplished for the first time. Features of our synthesis are a new Mannich-type cyclization to construct the key indole-fused azabicyclo[3.3.1]nonane common intermediate, a SmI_2_ mediated coupling to fuse the aza-bridged E-ring, stereoselective olefinations to install either the 19-*E* or 19-*Z* terminal alkenes presented in the natural alkaloids, and an efficient iodo-induced cyclization to establish the two vicinal all-carbon quaternary centers in the Koumine-type alkaloids.

## Abstract (web summary)

The sarpagine-ajmaline-koumine type alkaloids are among the most important groups of monoterpenoid indole alkaloids, having notable biological activity. Here the authors perform divergent syntheses of 14 natural products in this family from a common intermediate.

## Introduction

**Fig. 1:**
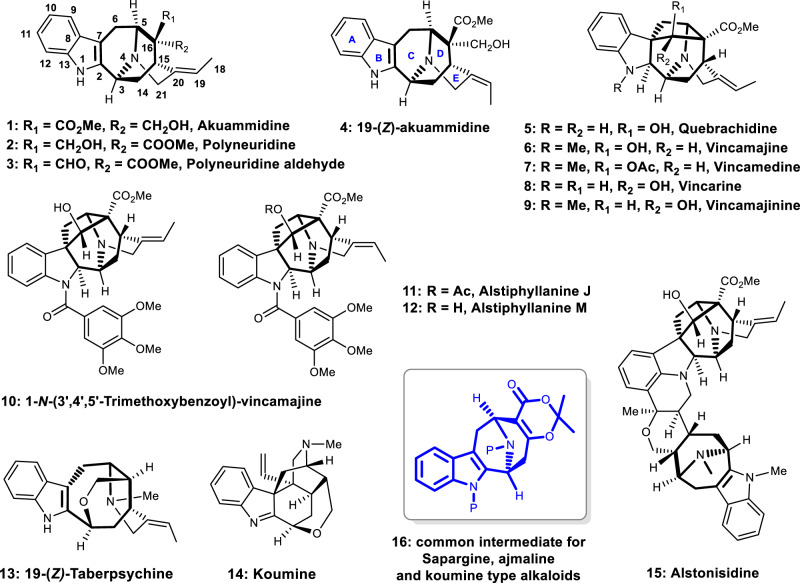
Some representative sarpagine-ajmaline-koumine type alkaloids. In this work, alkaloids **1**–**9**, **11**, **13**, and **14** are synthesized.

The sarpagine alkaloids feature a polycyclic ring system with an azabicyclo[3.3.1]-nonane core. These alkaloids serve as the biogenetic precursors for the more complex ajmaline and koumine type indole alkaloids^1–3^. The sarpagine-ajmaline-koumine type alkaloids are among the most important group of monoterpenoid indole alkaloids^4–6^. Those biogenetically related alkaloids have been isolated mainly from the medicinal plant family Apocynaceae and Loganiaceae, especially from the genera *Alstonia*, *Rauwolfia,* and *Gelsemium* in low natural abundance^7–10^. A number of these alkaloids possess important biological activities, including anti-leishmanial^11^, anti-malaria parasites^12^, anti-inflammatory^13^, antihypertensive^14^, anticancer^15^, and accelerating the sciatic nerve regeneration activities^16^. The typical molecules are indicated in Fig. 1. Because of their characteristic indole-fused azabicyclo[3.3.1]-nonane structures and prominent biological activities, sarpagine-ajmaline-koumine related alkaloids have attracted attention from the organic synthetic community for decades^17–25^. Synthetic efforts have resulted in a number of elegant strategies and culminated with the synthesis of a series of sarpagine˗ajmaline-koumine type alkaloids. Formation of the indole fused azabicyclo[3.3.1]nonane structures could be roughly classified to five categories: the Pictet-Spengler cyclization (forming the C2-C3 bond) approaches, the intramolecular condensation/addition methodologies (constructing the C15-C16 bond), the transition metal-mediated cyclizations, the cycloaddition/annulation strategies, and the Friedel-Crafts acylation approach (forming the C6-C7 bond). The first category includes Tamelen’s^26^, Masamune’s^27^, Craig’s^28^, Sudhakar’s^29^ Pictet-Spengler cyclization procedures, and most recently, Qi’s aza-Achmatowicz rearrangement of indole-tethered furan followed by Pictet-Spengler cyclization^30^. The second category constitutes Cook’s approach based on Dieckman condensation of carboline derivatives^31–42^, and Bailey’s intramolecular Michael addition procedures^43,44^. The third category comprises Martin’s approach via Pauson-Khand reaction^45^, olefin metathesis^46^, Kuethe’s intramolecular Heck cyclization approach^47^, and most recently, Zhang’s Copper-catalyzed oxidative cyclization^48,49^. The fourth category includes Ohba’s intramolecular oxazole–olefin Diels–Alder cycloaddition^50^, and Gaich’s [5 + 2] cycloaddition followed by ring enlargement and Fischer indole annulation^51–53^. The last category is Kwon’s intramolecular Friedel-Crafts acylation approach^54^. Numerous sarpagine˗ajmaline-koumine type alkaloids have been synthesized based on above-mentioned strategies, however, quebrachidine, vincamedine, and its analogues are not yet conquered to our knowledge by total synthesis^17–25^. The natural alkaloid quebrachidine (**5**) and its *N*-methyl congener vincamajine (**6**) and vincamedine (**7**) are among the most highly functionalized and synthetically challenging ajmaline type alkaloids. Quebrachidine is the biogenetic precursor of bisindole alkaloid alstonisidine (**15**) and has been isolated for more than 50 years^55^, while vincamedine and alstiphyllanines (Fig. 1, **11** and **12**) possess potent vasorelaxant activity^56^. It is also noteworthy that alstiphyllanines are 19-*Z*-derivatives of quebrachidine.

In this work, we report a structure-unit-oriented strategy towards the synthesis of 14 natural monoterpenoid indole alkaloids. Although numerous synthetic methods have been developed for sarpagine-ajmaline alkaloids as well as structurally related analogues, flexible and unified synthetic routes that lead to collective synthesis of these types of alkaloids, especially towards both 19-*E* and 19-*Z* isomers, are rarely documented^17–25^.

## Results

### Retrosynthetic analysis

**Fig. 2:**
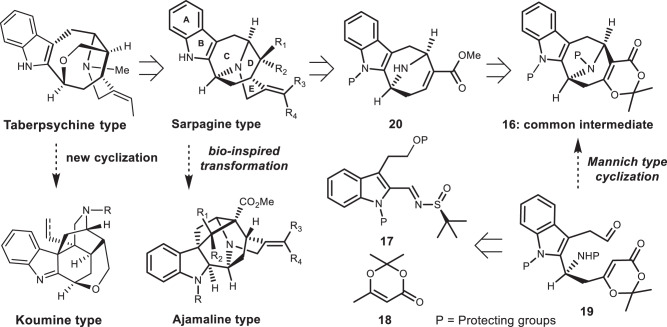
Retrosynthetic analysis based on amide-mediated Mannich-type cyclizations. The collective synthesis of sarpagine-ajmaline-koumine type alkaloids are based on common intermediate **16**.

As part of our ongoing program in seeking flexible and divergent synthetic strategies toward bioactive natural products and its analogues^57–61^, we recently disclosed a Lewis acid-mediated intramolecular Prins reaction of dioxinones to construct medium-sized carbocycles bearing oxa-bridged bicyclic structural units^62^. We envisioned that the Mannich-type-cyclization version of our previous protocol might lead to the highly functional 9-azabicyclo[3.3.1]nonane ring system (**16**, Fig. 2), an ideal intermediate with properly located functional groups and necessary carbons for the synthesis of akuammidine-related alkaloids shown in Fig. 1.

Our retrosynthetic analysis is outlined in Fig. 2. One of the key transformations in the synthetic plan is cascade cyclizations (Fig. 2, transformation of **19**→**16**) to form the aza-bridged eight-membered ring system from indole fused chain compound (**19**). Although we have succeeded previously the synthesis of oxa-bridged medium-sized ring systems^62^, and aza-Prins cyclization have been used elegantly in the synthesis of six-membered carbocyclic rings^63,64^, the proposed tandem cyclizations to obtain aza-bridged bicyclo[3.3.1]nonane common structure units (**16**) are challenging. We postulated that an amine or amide participated intramolecular Mannich-type reaction of dioxinones under Lewis acid conditions would provide access to the required common intermediate (**16**, Fig. 2). Based on the well-located functional groups, with proper manipulations, we would be able to synthesize the natural alkaloids shown in Fig. 1.

### Preparation of the common azabicyclo[3.3.1]-nonane core

**Fig. 3:**
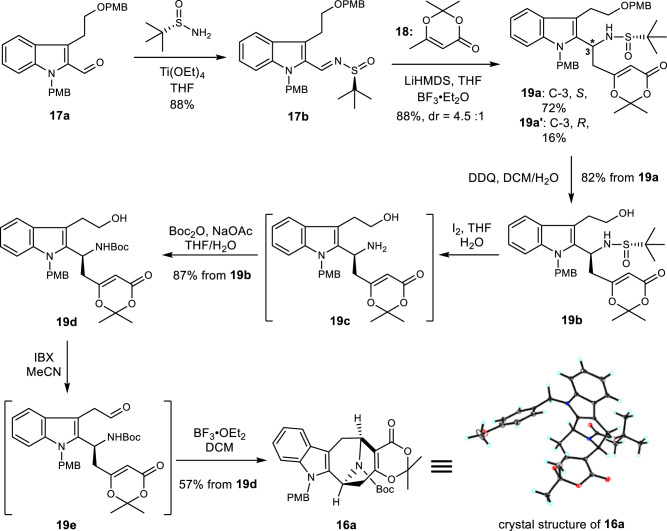
Gram-scale synthesis of common intermediate 16a. The key transformations to access the common intermediate **16a** include a vinylogous Mannich reaction of lithium dienolate **18**, and a Lewis acid mediated Mannich type cyclization of **19e**. Ac acetyl, Boc *t*-butoxycarbonyl, DCM dichloromethane, DDQ 2,3-dichloro-5,6-dicyano-1,4-benzoquinone, IBX *o*-iodoxybenzoic acid, LiHMDS lithium bis(trimethylsilyl)amide, PMB *p*-methoxybenzyl, THF tetrahydrofuran.

We commenced our synthetic studies with the preparation of *tert*-butanesulfinamide **19a** (Fig. 3). The desired sulfinamide **19a** was obtained after two steps in 63% yield from known aldehyde **17a**, obtained two steps from commercially available tryptophol^65^. Our initial plans were oxidation of sulfinamide **19b** (after removal of PMB with DDQ) or amine **19c** (after removal of PMB with DDQ and the *tert-*butanesulfinyl group with iodine^61^) to its corresponding aldehydes and subjected it to the proposed Mannich-type reactions. Unfortunately, both substrates (**19b** and **19c**) failed to yield reasonable amounts of aldehydes under a number of oxidation conditions. To circumvent this problem, the amine was converted to amide **19d** with Boc-anhydride (71% overall yields in three steps from **19a**). Oxidation of alcohol **19d** with IBX^66^ provided an unstable aldehyde (**19e**), which was used immediately in the cyclization under Lewis acid conditions. To our delight, the cascade sequence proceeded precisely and produced the desired **16a** as a single diastereoisomer in 57% yield (gram-scale) over two consecutive steps. The correct stereochemistry of the pentacyclic core was confirmed by single crystal X-ray analysis.

**Fig. 4:**
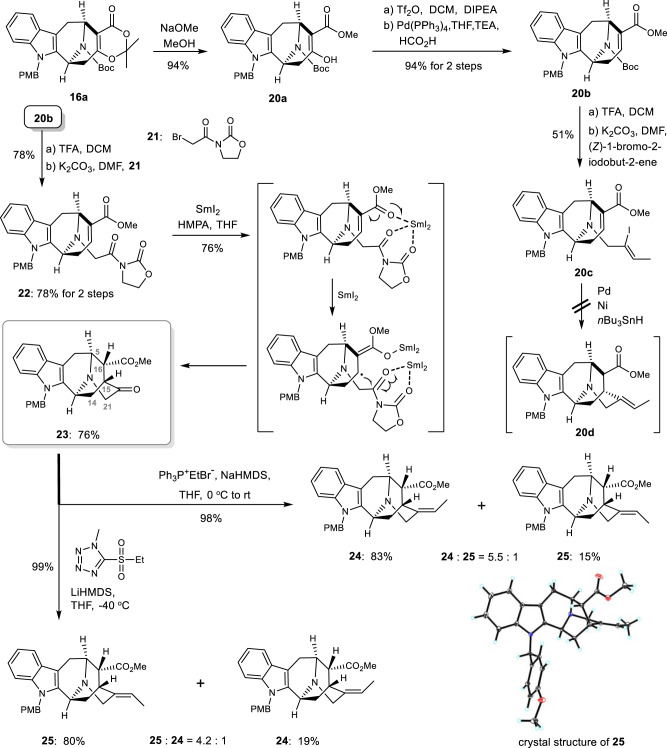
Synthesis of the common sarpagine type skeletons. After construction of the bridged E-ring via SmI_2_ mediated SET reaction, the double bonds were assembled by Wittig reaction (*E*-selectivity) and Julia olefination (*Z*-selectivity). DIPEA diisopropylethylamine, DMF *N*,*N*-dimethylformamide, HMPA hexamethylphosphoramide, Me methyl, Ph phenyl, TEA triethylamine, Tf trifluoromethanesulfonyl, TFA trifluoroacetic acid, Et ethyl, NaHMDS sodium bis(trimethylsilyl)amide.

With the common intermediate **16a** in hand, we next explored the synthesis of sarpagine-ajmaline alkaloids indicated in Fig. 1, aiming to synthesize both 19-*E* and 19-*Z* natural isomers. Treatment of **16a** with sodium methoxide afforded methyl ester **20a** in 94% yield. Deoxygenation of enol **20a** via formation of triflate with Comins reagent followed by palladium-catalyzed hydrogenolysis^67,68^ provided **20b** in excellent yield. Next, the Boc protecting group was removed with trifluoroacetic acid in dichloromethane and the resultant was treated with (*Z*)-1-bromo-2-iodobute-2-ene to afford intermediate **20c**. Our initial plan to fuse the C_15_-C_20_ carbon-carbon bond of akuammidine-related alkaloids was to follow the well-established procedure via reductive Heck-type reactions^69–76^. To our dismay, no desired product was obtained under various palladium and nickel mediated conditions, with dehalogenation product being isolated in most cases (see Supplementary Information for details). Treatment of iodide **20c** with tri-*n*-butyltin hydride^77^ was also fruitless, with a complex mixture being obtained. Difficulty in assembling the bridged E-ring prompted us to explore alternative methods. It was of our interests to use Skrydstrup’s chemistry^78–84^, namely SmI_2_ mediated acyl radical reaction. As indicated in Fig. 4, Skrydstrup coupling would lead to a tempting product, namely intermediate **23**, which bears a tunable carbonyl moiety for further manipulation towards desired 19-*E* and 19-*Z* olefins. The intermediate **20b** was then transformed to its corresponding *N*-acyl-oxazolidinone derivative **22** (2 steps, 76%). The well-established Skrydstrup’s conditions (SmI_2_/H_2_O/THF) unfortunately failed to promote the desire cyclization, however, after examining several other conditions, we finally established a reproducible procedure for constructing the bridged E-ring (Fig. 4). SmI_2_ mediated SET reaction gave the coupling product in 76% yield with excellent diastereoselectivity (single C_16_ isomers). We deduced that the chelation of samarium diiodide with the carbonyl groups as proposed by Skrydstrup^79^ (Fig. 4) secured the desired cyclization. The NOESY correlations of H-16 to both H-14 and H-15 for compound **23** indicated a β-orientation of the H-16 (Fig. 4, see Supplementary Information for spectra). Thus, the pentacyclic skeletons for akuammidine-related alkaloids had been successfully assembled and ready for late-stage manipulation.

We next focused on the selective synthesis of *E* and *Z* alkenes with ketone **23**. Treatment of ketone **23** with Wittig reagent^85–87^ in THF in the presence of NaHMDS provided olefins **24** (isolated as the major isomer, 83% yield) and **25** (15% yield) in a 98% combined yield. Treatment of ketone **23** with Julia reagent^88^ in THF altered the ratio and provided olefin **25** as the major isomer in 80% yield together with **24** (19% yield). Suitable crystal was obtained from compound **25**, and the 19-*Z* stereochemistry was established by X-ray crystallography (Fig. 4). This new approach enabled access to either 19-*E* (Wittig-olefination, *trans*-selective) or 19-*Z* (Julia-olefination, *cis*-selective) isomers selectively.

### Total synthesis of natural sarpagine-type and ajmaline-type alkaloids

**Fig. 5:**
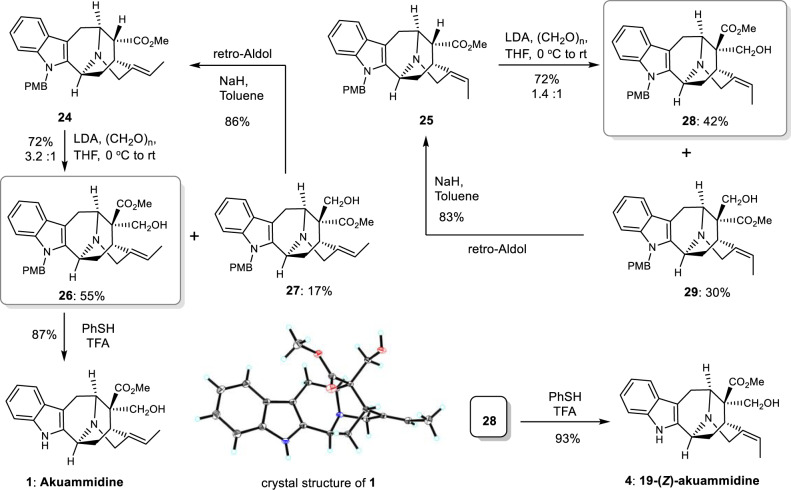
Synthesis of akuammidine (1) and 19-*Z*-akuammidine (4). Aldol condensation with formaldehyde. LDA lithium diisopropylamide.

With the advanced intermediates (**24** and **25**) in hand, we next began the journey towards akuammidine-related alkaloids. Treatment of **24** with LDA followed by formaldehyde (Fig. 5), we obtained desired C16 stereoisomer **26** (55% yield) together with byproduct **27** (17% yield). Using compound **25**, similar operation provided intermediates **28** (42%) and **29** (30%). It is worthwhile to note that the undesired C-16 isomers could be recycled, a retro-Aldol reaction in the presence of sodium hydride converted **27** and **29** back to its corresponding starting materials (**24**: 86% yield, and **25**: 83% yield, Fig. 5). Deprotections of PMB in intermediates **26** and **28** under acidic conditions furnished the first total synthesis of natural akuammidine (**1**, confirmed by X-ray analysis) and 19-*Z*-akuammidine (**4**). The NMR spectra as well as physical data of our synthetic sample were consistent with those reported in the literature^89,90^.

**Fig. 6:**
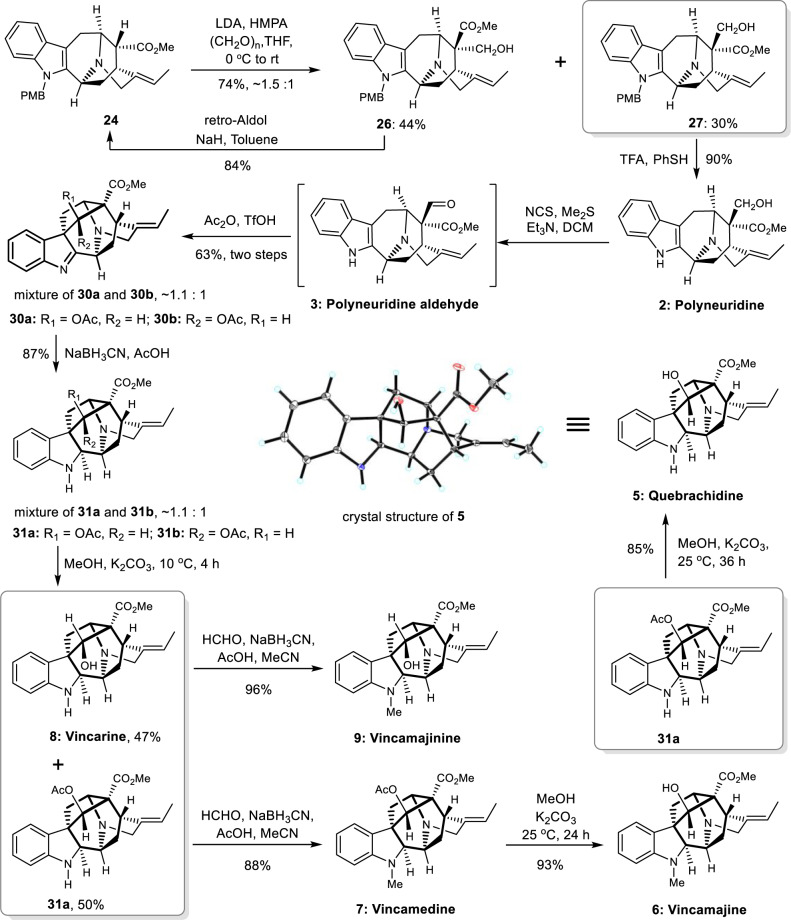
Synthesis of ajmaline type alkaloids. Quebrachidine, vincarine, vincamedine, and vincamajine were synthesized for the first time. NCS *N*-chlorosuccinimide.

Having secured the total synthesis of akuammidines, we next turned our attention to the synthesis of more challenging ajmaline-type alkaloids. To increase the ratio of intermediate **27**, a number of additives were used including BF_3_ ∙ Et_2_O, TMSCl (trimethylsilyl chloride), TMEDA (*N*,*N*,*N*′,*N*′-tetramethylethylenediamine), and HMPA. The addition of HMPA could slightly increase the ratio of compound **27** (30% yield) while undesired C-16 isomer **26** was converted back to starting material (**24**) through retro-aldol reaction in 84% yield (Fig. 6). Treatment of **27** with TFA in the presence of PhSH yielded natural polyneuridine (**2**) in 90% yield^91,92^. Corey-Kim oxidation of **2** provided the biosynthetic precursor polyneuridine aldehyde (**3**)^3,4,21^. Upon exposure to acidic conditions (Ac_2_O and TfOH, modification of Cook’s conditions^35^), an inseparable mixture of C17 stereoisomers (**30a** and **30b**, 63% yields in two steps, *dr* = 1.1:1 based on ^1^H-NMR) was obtained. Reduction of the mixture with sodium cyanoborohydride gave amines **31a** and **31b** (inseparable mixture) in 87% combined yield (Fig. 6). We envisioned that the inseparable C17-acetate isomers might react differently under hydrolysis conditions due to its different steric hindrances. Thus, the mixture of **31a** and **31b** were treated with K_2_CO_3_ in methanol. This process provided unchanged acetate **31a** in 50% yield together with natural vincarine (**8**, 47% yield, from hydrolysis of **31b**, the first total synthesis), the diastereoisomer of quebrachidine^6,36,93^. Reductive amination of **31a** with formaldehyde furnished the first total synthesis of vincamedine (**7**)^94^. Hydrolysis of **7** afforded vincamajine (**6**)^94^ in 93% yield. Further hydrolysis of **31a** provided the first total synthesis of natural alkaloid quebrachidine (**5**)^55,95^. The structure of synthetic quebrachidine was confirmed by X-ray crystallography.

Treatment of vincarine (**8**) with sodium cyanoborohydride in the presence of formaldehyde gave vincamajinine (**9**)^36^. The NMR spectra and physical data of our synthetic samples were in complete agreement with the reported data. It is noteworthy that vincarine has been documented in literature for more than 50 years, no complete reported NMR data are available in the literature^6,36^.

**Fig. 7:**
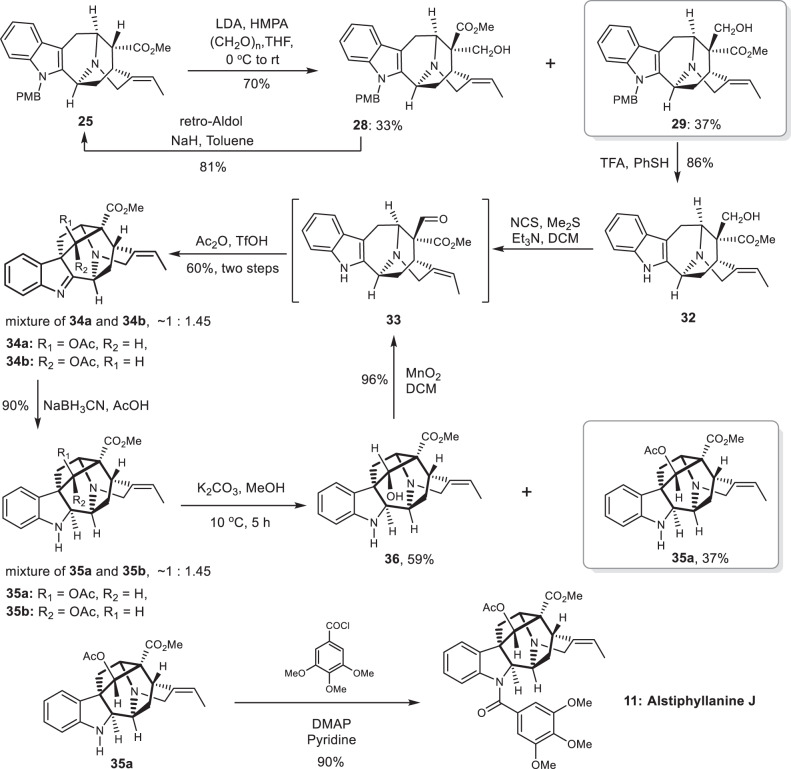
Synthesis of alstiphyllanine J. Alstiphyllanine J has a 19-*Z*-olefin moiety. DMAP *N*,*N*-4-dimethylaminopyridine.

Next, we concentrated on the total synthesis of alstiphyllanines (with a 19-*Z*-olefin moiety), a number of bioactive ajmaline-type alkaloids differs from each other only in amide substituents^56^. Aldol reaction of compound **25** (from Julia-olefination in Fig. 4) in the presence of HMPA afforded desired isomer **29** in 37% yield (Fig. 7). Treatment of **29** with TFA in the presence of PhSH afforded amine **32** in 86% yield. Oxidation of **32** under Corey-Kim conditions gave aldehyde **33**, which was directly subjected to acidic conditions to yield cyclization products **34a** and **34b** (60% yield over two steps, ∼1:1.45 ratio) as an inseparable mixture of C17 diastereoisomers. Selective reduction of the imine moiety presented in **34a** and **34b** with sodium cyanoborohydride afforded amines **35a** and **35b** (inseparable mixture of C17 diastereoisomers) in 90% combined yield. Hydrolysis of the mixture of **35a** and **35b** under our standard conditions provided separable alcohol **36** (59%) and acetate **35a** (37%). The alcohol **36** could be recycled by oxidation with MnO_2_ in dichloromethane^96^. Finally, treatment of **35a** with eudesmoyl chloride in pyridine furnished the first total synthesis of alstiphyllanine J (**11**). The NMR spectra of our synthetic sample agree well with the reported data^56^.

### Total synthesis of natural koumine-type alkaloids

**Fig. 8:**
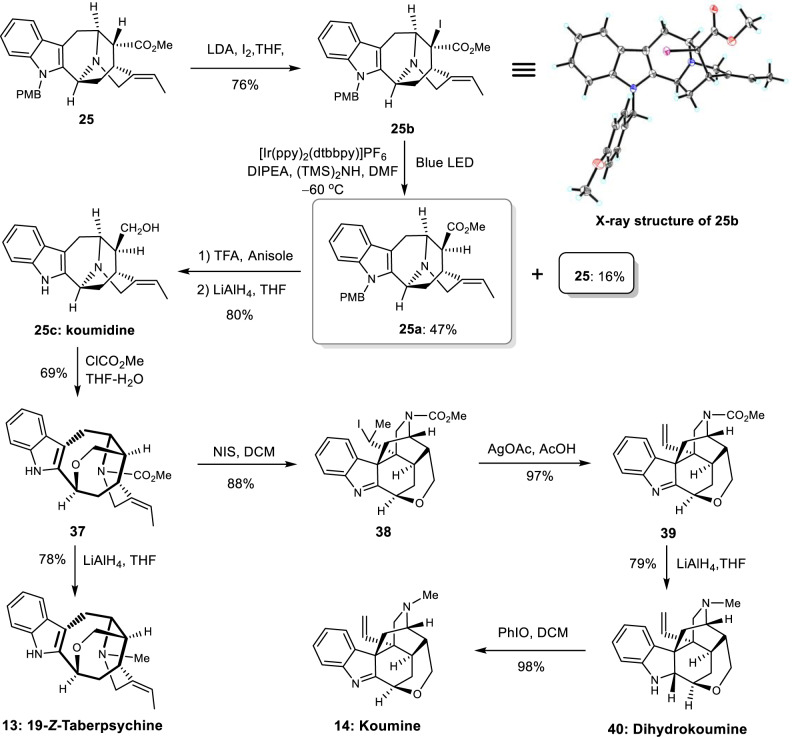
Total synthesis of koumine-type alkaloids. Key transformations are light induced radical reduction of iodide **25b** and NIS mediated cyclization of **37** to access koumine-type alkaloids. [Ir(ppy)_2_(dtbbpy)]PF_6_ (4,4’-di-*tert*-butyl-2,2’-bipyridine)bis[(2-pyridinyl)phenyl]iridium(III) hexafluorophosphate, NIS *N*-iodosuccinimide, DIPEA *N,N*-Diisopropylethylamine.

Finally, to further demonstrate the flexibility of our strategy, we decided to synthesize the challenging koumine-type alkaloids, a cage-like alkaloid family with two vicinal all-carbon quaternary centers^5^. There are two key issues in the synthesis of koumine-type alkaloids from common intermediate **25**, namely the epimerization of the C-16 stereochemistry and a cyclization to form the two vicinal all-carbon quaternary centers. Experiments (see Supplementary Information) to epimerize the C-16 stereochemistry using a number of bases and proton donors failed to produce any desired epimer **25a** (Fig. 8). Next, intermediate **25** was converted to iodide **25b**, aiming to alter the C-16 stereochemistry via radical reduction. Treatment of compound **25b** with *n*-Bu_3_SnH failed again to provide **25a**. Light-induced radical reductions were then attempted^97^. After extensive experiments (see Supplementary Information for details), we finally found that reduction of **25b** with catalytic amount of [Ir(ppy)_2_(dtbbpy)]PF_6_ in the presence of DIPEA and (TMS)_2_NH under blue LED (−60 °C) provided desired **25a** in 47% yield (Fig. 8), with **25** being recovery in 16% yield. Deprotection of **25a** with TFA followed by reduction with LiAlH_4_ (LAH) gave natural koumidine **25c** (80% yield over two steps)^98,99^.

Treatment of the koumidine **25c** with methyl chloroformate afforded amide **37** in 69% isolated yield. Reduction of **37** with LAH provided natural 19-*Z*-taberpsychine (**13**, 78%)^100^. Although alkaloid koumine has been prepared by both partial synthesis and total synthesis in the literature^5,24,49,99^, cyclizations to construct the vicinal all-carbon quaternary centers in koumine-type structures generally followed the chemistry developed by Liu (SeO_2_/H_2_O, oxidation followed by cyclization in low yield)^101^ and Sakai (Pd^0^, NaH in DMF, based on pre-synthesized 18-hydroxyl derivatives)^102^. Alternative way was reported recently via gold mediated indolyl addition to allene, and the cyclization adduct was converted to koumine in another five steps^49^. In this synthesis, we designed to fuse the two vicinal all-carbon quaternary centers via iodo-induced indolyl cyclization, as the resulting iodide (**38**) could be converted to olefin via elimination (Fig. 8). The amide **37** was thus treated with NIS in dichloromethane. To our delight, an efficient cyclization occurred, with iodide **38** being isolated as a single isomer in 88% yield. Treatment of iodide **38** with silver acetate in acetic acid furnished the olefin (**39**). After reduction of the amide bond with LAH, the first total synthesis of dihydrokoumine (**40**) was achieved. Oxidation of dihydrokoumine with PhIO in dichloromethane^49^ afforded koumine in 98% yield. The NMR spectra of our synthetic alkaloids are consistent with those of the natural alkaloids^103,104^.

## Discussion

We have developed a structure unit oriented strategy for the synthesis of cage-like Sarpagine-Ajmaline-Koumine type monoterpenoid indole alkaloids from readily available commercial materials. Among the natural alkaloids synthesized, eight molecules are synthesized for the first time. Key transformations developed are boron trifluoride etherate mediated tandem cascade cyclization to install the azabicyclo[3.3.1]nonane structure unit, samarium(II) iodide mediated SET cyclization to fuse the bridged-hexahydropyridine E-ring, trifluoromethanesulfonic acid induced cyclization to construct the all-carbon quaternary center in ajmaline-type alkaloids, and an efficient iodo-induced cyclization to establish the two vicinal all-carbon quaternary centers in the Koumine-type alkaloids. Our synthetic strategy provided a platform, enabling access to a wide variety of Sarpagine-Ajmaline-Koumine type natural products as well as their analogues, and should be found further application in the synthesis of bisindole alkaloids such as alstonisidine.

## Methods

### General

Melting points were measured on a Hanon MP 430 auto melting-point system and are uncorrected. The infrared (IR) spectra were recorded on a Nicolet iS10 FTIR spectrometer with 4 cm^−1^ resolution and 32 scans between wavenumber of 4000 cm^−1^ and 400 cm^−1^. Samples were prepared as KBr disks with 1 mg of samples in 100 mg of KBr. ^1^H-NMR and ^13^C-NMR spectra were recorded on Bruker Avance 400 and 600 spectrometers. Chemical shifts are reported in parts per million (δ) referenced to tetramethylsilane (0.0 ppm), chloroform (7.26 ppm or 77.16 ppm), and methanol (3.31 ppm or 49.0 ppm), respectively. Data for ^1^H-NMR and ^13^C-NMR spectroscopy are reported as follows: chemical shift (δ ppm), multiplicity (s = singlet, d = doublet, t = triplet, q = quartet, m = multiplet, br = broad), coupling constant (Hz), integration. High Resolution Mass spectra were taken on AB QSTAR Pulsar mass spectrometer or Aglient LC/MSD TOF mass spectrometer. Optical rotations were recorded on a JASCO P-2000 polarimeter. All new compounds were characterized by IR, ^1^H NMR, ^13^C NMR, and HRMS. Silica gel (200–300 mesh) for column chromatography and silica GF_254_ for TLC were obtained from Merck Chemicals Co. Ltd. (Shanghai). Anhydrous THF was dried by distillation over metallic sodium and benzophenone; dichloromethane, pyridine, and methanol were distilled from calcium hydride. Starting materials and reagents used in reactions were obtained commercially from Acros, Aldrich, and Adamas-beta^®^, and were used without purification, unless otherwise indicated. All reactions were conducted in dried glassware under a positive pressure of dry nitrogen or argon. Reagents and starting materials were accordingly transferred via syringe or cannula. Reaction temperatures refer to the external oil bath temperature.

## Supplementary information

Supplementary Information

## Data Availability

The authors declare that the data supporting the findings of this study are available within the article and its Supplementary Information files. For the experimental procedures and spectroscopic and physical data of compounds, see Supplementary Methods. For ^1^H and ^13^C{^1^H} NMR spectra of compounds, see Supplementary Figs. 1–109. For the comparisons of ^1^H and ^13^C NMR spectra of the natural and synthetic alkaloids, see Supplementary Tables 4–20. For the X-ray crystallographic data of compounds **16a**, **25**, **1**, **5**, and **25b**, see Supplementary Tables 21–25. The X-ray crystallographic coordinates for structures reported in this study have been deposited in the Cambridge Crystallographic Data Centre (**16a**: CCDC 2070256, **25**: CCDC 2070257, **1**: CCDC 2070258, **5**: CCDC 2070259, and **25b**: CCDC 2123071). These data can be obtained free of charge from The Cambridge Crystallographic Data Centre via www.ccdc.cam.ac.uk/data_request/cif.
