# Supplementary material for: Structure units oriented approach towards collective synthesis of sarpagine-ajmaline-koumine type alkaloids
Source: Nat Commun. 2022 Feb 17;13:908. doi: 10.1038/s41467-022-28535-x (PMC8854706; doi:10.1038/s41467-022-28535-x)
Supplement: Supplementary file 1 — Supplementary Information [file 41467_2022_28535_MOESM1_ESM.pdf]

Supplementary Information

Structure Units Oriented Approach Towards Collective Synthesis of Sarpagine-Ajmaline-Koumine Type Alkaloids

Wen Chen, Yonghui Ma, Wenyan He, Yinxia Wu, Yuancheng Huang, Yipeng Zhang, Hongchang Tian, Kai Wei, Xiaodong Yang* and Hongbin Zhang*

Key Laboratory of Medicinal Chemistry for Natural Resource, Ministry of Education, Yunnan Provincial Center for Research and Development of Natural Products, School of Chemical Science and Technology, Yunnan University, Kunming, Yunnan 650091, P. R. China.

*Correspondence to: zhanghb@ynu.edu.cn, yangxd@ynu.edu.cn

Table of Contents

1. Supplementary Methods **S4**

1. 1 General Information **S4**

1.2 Experimental Section **S5**

1.2.1 Synthesis of PMB-protected Tryptophol **S2** S5

1.2.2 Synthesis of Aldehyde **17a** S5

1.2.3 Synthesis of Sulfinimine **17b** S6

1.2.4 Synthesis of Sulfinamide **19a** and Its Diastereomer **19a'** S7

1.2.5 Synthesis of Alcohol **19b** S9

1.2.6 Synthesis of Amide **19d** S10

1.2.7 Synthesis of Amide **16a** S11

1.2.8 Synthesis of Methyl Ester **20a** S12

1.2.9 Synthesis of Ester **20b** S13

1.2.10 Synthesis of Tertiary Amine **20c** S15

1.2.11 Studies on the reductive Heck-type reaction S16

1.2.12 Synthesis of Tertiary Amine **22** S16

1.2.13 Synthesis of Ketone **23** S18
1.2.14 Synthesis of Olefins **24** and **25** S19
1.2.15 Synthesis of Alcohols **26** and **27** S21
1.2.16 Synthesis of Alcohols **28** and **29** S23
1.2.17 Synthesis of Olefin **24** from Alcohol **26** or **27** S26
1.2.18 Synthesis of Olefin **25** from Alcohol **28** or **29** S27
1.2.19 Synthesis of Akuammidine (**1**) S27
1.2.20 Synthesis of 19-(*Z*)-Akuammidine (**4**) S28
1.2.21 Synthesis of Polyneuridine (**2**) S29
1.2.22 Synthesis of the Mixture of **30a** and **30b** S30
1.2.23 Synthesis of the Mixture of **31a** and **31b** S32
1.2.24 Synthesis of Ester **31a** and Vincarine (**8**) S33
1.2.25 Synthesis of Vincamedine (**7**) S34
1.2.26 Synthesis of Vincamajine (**6**) S35
1.2.27 Synthesis of Quebrachidine (**5**) S36
1.2.28 Synthesis of Vincamajinine (**9**) S37
1.2.29 Synthesis of Indole **32** S38
1.2.30 Synthesis of the Mixture of **34a** and **34b** S39
1.2.31 Synthesis of the Mixture of **35a** and **35b** S41
1.2.32 Synthesis of Ester **35a** and Alcohol **36** S42
1.2.33 Synthesis of Aldehyde **33** from Alcohol **36** S43
1.2.33 Synthesis of Alstiphyllanine J (**11**) and its trifluoroacetic acid salt S44
1.2.34 Studies on the epimerization of the C-16 stereochemistry from **25** S46
1.2.35 Synthesis of Iodide **25b** S46
1.2.36 Optimization for light induced radical reduction S48
1.2.37 Synthesis of Olefin **25a** S49
1.2.38 Synthesis of Ester **S4** S50
1.2.39 Synthesis of Koumidine (**25c**) S51
1.2.40 Synthesis of Amide **37** S52
1.2.41 Synthesis of 19-*Z*-Taberpsychine (**13**) S53
1.2.42 Synthesis of Iodide **38** S54
1.2.43 Synthesis of Olefin **39** S55

1.2.44 Synthesis of Dihydrokoumine (**40**)........ S56
1.2.45 Synthesis of Koumine (**14**)........ S57
1.3 Spectral Data Comparison of Natural or/and Synthetic Products with Our Synthetic Products........ S58
1.3.1 Akuammidine (**1**)........ S58
1.3.2 19-(Z)-Akuammidine (**4**)........ S59
1.3.3 Polyneuridine (**2**)........ S60
1.3.4 Vincarine (**8**)........ S62
1.3.5 Vincamedine (**7**)........ S63
1.3.6 Vincamajine (**6**)........ S64
1.3.7 Quebrachidine (**5**)........ S65
1.3.8 Vincamajinine (**9**)........ S66
1.3.9 Alstiphyllanine J (**11**)·TFA........ S68
1.3.10 Koumidine (**25c**)........ S70
1.3.11 19-*Z*-Taberpsychine (**13**)........ S71
1.3.12 Dihydrokoumine (**40**)........ S73
1.3.13 Koumine (**14**)........ S74
1.5 Copies of ^{1}H and ^{13}C NMR Spectra........ S76
1.6 X-Ray Single Crystal Diffraction Data........ S185
6.1 X-Ray single crystal diffraction data for **16a**........ S185
6.2 X-Ray single crystal diffraction data for **25**........ S187
6.3 X-Ray single crystal diffraction data for Akuammidine (**1**)........ S189
6.4 X-Ray single crystal diffraction data for Quebrachidine (**5**)........ S191
6.5 X-Ray single crystal diffraction data for **25b**........ S194
2. Supplementary References........ S197

1. Supplementary Methods

1. 1 General Information

Melting points were measured on a Hanon MP 430 auto melting-point system and are uncorrected. The infrared (IR) spectra were recorded on a Nicolet iS10 FTIR spectrometer with 4 cm^{-1} resolution and 32 scans between wavenumber of 4000 cm^{-1} and 400 cm^{-1}. Samples were prepared as KBr disks with 1 mg of samples in 100 mg of KBr. ^{1}H-NMR and ^{13}C-NMR spectra were recorded on Bruker Avance 400, 500 and 600 spectrometers. Chemical shifts are reported in parts per million (δ) referenced to tetramethylsilane (0.0 ppm), chloroform (7.26 ppm or 77.0 ppm) and methanol (3.31 ppm or 49.0 ppm), respectively. Data for ^{1}H-NMR and ^{13}C-NMR spectroscopy are reported as follows: chemical shift (δ ppm), multiplicity (s = singlet, d = doublet, t = triplet, q = quartet, m = multiplet, br = broad), coupling constant (Hz), integration. High Resolution Mass spectra were taken on AB QSTAR Pulsar mass spectrometer or Aglient LC/MSD TOF mass spectrometer. Optical rotations were recorded on a JASCO P-2000 polarimeter. All new compounds were characterized by IR, ^{1}H NMR, ^{13}C NMR and HRMS. Silica gel (200–300 mesh) for column chromatography and silica GF_{254} for TLC were obtained from Merck Chemicals Co. Ltd. (Shanghai). Anhydrous THF was dried by distillation over metallic sodium and benzophenone; dichloromethane, pyridine and methanol were distilled from calcium hydride. Starting materials and reagents used in reactions were obtained commercially from Acros, Aldrich and Adamas-beta®, and were used without purification, unless otherwise indicated. All reactions were conducted in dried glassware under a positive pressure of dry nitrogen or argon. Reagents and starting materials were accordingly transferred via syringe or cannula. Reaction temperatures refer to the external oil bath temperature.

1.2 Experimental Section

1.2.1 Synthesis of PMB-protected Tryptophol S2

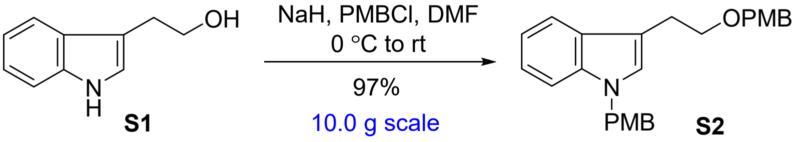

To a mixture of *p*-methoxybenzyl chloride (24.29 g, 155.07 mmol) and NaH (60% in oil, 9.93 g, 248.12 mmol) in DMF (120 mL) was added a solution of tryptophol **S1** (commercially available, 10.00 g, 62.03 mmol) in DMF (40 mL) at 0 °C. The resulting mixture was stirred at room temperature for 12 h. The reaction mixture was then quenched carefully with saturated *aq.* NH_4Cl (80 mL) at 0 ºC before being diluted with water (500 mL). The aqueous solution was extracted with EtOAc (4 × 250 mL). The combined organic phases were washed with brine (80 mL), dried over anhydrous Na_2SO_4, filtered and concentrated under reduced pressure. The residue was purified by flash column chromatography on silica gel (petroleum ether/EtOAc = 4:1) to afford PMB-protected tryptophol **S2** (24.10 g, 97%) as a colorless oil.

^{1}H NMR (400 MHz, $CDCl_3$): δ 7.64 (d, J = 7.6 Hz, 1H), 7.31-7.27 (m, 3H), 7.20 (td, J = 8.0, 0.8 Hz, 1H), 7.13 (td, J = 8.0, 0.8 Hz, 1H), 7.09 (d, J = 8.8 Hz, 2H), 6.97 (s, 1H), 6.89 (d, J = 8.4 Hz, 2H), 6.85 (d, J = 8.8 Hz, 2H), 5.21 (s, 2H), 4.52 (s, 2H), 3.83 (s, 3H), 3.79 (s, 3H), 3.78 (t, J = 7.2 Hz, 2H), 3.11 (t, J = 7.2 Hz, 2H).

^{13}C NMR (100 MHz, $CDCl_3$): δ 159.2, 159.1, 136.6, 130.7, 129.8, 129.4, 128.4, 128.3, 126.1, 121.7, 119.2, 119.0, 114.2, 113.9, 112.2, 109.7, 72.7, 70.5, 55.4, 49.4, 25.9.

NMR data were identical to those reported in the literature. [1]

1.2.2 Synthesis of Aldehyde 17a

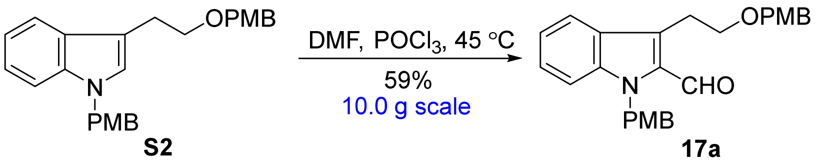

A solution of phosphoryl chloride (13.9 mL, 149.4 mmol) in DMF (120 mL) was stirred at 45 °C for 30 min. After being cooled to 0 °C, a solution of **S2** (10.00 g, 24.91 mmol) in DMF (30 mL) was added (ice bath). The resulting mixture was then stirred at 45 °C for 6 h. The reaction was quenched with *aq.* NaOH (6 N, 200 mL) at 0 °C before addition of saturated *aq.* NH_4Cl (500 mL). The resulting mixture was extracted with EtOAc (4 × 300 mL). The combined organic phases were washed with brine (100 mL), dried over anhydrous Na_2SO_4, filtered and concentrated under reduced pressure. The residue was purified by column chromatography on silica gel (petroleum ether/EtOAc = 5:1) to yield aldehyde **17a** (6.35 g, 59%) as a colorless oil.

^{1}H NMR (400 MHz, $CDCl_3$): δ 10.12 (s, 1H), 7.70 (d, *J* = 8.0 Hz, 1H), 7.36-7.33 (m, 2H), 7.16-7.11 (m, 3H), 7.03 (d, *J* = 8.4 Hz, 2H), 6.80 (d, *J* = 8.8 Hz, 2H), 6.74 (d, *J* = 8.8 Hz, 2H), 5.71 (s, 2H), 4.41 (s, 2H), 3.75 (s, 3H), 3.70 (t, *J* = 7.2 Hz, 2H), 3.69 (s, 3H), 3.37 (t, *J* = 6.8 Hz, 2H).

^{13}C NMR (100 MHz, $CDCl_3$): δ 181.9, 159.2, 158.8, 139.5, 131.3, 130.3, 129.1, 128.0, 127.98, 127.4, 126.7, 121.4, 120.6, 114.0, 113.8, 111.0, 72.8, 70.4, 55.3, 55.2, 47.3, 24.7.

NMR data were identical to those reported in the literature. [1]

1.2.3 Synthesis of Sulfinimine 17b

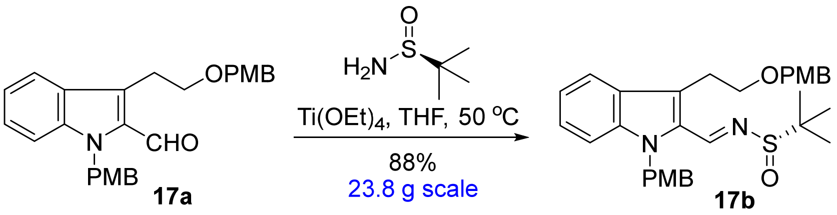

A mixture of aldehyde **17a** (23.81 g, 55.43 mmol), (*S*)-*tert*-butanesulfonamine (8.72 g, 71.95 mmol) and $Ti(OEt)_4$ (42.3 mL, 201.7 mmol) in THF (500 mL) was stirred at 50 °C for 12 h. After TLC analysis, the reaction was quenched with water (30 mL). The resulting suspension was filtered through a short column of silica gel and washed with ethyl acetate (ca. 800 mL). The organic layer was washed with brine (200 mL), dried over anhydrous Na_2SO_4, filtered, and concentrated under reduced pressure. The

residue was purified by column chromatography on silica gel (petroleum ether/EtOAc = 5:1) to afford sulfinimine **17b** (26.10 g, 88%) as a pale-yellow syrup.

R$_f$ = 0.57 (petroleum ether: ethyl acetate = 5:1).

$[\alpha]_D^{20}$ = +79.5 (*c* 1.00, $CHCl_3$).

FTIR (KBr, thin film) cm^{-1} : 2956, 2862, 2836, 1738, 1613, 1584, 1514, 1463, 1362, 1247, 1176, 1082, 1035, 820, 746.

^{1}H NMR (400 MHz, $CDCl_3$): δ 8.84 (s, 1H), 7.73 (d, *J* = 8.0 Hz, 1H), 7.33-7.27 (m 2H), 7.22 (d, *J* = 8.4 Hz, 2H), 7.17-7.13 (m, 1H), 6.87 (d, *J* = 8.4 Hz, 2H), 6.85-6.82 (m, 3H), 6.78-6.74 (m, 2H), 5.98 (d, *J* = 16.4 Hz, 1H), 5.70 (d, *J* = 16.4 Hz, 1H,), 4.46 (s, 2H), 3.79 (s, 3H), 3.73 (s, 3H), 3.73-3.70 (m, 2H), 3.40 (td, *J* = 7.2, 2.0 Hz, 2H), 1.08 (s, 9H).

^{13}C NMR (100 MHz, $CDCl_3$): δ 159.1, 158.7, 152.1, 139.7, 130.5, 130.2, 130.0, 129.2, 127.3, 127.2, 126.3, 124.1, 121.0, 120.4, 114.1, 113.8, 110.5, 72.7, 70.4, 57.5, 55.3, 47.5, 25.3, 22.3.

HRMS (ESI): Calcd for $C_{31}H_{36}N_2O_4SNa$ $[M+Na]^+$: 555.2288, found: 555.2284.

1.2.4 Synthesis of Sulfinamide 19a and Its Diastereomer 19a'

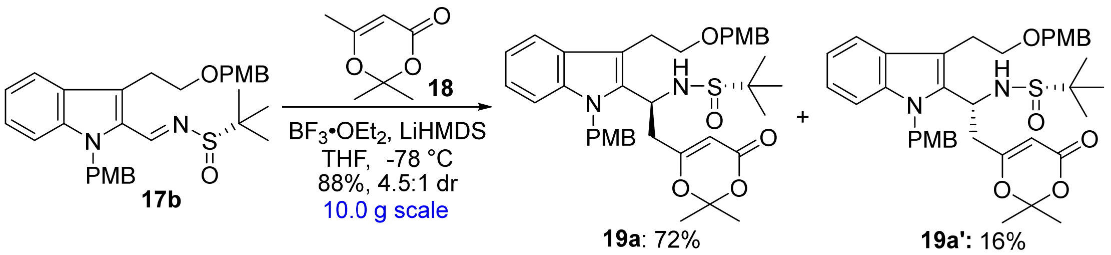

To a solution of sulfinimine **17b** (10.00 g, 18.77 mmol) in THF (150 mL) was added $BF_3{\cdot}Et_2O$ (5.9 mL, 46.76 mmol) at –78 °C. In the mean time, to a stirred solution of dioxinone **18** (12.01 g, 84.48 mmol) in THF (200 mL) was added lithium bis(trimethylsilyl)amide (1.0 M in THF, 84.5 mL, 84.5 mmol) at –78 °C. The resulting mixtures were stirred respectively at –78 °C for 1 h. The solution of dioxinone-derived lithium dienolate was then cannulated to the solution of sulfinimine at –78 °C. The reation mixture was allowed to stir at –78 °C for 3 h. After TLC analysis, the reaction was quenched with saturated *aq.* NH_4Cl (80 mL) at –78 °C. The

resulting mixture was then diluted with water (200 mL), and extracted with EtOAc (4 × 130 mL). The combined organic phases were washed with brine (100 mL), dried over anhydrous Na_2SO_4, filtered, and concentrated under reduced pressure. The residue was purified by column chromatography on silica gel (petroleum ether/EtOAc = 3:1 to 1:1) to give sulfinamide **19a** (9.12 g, 72%, petroleum ether/EtOAc = 2:1) and its diasteromer **19a'** (1.96 g, 16%, petroleum ether/EtOAc = 1:1) both as white solids.

19a:

M.p.: 60-63 °C.

R*f* = 0.34 (petroleum ether : ethyl acetate = 2:1).

$[\alpha]_D^{20}$ = +19.5 (*c* 1.04, $CHCl_3$).

FTIR (KBr, thin film) cm^{-1} : 3439, 2955, 2864, 1729, 1635, 1613, 1514, 1465, 1390, 1375, 1248, 1176, 1073, 1033, 819, 742.

^{1}H NMR (400 MHz, MeOD): δ 7.50 (d, *J* = 8.0 Hz, 1H), 7.27 (br s, 1H), 7.15-7.10 (m, 3H), 7.03 (t, *J* = 7.6 Hz, 1H), 6.89 (d, *J* = 8.4 Hz, 2H), 6.81 (d, *J* = 8.4 Hz, 2H), 6.75 (d, *J* = 8.0 Hz, 2H), 5.54-5.43 (m, 2H), 4.98 (br s, 2H), 4.44-4.37 (m, 2H), 3.75 (t, *J* = 6.0 Hz, 2H), 3.72 (s, 3H), 3.71 (s, 3H), 3.23 (br s, 1H), 3.19-3.14 (m, 1H), 2.94 (dd, *J* = 14.4, 8.8 Hz, 1H), 2.58 (br s, 1H), 1.51 (s, 3H), 1.49 (s, 3H), 1.12 (s, 9H).

^{13}C NMR (100 MHz, MeOD): δ 169.6, 162.9, 160.7, 160.5, 138.5, 136.3, 131.5, 131.3, 130.6, 128.9, 128.3, 123.6, 120.5, 119.8, 115.3, 114.7, 112.6, 110.9, 108.1, 96.2, 73.7, 71.0, 57.6, 55.7, 55.6, 50.7, 47.5, 41.8, 26.5, 25.3, 24.9, 23.0.

HRMS (ESI): Calcd for $C_{38}H_{46}N_2O_7SNa$ $[M+Na]^+$: 697.2918, found: 697.2916.

19a':

M.p.: 52-56 °C.

R*f* = 0.22 (petroleum ether : ethyl acetate = 1:1).

$[\alpha]_D^{20}$ = +62.8 (*c* 1.02, $CHCl_3$).

FTIR (KBr, thin film) cm^{-1} : 3432, 2957, 1728, 1632, 1613, 1514, 1465, 1248, 1177, 1071, 1035, 821, 743.

^{1}H NMR (400 MHz, DMSO): δ 7.53 (d, *J* = 7.6 Hz, 1H), 7.23 (d, *J* = 8.4 Hz, 2H), 7.08-6.99 (m, 3H), 6.88-6.82 (m, 6H), 5.62 (br s, 1H), 5.43 (br d, *J* = 14.4 Hz, 1H),

5.28 (d, J = 3.2 Hz, 1H), 4.96 (br s, 2H), 4.44 (s, 2H), 3.73 (s, 3H), 3.73-3.69 (m, 1H), 3.69 (s, 3H), 3.59 (br s, 1H), 3.04 (dd, J = 14.4, 6.8 Hz, 2H), 2.61 (dd, J = 14.4, 7.6 Hz, 1H), 1.47 (s, 6H), 1.03 (s, 9H).

^{13}C NMR (100 MHz, DMSO): δ 167.4, 159.8, 158.6, 158.3, 130.5, 130.0, 129.0, 127.1, 121.9, 119.0, 118.9, 113.9, 113.6, 110.2, 106.3, 94.6, 71.6, 69.8, 55.08, 55.04, 54.99, 47.8, 24.8, 23.9, 22.3.

HRMS (ESI): Calcd for $C_{38}H_{46}N_2O_7SNa$ $[M+Na]^+$: 697.2918, found: 697.2921.

1.2.5 Synthesis of Alcohol 19b

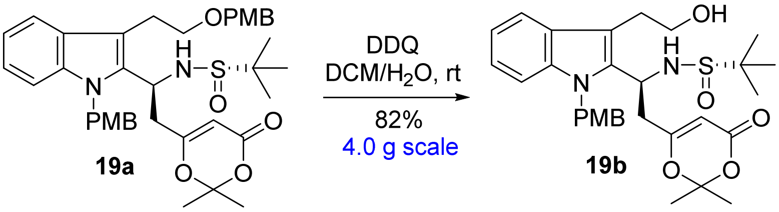

To a solution of sulfinamide **19a** (3.91 g, 5.79 mmol) in CH_2Cl_2/H_2O (10:1, 77 mL) was added DDQ (1.71 g, 7.54 mmol). The resulting mixture was then stirred at room temperature under air for 12 h. After TLC analysis, the reaction mixture was filtered through a short column of silica gel (200–300 mesh) and washed with dichloromethane (160 mL). The organic layer was washed with brine (30 mL), dried over anhydrous Na_2SO_4, filtered, and concentrated under reduced pressure. The residue was purified by flash column chromatography on silica gel (petroleum ether/EtOAc = 2:1) to provide alcohol **19b** (2.62 g, 82%) as a white solid.

M.p.: 81-84 °C.

R*f* = 0.26 (petroleum ether : ethyl acetate = 2:1).

$[\alpha]_D^{20}$ = +10.5 (*c* 1.00, $CHCl_3$).

FTIR (KBr, thin film) cm^{-1} : 3435, 2956, 1728, 1634, 1514, 1464, 1391, 1376, 1273, 1249, 1204, 1178, 1039, 1015, 810, 742.

^{1}H NMR (400 MHz, MeOD): δ 7.57 (d, J = 7.6 Hz, 1H), 7.32 (br s, 1H), 7.16 (t, J = 7.6 Hz, 1H), 7.07 (t, J = 7.6 Hz, 1H), 6.93 (d, J = 8.0 Hz, 2H), 6.84 (d, J = 7.6 Hz, 2H), 5.60 (d, J = 17.6 Hz, 1H), 5.47 (d, J = 17.6 Hz, 1H), 5.16 (br s, 1H), 4.99 (br s,

1H), 3.91-3.78 (m, 2H), 3.73 (s, 3H), 3.17-3.11 (m, 3H), 2.58 (br s, 1H), 1.54 (s, 3H), 1.48 (s, 3H), 1.16 (s, 9H).

^{13}C NMR (100 MHz, MeOD): δ 169.8, 163.0, 160.5, 138.6, 136.8, 131.7, 128.9, 128.4, 123.6, 120.5, 119.7, 115.4, 112.6, 110.9, 108.1, 96.2, 62.9, 57.8, 55.7, 50.7, 47.4, 41.4, 29.1, 25.4, 24.8, 23.2.

HRMS (ESI): Calcd for $C_{30}H_{38}N_2O_6SNa$ $[M+Na]^+$: 577.2343, found: 577.2348.

1.2.6 Synthesis of Amide 19d

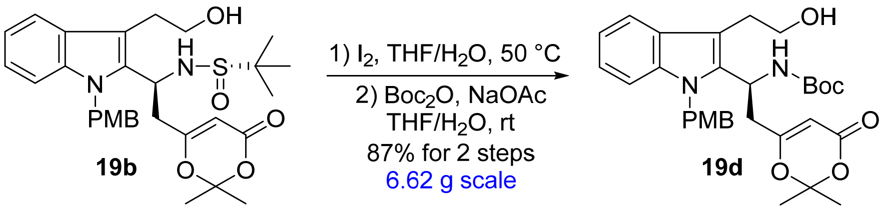

To a solution of sulfinamide **19b** (6.62 g, 11.93 mmol) in THF/H_2O (5:1, 150 mL) was added iodine (0.61 g, 2.39 mmol). The dark-purple mixture was then stirred at 50 °C under air. After the sulfinamide being completely consumed (TLC), triethylamine (7 mL) was added at room temperature. The resulting mixture was then extracted with EtOAc (3 × 80 mL). The combined organic phases were washed with brine (80 mL) and dried over Na_2SO_4. After removal of the solvents under reduced pressure, the resulting residue was directly dissolved in THF/H_2O (3:1, 160 mL) at room temperature, and Boc_2O (3.91 g, 17.90 mol) and NaOAc (1.96 g, 23.86 mmol) were added. The reaction mixture was stirred at room temperature for 2 h before being quenched with saturated *aq.* NH_4Cl (100 mL). The aqueous layer was extracted with EtOAc (4 × 75 mL). The combined organic phases were washed with brine (80 mL) and dried over anhydrous Na_2SO_4. After removal of the solvents under reduced pressure, the residue was purified by column chromatography on silica gel (petroleum ether/EtOAc = 3:1-2:1) to give amide **19d** (5.70 g, 87%) as a white solid.

M.p.: 77-79 °C.

R_f = 0.45 (petroleum ether : ethyl acetate = 1:1).

$[\alpha]_D^{20}$ = –68.4 (*c* 1.00, $CHCl_3$).

FTIR (KBr, thin film) cm^{-1} : 3432, 2933, 1709, 1633, 1514, 1465, 1392, 1377, 1248, 1175, 1015, 808, 741.

^{1}H NMR (400 MHz, MeOD): δ 7.56 (d, *J* = 8.0 Hz, 1H), 7.26 (d, *J* = 8.0 Hz, 1H), 7.12 (t, *J* = 7.6 Hz, 1H), 7.05 (t, *J* = 7.6 Hz, 1H), 6.91 (d, *J* = 8.4 Hz, 2H), 6.79 (d, *J* = 8.8 Hz, 2H), 5.47 (br s, 2H), 5.32-5.29 (m, 1H), 5.23 (s, 1H), 3.89-3.81 (m, 1H), 3.79-3.74 (m, 1H), 3.71 (s, 3H), 3.12 (t, *J* = 6.8 Hz, 2H), 2.91 (dd, *J* = 14.8, 10.8 Hz, 1H), 2.61 (dd, *J* = 14.8, 4.8 Hz, 1H), 1.57 (s, 3H), 1.56 (s, 3H), 1.35 (s, 9H).

^{13}C NMR (100 MHz, MeOD): δ 170.1, 163.3, 160.3, 157.2, 138.2, 137.0, 131.7, 129.2, 128.5, 123.2, 120.4, 119.6, 115.1, 111.3, 110.8, 108.2, 95.8, 80.5, 63.3, 55.7, 47.2, 45.7, 39.8, 28.9, 28.7, 25.7, 24.3.

HRMS (ESI): Calcd for $C_{31}H_{38}N_2O_7Na$ $[M+Na]^+$: 573.2571, found: 573.2573.

1.2.7 Synthesis of Amide 16a

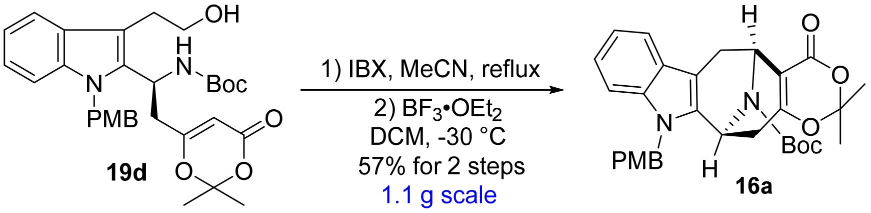

19d **16a**

To a solution of **19d** (1.10 g, 2.00 mmol) in dry MeCN (25 mL) was added IBX (840.1 mg, 3.00 mmol). The reaction mixture was allowed to stir at reflux for 40 min under Ar. After being cooled to room temperature, the mixture was filtered through a short column of celite and washed with dry MeCN (ca. 50 mL), the filtrate was concentrated under reduced pressure to give the crude aldehyde which was used for the next step without further purification. The crude aldehyde was dissolved in dry DCM (25 mL), and BF_3•Et_2O (303 μL, 2.40 mmol) was added at –30 °C. After being stirred at –30 °C for 40 min, the reaction was quenched with saturated *aq.* $NaHCO_3$ (10 mL) and diluted with water (50 mL). The aqueous layer was then extracted with DCM (3 × 25 mL). The combined organic phases were washed with brine (30 mL) and dried over anhydrous Na_2SO_4. After removal of the solvents under reduced pressure, the residue was purified by column chromatography on silica gel (petroleum

ether/EtOAc = 3:1) to yield amide **16a** (0.61 g, 57%) as a white solid.

M.p.: 180-183 °C.

R*f* = 0.42 (petroleum ether : ethyl acetate = 2:1).

$[\alpha]_D^{20}$ = −100.4 (*c* 1.21, $CHCl_3$).

^{1}H NMR (400 MHz, $CDCl_3$): spectrum appears as a mixture of rotamers, δ 7.51 (d, *J* = 7.2 Hz, 1H), 7.24-7.10 (m, 3H), 6.94-6.88 (m, 2H), 6.81 (d, *J* = 8.8 Hz, 2H), 5.68 (d, *J* = 5.2 Hz, 0.75H), 5.55 (d, *J* = 5.2 Hz, 0.25H), 5.41 (d, *J* = 5.2 Hz, 1H), 5.33 (d, *J* = 16.8 Hz, 1H), 5.19 (d, *J* = 16.8 Hz, 1H), 3.77 (s, 3H), 3.28-3.19 (m, 1H), 3.05 (d, *J* = 15.6 Hz, 1H), 2.81-2.72 (m, 1H), 2.16 (d, *J* = 18.0 Hz, 0.25H), 1.94 (d, *J* = 18.0 Hz, 0.75H), 1.63 (s, 3H), 1.49 (s, 6.75H), 1.46 (s, 3H), 1.36 (s, 2.25H).

^{13}C NMR (100 MHz, $CDCl_3$): δ 163.7 (162.6), 160.0 (159.6), 159.2, 153.8 (153.0), 137.1, 133.6 (133.1), 129.3, 127.4, 127.0, 122.2, 119.7, 118.7 (118.9), 114.4, 109.9 (109.6), 106.9 (107.9), 106.3, 103.9 (104.3), 81.2 (80.9), 55.4, 46.4, 45.7 (45.1), 43.8 (44.5), 33.1 (33.6), 28.5 (28.3), 26.0 (25.3), 25.8 (26.3), 24.6 (24.2).

FTIR (KBr, thin film) cm^{-1} : 3424, 2977, 2929, 1727, 1689, 1649, 1515, 1462, 1403, 1320, 1298, 1254, 1175, 1137, 1036, 1012, 781, 763.

HRMS (ESI): Calcd for $C_{31}H_{34}N_2O_6Na$ $[M+Na]^+$: 553.2309, found: 553.2312.

1.2.8 Synthesis of Methyl Ester 20a

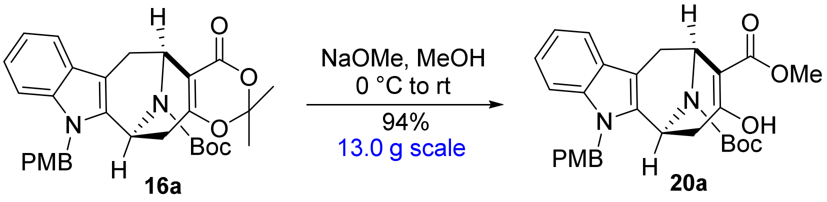

Sodium (2.84 g, 123.25 mmol) was added portionwise to methanol (300 mL) at 0 °C. After addition, the reaction flask was removed from the ice bath and warmed up to room temperature. After being stirred at room temperature for 30 min, compound **16a** (13.08 g, 24.65 mmol) was introduced to the flask in one portion, and the reaction mixture was then stirred for 6 h. After TLC analysis, saturated *aq.* NH_4Cl (80 mL) was added to the reaction mixture at 0 °C, and the resulting suspension was filtered through a Büchner funnel. The solid cake was washed with H_2O (3 × 150 mL), and

dried under reduced pressure to yield methyl ester **20a** (11.69 g, 94%) as a white solid.

M.p.: 187-189 °C.

$\mathbf{R}_f$ = 0.56 (petroleum ether : ethyl acetate = 1:1).

$[\alpha]_D^{20}$ = –101.0 (*c* 0.99, $CHCl_3$).

FTIR (KBr, thin film) cm^{-1} : 3435, 2974, 2936, 2851, 1698, 1655, 1616, 1513, 1463, 1442, 1400, 1341, 1319, 1271, 1249, 1230, 1176, 1064, 826, 750.

^{1}H NMR (400 MHz, $CDCl_3$): spectrum appears as a mixture of rotamers, δ 12.1 (s, 1H), 7.51 (t, *J* = 8.0 Hz, 1H), 7.49-6.98 (m, 3H), 6.97 (d, *J* = 8.4 Hz, 1H), 6.93 (d, *J* = 8.4 Hz, 1H), 6.83 (d, *J* = 8.4 Hz, 2H), 5.65 (d, *J* = 5.2 Hz, 0.5H), 5.51 (d, *J* = 5.2 Hz, 0.5H), 5.36-5.14 (m, 3H), 3.87 (s, 1.5H), 3.83 (s, 1.5H), 3.77 (s, 3H), 3.27-3.18 (m, 1H), 2.91-2.86 (m, 2H), 2.78 (d, *J* = 17.6 Hz, 0.5H), 2.11 (d, *J* = 17.6 Hz, 0.5H), 1.51 (s, 4.5H), 1.38 (s, 4.5H).

^{13}C NMR (100 MHz, $CDCl_3$): δ 171.2 (171.1), 170.6 (169.6), 159.2, 153.7 (153.1), 137.2, 134.5 (133.9), 129.3, 127.44 (127.35), 127.2, 122.1 (122.0), 119.7 (119.6), 118.5 (118.3), 114.5, 110.1 (109.7), 107.4 (106.5), 100.6 (100.0), 80.8 (80.7), 55.3, 51.8, 46.5 (46.4), 45.9 (45.1), 44.5 (43.7), 34.6 (34.2), 28.5 (28.4), 27.1 (26.6).

HRMS (ESI): Calcd for $C_{29}H_{32}N_2O_6Na$ $[M+Na]^+$: 527.2153, found: 527.2153.

1.2.9 Synthesis of Ester 20b

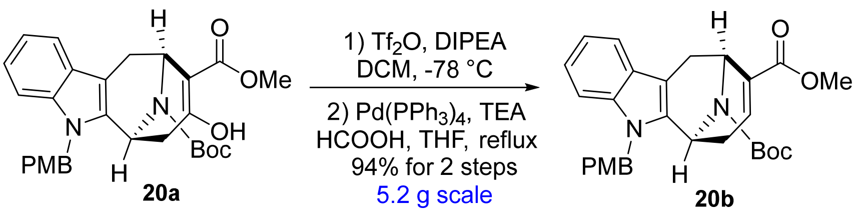

To a solution of enol **20a** (5.24 g, 10.38 mmol) in anhydrous DCM (100 mL) was added DIPEA (7.3 mL, 41.6 mmol) at –78 °C. After being stirred at –78 °C for 10 min, Tf_2O (3.5 mL, 20.8 mmol) was introduced. The reaction mixture was then stirred at –78 °C for 1 h before being quenched with saturated *aq.* $NaHCO_3$ (100 mL). The aqueous layer was extracted with DCM (3 × 80 mL), and the combined organic

phases were dried over anhydrous Na_2SO_4. After removal of the solvents under reduced pressure, the residue was purified by column chromatography on silica gel (petroleum ether/EtOAc = 5:1) to give triflate (6.41 g, 97%) which was used directly in the next reaction without purification. To a solution of triflate (6.41 g, 10.07 mmol) in THF (130 mL) were added $Pd(PPh_3)_4$ (1.17 g, 1.01 mmol), TEA (14.2 mL, 101 mmol) and HCO_2H (3.8 mL, 101 mmol). The resulting mixture was then stirred at reflux for 12 h. After TLC analysis, the mixture was filtered through a short column of silica gel and washed with EtOAc (20 mL). The filtrate was concentrated under reduced pressure and the residue was purified by flash column chromatography on silica gel (PE/EtOAc 5:1) to afford ester **20b** (4.78 g, 94% from **20a**) as a white solid.

M.p.: 85-87 °C.

$\mathbf{R}_f$ = 0.35 (petroleum ether : ethyl acetate = 5:1).

$[\alpha]_D^{20}$ = –98.5 (*c* 1.07, $CHCl_3$).

FTIR (KBr, thin film) cm^{-1} : 3434, 2975, 2931, 1714, 1695, 1514, 1463, 1317, 1248, 1175, 1107, 1125, 1034, 741.

^{1}H NMR (400 MHz, $CDCl_3$): spectrum appears as a mixture of rotamers, δ 7.49 (t, *J* = 8.0 Hz, 1H), 7.24-7.08 (m, 3H), 6.96 (d, *J* = 8.4 Hz, 1H), 6.92-6.87 (m, 2H), 6.81 (d, *J* = 8.8 Hz, 2H), 5.61 (d, *J* = 5.6 Hz, 0.6H), 5.51 (d, *J* = 5.6 Hz, 0.4H), 5.34 (br s, 1H), 5.30-5.16 (m, 2H), 3.77 (s, 3H), 3.76 (s, 3H), 3.32-3.20 (m, 1H), 2.92 (d, *J* = 16.0 Hz, 1H), 2.79-2.69 (m, 1H), 2.21 (dd, *J* = 18.8, 4.8 Hz, 0.4H), 2.00 (dd, *J* = 18.8, 4.8 Hz, 0.6H), 1.49 (s, 5.4H), 1.36 (s, 3.6H).

^{13}C NMR (100 MHz, $CDCl_3$): δ 165.7 (165.5), 159.1, 153.9 (153.3), 137.2, 137.0, 136.3, 134.9 (134.4), 131.5 (132.1), 129.6, 127.5 (127.3), 127.1, 121.9 (122.0), 119.56 (119.63), 118.3 (118.6), 114.4, 109.9 (109.6), 106.7 (107.6), 80.7 (80.4), 55.4, 51.9, 47.5 (46.1), 46.5 (46.4), 43.3 (44.8), 31.4 (31.8), 28.6 (28.4), 26.2 (25.7).

HRMS (ESI): Calcd for $C_{29}H_{32}N_2O_5Na$ $[M+Na]^+$: 511.2203, found: 511.2208.

1.2.10 Synthesis of Tertiary Amine 20c

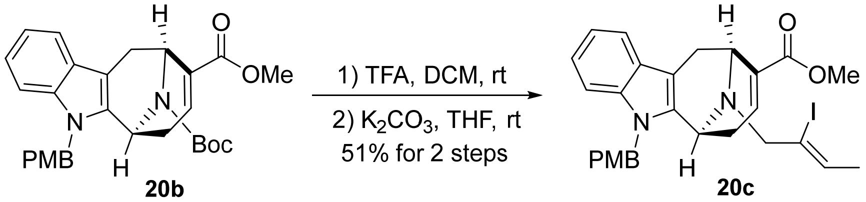

To a solution of ester **20b** (350.0 mg, 0.72 mmol) in DCM (4.5 mL) was added trifluoroacetic acid (1.5 mL). The reaction mixture was stirred at room temperature for 5 min before being quenched with saturated *aq.* $NaHCO_3$ (50 mL). The mixture was then extracted with DCM (3 × 10 mL). The combined organic phases were washed with brine (10 mL), dried over anhydrous Na_2SO_4, filtered and concentrated. The resulting residue was dissolved in THF (10 mL). To this mixture, K_2CO_3 (149.3 mg, 1.08 mmol) and (Z)-1-bromo-2-iodobute-2-ene (375.7 mg, 1.44 mmol) were added at room temperature under Ar. The reaction mixture was stirred at room temperature for 48 h before being quenched with saturated *aq.* NH_4Cl (30 mL). The resulting mixture was extracted with EtOAc (3 × 10 mL). The combined organic phases were washed with brine, dried over anhydrous Na_2SO_4, filtered and concentrated under reduced pressure. The residue was purified by flash column chromatography on silica gel (petroleum ether/EtOAc = 3:1) to provide amine **20c** (208.1 mg, 51%) as a yellowish solid.

M.p.: 73-75 °C.

R*f* = 0.35 (petroleum ether : ethyl acetate = 4:1).

$[\alpha]_D^{20}$ = –122.8 (*c* 0.22, $CHCl_3$).

FTIR (KBr, thin film) cm^{-1} : 3440, 2918, 1709, 1645, 1613, 1512, 1463, 1437, 1248, 1175, 1036, 825, 741.

^{1}H NMR (400 MHz, $CDCl_3$): δ 7.41 (d, *J* = 7.6 Hz, 1H), 7.13 (d, *J* = 7.2 Hz, 1H), 7.06-6.99 (m, 2H), 6.82 (d, , *J* = 3.2 Hz, 1H), 6.76 (d, *J* = 8.8 Hz, 2H), 6.69 (d, *J* = 8.4 Hz, 2H), 5.42-5.41 (m , 1H), 5.17 (d, *J* = 16.8 Hz, 1H), 5.00 (d, *J* = 16.8 Hz, 1H), 4.03 (d, *J* = 5.2 Hz, 1H), 3.83 (d, *J* = 5.6 Hz, 1H), 3.66 (s, 3H), 3.64 (s, 3H), 3.29 (d, *J* = 13.6 Hz, 1H), 3.11 (d, *J* = 13.6 Hz, 1H), 3.04 (dd, *J* = 16.4, 5.6 Hz, 1H), 2.75-2.68 (m,

2H), 2.06 (dd, J = 18.4, 5.6 Hz, 1H), 1.56 (d, J = 6.4 Hz, 3H).

^{13}C NMR (100 MHz, $CDCl_3$): δ 166.1, 159.0, 137.0, 136.9, 134.3, 132.9, 132.5, 129.7, 127.2, 121.7, 119.4, 118.4, 114.3, 109.5, 109.0, 106.2, 63.9, 55.3, 51.7, 46.9, 46.1, 32.3, 22.6, 21.8.

HRMS (ESI): Calcd for $C_{28}H_{30}IN_2O_3$ $[M+H]^+$: 569.1296, found: 569.1297.

1.2.11 Studies on the reductive Heck-type reaction

Supplementary Table 1 Some attempted conditions for reductive Heck-type reaction

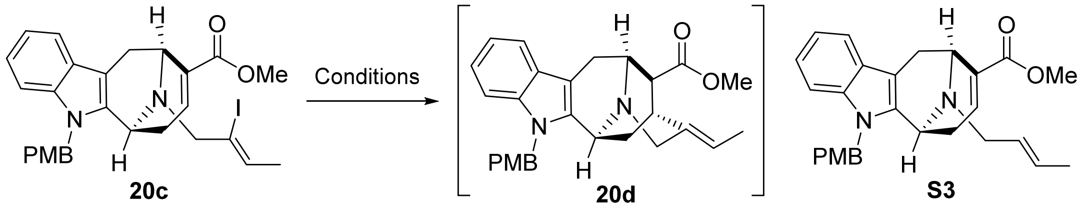

Entry	Conditions	Results[a]
1	$Ni(COD)_2$ (5 equiv), Et_3N (10 equiv), Et_3SiH (2 equiv), MeCN, rt	**S3**: 52%
2	$Ni(COD)_2$ (5 equiv), Et_3N (10 equiv), BHT (2 equiv), MeCN, rt	**S3**:67%
3	Bu_3SnH (5 equiv), AIBN (30 mol %), benzene, reflux	**S3**:41%
4	$Pd(PPh_3)_2Cl_2$ (10 mol %), PPh_3 (20 mol %), CsF (3 equiv), THF, reflux	**S3**:51%
5	SmI_2 (3 equiv), HMPA (12 equiv), THF, rt	**S3**:33%

[a]Isolated yield after purification by silica gel chromatography.

1.2.12 Synthesis of Tertiary Amine 22

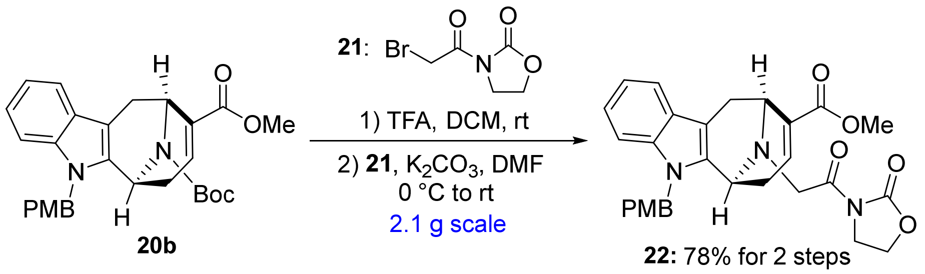

To a solution of compound **20b** (2.10 g, 4.30 mmol) in DCM (27 mL) was added trifluoroacetic acid (9 mL). The reaction mixture was then stirred at room temperature for 5 min before being quenched with saturated *aq.* $NaHCO_3$ (200 mL). The aqueous layer was extracted with DCM (3 × 60 mL). The combined organic phases were washed with brine (100 mL) and dried over Na_2SO_4. After removal of the solvent under reduced presure, the residue was dissolved in DMF (30 mL), and K_2CO_3 (891.1

mg, 6.45 mmol) was added at 0 °C under Ar. After being stirred at 0 °C for 30 min, **21**[2] (1.34 g, 6.45 mmol) was introduced. The mixture was then stirred at room temperature for 6 h. After TLC analysis, the reaction was quenched with saturated *aq.* NH_4Cl (60 mL). The mixture was extracted with EtOAc (4 × 50 mL). The combined organic phases were washed with brine (80 mL), dried over anhydrous Na_2SO_4. After removal of the solvents under reduced pressure, the residue was purified by column chromatography on silica gel (petroleum ether/EtOAc = 1:1-1:3) to afford amine **22** (1.73 g, 78%) as a yellowish solid.

M.p.: 110-113 °C.

$\mathbf{R}_f$ = 0.32 (petroleum ether : ethyl acetate = 1:2).

$[\alpha]_D^{20}$ = –182.8 (*c* 0.61, $CHCl_3$).

FTIR (KBr, thin film) cm^{-1} : 3436, 2920, 2840, 1779, 1709, 1612, 1513, 1388, 1264, 1247, 1197, 1038, 744.

^{1}H NMR (400 MHz, $CDCl_3$): δ 7.50 (d, *J* = 7.6 Hz, 1H), 7.24 (d, *J* = 8.0 Hz, 1H), 7.15 (t, *J* = 8.0 Hz, 1H), 7.10 (t, *J* = 7.6 Hz, 1H), 6.92 (br d, *J* = 3.2 Hz, 1H), 6.75 (br s, *J* 4H), 5.30 (d, *J* = 16.8 Hz, 1H), 5.09 (d, *J* = 16.8 Hz, 1H), 4.37 (t, *J* = 8.0 Hz, 2H), 4.25 (d, *J* = 5.6 Hz, 1H), 4.12 (d, *J* = 5.6 Hz, 1H), 4.00 (d, *J* = 18.4 Hz, 1H), 3.93-3.88 (m, 1H), 3.84-3.77 (m, 2H), 3.73 (s, 3H), 3.72 (s, 3H), 3.22 (dd, *J* = 16.8, 6.0 Hz, 1H), 2.95 (dd, *J* = 19.2, 5.6 Hz, 1H), 2.81 (d, *J* = 16.8 Hz, 1H), 2.15 (dd, *J* = 18.8, 5.2 Hz, 1H).

^{13}C NMR (100 MHz, $CDCl_3$): δ 170.2, 165.9, 158.9, 153.3, 137.5, 136.5, 133.9, 132.6, 130.1, 127.23, 127.15, 121.9, 119.5, 118.6, 114.2, 109.5, 106.1, 62.6, 55.4, 55.3, 52.3, 51.7, 49.1, 46.1, 42.2, 31.9, 22.8.

HRMS (ESI): Calcd for $C_{29}H_{30}N_3O_6$ $[M+H]^+$: 516.2129, found: 516.2134.

1.2.13 Synthesis of Ketone 23

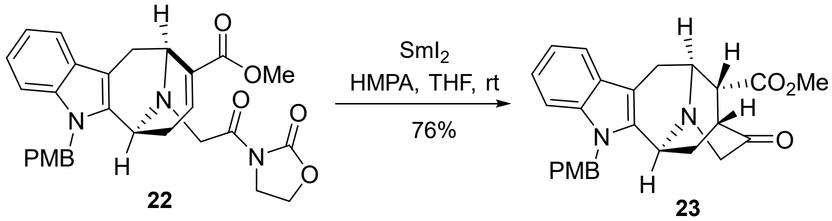

HMPA (1.75 mL, 10.0 mmol) was added to a solution of compound **22** (515.6 mg, 1 mmol) in THF (20 mL) at room temperature. To this solution, SmI_2 (0.1M in THF, 40 mL, 40 mmol) was added dropwise via syringe under Ar, and the color of the reaction mixture eventually changed to purple. The reaction was then quenched with saturated *aq.* NH_4Cl (30 mL). The mixture was extracted with EtOAc (3 × 20 mL). The combined organic phases were washed with brine (30 mL), dried over anhydrous Na_2SO_4, filtered and concentrated. The residue was purified by flash column chromatography on silica gel (petroleum ether/EtOAc = 1:3) to yield alcohol **23** (327.7 mg, 76%) as yellowish solid.

M.p.: 90-92 °C.

$\mathbf{R}_f$ = 0.76 (petroleum ether/EtOAc = 1:3).

$[\alpha]_D^{20}$ = –69.0 (*c* 1.04, $CHCl_3$).

FTIR (KBr, thin film) cm^{-1} : 3440, 2930, 2839, 1740, 1612, 1513, 1465, 1438, 1349, 1247, 1175, 1123, 1109, 1031, 819, 743.

^{1}H NMR (400 MHz, $CDCl_3$): δ 7.55 (d, *J* = 7.2 Hz, 1H), 7.29 (d, *J* = 8.0 Hz, 1H), 7.20 (td, *J* = 8.0 Hz, 1H), 7.15 (td, *J* = 7.2 Hz, 1H), 6.96 (d, *J* = 8.8 Hz, 2H), 6.80 (d, *J* = 8.8, 2.4 Hz, 2H), 5.23 (d, *J* = 16.8 Hz, 1H), 5.17 (d, *J* = 16.8 Hz, 1H), 4.21 (dd, *J* = 10.0, 2.4 Hz, 1H), 3.76 (s, 3H), 3.69 (s, 3H), 3.68 (d, *J* = 10.0 Hz, 1H), 3.51 (d, *J* = 2.4 Hz, 1H), 3.27 (dd, *J* = 16.0, 5.6 Hz, 1H), 2.95-2.88 (m, 2H), 2.59-2.58 (m, 1H), 2.14 (ddd, *J* = 13.6, 10.0, 2.0 Hz, 1H), 1.79 (dt, *J* = 13.6, 3.6 Hz, 1H).

^{13}C NMR (100 MHz, $CDCl_3$): δ 214.1, 173.9, 159.3, 138.2, 137.5, 129.4, 127.5, 127.2, 122.1, 119.7, 118.6, 114.5, 109.7, 103.5, 62.3, 55.4, 54.8, 52.6, 47.63, 47.55, 46.5, 41.8, 31.5, 27.4.

HRMS (ESI): Calcd for $C_{26}H_{27}N_2O_4$ $[M+H]^+$: 431.1965, found: 431.1969.

1.2.14 Synthesis of Olefins 24 and 25

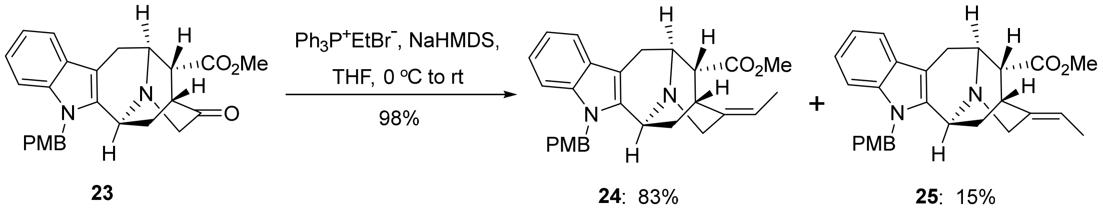

NaHMDS (1.0 M in THF, 2.1 mL, 2.1 mmol) was added to a solution of ethyltriphenylphosphonium bromide (862.1 mg, 2.32 mmol, which was dried under high vacuum for 5 h) in THF (20 mL) at 0 ºC under Ar. The resultant orange solution was stirred at 0 ºC for 30 min, then a solution of ketone **23** (200.0 mg, 0.465 mmol) in THF (7 mL) was added. The mixture was stirred at room temperature for a further 4 h before being quenched with saturated *aq.* NH_4Cl (3 mL). The mixture was diluted with water (15 mL) and extracted with EtOAc (3 × 8 mL). The combined organic phases were washed with brine (20 mL), dried over Na_2SO_4, filtered, and concentrated. The residue was purified by column chromatography on silica gel (petroleum ether/EtOAc = 2:1-1:1) to yield olefin **25** (29.6 mg, 15%, petroleum ether/EtOAc = 2:1) as a white solid, and olefin **24** (168.5 mg, 83%, petroleum ether/EtOAc = 1:1) as a white solid.

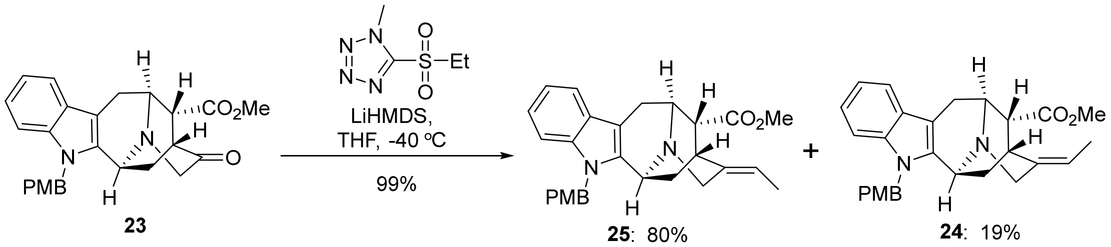

To a stirred solution of ketone **23** (200.0 mg, 0.465 mmol) and 1-methyl-1*H*-tetrazol-5-yl ethyl sulfone (204.5 mg, 1.16 mmol) in THF (10 mL) at –40 ºC under Ar was added LiHMDS (1.0 M in THF, 0.93 mL, 0.93 mmol). After 30 min and 1 h, LiHMDS (1.0 M in THF, 0.19 mL, 0.19 mmol) was added respectively. The mixture was stirred at –40 ºC for a further 30 min before being quenched with

saturated *aq.* NH_4Cl (3 mL). The mixture was diluted with water (15 mL) and extracted with EtOAc (3 × 8 mL). The combined organic phases were washed with brine (20 mL), dried over Na_2SO_4, filtered, and concentrated. The residue was purified by column chromatography on silica gel (petroleum ether/EtOAc = 2:1-1:1) to yield olefin **25** (164.5 mg, 80%, petroleum ether/EtOAc = 2:1) as a white solid, and olefin **24** (39.2 mg, 19%, petroleum ether/EtOAc = 1:1) as a white solid.

24:

M.p.: 182-183 °C.

R*f* = 0.35 (petroleum ether : ethyl acetate = 1:1).

$[\alpha]_D^{20}$ = –73.7 (*c* 0.35, $CHCl_3$).

FTIR (KBr, thin film) cm^{-1} : 3429, 2932, 1727, 1611, 1510, 1467, 1439, 1382, 1346, 1269, 1245, 1177, 1028, 812, 762.

^{1}H NMR (400 MHz, $CDCl_3$): δ 7.52 (d, *J* = 7.2 Hz, 1H), 7.24 (d, *J* = 8.0 Hz, 1H), 7.15 (t, *J* = 7.2 Hz, 1H), 7.09 (t, *J* = 7.2 Hz, 1H), 6.95 (d, *J* = 8.4 Hz, 2H), 6.79 (d, *J* = 8.4 Hz, 2H), 5.31 (q, *J* = 4.8 Hz, 1H), 5.24 (d, *J* = 16.8 Hz, 1H), 5.15 (d, *J* = 16.8 Hz, 1H), 4.12 (d, *J* = 9.2 Hz, 1H), 3.75 (s, 3H), 3.69-3.67 (m, 1H), 3.67 (s, 3H), 3.64 (d, *J* = 16.8 Hz, 1H), 3.54 (d, *J* = 16.8 Hz, 1H), 3.18 (dd, *J* = 15.6, 4.8 Hz, 1H), 3.13 (br s, 1H), 2.73 (d, *J* = 15.6 Hz, 1H), 2.55 (d, *J* = 7.6 Hz, 1H), 1.89 (t, *J* = 11.2 Hz, 1H), 1.58-1.57 (m, 1H), 1.57 (d, *J* = 6.4 Hz, 3H).

^{13}C NMR (100 MHz, $CDCl_3$): δ 174.2, 159.0, 139.3, 137.3, 134.5, 129.7, 127.6, 127.5, 121.5, 119.4, 118.4, 117.0, 114.3, 109.6, 104.1, 56.3, 55.4, 52.9, 51.8, 49.1, 47.1, 46.3, 33.0, 28.7, 27.5, 12.9.

HRMS (ESI): Calcd for $C_{28}H_{31}N_2O_3$ $[M+H]^+$: 443.2329, found: 443.2328.

25:

M.p.: 190-192 °C.

R*f* = 0.38 (petroleum ether : ethyl acetate = 1:1).

$[\alpha]_D^{20}$ = –53.5 (*c* 0.39, $CHCl_3$).

FTIR (KBr, thin film) cm^{-1} : 3430, 2922, 2938, 2889, 2839, 1727, 1610, 1509, 1467,

1434, 1347, 1301, 1243, 1179, 1098, 1031, 838, 812, 748.

^{1}H NMR (400 MHz, $CDCl_3$): δ 7.51 (d, *J* = 7.6 Hz, 1H), 7.24 (d, *J* = 7.6 Hz, 1H), 7.15 (t, *J* = 7.2 Hz, 1H), 7.10 (t, *J* = 7.2 Hz, 1H), 6.95 (d, *J* = 8.4 Hz, 2H), 6.79 (d, *J* = 8.4 Hz, 2H), 5.31-5.26 (m, 1H), 5.22 (d, *J* = 16.8 Hz, 1H), 5.14 (d, *J* = 16.8 Hz, 1H), 4.17 (d, *J* = 9.2 Hz, 1H), 3.80 (d, *J* = 17.2 Hz, 1H), 3.75 (s, 3H), 3.75-3.71 (m, 1H), 3.68 (s, 3H), 3.54 (d, *J* = 17.6 Hz, 1H), 3.26 (dd, *J* = 15.6, 4.8 Hz, 1H), 2.74 (d, *J* = 15.6 Hz, 1H), 2.66 (br s, 1H), 2.51 (d, *J* = 7.6 Hz, 1H), 1.95-1.89 (m, 1H), 1.57-1.54 (m, 1H), 1.53 (d, *J* = 6.4 Hz, 3H).

^{13}C NMR (100 MHz, $CDCl_3$): δ 174.1, 159.0, 139.1, 137.3, 134.7, 129.6, 127.6, 127.5, 121.5, 119.4, 118.4, 117.7, 114.3, 109.6, 103.8, 55.4, 53.7, 53.4, 52.0, 48.9, 47.6, 46.3, 35.5, 34.1, 27.4, 12.7.

HRMS (ESI): Calcd for $C_{28}H_{31}N_2O_3$ $[M+H]^+$: 443.2329, found: 443.2328.

1.2.15 Synthesis of Alcohols 26 and 27

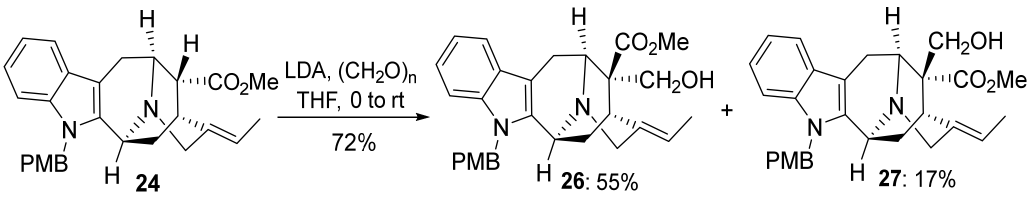

To a solution of ester **24** (442.6 mg, 1.0 mmol) in anhydrous THF (20 mL) was added LDA (1.0 M, 6.0 mL, 6.0 mmol) via syringe at 0 ºC under Ar. After being stirred at 0 ºC for 10 min, a powder of $(HCHO)_n$ (87.0 mg, 3.0 mmol) was added in one portion, and the resulting mixture was stirred at 0 ºC for 50 min. The reaction was quenched with saturated *aq.* NH_4Cl (5 mL). The mixture was diluted with water (20 mL) and extracted with EtOAc (3 × 15 mL). The combined organic phases were washed with brine (15 mL), dried over Na_2SO_4, filtered, and concentrated. The residue was purified by column chromatography on silica gel to yield alcohol **27** (80.6 mg, 17%, petroleum ether/EtOAc = 1:3) as a white solid. Futher elution provided alcohol **26** (262.1 mg, 55%, EtOAc) as white solid.

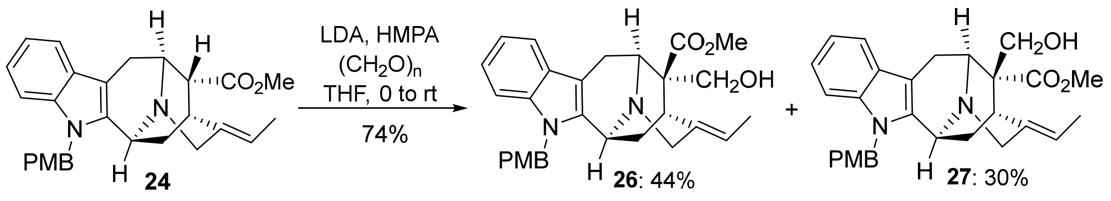

To a solution of ester **24** (200.0 mg, 0.452 mmol) and HMPA (0.18 mL) in anhydrous THF (15 mL) was added LDA (1.0 M, 2.7 mL, 2.7 mmol) via syringe at 0 ºC under Ar. After being stirred at 0 ºC for 10 min, a powder of $(HCHO)_n$ (40.6 mg, 1.36 mmol) was added in one portion, and the resulting mixture was stirred at 0 ºC for 50 min. The reaction was quenched with saturated *aq.* NH_4Cl (3 mL). The mixture was diluted with water (10 mL) and extracted with EtOAc (3 × 10 mL). The combined organic phases were washed with brine (15 mL), dried over Na_2SO_4, filtered, and concentrated. The residue was purified by column chromatography on silica gel to yield alcohol **27** (63.3 mg, 30%, petroleum ether/EtOAc = 1:3) as a white solid. Futher elution provided alcohol **26** (93.2 mg, 44%, EtOAc) as white solid.

26:

M.p.: 120-122 ºC.

R*f* = 0.21 (petroleum ether : ethyl acetate = 1:3).

$[\alpha]_D^{20}$ = –21.5 (*c* 0.66, $CHCl_3$).

FTIR (KBr, thin film) cm^{-1} : 3435, 2906, 1716, 1612, 1513, 1465, 1439, 1297, 1241, 1215, 1177, 1100, 1031, 841, 818, 743.

^{1}H NMR (400 MHz, $CDCl_3$): δ 7.47 (d, *J* = 6.8 Hz, 1H), 7.15 (d, *J* = 7.2 Hz, 1H), 7.08 (t, *J* = 6.8 Hz, 1H), 7.07 (t, *J* = 6.8 Hz, 1H), 6.94 (d, *J* = 8.4 Hz, 2H), 6.79 (d, *J* = 8.4 Hz, 2H), 5.38 (q, *J* = 6.8 Hz, 1H), 5.25 (d, *J* = 16.8 Hz, 1H), 5.08 (d, *J* = 16.8 Hz, 1H), 4.17 (d, *J* = 9.6 Hz, 1H), 3.83 (d, *J* = 10.4 Hz, 1H), 3.75 (s, 3H), 3.67 (d, *J* = 10.4 Hz, 1H), 3.58 (br s, 2H), 3.35 (d, *J* = 15.2 Hz, 1H), 3.95-3.04 (m, 1H), 3.04 (s, 3H), 2.96 (dd, *J* = 15.2, 4.0 Hz, 1H), 2.58 (d, *J* = 11.6 Hz, 1H), 2.15 (br s, 1H), 1.79 (t, *J* = 11.6 Hz, 1H), 1.63 (d, *J* = 6.4 Hz, 3H).

^{13}C NMR (100 MHz, $CDCl_3$): δ 174.0, 159.0, 138.6, 137.5, 129.7, 127.4, 127.0, 121.3, 119.3, 118.2, 116.7, 114.3, 109.6, 105.6, 69.0, 57.9, 55.9, 55.4, 51.6, 49.8, 46.4,

29.3, 28.9, 24.9, 13.1.

HRMS (ESI): Calcd for $C_{29}H_{33}N_2O_4$ $[M+H]^+$: 473.2435, found: 473.2438.

27:

M.p.: 147-148 °C.

R*f* = 0.43 (petroleum ether : ethyl acetate = 1:3).

$[\alpha]_D^{20}$ = –52.4 (*c* 1.05, $CHCl_3$).

FTIR (KBr, thin film) cm^{-1} : 3439, 2955, 1793, 1736, 1631, 1455, 1348, 1212, 1236, 1174, 1154, 745.

^{1}H NMR (400 MHz, $CDCl_3$): δ 7.51 (d, *J* = 7.2 Hz, 1H), 7.23 (d, *J* = 8.0 Hz, 1H), 7.15 (t, *J* = 7.2 Hz, 1H), 7.10 (t, *J* = 7.2 Hz, 1H), 6.95 (d, *J* = 8.4 Hz, 2H), 6.79 (d, *J* = 8.4 Hz, 2H), 5.38 (q, *J* = 6.8 Hz, 1H), 5.21 (d, *J* = 16.8 Hz, 1H), 5.11 (d, *J* = 16.8 Hz, 1H), 4.28 (d, *J* = 6.0 Hz, 1H), 4.04 (dd, *J* = 10.0, 3.2 Hz, 1H), 3.75 (s, 3H), 3.72 (s, 3H), 3.71 (d, *J* = 11.2 Hz, 1H), 3.64 (d, *J* = 17.6 Hz, 1H), 3.60 (d, *J* = 11.2 Hz, 1H), 3.54 (d, *J* = 16.8 Hz, 1H), 3.17-3.12 (m, 2H), 2.99 (d, *J* = 16.4 Hz, 1H), 1.79-1.72 (m, 1H), 1.64 (dt, *J* = 13.6, 3.2 Hz, 1H), 1.57 (d, *J* = 6.8 Hz, 3H).

^{13}C NMR (100 MHz, $CDCl_3$): δ 176.4, 159.1, 138.4, 137.3, 136.3, 129.7, 127.5, 126.5, 121.6, 119.3, 118.6, 116.2, 114.3, 109.5, 105.8, 63.4, 56.0, 55.4, 53.7, 53.5, 52.3, 48.1, 46.3, 30.6, 28.6, 22.5, 12.9.

HRMS (ESI): Calcd for $C_{29}H_{33}N_2O_4$ $[M+H]^+$: 473.2435, found: 473.2434.

1.2.16 Synthesis of Alcohols 28 and 29

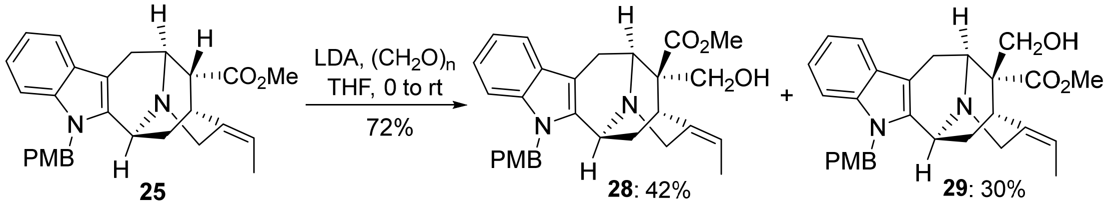

To a solution of ester **25** (170.1 mg, 0.384 mmol) in anhydrous THF (10 mL) was added LDA (1.0 M, 2.3 mL, 2.3 mmol) via syringe at 0 °C under Ar. After being stirred at 0 °C for 10 min, a powder of $(HCHO)_n$ (33.0 mg, 1.15 mmol) was added in

one portion, and the resulting mixture was stirred at 0 ºC for 50 min. The reaction was quenched with saturated *aq.* NH_4Cl (3 mL). The mixture was diluted with water (10 mL) and extracted with EtOAc (3 × 10 mL). The combined organic phases were washed with brine (15 mL), dried over Na_2SO_4, filtered, and concentrated. The residue was purified by column chromatography on silica gel to yield alcohol **29** (55.1 mg, 30%, petroleum ether/EtOAc = 1:3) as a white solid. Futher elution provided alcohol **28** (77.3 mg, 42%, EtOAc) as white solid.

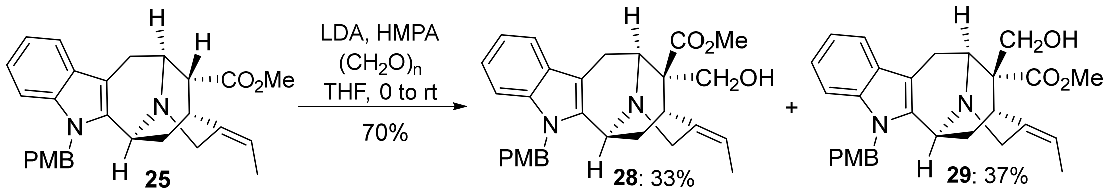

To a solution of ester **25** (200.0 mg, 0.452 mmol) and HMPA (0.18 mL) in anhydrous THF (15 mL) was added LDA (1.0 M, 2.7 mL, 2.7 mmol) via syringe at 0 ºC under Ar. After being stirred at 0 ºC for 10 min, a powder of $(HCHO)_n$ (40.6 mg, 1.36 mmol) was added in one portion, and the resulting mixture was stirred at 0 ºC for 50 min. The reaction was quenched with saturated *aq.* NH_4Cl (3 mL). The mixture was diluted with water (10 mL) and extracted with EtOAc (3 × 10 mL). The combined organic phases were washed with brine (15 mL), dried over Na_2SO_4, filtered, and concentrated. The residue was purified by column chromatography on silica gel to yield alcohol **29** (78.9 mg, 37%, petroleum ether/EtOAc = 1:3) as a white solid. Futher elution provided alcohol **28** (71.6 mg, 33%, EtOAc) as white solid.

28:

M.p.: 115-118 ºC.

R*f* = 0.25 (petroleum ether : ethyl acetate = 1:3).

$[\alpha]_D^{20} = -18.1$ (*c* 0.31, $CHCl_3$).

FTIR (KBr, thin film) cm^{-1} : 3435, 2904, 2838, 1716, 1613, 1513, 1465, 1437, 1305, 1240, 1219, 1096, 1030, 843, 743.

^{1}H NMR (400 MHz, $CDCl_3$): δ 7.46 (dd, *J* = 6.8, 1.2 Hz, 1H), 7.15 (d, *J* = 7.2 Hz, 1H), 7.08 (td, *J* = 6.8, 1.2 Hz, 1H), 7.05 (td, *J* = 6.8, 1.2 Hz, 1H), 6.93 (d, *J* = 8.4 Hz,

2H), 6.78 (d, *J* = 8.4 Hz, 2H), 5.35-5.30 (m, 1H), 5.24 (d, *J* = 16.8 Hz, 1H), 5.07 (d, *J* = 16.8 Hz, 1H), 4.14 (d, *J* = 8.8 Hz, 1H), 3.83 (d, *J* = 10.4 Hz, 1H), 3.75 (s, 3H), 3.68 (d, *J* = 10.4 Hz, 1H), 3.66 (d, *J* = 17.2 Hz, 1H), 3.56 (d, *J* = 17.2 Hz, 1H), 3.36 (d, *J* = 15.6 Hz, 1H), 3.06 (s, 3H), 3.06-3.04 (m, 1H), 2.98 (dd, *J* = 15.6, 4.8 Hz, 1H), 2.59-2.54 (m, 2H), 2.11 (br s, 1H), 1.80 (t, *J* = 11.2 Hz, 1H), 1.57 (d, *J* = 6.8 Hz, 3H).

^{13}C NMR (100 MHz, $CDCl_3$): δ 174.0, 159.0, 138.7, 138.4, 137.5, 129.7, 127.4, 127.0, 121.3, 119.3, 118.2, 117.0, 114.3, 109.6, 105.3, 69.3, 58.2, 55.4, 53.8, 51.5, 51.4, 49.6, 46.4, 36.3, 30.1, 24.8, 12.6.

HRMS (ESI): Calcd for $C_{29}H_{33}N_2O_4$ $[M+H]^+$: 473.2435, found: 473.2434.

29:

M.p.: 100-102 °C.

R*f* = 0.47 (petroleum ether : ethyl acetate = 1:3).

$[\alpha]_D^{20}$ = –60.5 (*c* 0.36, $CHCl_3$).

FTIR (KBr, thin film) cm^{-1} : 3436, 2949, 1735, 1614, 1513, 1464, 1247, 1210, 1184, 1112, 1034, 821, 741.

^{1}H NMR (400 MHz, $CDCl_3$): δ 7.50 (d, *J* = 7.6 Hz, 1H), 7.23 (d, *J* = 8.0 Hz, 1H), 7.14 (td, *J* = 7.2, 1.2 Hz, 1H), 7.10 (td, *J* = 7.2, 1.2 Hz, 1H), 6.95 (d, *J* = 8.8 Hz, 2H), 6.78 (d, *J* = 8.8 Hz, 2H), 5.26-5.21 (m, 1H), 5.21 (d, *J* = 16.8 Hz, 1H), 5.10 (d, *J* = 16.8 Hz, 1H), 4.28 (d, *J* = 6.0 Hz, 1H), 4.01 (dd, *J* = 10.4, 3.6 Hz, 1H), 3.74 (s, 3H), 3.71 (s, 3H), 3.71 (d, *J* = 11.2 Hz, 2H), 3.55-3.50 (m, 2H), 3.13 (dd, *J* = 16.4, 6.4 Hz, 1H), 2.98 (d, *J* = 16.4 Hz, 1H), 2.62 (t, *J* = 2.4 Hz, 1H), 2.20 (br s, 1H), 1.82-1.75 (m, 1H), 1.61 (dt, *J* = 13.6, 3.2 Hz, 1H), 1.51 (d, *J* = 6.8 Hz, 3H).

^{13}C NMR (100 MHz, $CDCl_3$): δ 176.6, 159.0, 138.6, 137.6, 137.2, 129.7, 127.4, 126.5, 121.5, 119.3, 118.5, 116.7, 114.3, 109.5, 105.6, 63.2, 55.4, 54.3, 53.8, 53.7, 52.4, 48.0, 46.2, 38.0, 29.2, 22.4, 12.6.

HRMS (ESI): Calcd for $C_{29}H_{33}N_2O_4$ $[M+H]^+$: 473.2435, found: 473.2436.

1.2.17 Synthesis of Olefin 24 from Alcohol 26 or 27

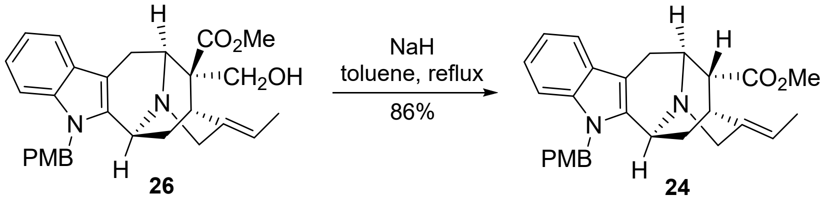

To a solution of compound **26** (67.0 mg, 0.14 mmol) in dry toluene (10 mL) was added NaH (90% wt, 37.2 mg, 1.40 mmol) at 0 ºC under Ar. After being stirred at reflux for 10 h, the reaction was quenched with saturated *aq.* NH_4Cl (3 mL) at 0 ºC and diluted with water (10 mL). The mixture was extracted with EtOAc (3 × 5 mL). The combined organic phases were washed with brine (10 mL), dried over Na_2SO_4, filtered, and concentrated. The residue was purified by flash column chromatography on silica gel (petroleum ether/EtOAc = 1:1) to give olefin **24** (53.5 mg, 86%).

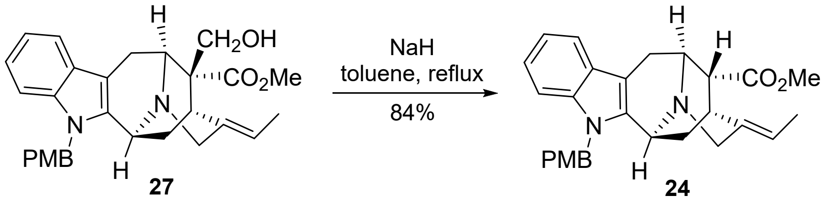

To a solution of compound **27** (66.0 mg, 0.14 mmol) in dry toluene (10 mL) was added NaH (90% wt, 111.6 mg, 4.20 mmol) at 0 ºC under Ar. After being stirred at reflux for 3 h, the reaction was quenched with saturated *aq.* NH_4Cl (3 mL) at 0 ºC and diluted with water (10 mL). The mixture was extracted with EtOAc (3 × 5 mL). The combined organic phases were washed with brine (10 mL), dried over Na_2SO_4, filtered, and concentrated. The residue was purified by flash column chromatography on silica gel (petroleum ether/EtOAc = 1:1) to give olefin **24** (52.3 mg, 84%).

1.2.18 Synthesis of Olefin 25 from Alcohol 28 or 29

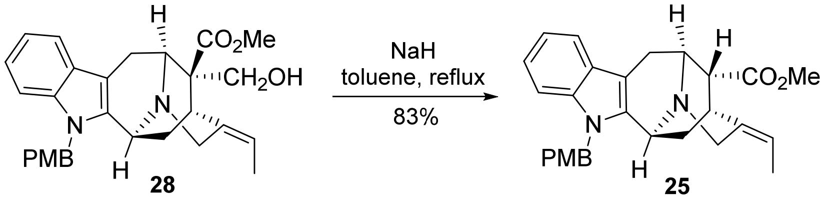

To a solution of compound **28** (360.0 mg, 0.76 mmol) in dry toluene (30 mL) was added NaH (90% wt, 204.0 mg, 7.60 mmol) at 0 °C under Ar. After being stirred at reflux for 10 h, the reaction was quenched with saturated *aq.* NH_4Cl (10 mL) at 0 °C and diluted with water (15 mL). The mixture was extracted with EtOAc (3 × 20 mL). The combined organic phases were washed with brine (30 mL), dried over Na_2SO_4, filtered, and concentrated. The residue was purified by column chromatography on silica gel (petroleum ether/EtOAc = 1:1) to afford olefin **25** (278.4 mg, 83%).

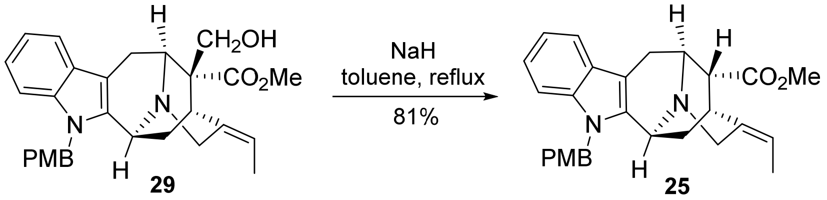

To a solution of compound **29** (56.0 mg, 0.118 mmol) in dry toluene (5 mL) was added NaH (90% wt, 31.6 mg, 3.54 mmol) at 0 °C under Ar. After being stirred at reflux for 3 h, the reaction was quenched with saturated *aq.* NH_4Cl (5 mL) at 0 °C and diluted with water (15 mL). The mixture was extracted with EtOAc (3 × 10 mL). The combined organic phases were washed with brine (20 mL), dried over Na_2SO_4, filtered, and concentrated. The residue was purified by column chromatography on silica gel (petroleum ether/EtOAc = 1:1) to afford olefin **25** (42.3 mg, 81%).

1.2.19 Synthesis of Akuammidine (1)

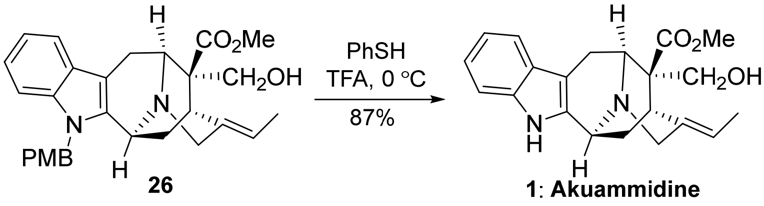

To compound **26** (220.0 mg, 0.466 mmol) in PhSH (2.4 mL) was added TFA (22 mL)

at 0 °C. After being stirred at 0 °C for 3 d, the resulting mixture was poured into ice-water (100 mL) containing sodium bicarbonate (25.2 g, > 0.3 mol). The mixture was then extracted with EtOAc (3 × 25 mL). The combined organic phases were washed with brine (30 mL), dried over Na_2SO_4, filtered, and concentrated. The residue was purified by flash column chromatography on silica gel (CH_2Cl_2/MeOH = 20:1) to yield Akuammidine (**1**, 143.3 mg, 87%) as a yellowish solid.

M.p.: 223-225 °C.

$\mathbf{R}_f$ = 0.15 (ethyl acetate).

$[\alpha]_D^{20}$ = 21.6 (*c* 1.14, MeOH). Lit[3]: $[\alpha]_D^{15}$ = 24 (MeOH).

FTIR (KBr, thin film) cm^{-1} : 3405, 2943, 2855, 1716, 1625, 1455, 1439, 1343, 1298, 1223, 1100, 1033, 1001, 846, 734.

^{1}H NMR (400 MHz, MeOD): δ 7.37 (d, *J* = 7.6 Hz, 1H), 7.27 (d, *J* = 8.0 Hz, 1H), 7.04 (td, *J* = 7.6, 0.8 Hz, 1H), 6.96 (td, *J* = 7.6, 0.8 Hz, 1H), 5.44 (q, *J* = 6.8 Hz, 1H), 4.22 (d, *J* = 9.6 Hz, 1H), 3.77 (d, *J* = 9.6 Hz, 1H), 3.65 (d, *J* = 9.6 Hz, 1H), 3.63 (dt, *J* = 16.8, 2.4 Hz, 1H), 3.51 (d, *J* = 16.8 Hz, 1H), 3.41 (dd, *J* = 18.4, 4.4 Hz, 1H), 3.25 (d, *J* = 3.2 Hz, 1H), 2.94 (s, 3H), 2.83 (dd, *J* = 18.4, 4.4 Hz, 1H), 2.81 (s, 1H), 2.70 (ddd, *J* = 12.8, 4.4, 2.0 Hz, 1H), 1.89 (ddd, *J* = 12.8, 10.4, 2.0 Hz, 1H), 1.69 (dt, *J* = 6.8, 2.0 Hz, 3H).

^{13}C NMR (100 MHz, MeOD): δ 173.3, 137.08, 137.05, 136.4, 126.5, 120.7, 118.3, 117.2 116.8, 110.5, 104.7, 67.5, 57.6, 54.7, 51.2, 50.5, 50.2, 28.9, 28.7, 23.6, 11.9.

HRMS (ESI): Calcd for $C_{21}H_{25}N_2O_3$ $[M+H]^+$: 353.1860, found: 353.1856.

1.2.20 Synthesis of 19-(*Z*)-Akuammidine (4)

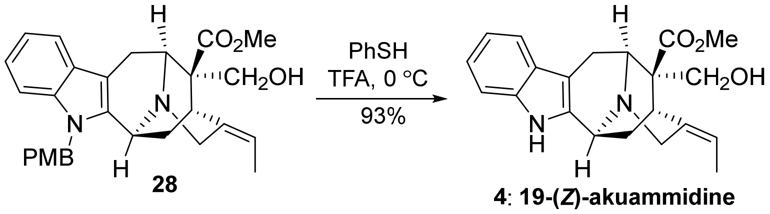

To compound **28** (87.0 mg, 0.184 mmol) in PhSH (0.9 mL), TFA (9 mL) was added at 0 °C. After being stirred at 0 °C for 3 d, the resulting mixture was poured into

ice-water (100 mL) containing sodium bicarbonate (10.3 g, > 0.12 mol) and extracted with EtOAc (3 × 10 mL). The combined organic phases were washed with brine (10 mL), dried over Na_2SO_4, filtered, and concentrated. The residue was purified by flash column chromatography on silica gel (CH_2Cl_2/MeOH = 20:1) to afford 19-(*Z*)-Akuammidine (**4**, 60.2 mg, 93%) as a yellowish solid.

M.p.: 238-239 °C.

R*f* = 0.19 (ethyl acetate).

$[\alpha]_D^{20}$ = 8.8 (*c* 0.63, MeOH). Lit[4]: $[\alpha]_D^{16}$ = 9 (*c* 0.16, MeOH).

FTIR (KBr, thin film) cm^{-1} : 3404, 2939, 2900, 1717, 1457, 1434, 1310, 1222, 1099, 1083, 1063, 1035, 840, 738.

^{1}H NMR (400 MHz, MeOD): δ 7.37 (d, *J* = 7.6 Hz, 1H), 7.27 (d, *J* = 8.0 Hz, 1H), 7.04 (td, *J* = 7.2, 0.8 Hz, 1H), 6.97 (td, *J* = 7.2, 0.8 Hz, 1H), 5.47-5.43 (m, 1H), 4.20 (d, *J* = 10.0 Hz, 1H), 3.74 (d, *J* = 10.4 Hz, 1H), 3.70 (d, *J* = 10.4 Hz, 1H), 3.64 (d, *J* = 17.2 Hz, 1H), 3.58 (d, *J* = 17.2 Hz, 1H), 3.42 (dd, *J* = 15.6, 1.2 Hz, 1H), 2.97 (s, 3H), 2.85 (dd, *J* = 15.6, 4.4 Hz, 1H), 2.78 (d, *J* = 4.0 Hz, 1H), 2.76 (d, *J* = 2.4 Hz, 1H), 2.68 (dd, *J* = 13.2, 1.6 Hz, 1H), 1.93 (ddd, *J* = 12.0, 10.0, 1.2 Hz, 1H), 1.62 (d, *J* = 6.8 Hz, 3H).

^{13}C NMR (100 MHz, MeOD): δ 174.9, 139.1, 138.9, 138.7, 128.2, 122.3, 119.9, 118.8, 118.6, 112.2, 106.0, 69.1, 59.5, 54.1, 53.2, 51.9, 51.8, 37.2, 31.4, 25.2, 12.8.

HRMS (ESI): Calcd for $C_{21}H_{25}N_2O_3$ $[M+H]^+$: 353.1860, found: 353.1859.

1.2.21 Synthesis of Polyneuridine (2)

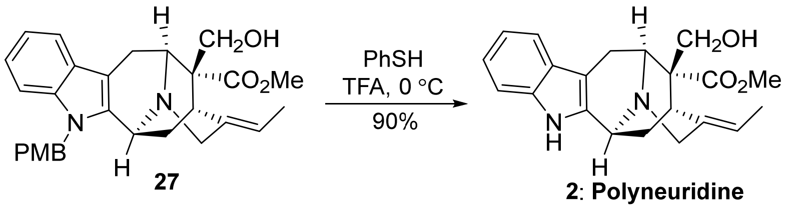

To compound **27** (90.0 mg, 0.19 mmol) in PhSH (1 mL), TFA (10 mL) was added at 0 °C. After being stirred at 0 °C for 3 d, the resulting mixture was poured into ice-water (100 mL) containing sodium bicarbonate (11.5 g, > 0.13 mol) and extracted with

EtOAc (3 × 10 mL). The combined organic phases were washed with brine (10 mL), dried over Na_2SO_4, filtered, and concentrated. The residue was purified by flash column chromatography on silica gel (CH_2Cl_2/MeOH = 20:1) to afford Polyneuridine (**2**, 60.5 mg, 90%) as a white solid.

M.p.: 230-231 °C.

R*f* = 0.22 (ethyl acetate).

$[\alpha]_D^{20}$ = 1.8 (*c* 0.15, $CHCl_3$). Lit[5]: $[\alpha]_D$ = 1 ($CHCl_3$).

FTIR (KBr, thin film) cm^{-1} : 3425, 2949, 1731, 1630, 1452, 1230, 1214, 1177, 1084, 1048, 749.

^{1}H NMR (400 MHz, $CDCl_3$): δ 7.86 (br s, 1H), 7.48 (d, *J* = 7.6 Hz, 1H), 7.30 (d, *J* = 8.0 Hz, 1H), 7.15 (td, *J* = 7.2, 0.8 Hz, 1H), 7.10 (t, *J* = 7.2, 0.8 Hz, 1H), 5.27 (br q, *J* = 6.8 Hz, 1H), 4.27 (d, *J* = 6.4 Hz, 1H), 4.03 (dd, *J* = 10.0, 4.0 Hz, 1H), 3.72 (s, 3H), 3.70 (d, *J* = 11.6 Hz, 1H), 3.60 (d, *J* = 11.2 Hz, 1H), 3.64-3.55 (m, 2H), 3.19 (dd, *J* = 3.5, 2.4 Hz, 1H), 3.09 (dd, *J* = 16.4, 6.4 Hz, 1H), 2.94 (br d, *J* = 16.4 Hz, 1H), 1.89 (ddd, *J* = 13.2, 9.6, 2.4 Hz, 1H), 1.84 (ddd, *J* = 13.2, 3.6, 3.2 Hz, 1H), 1.60 (d, *J* = 6.8 Hz, 3H).

^{13}C NMR (100 MHz, $CDCl_3$): δ 176.4, 136.9, 136.5, 136.2, 126.5, 121.6, 119.5, 118.3, 116.0, 110.9, 106.2, 63.2, 55.7, 53.6, 53.4, 52.2, 49.0, 30.6, 28.9, 22.3, 12.7.

HRMS (ESI): Calcd for $C_{21}H_{25}N_2O_3$ $[M+H]^+$: 353.1860, found: 353.1867.

1.2.22 Synthesis of the Mixture of 30a and 30b

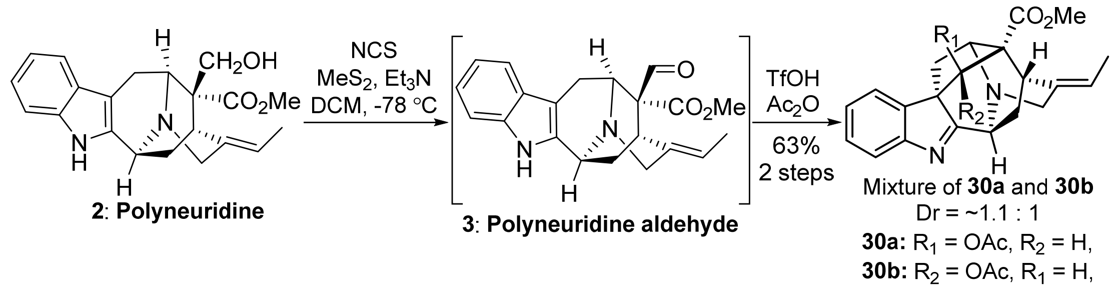

Dimethyl sulfide (154 μL, 2.13 mmol) was added to a solution of *N*-chlorosuccinimide (57.0 mg, 0.43 mmol) in dry CH_2Cl_2 (5 mL) at 0 °C under Ar. White precipitate appeared immediately after addition of the dimethyl sulfide. The

resulting mixture was then stirred at –78 ºC for 30 min. A solution of compound **2** (30.0 mg, 0.0851 mmol) in dry CH_2Cl_2 (10 mL) was then introduced, and the resulting mixture was then stirred at –78 ºC for another 30 min. A solution of triethylamine (118 μL, 2.41 mmol) in CH_2Cl_2 (2 mL) was added to the mixture. After being stirred at 0 ºC for an additional 40 min, the reaction was quenched with saturated *aq*. NH_4Cl (2 mL). The mixture was diluted with water (15 mL) and extracted with CH_2Cl_2 (3 × 10 mL). The combined organic phases were washed with brine (15 mL), dried over Na_2SO_4, filtered, and concentrated under reduced pressure (water bath temperature must be below 25 ºC). The residue was purified by flash column chromatography on silica gel (CH_2Cl_2/MeOH = 20:1) to afford Polyneuridine aldehyde which was confirmed by HRMS (Calcd for $C_{21}H_{23}N_2O_3$ $[M+H]^+$: 351.1703, found: 351.1707) and used directly in the next step. To the aldehyde in Ac_2O (0.3 mL) was added cold CF_3SO_3H (85 μL, 0.85 mmol) at room temperature under Ar. After being stirred at room temperature for 10 min, the mixture was poured into a cold saturated aqueous solution of sodium bicarbonate (15 mL) and extracted with EtOAc (3 × 10 mL). The combined organic phases were washed with brine (15 mL), dried over Na_2SO_4, filtered, and concentrated. The residue was purified by preparative TLC on silica gel (hexane/EtOAc = 1:3) to afford an inseparable mixture of **30a** and **30b** (21.0 mg, 63% from **2**, dr = ~ 1.1:1).

^{1}H NMR (400 MHz, $CDCl_3$): δ 7.61 (d, *J* = 7.6 Hz, 0.9H), 7.57 (d, *J* = 7.6 Hz, 1H), 7.39-7.34 (m, 2.9H), 7.25-7.18 (m, 2.8H), 5.96 (s, 1H), 5.60 (d, *J* = 1.2 Hz, 0.9H), 5.37-5.29 (m, 1.9H), 4.32 (d, *J* = 9.6 Hz, 1H), 4.21 (d, *J* = 8.8 Hz, 0.9H), 3.85 (d, *J* = 8.8 Hz, 0.9H), 3.75 (s , 3H), 3.71 (s , 2.7H), 3.62 (d, *J* = 4.4 Hz, 1H), 3.61 (d, *J* = 4.4 Hz, 1H), 3.57-3.55 (m, 1.9H), 3.53-3.48 (m, 1.9H), 3.33 (d, *J* = 5.2 Hz, 1H), 2.82 (dd, *J* = 12.0, 4.8 Hz, 0.9H), 2.60 (dd, *J* = 12.4, 4.8 Hz, 1H), 2.39 (dd, *J* = 14.0, 5.2 Hz, 1H), 2.12 (s, 2.7H), 2.08-1.94 (m 3.7H), 1.83 (s, 3H), 1.71 (d, *J* = 11.2 Hz, 1H), 1.68 (d, *J* = 12.4 Hz, 1H), 1.62 (d, *J* = 6.8 Hz, 3H), 1.57 (d, *J* = 6.8 Hz, 2.7H).

^{13}C NMR (100 MHz, $CDCl_3$): δ 183.5 (182.5), 174.4 (171.8), 169.6 (168.6), 156.6, 135.2 (135.6), 134.87 (134.94), 128.9 (129.1), 125.7 (125.9), 122.6 (123.2), 120.9 (121.4), 117.4 (117.9), 78.5 (75.9), 64.3 (65.0), 61.4 (60.6), 61.1, 56.1 (55.6), 54.2

(53.9), 53.4, 52.5 (52.1), 36.4 (37.5), 30.2 (31.9), 28.1 (26.6), 20.8 (20.9), 13.2 (12.8).

1.2.23 Synthesis of the Mixture of 31a and 31b

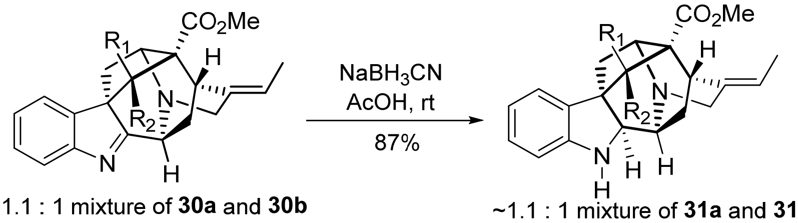

~1.1 : 1 mixture of **30a** and **30b**
30a: R_1 = OAc, R_2 = H
30b: R_2 = OAc, R_1 = H

~1.1 : 1 mixture of **31a** and **31b**
31a: R_1 = OAc, R_2 = H
31b: R_2 = OAc, R_1 = H

To a mixture of **30a** and **30b** (39.0 mg, 0.099 mmol) in AcOH (1.1 mL) was added $NaBH_3CN$ (10.0 mg, 0.15 mmol) in one portion at room temperature. After being stirred at room temperature for 1 h, the mixture was poured into a cold saturated aqueous solution of sodium bicarbonate (20 mL), and extracted with EtOAc (3 × 10 mL). The combined organic phases were washed with brine (15 mL) and dried over anhydrous Na_2SO_4. After removal of the solvents under reduced pressure, the residue was purified by flash column chromatography on silica gel (CH_2Cl_2/MeOH = 20:1) to afford an inseparable mixture of **31a** and **31b** (34.3 mg, ~ 1.1:1, 87%).

^{1}H NMR (400 MHz, $CDCl_3$): δ 7.10-7.06 (m, 2.8H), 6.99 (d, J = 6.8 Hz, 1H), 6.78 (d, J = 7.6 Hz, 0.9H), 6.76-6.71 (m, 2.9H), 5.70 (d, J = 1.2 Hz, 1H), 5.30-5.21 (m, 2.8H), 4.08 (d, J = 4.8 Hz, 0.9H), 3.85 (d, J = 5.2 Hz, 2H), 3.66 (s, 2.7H), 3.65 (s, 3H), 3.61 (d, J = 4.4 Hz, 1.9H), 3.53 (dd, J = 9.6, 5.2 Hz, 0.9H), 3.50-3.47 (m, 3.8H), 3.46-3.42 (m, 2H), 3.24 (d, J = 4.8 Hz, 0.9H), 3.09 (dd, J = 13.6, 4.8 Hz, 0.9H), 2.75 (dd, J = 14.4, 4.8 Hz, 1H), 2.56 (dd, J = 11.6, 4.8 Hz, 1H), 2.46 (dd, J = 12.0, 4.8 Hz, 0.9H), 1.87 (d, J = 11.6 Hz, 0.9H), 1.86 (s, 3H), 1.82 (s, 2.7H), 1.81 (d, J = 12.4 Hz, 1H), 1.60 (d, J = 6.8 Hz, 2.7H), 1.55 (d, J = 6.8 Hz, 3H), 1.53-1.51 (m, 1.9H).

^{13}C NMR (100 MHz, $CDCl_3$): δ 172.4 (174.7), 168.6 (169.7), 151.6 (152.2), 136.6 (136.5), 128.64 (128.58), 128.5 (128.4), 124.0 (123.1), 119.5 (120.1), 117.0 (116.5), 111.23 (111.16), 75.2 (83.5), 68.6 (69.5), 61.6 (62.2), 57.0 (59.0), 55.4 (55.6), 54.7 (55.3), 54.2 (52.7), 51.8 (52.2), 36.6 (37.0), 30.7 (29.8), 22.3 (23.5), 20.84 (20.81), 12.9 (13.2).

1.2.24 Synthesis of Ester 31a and Vincarine (8)

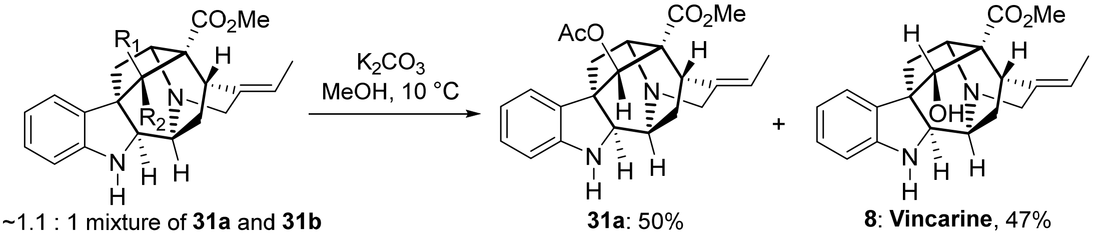

~1.1 : 1 mixture of **31a** and **31b**
31a: R_1 = OAc, R_2 = H
31b: R_2 = OAc, R_1 = H

31a: 50%

8: **Vincarine**, 47%

To a solution of **31a** and **31b** (18.0 mg, 0.046 mmol) in MeOH (2 mL) was added K_2CO_3 (19.0 mg, 0.137 mmol) at 10 ºC. After being stirred at 10 ºC for 4 h, the reaction was quenched with saturated *aq.* NH_4Cl (1 mL). The mixture was diluted with water (15 mL) and extracted with CH_2Cl_2 (3 × 8 mL). The combined organic phases were washed with brine (10 mL), dried over Na_2SO_4, filtered, and concentrated. The residue was purified by flash column chromatography on silica gel (CH_2Cl_2/MeOH = 25:1) to give compound **31a** (8.9 mg, 50%) as a white solid. Futher elution provides Vincarine (**8**, 7.8 mg, 47%) as white plate.

31a:

M.p.: 168-170 ºC.

R*f* = 0.43 (CH_2Cl_2 : MeOH = 25:1).

$[\alpha]_D^{20}$ = 1.5 (*c* 0.57, $CHCl_3$).

FTIR (KBr, thin film) cm^{-1} : 3436, 2948, 2853, 1736, 1612, 1480, 1372, 1308, 1285, 1243, 1134, 1097, 1056, 823, 758, 742.

^{1}H NMR (400 MHz, $CDCl_3$): δ 7.08 (td, *J* = 7.6, 0.8 Hz, 1H), 7.00 (d, *J* = 7.6 Hz, 1H), 6.75 (d, *J* = 8.0 Hz, 1H), 6.73 (t, *J* = 7.6 Hz, 1H), 5.70 (d, *J* = 1.2 Hz, 1H), 5.26 (q, *J* = 6.8 Hz, 1H), 3.85 (br s, 1H), 3.84 (br s, 1H), 3.65 (s, 3H), 3.60 (d, *J* = 4.8 Hz, 1H), 3.53-3.49 (m, 2H), 3.45-3.38 (m, 2H), 2.74 (dd, *J* = 14.0, 4.8 Hz, 1H), 2.56 (dd, *J* = 12.0, 4.8 Hz, 1H), 1.86 (s, 3H), 1.80 (d, *J* = 11.6 Hz, 1H), 1.55 (d, *J* = 7.6 Hz, 3H), 1.52 (d, *J* = 10.0 Hz, 1H).

^{13}C NMR (100 MHz, $CDCl_3$): δ 172.4, 168.6, 151.7, 136.8, 128.63, 128.56, 124.0, 119.5, 116.9, 111.2, 75.2, 68.7, 61.6, 59.0, 57.0, 55.5, 54.8, 51.8, 36.7, 30.7, 22.3, 20.9, 12.9.

HRMS (ESI): Calcd for $C_{23}H_{27}N_2O_4$ $[M+H]^+$: 395.1965, found: 395.1961.

Vincarine (**8**):

M.p.: 136-138 °C.

R*f* = 0.32 (CH_2Cl_2 : MeOH = 25:1).

$[\alpha]_D^{20}$ = 33.4 (*c* 1.03, MeOH). Lit[6]: $[\alpha]_D$ = 13.98 (*c* 0.785, MeOH).

FTIR (KBr, thin film) cm^{-1} : 3435, 2949, 1725, 1612, 1480, 1463, 1310, 1241, 1115, 1058, 824, 749.

^{1}H NMR (400 MHz, $CDCl_3$): δ 7.12 (td, *J* = 7.6, 0.8 Hz, 1H), 7.06 (d, *J* = 6.8 Hz, 1H), 6.84 (t, *J* = 7.6 Hz, 1H), 6.82 (d, *J* = 7.6 Hz, 1H), 5.23 (q, *J* = 6.8 Hz, 1H), 4.00 (d, *J* = 5.2 Hz, 1H), 3.98 (s, 1H), 3.68 (s, 3H), 3.60 (dd, *J* = 9.6, 4.8 Hz, 1H), 3.48 (d, *J* = 4.4 Hz, 1H), 3.45 (br s, 2H), 3.42 (d, *J* = 4.8 Hz, 1H), 3.06 (dd, *J* = 13.6, 5.2 Hz, 1H), 2.75 (br s, 1H), 2.24 (dd, *J* = 12.0, 4.8 Hz, 1H), 1.74 (d, *J* = 12.0 Hz, 1H), 1.58 (d, *J* = 6.8 Hz, 3H), 1.50 (dd, *J* = 13.6, 9.6 Hz, 1H).

^{13}C NMR (100 MHz, $CDCl_3$): δ 175.5, 152.1, 137.0, 130.1, 128.6, 122.3, 120.6, 116.0, 111.8, 86.5, 70.1, 61.7, 55.7, 55.3, 54.8, 54.3, 52.1, 36.4, 30.3, 23.7, 13.1.

HRMS (ESI): Calcd for $C_{21}H_{25}N_2O_3$ $[M+H]^+$: 353.1860, found: 353.1859.

1.2.25 Synthesis of Vincamedine (7)

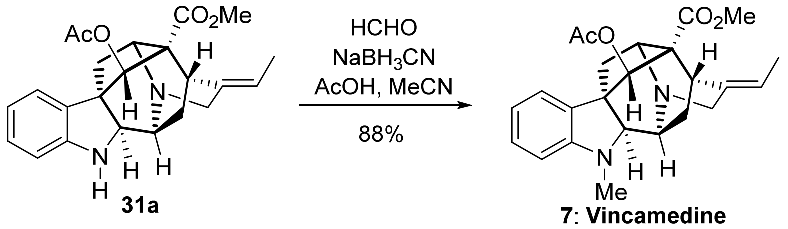

Compound **31a** (11.0 mg, 0.028 mmol) was dissolved in MeCN (2.4 mL). To this mixture, HCHO (37% in H_2O, 0.5 mL), AcOH (0.27 mL) and $NaBH_3CN$ (9.1 mg, 0.14 mmol) were added at room temperature. After being stirred at room temperature for 4 h, the reaction was quenched with saturated *aq.* $NaHCO_3$ (15 mL). The mixture was extracted with CH_2Cl_2 (3 × 8 mL). The combined organic phases were washed with brine (10 mL), dried over Na_2SO_4, filtered, and concentrated. The residue was

purified by flash column chromatography on silica gel (CH_2Cl_2/MeOH = 20:1) to afford Vincamedine (**7**, 10.2 mg, 88%) as a yellowish solid.

M.p.: 161-162 °C.

R*f* = 0.48 (CH_2Cl_2 : MeOH = 25:1).

$[\alpha]_D^{20}$ = –48.1 (*c* 0.82, $CHCl_3$). Lit[7]: **$[\alpha]_D$**= –66 (*c* 0.52, $CHCl_3$).

FTIR (KBr, thin film) cm^{-1} : 3441, 2951, 2912, 2856, 1751, 1725, 1609, 1478, 1466, 1368, 1294, 1231, 1133, 1113, 1092, 1029, 976, 830, 780, 760.

^{1}H NMR (400 MHz, $CDCl_3$): δ 7.17 (t, *J* = 7.6 Hz, 1H), 6.98 (d, *J* = 6.8 Hz, 1H), 6.72 (t, *J* = 7.6 Hz, 1H), 6.66 (d, *J* = 8.0 Hz, 1H), 5.67 (s, H), 5.28 (q, *J* = 6.8 Hz, 1H), 3.66 (s, 3H), 3.61 (d, *J* = 4.4 Hz, 1H), 3.56 (dd, *J* = 9.6, 4.8 Hz, 1H), 3.50 (d, *J* = 4.8 Hz, 1H), 3.50-3.41 (m, 2H), 3.21 (d, *J* = 4.8 Hz, 1H), 2.65 (s, 3H), 2.62 (dd, *J* = 10.0, 4.8 Hz, 1H), 2.55 (dd, *J* = 11.6, 4.8 Hz, 1H), 1.87 (s, 3H), 1.78 (d, *J* = 11.2 Hz, 1H), 1.55 (d, *J* = 7.6 Hz, 3H), 1.52 (d, *J* = 10.0 Hz, 1H).

^{13}C NMR (100 MHz, $CDCl_3$): δ 172.3, 168.4, 154.3, 136.6, 128.9, 128.6, 123.3, 118.9, 116.8, 109.3, 75.5, 74.8, 61.6, 58.9, 56.1, 55.5, 53.2, 51.6, 36.5, 34.1, 30.4, 21.8, 20.7, 12.7.

HRMS (ESI): Calcd for $C_{24}H_{29}N_2O_4$ $[M+H]^+$: 409.2122, found: 409.2125.

1.2.26 Synthesis of Vincamajine (6)

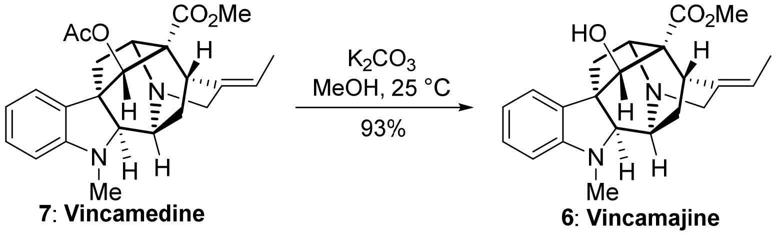

To a solution of acetate **7** (7.4 mg, 0.018 mmol) in MeOH (2 mL) was added K_2CO_3 (38.0 mg, 0.272 mmol) at room temperature. After being stirred at room temperature for 24 h, the reaction was quenched with saturated *aq.* NH_4Cl (2 mL) and diluted with water (8 mL). The mixture was then extracted with CH_2Cl_2 (3 × 5 mL). The combined organic phases were washed with brine (8 mL), dried over anhydrous Na_2SO_4, filtered, and concentrated. The residue was purified by column chromatography on silica gel

(CH_2Cl_2/MeOH = 20:1) to give Vincamajine (**6**, 6.2 mg, 93%) as a white solid.

M.p.: 107-109 °C.

R*f* = 0.41 (CH_2Cl_2 : MeOH = 25:1).

$[\alpha]_D^{20}$ = –10.8 (*c* 0.59, $CHCl_3$). Lit[8]: **$[\alpha]_D$**= –16 (*c* 1, 5% EtOH in $CHCl_3$).

FTIR (KBr, thin film) cm^{-1} : 3440, 2948, 2856, 1732, 1610, 1478, 1464, 1291, 1243, 1113, 1090, 1075, 1058, 742.

^{1}H NMR (400 MHz, $CDCl_3$): δ 7.18 (td, *J* = 7.6, 0.8 Hz, 1H), 7.15 (d, *J* = 6.8 Hz, 1H), 6.79 (t, *J* = 7.2 Hz, 1H), 6.65 (d, *J* = 8.0 Hz, 1H), 5.25 (q, *J* = 6.8 Hz, 1H), 4.24 (s, H), 3.69 (s, 3H), 3.53 (d, *J* = 4.8 Hz, 1H), 3.50-3.47 (m, 2H), 3.42-3.40 (m, 2H), 3.21 (d, *J* = 5.2 Hz, 1H), 2.64 (s, 3H), 2.61 (dd, *J* = 11.6, 4.8 Hz, 1H), 2.43 (dd, *J* = 14.0, 4.8 Hz, 1H), 1.99 (br s, 1H), 1.68 (d, *J* = 11.6 Hz, 1H), 1.59 (br d, *J* = 6.8 Hz, 3H), 1.49 (dd, *J* = 13.6, 10.0 Hz, 1H).

^{13}C NMR (100 MHz, $CDCl_3$): δ 173.1, 154.5, 136.9, 130.0, 128.5, 124.1, 119.1, 116.4, 109.1, 75.0 74.8, 61.7, 59.5, 57.1, 55.5, 53.1, 51.6, 35.6, 34.3, 30.2, 22.0, 12.8.

HRMS (ESI): Calcd for $C_{22}H_{27}N_2O_3$ $[M+H]^+$: 367.2016, found: 367.2014.

1.2.27 Synthesis of Quebrachidine (5)

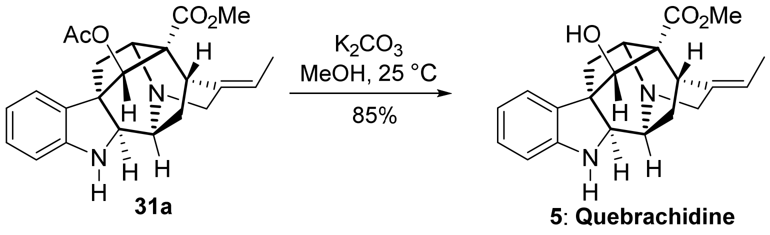

To a solution of acetate **31a** (12.0 mg, 0.03 mmol) in MeOH (1 mL) was added K_2CO_3 (41.5 mg, 0.3 mmol) at room temperature. After being stirred at room temperature for 36 h, the reaction was quenched with saturated *aq.* NH_4Cl (2 mL) and diluted with water (8 mL). The mixture was then extracted with CH_2Cl_2 (3 × 5 mL). The combined organic phases were washed with brine (8 mL), dried over anhydrous Na_2SO_4, filtered, and concentrated under reduced pressure. The residue was purified by flash column chromatography on silica gel (CH_2Cl_2/MeOH = 20:1) to afford

Quebrachidine (**5**, 9.3 mg, 85%) as a yellowish solid.

M.p.: 238-240 °C.

R*f* = 0.32 (CH_2Cl_2 : MeOH = 25:1).

$[\alpha]_D^{20}$ = 10.7 (*c* 0.11, MeOH). Lit[9]: **$[\alpha]_D^{20}$** = 14 (MeOH).

FTIR (KBr, thin film) cm^{-1} : 3433, 1726, 1632, 1462, 1311, 1241, 1131, 1058, 888, 743.

^{1}H NMR (400 MHz, MeOD): δ 7.04 (d, *J* = 7.2 Hz, 1H), 6.93 (t, *J* = 7.8 Hz, 1H), 6.63 (d, *J* = 8.0 Hz, 1H), 6.62 (t, *J* = 8.0 Hz, 1H), 5.20 (q, *J* = 6.4 Hz, 1H), 4.12 (s, 1H), 3.56 (s, 3H), 3.56-3.55 (m, 1H), 3.45-3.42 (m, 3H), 3.39 (d, *J* = 10.8 Hz, 1H), 3.34 (d, *J* = 15.6 Hz, 1H), 2.54 (dd, *J* = 14.0, 4.8 Hz, 1H), 2.50 (dd, *J* = 12.0, 4.8 Hz, 1H), 1.55 (d, *J* = 12.0 Hz, 1H), 1.51 (d, *J* = 6.4 Hz, 3H), 1.44 (dd, *J* = 14.0, 10.0 Hz, 1H).

^{13}C NMR (100 MHz, MeOD): δ 174.4, 153.6, 137.3, 131.0, 128.8, 126.5, 120.1, 117.7, 111.7, 75.0, 69.7, 62.8, 61.6, 58.9, 56.2, 55.9, 52.0, 36.8, 31.3, 22.8, 13.1.

^{1}H NMR (400 MHz, $CDCl_3$): δ 7.17 (d, *J* = 7.2 Hz, 1H), 7.11 (td, *J* = 7.6, 1.2 Hz, 1H), 6.80 (t, *J* = 7.6 Hz, 1H), 6.77 (d, *J* = 7.6 Hz, 1H), 5.25 (q, *J* = 6.8 Hz, 1H), 4.27 (s, 1H), 3.85 (d, *J* = 4.8 Hz, 1H), 3.69 (s, 3H), 3.55 (d, *J* = 4.4 Hz, 1H), 3.50-3.45 (m, 2H), 3.41-3.36 (m, 2H), 2.62 (dd, *J* = 12.0, 4.8 Hz, 1H), 2.56 (dd, *J* = 14.0, 4.8 Hz, 1H), 2.13 (br s, 2H), 1.74 (d, *J* = 11.6 Hz, 1H), 1.59 (d, *J* = 6.8 Hz, 3H), 1.50 (dd, *J* = 13.6, 10.0 Hz, 1H).

^{13}C NMR (100 MHz, $CDCl_3$): δ 173.1, 151.8, 136.6, 129.6, 128.5, 124.9, 119.8, 111.1, 74.5, 68.5, 61.8, 59.6, 57.9, 55.3, 54.8, 51.8, 35.7, 30.5, 22.4, 13.0.

HRMS (ESI): Calcd for $C_{21}H_{25}N_2O_3$ $[M+H]^+$: 353.1860, found: 353.1863.

1.2.28 Synthesis of Vincamajinine (9)

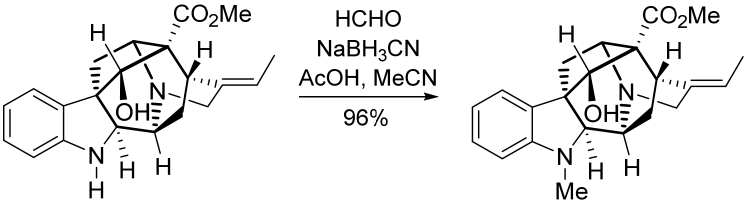

8: **Vincarine** **9**: **Vincamajinine**

Compound **8** (9.0 mg, 0.026 mmol) was dissolved in MeCN (2 mL). To this mixture, HCHO (37% in H_2O, 0.5 mL), AcOH (0.24 mL) and $NaBH_3CN$ (8.0 mg, 0.128 mmol) were added at room temperature. After being stirred at room temperature for 4 h, the reaction was quenched with saturated *aq.* $NaHCO_3$ (15 mL). The mixture was extracted with CH_2Cl_2 (3 × 8 mL). The combined organic phases were washed with brine (10 mL), dried over anhydrous Na_2SO_4, filtered, and concentrated. The residue was purified by flash column chromatography on silica gel (CH_2Cl_2/MeOH = 20:1) to afford Vincamajinine (**9**, 9.1 mg, 96%) as a white solid.

M.p.: 198-200 °C.

R*f* = 0.39 (CH_2Cl_2 : MeOH = 25:1).

$[\alpha]_D^{20}$ = –8.6 (*c* 0.96, $CHCl_3$). Lit[10]: $[\alpha]_D$= –9 ($CHCl_3$); Lit[11]: $[\alpha]_D$= –4.00 (*c* 0.3, $CHCl_3$).

FTIR (KBr, thin film) cm^{-1} : 3435, 2949, 2856, 2807, 1726, 1609, 1479, 1464, 1295, 1239, 1219, 1110, 1091, 1058, 973, 828, 749.

^{1}H NMR (400 MHz, $CDCl_3$): δ 7.20 (t, *J* = 7.6 Hz, 1H), 7.07 (d, *J* = 7.2 Hz, 1H), 6.85 (t, *J* = 7.6 Hz, 1H), 6.74 (d, *J* = 8.0 Hz, 1H), 5.24 (q, *J* = 6.8 Hz, 1H), 4.00 (s, 1H), 3.69 (s, 3H), 3.64 (dd, *J* = 9.6, 4.8 Hz, 1H), 3.50-3.48 (m, 3H), 3.43 (d, *J* = 4.4 Hz, 1H), 3.37 (d, *J* = 5.2 Hz, 1H), 2.91 (dd, *J* = 13.6, 5.2 Hz, 1H), 2.64 (s, 3H), 2.64 (br s, 1H), 2.26 (dd, *J* = 12.0, 4.8 Hz, 1H), 1.74 (d, *J* = 11.6 Hz, 1H), 1.59 (d, *J* = 6.8 Hz, 3H), 1.51 (dd, *J* = 13.6, 9.6 Hz, 1H).

^{13}C NMR (100 MHz, $CDCl_3$): δ 175.4, 154.7, 136.9, 130.4, 128.5, 121.6, 120.0, 115.8, 109.8, 86.2, 76.3, 61.7, 55.6, 54.1, 53.8, 53.5, 51.9, 36.1, 34.9, 29.9, 23.1, 12.9.

HRMS (ESI): Calcd for $C_{22}H_{27}N_2O_3$ $[M+H]^+$: 367.2016, found: 367.2014.

1.2.29 Synthesis of Indole 32

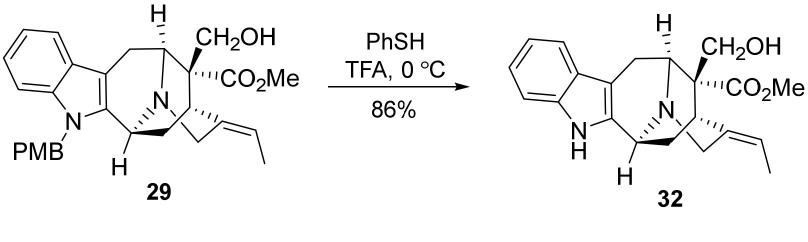

To a mixture of compound **29** (144.0 mg, 0.305 mmol) in PhSH (1.4 mL) was added

TFA (14 mL) at 0 °C. After being stirred at 0 °C for 3 d, the mixture was poured into ice-water (130 mL) containing sodium bicarbonate (16.0 g, > 0.19 mol) and extracted with EtOAc (4 × 20 mL). The combined organic phases were washed with brine (20 mL), dried over Na_2SO_4, filtered, and concentrated under reduced pressure. The residue was purified by flash column chromatography on silica gel (CH_2Cl_2/MeOH = 20:1) to afford indole **32** (92.2 mg, 86%) as a yellowish solid.

M.p.: 225-227 °C.

$\mathbf{R}_f$ = 0.27 (ethyl acetate).

$[\alpha]_D^{20}$ = –59.0 (*c* 0.24, $CHCl_3$).

FTIR (KBr, thin film) cm^{-1} : 3416, 2951, 1727, 1678, 1454, 1319, 1207, 1145, 1088, 1046, 843, 801, 743.

^{1}**H NMR** (400 MHz, $CDCl_3$): δ 8.23 (br s, 1H), 7.45 (d, *J* = 7.6 Hz, 1H), 7.28 (d, *J* = 8.0 Hz, 1H), 7.14 (t, *J* = 7.2 Hz, 1H), 7.09 (t, *J* = 7.2 Hz, 1H), 5.26-5.22 (m, 1H), 4.27 (d, *J* = 6.4 Hz, 1H), 4.01 (dd, *J* = 10.0, 2.8 Hz, 1H), 3.71 (s, 3H), 3.67-3.62 (m, 2H), 3.52 (d, *J* = 11.2 Hz, 1H), 3.41 (d, *J* = 17.2 Hz, 1H), 3.07 (dd, *J* = 16.4, 6.0 Hz, 1H), 2.94 (d, *J* = 16.4 Hz, 1H), 2.65 (br s, 1H), 1.89-1.84 (m, 1H), 1.75 (br d, *J* = 13.2 Hz, 1H), 1.48 (d, *J* = 6.8 Hz, 3H).

^{13}**C NMR** (100 MHz, $CDCl_3$): δ 176.6, 137.1, 136.8, 136.3, 126.5, 121.8, 119.6, 118.5, 116.9, 111.1, 105.8, 63.1, 54.3, 53.8, 53.5, 52.5, 49.0, 38.0, 29.5, 22.3, 12.7.

HRMS (ESI): Calcd for $C_{21}H_{25}N_2O_3$ $[M+H]^+$: 353.1860, found: 353.1864.

1.2.30 Synthesis of the Mixture of 34a and 34b

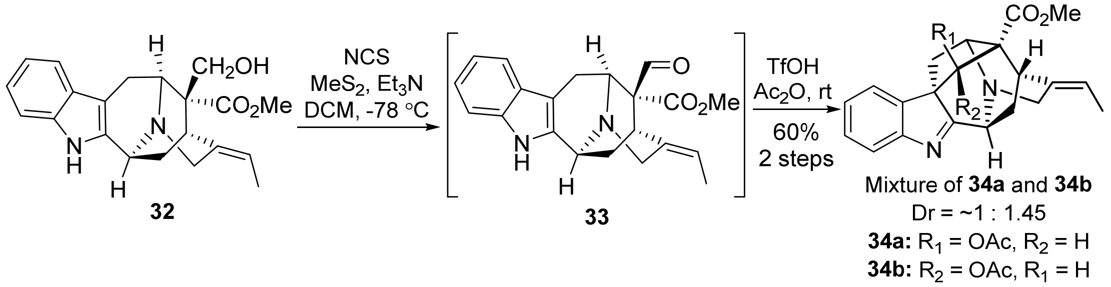

Dimethyl sulfide (329 μL, 4.54 mmol) was added to a solution of *N*-chlorosuccinimide (122.0 mg, 0.43 mmol) in dry CH_2Cl_2 (10 mL) at 0 °C under Ar,

and white precipitate appeared immediately after the addition. The resulting mixture was then stirred at -78 ºC for 10 min. To this mixture, a solution of **32** (64.0 mg, 0.182 mmol) in dry CH_2Cl_2 (20 mL) was introduced at -78 ºC, and the reaction was continued at -78 ºC for 30 min. A solution of triethylamine (253 μL, 1.82 mmol) in CH_2Cl_2 (2 mL) was added. After being stirred at 0 ºC for 30 min, the reaction was quenched with saturated *aq.* NH_4Cl (5 mL) and diluted with water (20 mL). The mixture was extracted with CH_2Cl_2 (3 × 15 mL). The combined organic phases were washed with brine (25 mL), dried over Na_2SO_4, filtered, and concentrated under reduced pressure (water bath temperature must be below 25 ºC). The residue was purified by flash column chromatography on silica gel (CH_2Cl_2/MeOH = 20:1) to afford **33** which was confirmed by HRMS (Calcd for $C_{21}H_{23}N_2O_3$ $[M+H]^+$: 351.1703, found: 351.1707) and used directly in the next step. To the resulting aldehyde in Ac_2O (0.6 mL) was added cold CF_3SO_3H (182 μL, 1.82 mmol) at room temperature under Ar. After being stirred at room temperature for 10 min, the resulting mixture was poured into a cold saturated aqueous solution of sodium bicarbonate (20 mL), and extracted with EtOAc (3 × 15 mL). The combined organic phases were washed with brine (20 mL), dried over Na_2SO_4, filtered, and concentrated under reduced pressure. The residue was purified by preparative TLC on silica gel (hexane /EtOAc = 1:3) to give an inseparable mixture of **34a** and **34b** (43.1 mg, 60% from **32**, dr = ~ 1:1.45).

^{1}H NMR (400 MHz, $CDCl_3$): δ 7.58-7.37 (m, 2.45H), 7.37-7.33 (m, 3.9H), 7.24-7.15 (m, 3.45H), 5.93 (s, 1.45H), 5.56 (s, 1H), 5.38-5.30 (m, 2.45H), 4.30 (d, *J* = 9.2 Hz, 1.45H), 4.19 (d, *J* = 8.4 Hz, 1H), 3.82 (d, *J* = 4.4 Hz, 1H), 3.76 (s , 4.35H), 3.76-3.70 (m, 2.45H), 3.71 (s , 3H), 3.56 (d, *J* = 4.4 Hz, 1.45H), 3.47 (d, *J* = 16.8 Hz, 1.45H), 3.41 (d, *J* = 16.8 Hz, 1H), 3.14 (d, *J* = 4.4 Hz, 1H), 2.90 (d, *J* = 4.4 Hz, 1.45H), 2.82 (dd, *J* = 12.0, 4.8 Hz, 1H), 2.62 (dd, *J* = 12.0, 4.8 Hz, 1.45H), 2.39 (dd, *J* = 13.6, 4.8 Hz, 1H), 2.36 (d, *J* = 12.4 Hz, 1.45H), 2.12 (s, 3H), 2.03-1.97 (m, 4.35H), 1.81 (s, 4.35H), 1.71 (d, *J* = 10.8 Hz, 1H), 1.68 (d, *J* = 12.0 Hz, 1.45H), 1.54 (d, *J* = 6.8 Hz, 3H), 1.54 (d, *J* = 6.8 Hz, 4.35H).

^{13}C NMR (100 MHz, $CDCl_3$): δ 183.6 (182.6), 174.5 (171.9), 169.7 (168.5), 156.5, 136.1 (136.0), 134.9 (135.6), 128.8 (129.0), 125.7 (125.8), 122.5 (123.2), 120.9

(121.3), 118.13 (118.08), 78.6 (75.9), 64.1 (64.9), 62.0 (61.1), 61.6, 55.8 (55.3), 53.9, 52.7 (52.3), 51.2 (50.8), 36.6 (37.6), 36.4 (38.5), 28.7 (27.3), 20.7 (21.0), 12.7.

1.2.31 Synthesis of the Mixture of 35a and 35b

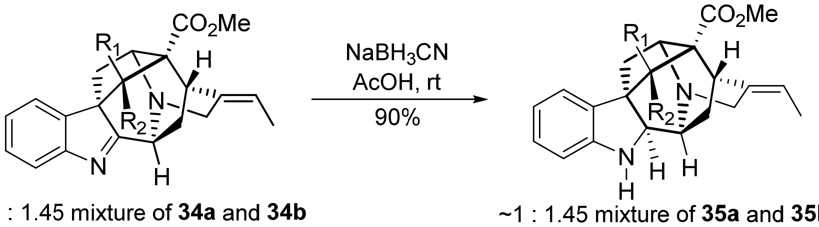

~1 : 1.45 mixture of **34a** and **34b**
34a: R_1 = OAc, R_2 = H
34b: R_2 = OAc, R_1 = H

~1 : 1.45 mixture of **35a** and **35b**
35a: R_1 = OAc, R_2 = H
35b: R_2 = OAc, R_1 = H

To a mixture of **34a** and **34b** (27.0 mg, 0.069 mmol) in AcOH (0.6 mL) was added $NaBH_3CN$ (8.6 mg, 0.138 mmol) at room temperature. After being stirred at room temperature for 1 h, the mixture was poured into a cold saturated aqueous solution of sodium bicarbonate (20 mL), and extracted with CH_2Cl_2 (3 × 10 mL). The combined organic phases were washed with brine (10 mL), dried over Na_2SO_4, filtered, and concentrated under reduced pressure. The residue was purified by flash column chromatography on silica gel (CH_2Cl_2/MeOH = 20:1) to afford an inseparable mixture of **35a** and **35b** (~ 1:1.45, 24.5 mg, 90%).

^{1}H NMR (400 MHz, $CDCl_3$): δ 7.09-7.06 (m, 3.9H), 6.99 (d, *J* = 6.8 Hz, 1H), 6.78 (d, *J* = 7.6 Hz, 1.45H), 6.74-6.71 (m, 3.45H), 5.65 (s, 1H), 5.31-5.24 (m, 3.9H), 4.01 (d, *J* = 4.4 Hz, 1.45H), 3.85 (br s, 2H), 3.67 (s , 4.35H), 3.67-3.65 (m, 2H), 3.65 (s, 3H), 3.61-3.57 (m, 2.9H), 3.48 (br s, 1.45H), 3.42-3.29 (m, 3.9H), 3.09 (dd, *J* = 13.6 4.8 Hz, 1.45H), 3.01 (d, *J* = 4.4 Hz, 1H), 2.80 (d, *J* = 12.0 Hz, 1.45H), 2.75 (dd, *J* = 14.4, 4.8 Hz, 1H), 2.57 (dd, *J* = 10.8, 4.8 Hz, 1H), 2.49 (dd, *J* = 12.0, 4.8 Hz, 1.45H), 1.86 (d, *J* = 9.2 Hz, 1.45H), 1.85 (s, 3H), 1.81 (d, *J* = 12.4 Hz, 1H), 1.79 (s, 4.35H), 1.54-1.50 (m, 2.45H), 1.51 (d, *J* = 6.8 Hz, 3H), 1.51 (d, *J* = 6.8 Hz, 4.35H).

^{13}C NMR (100 MHz, $CDCl_3$): δ 174.8 (172.6), 169.7 (168.6), 152.3 (151.7), 137.6 (137.8), 128.6, 128.53 (128.51), 123.0 (123.9), 120.0 (119.4), 116.9 (117.0), 111.14 (111.17), 83.7 (75.3), 69.6 (68.8), 63.0 (62.2), 57.0 (59.5), 55.1 (54.5), 54.1 (53.2), 52.7 (52.3), 52.3 (52.0), 37.3 (36.9), 36.2 (37.4), 24.3 (23.3), 20.8 (20.9), 12.7.

1.2.32 Synthesis of Ester 35a and Alcohol 36

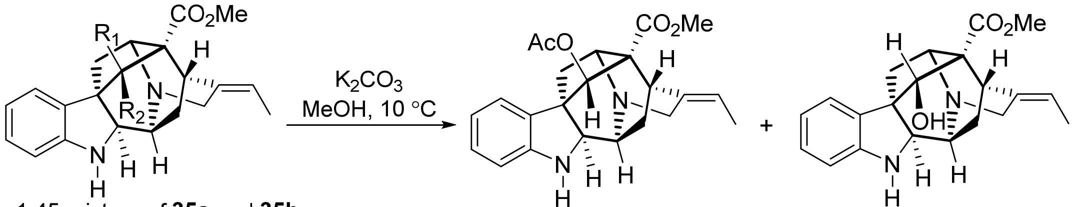

~1 : 1.45 mixture of **35a** and **35b**
35a: R_1 = OAc, R_2 = H
35b: R_1 = OAc, R_2 = H

35a: 37%

36: 59%

To a solution of **35a** and **35b** (19.0 mg, 0.048 mmol) in MeOH (2 mL) was added K_2CO_3 (20.0 mg, 0.145 mmol) at 10 °C. After being stirred at 10 °C for 5 h, the reaction was quenched with saturated *aq.* NH_4Cl (1 mL) and diluted with water (15 mL). The mixture was then extracted with CH_2Cl_2 (3 × 8 mL). The combined organic phases were washed with brine (10 mL), dried over Na_2SO_4, filtered, and concentrated under reduced pressure. The residue was purified by flash column chromatography on silica gel (CH_2Cl_2/MeOH = 25:1) to yield ester **35a** (7.0 mg, 37%) as a white solid. Futher elution provides alcohol **36** (10.1 mg, 59%) as a white solid.

35a:

M.p.: 91-92 °C.

R*f* = 0.47 (CH_2Cl_2 : MeOH = 25:1).

$[\alpha]_D^{20}$ = –13.9 (*c* 0.85, $CHCl_3$).

FTIR (KBr, thin film) cm^{-1} : 3439, 2949, 1737, 1613, 1464, 1435, 1373, 1290, 1244, 1136, 1065, 1027, 824, 755, 742.

^{1}H NMR (400 MHz, $CDCl_3$): δ 7.08 (td, *J* = 7.6, 1.2 Hz, 1H), 6.99 (d, *J* = 6.8 Hz, 1H), 6.75 (d, *J* = 8.0 Hz, 1H), 6.73 (t, *J* = 7.6 Hz, 1H), 5.65 (d, *J* = 1.2 Hz, 1H), 5.28-5.23 (m, 1H), 3.86-3.83 (m, 2H), 3.67 (d, *J* = 14.8 Hz, 1H), 3.65 (s, 3H), 3.58 (d, *J* = 4.4 Hz, 1H), 3.50 (dd, *J* = 9.2, 4.8 Hz, 1H), 3.31 (d, *J* = 16.4 Hz, 1H), 3.02 (d, *J* = 4.8 Hz, 1H), 2.76 (dd, *J* = 14.4, 4.8 Hz, 1H), 2.57 (dd, *J* =11.6, 4.8 Hz, 1H), 1.86 (s, 3H), 1.81 (d, *J* = 11.6 Hz, 1H), 1.56 (d, *J* = 10.0 Hz, 1H), 1.52 (d, *J* = 6.4 Hz, 3H).

^{13}C NMR (100 MHz, $CDCl_3$): δ 172.6, 168.6, 151.7, 137.9, 128.63, 128.60, 124.0, 119.5, 117.0, 111.2, 75.4, 68.8, 62.3, 59.6, 57.0, 54.6, 52.3, 52.0, 37.4, 36.9, 23.3, 20.9, 12.8.

HRMS (ESI): Calcd for $C_{23}H_{27}N_2O_4$ $[M+H]^+$: 395.1965, found: 395.1969.

36:

M.p.: 128-130 °C.

R*f* = 0.36 (CH_2Cl_2 : MeOH = 25:1).

$[\alpha]_D^{20}$ = 42.2 (*c* 0.82, $CHCl_3$).

FTIR (KBr, thin film) cm^{-1} : 3436, 2949, 1723, 1611, 1481, 1463, 1435, 1381, 1308, 1242, 1214, 1114, 1094, 1054, 825, 750.

^{1}H NMR (400 MHz, $CDCl_3$): δ 7.12 (td, *J* = 7.6, 1.2 Hz, 1H), 7.07 (d, *J* = 7.2 Hz, 1H), 6.84 (t, *J* = 7.6 Hz, 1H), 6.82 (d, *J* = 8.0 Hz, 1H), 5.29-5.23 (m, 1H), 4.02 (d, *J* = 4.8 Hz, 1H), 3.98 (s, 1H),3.82 (br s, 1H), 3.68 (s, 3H), 3.65 (d, *J* = 16.8 Hz, 1H), 3.59 (dd, *J* = 9.6, 4.8 Hz, 1H), 3.44 (d, *J* = 4.8 Hz, 1H), 3.34 (d, *J* = 16.8 Hz, 1H), 3.09 (dd, *J* = 13.6, 5.2 Hz, 1H), 2.95 (d, *J* = 4.4 Hz, 1H), 2.62 (br s, 1H), 2.27 (dd, *J* = 12.0, 4.8 Hz, 1H), 1.77 (d, *J* = 12.0 Hz, 1H), 1.52 (d, *J* = 6.8 Hz, 3H), 1.51 (dd, *J* = 13.6, 9.6 Hz, 1H).

^{13}C NMR (100 MHz, $CDCl_3$): δ 175.7, 152.1, 138.1, 130.1, 128.6, 122.3, 120.6, 116.2, 111.8, 86.5, 70.2, 62.4, 55.1, 54.9, 54.8, 52.6, 52.3, 36.9, 36.7, 24.6, 12.7.

HRMS (ESI): Calcd for $C_{21}H_{25}N_2O_3$ $[M+H]^+$: 353.1860, found: 353.1858.

1.2.33 Synthesis of Aldehyde 33 from Alcohol 36

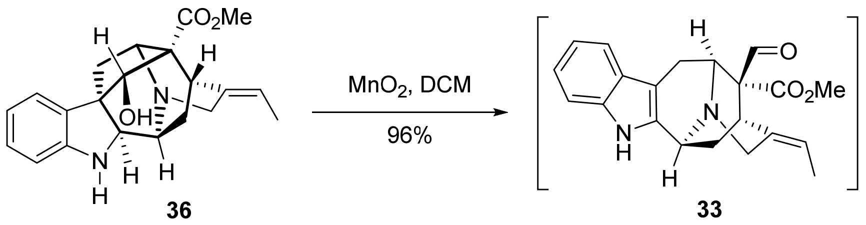

To a solution of alcohol **36** (10.0 mg, 0.0284 mmol) in DCM (2 mL) was added manganese oxide (49.4 mg, 0.56 mmol). After 1 h stirring, the reaction mixture was filtered through a short column of silica gel and washed with dichloromethane/methanol (95/5, 20 mL). The filtrate was concentrated under reduced pressure affording aldehyde **33** as a yellowish solid (9.5 mg, 96%). Aldehyde **33**

decomposed after a few hours in solution via NMR analysis and full analysis had to be recorded within 3 h.

^{1}H NMR (400 MHz, DMSO-d_6): δ 8.91 (s, 1H), 7.33 (d, J = 7.6 Hz, 1H), 7.27 (d, J = 8.0 Hz, 1H), 7.03 (t, J = 7.6 Hz, 1H), 6.94 (d, J = 7.2 Hz, 1H), 5.23 (q, J = 6.8 Hz, 1H), 4.16 (d, J = 9.6 Hz, 1H), 4.00 (br d, J = 2.8 Hz, 1H), 3.61 (s, 3H), 3.61-3.58 (m, 1H), 2.94-2.86 (m, 3H), 2.16 (br d, J = 12.8 Hz, 1H), 1.87 (t, J = 12.0 Hz, 1H), 1.50 (d, J = 6.4 Hz, 3H).

^{13}C NMR (100 MHz, $CDCl_3$): δ 196.1, 171.1, 139.0, 137.6, 136.9, 126.1, 121.4, 119.0, 118.2, 116.7, 111.6, 104.3, 63.5, 57.8, 53.2, 52.8, 49.0, 36.4, 28.7, 23.5, 12.8.

HRMS (ESI): Calcd for $C_{21}H_{23}N_2O_3$ $[M+H]^+$: 351.1703, found: 351.1707.

1.2.33 Synthesis of Alstiphyllanine J (11) and its trifluoroacetic acid salt

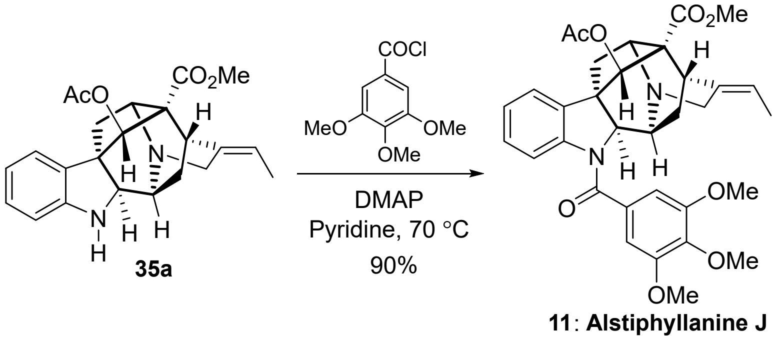

To a solution of **35a** (5.6 mg, 0.014 mmol) in anhydrous pyridine (0.5 mL) were added DMAP (3 × 17.0 mg, 0.426 mmol) and 3,4,5-trimethoxybenzoyl chloride (3 × 19.6 mg, 0.255 mmol) in 3 portions (in three-hour interval) at 60 ºC under Ar. The progress of the reaction was monitored by TLC. After complete consumption of amine **35a**, saturated *aq.* $NaHCO_3$ (5 mL) was added to the reaction system at room temperature, and the resulting mixture was diluted with water (5 mL) and extracted with CH_2Cl_2 (3 × 6 mL). The combined organic phase were washed with brine (10 mL), dried over Na_2SO_4, filtered, and concentrated under reduced pressure. The residue was purified by flash column chromatography on silica gel (CH_2Cl_2/MeOH = 25:1) to afford Alstiphyllanine J (**11**, 7.5 mg, 90%).

M.p.: 107-108 ºC.

R$_f$ = 0.53 (CH_2Cl_2 : MeOH = 25:1).

$[\alpha]_D^{20}$ = –176.2 (*c* 0.64, $CHCl_3$).

FTIR (KBr, thin film) cm^{-1} : 3441, 2947, 1744, 1652, 1585, 1504, 1478, 1461, 1415, 1379, 1342, 1235, 1127, 1029, 755.

^{1}H NMR (400 MHz, MeOD): δ 7.14 (dd, *J* = 6.0, 2.4 Hz, 1H), 7.01-6.96 (m, 2H), 6.95-6.90 (m, 2H), 6.18 (d, *J* = 6.8 Hz, 1H), 5.93 (s, 1H), 5.31-5.27 (m, 1H), 4.43 (dd, *J* = 9.6, 4.8 Hz, 1H), 4.19 (d, *J* = 4.8 Hz, 1H), 3.83-3.81 (m, 6H), 3.76-3.64 (m, 8H), 3.40 (d, *J* = 16.4 Hz, 1H), 3.01 (d, *J* = 4.8 Hz, 1H), 2.57 (dd, *J* = 12.0, 4.8 Hz, 1H), 2.33 (dd, *J* = 14.4, 4.8 Hz, 1H), 1.82 (s, 3H), 1.81 (d, *J* =17.6 Hz, 1H), 1.67 (dd, *J* = 14.4, 10.0 Hz, 1H), 1.53 (d, *J* = 6.8 Hz, 3H).

^{13}C NMR (100 MHz, MeOD): δ 173.2, 171.0, 170.1, 154.8, 145.9, 142.7, 137.7, 131.8, 131.4, 129.0, 124.9, 124.5, 118.9, 117.1, 107.5, 105.6, 76.1, 71.5, 63.1, 61.3, 60.6, 58.1, 56.9, 56.8, 54.1, 52.8, 52.7, 38.2, 35.8, 24.8, 20.7, 12.6.

HRMS (ESI): Calcd for $C_{33}H_{37}N_2O_8$ $[M+H]^+$: 589.2544, found: 589.2539.

Alstiphyllanine J (11)·TFA: To a solution of Alstiphyllanine J (5.6 mg, 0.0095 mmol) in CH_2Cl_2 (1 mL) was added trifluoroacetic acid (190 μL, 0.1 N in CH_2Cl_2, 0.019 mmol). After being stirred at room temperature for 30 min, the mixture was concentrated under reduced pressure to give trifluoroacetic acid salt of Alstiphyllanine J (**11**).

$[\alpha]_D^{20}$ = –144.3 (*c* 0.61, MeOH). Lit[12]: **$[\alpha]_D^{26}$** = –94 (*c* 1.0, MeOH).

^{1}H NMR (400 MHz, MeOD): δ 7.23 (dd, *J* = 7.6, 1.6 Hz, 1H), 7.05-6.96 (m, 4H), 6.21 (d, *J* = 7.2 Hz, 1H), 6.03 (s, 1H), 5.59-5.58 (m, 1H), 5.20 (dd, *J* = 10.0, 4.8 Hz, 1H), 4.46 (d, *J* = 4.0 Hz, 1H), 4.37 (d, *J* = 15.2 Hz, 1H), 4.69 (d, *J* = 5.2 Hz, 1H), 4.12 (d, *J* = 15.2 Hz, 1H), 3.83 (br s, 6H), 3.76 (br s, 6H), 3.39 (d, *J* = 4.8 Hz, 1H), 2.86 (dd, *J* = 13.6, 4.4 Hz, 1H), 2.58 (dd, *J* = 15.2, 5.2 Hz, 1H), 2.20 (d, *J* = 13.2 Hz, 1H), 2.07 (dd, *J* = 14.8, 10.4 Hz, 1H), 1.88 (s, 3H), 1.60 (d, *J* = 6.8 Hz, 3H).

^{13}C NMR (100 MHz, MeOD): δ 170.8, 170.5, 169.7, 155.1, 145.3, 143.1, 130.5, 129.8, 129.6, 128.6, 125.2, 125.1, 123.9, 117.2, 107.5, 74.8, 68.3, 64.8, 61.3, 60.1, 57.2, 57.1, 56.9, 53.5, 52.2, 36.6, 34.2, 22.5, 20.5, 12.9.

1.2.34 Studies on the epimerization of the C-16 stereochemistry from 25

Supplementary Table 2 Attempted conditions for epimerization of the C-16 stereochemistry from **25**

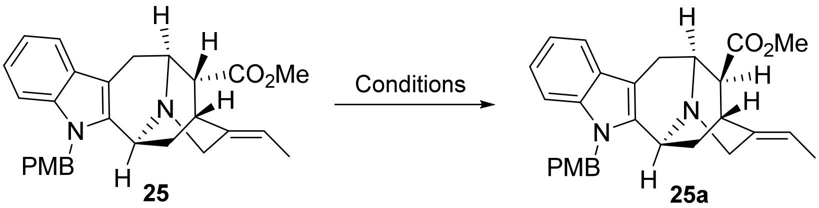

Entry	Base	Solvent	Additive	Proton source	Temperature	Results
1[a]	DBU	Toluene	-	-	Reflux	Recovery of **25**
2[a]	^{t}BuOK	THF	-	-	Reflux	Recovery of **25**
3[b]	LiHMDS	THF		MeOH	0 °C to –78 °C	Recovery of **25**
4[b]	NaHMDS	THF	-	MeOH	0 °C to –78 °C	Recovery of **25**
5[b]	KHMDS	THF	-	MeOH	0 °C to –78 °C	Recovery of **25**
6[b]	LDA	THF	-	MeOH	0 °C to –78 °C	Recovery of **25**
7[b]	LDA	THF	-	^{t}BuOH	0 °C to –78 °C	Recovery of **25**
8[b]	LDA	THF	-	BHT	0 °C to –78 °C	Recovery of **25**
9[b]	LDA	THF	-	CSA	0 °C to –78 °C	Recovery of **25**
10[b]	LDA	THF	-	$(TMS)_2NH$	0 °C to –78 °C	Recovery of **25**
11[c]	LDA	THF	TMSCl	^{t}BuOH	0 °C to –78 °C	Recovery of **25**
12[c]	LDA	THF	TMSOTf	^{t}BuOH	0 °C to –78 °C	Recovery of **25**

[a]Base (5 equiv). [b]Base (5 equiv), proton source (10 equiv). [c]Base (5 equiv), additive (1.2 equiv), proton source (10 equiv).

1.2.35 Synthesis of Iodide 25b

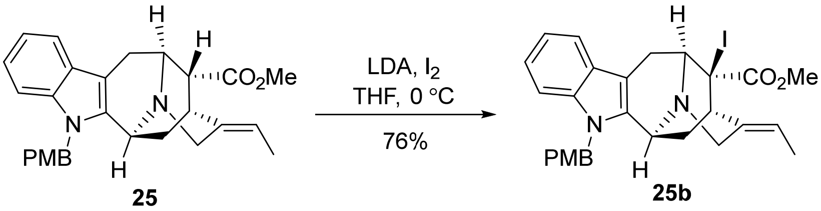

To a solution of ester **25** (180.0 mg, 0.41 mmol) in dry THF (15 mL) was added LDA (1.0 M, 2.0 mL, 2.0 mmol) via syringe at 0 °C under Ar. After being stirred at 0 °C for 10 min, a solution of I_2 (309.9 mg, 1.22 mmol) in anhydrous THF (20 mL) was added, and the resulting mixture was stirred at 0 °C for 30 min. The reaction was quenched with saturated *aq.* NH_4Cl (5 mL) and saturated *aq.* $Na_2S_2O_3$ (2 mL). The mixture was diluted with water (15 mL) and extracted with EtOAc (3 × 20 mL). The combined

organic phases were washed with brine (25 mL) and dried over anhydrous Na_2SO_4. After removal of the solvents under reduced pressure, the residue was purified by flash column chromatography on silica gel to afford iodide **25b** (175.3 mg, 76%, petroleum ether/EtOAc = 1:1.5) as white solids.

M.p.: 97-101 °C.

$\mathbf{R}_f$ = 0.62 (petroleum ether : ethyl acetate = 1:2).

$[\alpha]_D^{20}$ = –6.2 (*c* 0.43, $CHCl_3$).

FTIR (KBr, thin film) cm^{-1} : 2921, 1719, 1685, 1641, 1561, 1512, 1244, 1032, 739.

^{1}H NMR (400 MHz, $CDCl_3$): δ 7.54 (dd, *J* = 6.8, 1.6 Hz, 1H), 7.23 (d, *J* = 7.2 Hz, 1H), 7.15 (td, *J* = 6.8, 1.6 Hz, 1H), 7.13 (td, *J* = 6.8, 1.6 Hz, 1H), 6.99 (d, *J* = 8.6 Hz, 2H), 6.80 (d, *J* = 8.7 Hz, 2H), 5.41-5.36 (m, 1H), 5.27 (d, *J* = 16.8 Hz, 1H), 5.18 (d, *J* = 16.8 Hz, 1H), 4.03 (t, *J* = 6.6 Hz, 1H), 3.76 (s, 3H), 3.75 (s, 3H), 3.69-3.58 (m, 2H), 3.51 (d, *J* = 6.1 Hz, 1H), 3.50-3.43 (m, 1H), 3.09 (d, *J* = 16.0 Hz, 1H), 2.97 (t, *J* = 2.9 Hz, 1H), 2.04 (dd, *J* = 6.8, 2.8 Hz, 2H), 1.51 (d, *J* = 10.3 Hz, 3H).

^{13}C NMR (100 MHz, $CDCl_3$): δ 173.8, 159.0, 138.0, 137.5, 134.5, 129.8, 127.4, 127.1, 121.5, 120.4, 119.2, 118.7, 114.3, 109.5, 104.6, 57.0, 55.4, 53.9, 53.8, 53.1, 48.7, 46.3, 44.70, 33.9, 31.7, 13.0.

HRMS (ESI): Calcd for $C_{28}H_{30}IN_2O_3$ $[M+H]^+$: 569.1296, found: 569.1292.

1.2.36 Optimization for light induced radical reduction

Supplementary Table 3 Optimization for light induced radical reduction

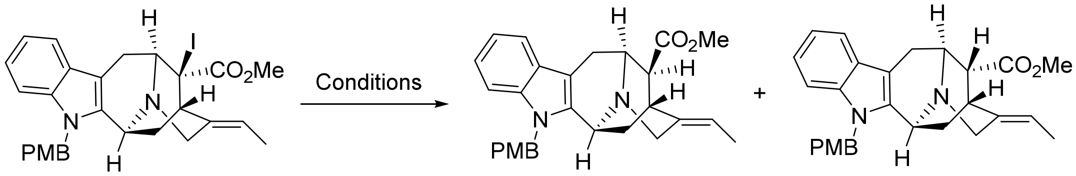

25b **25a** **25**

Entry	Conditions	Results[a]
1	Raney Ni, THF, rt	**25**: 72%
2	SmI_2 (10 equiv), MeOH (200 equiv), THF, Ar, 0 °C to rt	**25a**: 18%, **25**: 12%
3	Et_3B (0.5 equiv), $PhMe_2SiH$ (2 equiv), THF, air, –78 °C,	complex
4	Et_3B (0.2 equiv), Bn_3SnH (2 equiv), THF, air, –78 °C	**25**: 67%
5[b]	**P1**, DIPEA, $(TMS)_2NH$, MeCN, Blue LED, Ar, rt	**25a**: 3% **25**: 2%
6[b]	**P2**, DIPEA, $(TMS)_2NH$, MeCN, Blue LED, Ar, rt	**25a**: 3%, **25**: 2%
7[b]	**P4**, DIPEA, $(TMS)_2NH$, MeCN, Blue LED, Ar, rt	**25a**: 13%, **25**: 17%
8[b]	**P3**, DIPEA, $(TMS)_2NH$, MeCN, Blue LED, Ar, rt	**25a**: 21%, **25**: 20%
9[b]	**P3**, DIPEA, Hantzsch ester, DMSO, Blue LED, Ar, rt	**25a**: 12%, **25**: 24%
10[b]	**P3**, DIPEA, TTMSS, DMSO, Blue LED, Ar, rt	**25a**: 15%, **25**: 27%
11[b]	**P3**, DIPEA, TTMSS, DMF, Blue LED, Ar, –10 °C	**25a**: 22%, **25**: 22%
12[b]	**P3**, DIPEA, TTMSS, DMF, Blue LED, Ar, –40 °C	**25a**: 26%, **25**: 17%
13[b]	**P3**, DIPEA, TTMSS, DMF, Blue LED, Ar, –60 °C	**25a**: 28%, **25**: 16%
14[b]	**P3**, DIPEA, Ph_3SiH, DMF, Blue LED, Ar, –60 °C	**25a**: 18%, **25**: 18%
15[b]	**P3**, DIPEA, Ph_2SiH_2, DMF, Blue LED, Ar, –60 °C	**25a**: 19%, **25**: 18%
16[b]	**P3**, DIPEA, $(EtO)_3SiH$, DMF, Blue LED, Ar, –60 °C	**25a**: 21%, **25**: 20%
17[b]	**P3**, DIPEA, Et_3SiH, DMF, Blue LED, Ar, –60 °C	**25a**: 19%, **25**: 19%
18[b]	**P3**, DIPEA, $(TMS)_2NH$, DMF, Blue LED, Ar, –60 °C	**25a**: 47%, **25**: 16%
19[b]	**P3**, DIPEA, $(TMS)_2NH$, THF, Blue LED, Ar, –78 °C	No reaction
20[b]	**P3**, DIPEA, $(TMS)_2NH$, DCM, Blue LED, Ar, –78 °C	No reaction

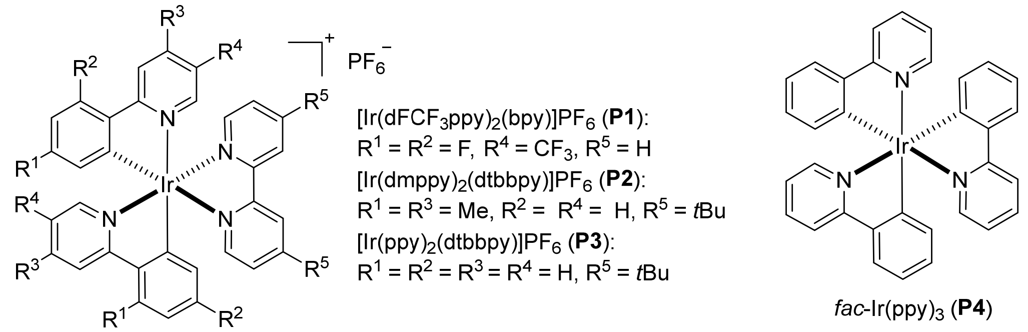

fac-Ir(ppy)3 (**P4**)

[a]Isolated yield after purification by silica gel chromatography. [b]Photocatalyst (5 mol %), DIPEA (5 equiv), hydrogen source (3 equiv), solvent, 24 W Bule LED, 1h.

1.2.37 Synthesis of Olefin 25a

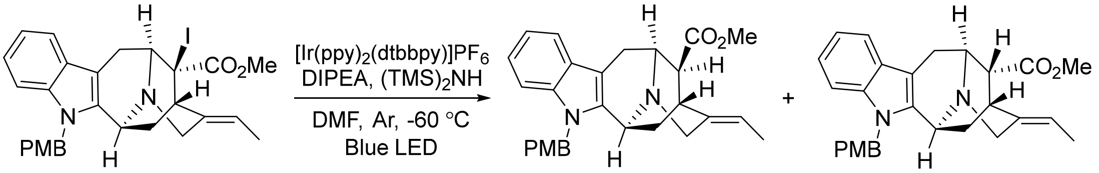

25b **25a**: 47% **25**: 16%

To a stirred solution of **25b** (96.0 mg, 0.17 mmol) in DMF (10 mL) were added DIPEA (147 μL, 0.85 mmol) and $(TMS)_2NH$ (106 μL, 0.51 mmol) at –60 ºC (refrigerant: EtOH) under Ar atmosphere, followed by addition of $[Ir(ppy)_2(dtbbpy)]PF_6$ (7.8 mg, 0.0084 mmol), and the resultant mixture was stirred under Blue LED (12 W × 2) for 1 h [*The ice formed on the outside surface of the flask must be removed with $HOCH_2CH_2OH/H_2O$ (2:1) every 10 min]. The reaction mixture was quenched with water (20 mL), and extracted with EtOAc (3 × 15 mL). The combined organic layers were washed with brine (15 mL), dried over Na_2SO_4, filtered, and concentrated under reduced pressure. The residue was purified by flash column chromatography on silica gel (petroleum ether/EtOAc) to recover ester **25** (11.8 mg, 16%) as a white solid. Futher elution provided ester **25a** (35.3 mg, 47%) as a colorless syrup.

25a:

$\mathbf{R}_f$ = 0.35 (petroleum ether : ethyl acetate = 1:1).

$[\alpha]_D^{20}$ = –41.3 (*c* 0.95, $CHCl_3$).

FTIR (KBr, thin film) cm^{-1} : 2930, 2837, 1732, 1614, 1513, 1466, 1348, 1247, 1201, 1176, 1037, 1013, 838, 821, 741.

^{1}**H NMR** (400 MHz, $CDCl_3$): δ 7.46 (d, *J* = 7.2 Hz, 1H), 7.16 (d, *J* = 7.6 Hz, 1H), 7.09 (t, *J* = 7.2 Hz, 1H), 7.05 (t, *J* = 7.2 Hz, 1H), 6.95 (d, *J* = 8.4 Hz, 2H), 6.79 (d, *J* = 8.0 Hz, 2H), 5.34-5.30 (m, 1H), 5.26 (d, *J* = 16.8 Hz, 1H), 5.08 (d, *J* = 16.8 Hz, 1H), 4.15 (d, *J* = 9.2 Hz, 1H), 3.82 (d, *J* = 17.6 Hz, 1H), 3.74 (s, 3H), 3.73-3.69 (m, 1H), 3.57 (d, *J* = 17.6 Hz, 1H), 3.27 (d, *J* = 15.6 Hz, 1H), 3.19 (s, 3H), 2.99 (dd, *J* = 15.6, 5.2 Hz, 1H), 2.94 (d, *J* = 11.6 Hz, 1H), 2.53 (dr d, *J* = 3.2 Hz, 1H), 1.73 (t, *J* = 11.2 Hz, 1H), 1.57 (d, *J* = 6.4 Hz, 3H).

^{13}**C NMR** (100 MHz, $CDCl_3$): δ 172.9, 158.9, 140.4, 138.9, 137.4, 129.8, 127.4, 127.1, 121.1, 119.1, 118.0, 115.1, 114.2, 109.6, 104.6, 55.3, 53.9, 53.2, 51.0, 49.3, 46.3, 44.7, 34.2, 27.9, 24.3, 12.6.

HRMS (ESI): Calcd for $C_{28}H_{31}N_2O_3$ $[M+H]^+$: 443.2329, found: 443.2331.

1.2.38 Synthesis of Ester S4

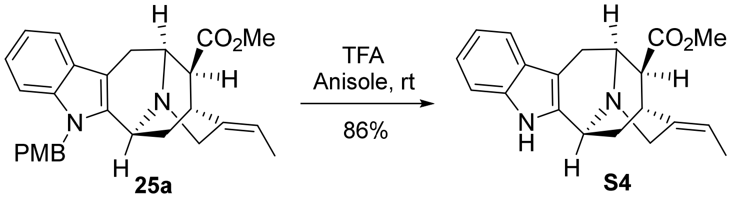

To compound **25a** (83.0 mg, 0.188 mmol) was added anisole (422 μL, 4.1 mmol) and cold TFA (2 mL). After being stirred at room temperature for 24 h, the reaction mixture was poured into an ice-cold saturated solution of sodium bicarbonate (30 mL). The resulting mixture was extracted with dichloromethane (3 × 15 mL). The combined organic phases were washed with brine (20 mL), dried over Na_2SO_4, filtered, and concentrated. The residue was purified by column chromatography on silica gel (CH_2Cl_2/MeOH = 20:1) to give ester **S4** (52.2 mg, 86%) as a white solid.

M.p.: 208-209 °C.

R*f* = 0.21 (ethyl acetate).

$[\alpha]_D^{20}$ = –25.0 (*c* 0.20, MeOH).

FTIR (KBr, thin film) cm^{-1} : 3404, 3142, 2945, 1715, 1460, 1449, 1435, 1308, 1216, 1166, 1019, 798, 733.

^{1}**H NMR** (400 MHz, $CDCl_3$): δ 8.14 (br s, 1H), 7.40 (d, *J* = 7.6 Hz, 1H), 7.22 (d, *J* = 8.0 Hz, 1H), 7.09 (td, *J* = 7.2, 0.8 Hz, 1H), 7.03 (td, *J* = 7.2, 0.8 Hz, 1H), 5.33-5.28 (m, 1H), 3.99 (d, *J* = 10.0 Hz, 1H), 3.75 (br d, *J* = 17.2 Hz, 1H), 3.66 (dd, *J* = 10.8, 4.0 Hz, 1H), 3.49 (d, *J* = 17.2 Hz, 1H), 3.22 (dd, *J* = 15.6, 2.0 Hz, 1H), 3.10 (s, 3H), 2.94-2.88 (m, 2H), 2.51-2.48 (m, 2H), 1.65-1.66 (m, 1H), 1.58 (d, *J* = 6.8 Hz, 3H).

^{13}**C NMR** (100 MHz, $CDCl_3$): δ 173.0, 140.6, 137.7, 136.7, 127.2, 121.3, 119.3, 117.9, 115.0, 111.0, 105.2, 53.6, 53.5, 51.0, 50.1, 44.6, 34.5, 28.3, 24.3, 12.6.

HRMS (ESI): Calcd for $C_{20}H_{23}N_2O_2$ $[M+H]^+$: 323.1754, found: 323.1749.

1.2.39 Synthesis of Koumidine (25c)

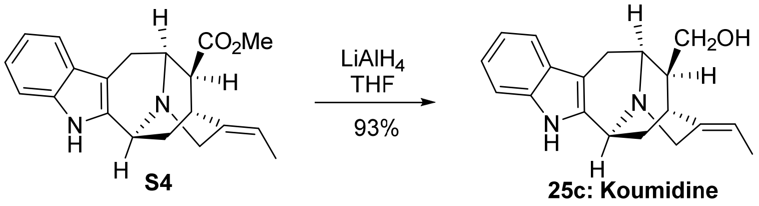

To a solution of compound **S4** (34.0 mg, 0.102 mmol) in dry THF (5 mL) was added $LiAlH_4$ (24.0 mg, 0.62 mmol) at 0 °C. The reaction mixture was stirred at room temperature under Ar for 4 h before being quenched with saturated *aq.* NH_4Cl (2 mL) and diluted with water (15 mL). The mixture was extracted with EtOAc (3 × 10 mL). The combined organic phases were washed with brine (10 mL), dried over Na_2SO_4, filtered, and concentrated. The residue was purified by column chromatography on silica gel (CH_2Cl_2/MeOH = 10:1) to yield Koumidine (**25c**, 28.3 mg, 93%) as a white solid.

M.p.: 154-156 °C.

$\mathbf{R}_f$ = 0.56 (CH_2Cl_2 : MeOH = 10:1).

$[\alpha]_D^{20}$ = –12.8 (*c* 0.80, MeOH). Lit[13]: $[\alpha]_D$ = –11 (*c* 0.07, MeOH); Lit[14]: $[\alpha]_D^{25}$ = –10.29 (*c* 0.175, MeOH).

FTIR (KBr, thin film) cm^{-1} : 3398, 2918, 1625, 1452, 1383, 1311, 1170, 1030, 843, 741.

^{1}H NMR (600 MHz, MeOD): δ 7.35 (d, *J* = 7.2 Hz, 1H), 7.22 (d, *J* = 7.8 Hz, 1H), 7.00 (t, *J* = 7.8 Hz, 1H), 6.93 (t, *J* = 7.8 Hz, 1H), 5.35-5.31 (m, 1H), 4.14 (dd, *J* = 10.2, 1.8 Hz, 1H), 3.75 (d, *J* = 17.4 Hz, 1H), 3.62 (d, *J* = 17.4 Hz, 1H), 3.58 (dd, *J* = 12.0, 6.6 Hz, 1H), 3.45 (dd, *J* = 10.8, 6.6 Hz, 1H), 3.08 (dd, *J* = 10.8, 9.0 Hz, 1H), 2.98 (d, *J* = 16.2, Hz, 1H), 2.88 (dd, *J* = 16.2, 6.0 Hz, 1H), 2.41 (br s, 1H), 2.22-2.19 (m, 1H), 1.90-1.86 (m, 1H), 1.81-1.79 (m, 1H), 1.54 (d, *J* = 6.6 Hz, 3H).

^{13}C NMR (100 MHz, MeOD): δ 140.4, 138.3,137.2, 127.3, 122.4, 120.0, 118.8, 116.2, 112.1, 105.7, 61.0, 54.5, 54.4, 51.3, 43.9, 34.8, 29.1, 23.1, 12.6.

HRMS (ESI): Calcd for $C_{19}H_{23}N_2O$ $[M+H]^+$: 295.1805, found: 295.1800.

1.2.40 Synthesis of Amide 37

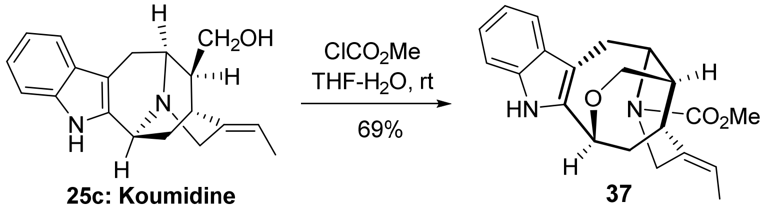

To a solution of **25c** (120.0 mg, 0.41 mmol) in THF (15 mL) and H_2O (3 mL) was added $ClCO_2Me$ (316 μL, 4.07 mmol) at 0 °C under argon atmosphere, and the reaction mixture was stirred at room temperature for 24 h. After addition of water (8 mL), the mixture was extracted with EtOAc (3 × 10 mL). The combined organic phases were washed with brine (10 mL), dried over Na_2SO_4, filtered, and concentrated. The residue was purified by column chromatography on silica gel (petroleum ether/EtOAc = 1:1.5) to yield amide (**37**, 99.5 mg, 69%) as a white solid.

M.p.: 146-148 °C.

R*f* = 0.65 (petroleum ether/EtOAc = 1:1.5).

$[\alpha]_D^{20}$ = –98.3 (*c* 1.36, $CHCl_3$).

FTIR (KBr, thin film) cm^{-1} : 2920, 1735, 1685, 1654, 1458, 1401, 1246, 1117, 738.

^{1}H NMR (400 MHz, $CDCl_3$): δ 8.13 (s, 0.5H), 8.08 (s, 0.5H), 7.62 (d, *J* = 7.8 Hz, 0.5H), 7.60 (d, *J* = 7.8 Hz, 0.5H), 7.30 (d, *J* = 7.6 Hz, 0.5H), 7.27 (d, *J* = 7.6 Hz, 0.5H), 7.21-7.09 (m, 2H), 5.44-5.37 (m, 1H), 5.17 (d, *J* = 9.6 Hz, 1H), 4.53 -4.48 (m, 1H), 4.41 (d, *J* = 15.8 Hz, 0.5H), 4.35-4.29 (m, 0.5H), 4.01-3.93 (m, 1H), 3.91-3.86 (m, 1H), 3.78 (s, 1.5H), 3.77 (s, 1.5H), 3.44-3.29 (m, 2H), 3.18 (dd, *J* = 10.1, 5.4 Hz, 0.5H), 3.14 (dd, *J* = 10.1, 5.4 Hz, 0.5H), 2.93-2.87 (m, 1H), 2.66-2.55 (m, 1H), 2.42-2.41 (m, 1H), 2.18 (d, *J* = 10.9 Hz, 0.5H), 2.14 (d, *J* = 9.2 Hz, 0.5H), 1.61 (d, *J* = 6.9 Hz, 1.5H), 1.57 (d, *J* = 6.8 Hz, 1.5H).

^{13}C NMR (100 MHz, $CDCl_3$): δ 155.9, 135.4, 134.7 (134.6), 132.6, 128.3 (128.2), 122.6 (122.5), 121.1 (120.5), 119.6, 118.7 (118.4), 111.1 (110.9), 110.7 (110.5), 67.7 (67.6), 61.8, 54.2 (53.8), 52.9 (52.8), 38.9 (38.6), 36.9 (36.8), 32.5 (32.3), 30.5 (30.4), 24.8 (24.2), 13.1 (13.0).

HRMS (ESI): Calcd for $C_{21}H_{25}N_2O_3$ $[M+H]^+$: 353.1860, found: 353.1859.

1.2.41 Synthesis of 19-*Z*-Taberpsychine (13)

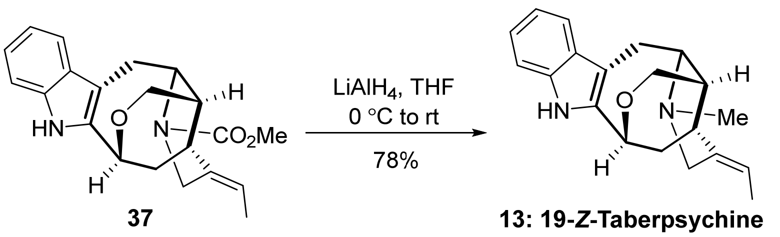

37 **13: 19-*Z*-Taberpsychine**

To a solution of compound **37** (24.6 mg, 0.070 mmol) in dry THF (5 mL) was added $LiAlH_4$ (40.0 mg, 1.05 mmol) at 0 °C. The reaction mixture was stirred at room temperature under Ar for 4 h before being quenched with saturated *aq.* NH_4Cl (2 mL) and diluted with water (15 mL). The mixture was extracted with CH_2Cl_2/MeOH (10:1, 3 × 10 mL). The combined organic phases were washed with brine (10 mL), dried over Na_2SO_4, filtered, and concentrated. The residue was purified by column chromatography on silica gel (CH_2Cl_2/MeOH = 7:1) to yield 19-*Z*-Taberpsychine (**13**, 16.8 mg, 78%) as a white solid.

M.p.: 128-129 °C.

R_f = 0.23 (CH_2Cl_2 : MeOH = 15:1).

$[\alpha]_D^{20}$ = –165.9 (*c* 0.58, $CHCl_3$). Lit[15]: **$[\alpha]_D^{20}$** = –151 (*c* 0.3, $CHCl_3$).

FTIR (KBr, thin film) cm^{-1} : 2923, 1735, 1685, 1654, 1630, 1618, 1459, 1383, 1088, 743.

^{1}H NMR (400 MHz, $CDCl_3$): δ 8.03 (br s, 1H), 7.62 (d, *J* = 7.6 Hz, 1H), 7.32 (d, *J* = 7.6 Hz, 1H), 7.19 (td, *J* = 7.2, 1.2 Hz, 1H), 7.14 (td, *J* = 8.0, 1.2 Hz, 1H), 5.44 (br q, *J* = 6.8 Hz, 1H), 5.13 (d, *J* = 9.2 Hz, 1H), 3.85 (dd, J = 11.2, 10.4 Hz, 1H), 3.42 (d, J = 14.8 Hz, 1H), 3.37-3.20 (m, 4H), 3.17-3.14 (m 1H), 2.83 (td, *J* = 9.6, 4.0 Hz, 1H), 2.61 (s, 3H), 2.58-2.57 (m, 1H), 2.43 (dt, *J* = 14.4, 9.6 Hz, 1H), 2.11 (dd, *J* = 14.0, 11.2 Hz, 1H), 1.60 (d, *J* = 6.8Hz, 3H).

^{13}C NMR (100 MHz, $CDCl_3$): δ 135.9, 135.5, 132.3, 128.4, 122.5, 120.5, 119.5, 118.3, 111.1, 110.8, 67.8, 62.0, 60.9, 46.2, 43.0, 37.4, 33.4, 30.7, 18.4, 12.9.

HRMS (ESI): Calcd for $C_{20}H_{25}N_2O$ $[M+H]^+$: 309.1961, found: 309.1960.

1.2.42 Synthesis of Iodide 38

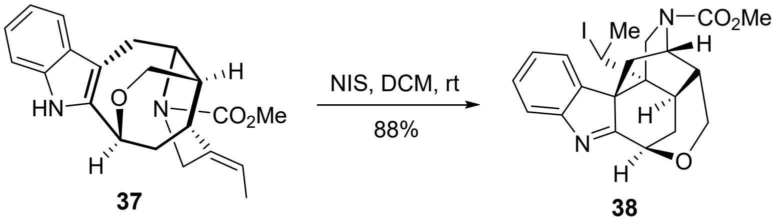

37 **38**

To a solution of compound **37** (35.0 mg, 0.10 mmol) in dry DCM (5 mL) was added NIS (26.8 mg, 0.12 mmol) at 0 °C. The reaction mixture was stirred at room temperature under Ar for 20 min before being quenched with saturated *aq.* $Na_2S_2O_3$ (0.3 mL) and diluted with water (5 mL). The mixture was extracted with CH_2Cl_2 (3 × 8 mL). The combined organic phases were washed with brine (10 mL), dried over Na_2SO_4, filtered, and concentrated. The residue was purified by column chromatography on silica gel (petroleum ether/EtOAc = 1:2) to yield iodide **38** (42.2 mg, 88%, white solid) as a single diastereoisomer.

M.p.: 97-99 °C.

$\mathbf{R}_f$ = 0.41 (petroleum ether/EtOAc = 1:2).

$[\alpha]_D^{20}$ = –216.0 (*c* 1.18, $CHCl_3$).

FTIR (KBr, thin film) cm^{-1} : 2924, 1735, 1686, 1654, 1450, 1401, 1113, 771.

^{1}H NMR (400 MHz, $CDCl_3$): δ 7.62 (d, *J* = 7.7 Hz, 1H), 7.47 (t, *J* = 7.6 Hz, 1H), 7.37 (t, *J* = 7.6 Hz, 1H), 7.24 (t, *J* = 7.6 Hz, 1H), 5.01 (s, 1H), 4.35 (s, 0.6H), 4.21 (s, 0.4H), 4.15 (d, *J* = 12.4 Hz, 1H), 3.91 (s, 0.4H), 3.88 (s, 0.6H), 3.82 (s, 1.8H), 3.78 (s, 1.2H), 3.78-3.74 (m, 0.4H), 3.69-3.59 (m, 1.6H), 3.53 (p, *J* = 6.6 Hz, 1H), 2.72-2.47 (m, 4H), 2.29 (d, *J* = 14.4 Hz, 1H), 1.85 (dd, *J* = 15.6, 6.4 Hz, 1H), 1.38 (d, *J* = 6.4 Hz, 1.2H), 1.36 (d, *J* = 6.4 Hz, 1.8H).

^{13}C NMR (100 MHz, $CDCl_3$): δ 184.4 (184.25), 155.5 (155.4), 154.23 (154.20), 143.4 (143.3), 129.15, 126.4 (126.5), 124.8 (124.9), 122.01 (121.97), 70.6 (70.7), 60.7 (60.8), 55.00 (55.03), 52.89, 49.0 (49.7), 47.33, 44.9 (45.0), 39.3 (39.7), 38.6, (38.5) 37.7 (37.2), 35.8 (35.9), 27.72 (27.66), 24.15 (24.18).

HRMS (ESI): Calcd for $C_{21}H_{24}IN_2O_3$ $[M+H]^+$: 479.0826, found: 479.0827.

1.2.43 Synthesis of Olefin 39

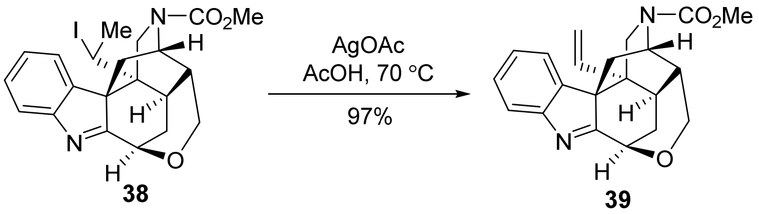

To a solution of compound **38** (14.8 mg, 0.031 mmol) in AcOH (1 mL) was added AgOAc (18.4 mg, 0.11 mmol) at room temperature under Ar. After being stirred at 70 °C for 3.5 h, the reaction mixture was poured into an ice-cold saturated solution of sodium bicarbonate (20 mL). The mixture was extracted with EtOAc(3 × 8 mL). The combined organic phases were washed with brine (10 mL), dried over Na_2SO_4, filtered, and concentrated. The residue was purified by column chromatography on silica gel (petroleum ether/EtOAc = 1:2) to yield olefin **39** (10.5 mg, 97%) as a white solid.

M.p.: 85-86 °C.

R*f* = 0.32 (petroleum ether/EtOAc = 1:2).

$[\alpha]_D^{20}$ = –235.7 (*c* 0.46, $CHCl_3$).

FTIR (KBr, thin film) cm^{-1} : 2923, 1735, 1701, 1686, 1654, 1450, 1401, 1122, 1077, 770.

^{1}H NMR (500 MHz, $CDCl_3$): δ 7.63 (d, *J* = 7.5 Hz, 1H), 7.41 (t, *J* = 8.5 Hz, 1H), 7.37 (t, *J* = 7.5 Hz, 1H), 7.26 (d, *J* = 8.5 Hz, 1H), 5.05 (s, 1H), 4.96-4.85 (m, 2H), 4.70 (dd, *J* = 17.5, 11.5 Hz, 1H), 4.38 (s, 0.6H), 4.23-4.20 (m, 1.4H), 3.85 (d, *J* = 12.0 Hz, 1H), 3.81 (s, 1.6H), 3.78 (s, 1.4H), 3.78-3.73 (m, 0.4H), 3.70-3.64 (m, 1.6H), 2.77-2.60 (m, 3H), 2.29-2.23 (m, 2H), 1.91 (d, *J* = 15.0 Hz, 1H).

^{13}C NMR (100 MHz, $CDCl_3$): δ 184.9 (184.7), 155.5 (155.4), 154.9 (154.8), 143.03 (142.98), 136.4 (136.1), 128.61, 126.4 (126.5), 123.0 (123.1), 121.40 (121.36), 116.6 (116.8), 70.9 (71.0), 60.8 (60.9), 57.24 (57.15), 52.79 (52.82), 49.46, 48.79 (48.83), 45.8 (45.7), 39.0 (39.4), 34.1 (34.0), 25.1.

HRMS (ESI): Calcd for $C_{21}H_{23}N_2O_3$ $[M+H]^+$: 351.1703, found: 351.1698.

1.2.44 Synthesis of Dihydrokoumine (40)

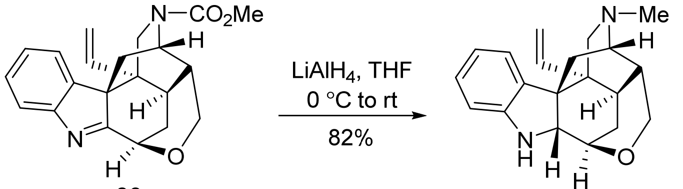

39 **40: Dihydrokoumine**

To a solution of compound **39** (15.0 mg, 0.0428 mmol) in anhydrous THF (4 mL) was added $LiAlH_4$ (25.4 mg, 0.668 mmol) at 0 °C. The reaction mixture was stirred at room temperature under Ar for 3 h before being quenched with saturated *aq.* NH_4Cl (0.2 mL) and *aq.* NaOH (3 M, 6 mL). The mixture was stirred at room temperature for 30 min, and then extracted with $CHCl_3$ (3 × 10 mL). The combined organic phases were washed with brine (10 mL), dried over Na_2SO_4, filtered, and concentrated. The residue was purified by column chromatography on silica gel (CH_2Cl_2/MeOH = 7:1) to yield dihydrokoumine (**40**, 10.8 mg, 82%) as a white solid.

M.p.: 128-132 °C.

$\mathbf{R}_f$ = 0.59 (CH_2Cl_2/MeOH = 7:1).

$[\alpha]_D^{20}$ = –26.0 (*c* 0.21, EtOH). Lit[16]: $[\alpha]_D^{20}$ = –30 (*c* 0.15, EtOH).

FTIR (KBr, thin film) cm^{-1} : 2921, 1685, 1630, 1610, 1481, 1465, 1249, 1219, 1085, 753.

^{1}H NMR (600 MHz, $CDCl_3$): δ 7.26 (d, *J* = 7.2 Hz, 1H), 7.11 (td, *J* = 7.2, 1.2 Hz, 1H), 6.85 (t, *J* = 7.2 Hz, 1H), 6.72 (d, *J* = 7.2 Hz, 1H), 5.50 (dd, *J* = 18.0, 11.4 Hz, 1H), 4.75 (d, *J* = 11.4 Hz, 1H), 4.68 (d, *J* = 18.0 Hz, 1H), 4.25 (d, *J* = 2.5 Hz, 1H), 4.17 (dd, *J* = 12.0, 4.8 Hz, 1H), 3.77 (br s, 1H), 3.63 (d, *J* = 12.0 Hz, 1H), 3.57 (br s, 1H), 2.74-2.72 (m, 2H), 2.65-2.63 (m, 3H), 2.54 (s, 3H), 2.31 (d, *J* = 12.0 Hz, 1H), 2.24-2.19 (m, 2H), 2.10 (dd, *J* = 15.0, 3.6 Hz, 1H).

^{13}C NMR (150 MHz, $CDCl_3D$): δ 151.6, 140.9, 134.1, 128.1, 123.6, 119.5, 113.7, 110.7, 75.6, 69.1, 61.9, 58.27, 58.21, 46.5, 42.3, 41.9, 38.5, 31.5, 29.8, 29.5, 20.2.

HRMS (ESI): Calcd for $C_{20}H_{25}N_2O$ $[M+H]^+$: 309.1961, found: 309.1956.

1.2.45 Synthesis of Koumine (14)

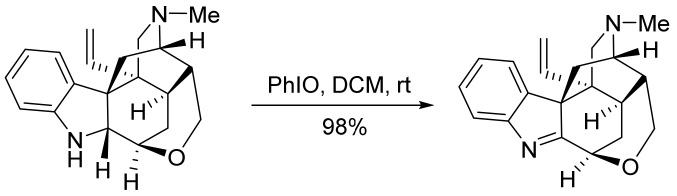

40: Dihydrokoumine **14: Koumine**

To a solution of dihydrokoumine **40** (8.0 mg, 0.026 mmol) in dry DCM (4 mL) was added PhIO (15.6 mg, 0.078 mmol) at 0 ºC. The reaction was stirred at room temperature for 1 h before being quenched with saturated *aq.* $Na_2S_2O_3$ (0.1 mL) and diluted with water (3 mL). The mixture was stirred at room temperature for 30 min, and then extracted with $CHCl_3$ (3 × 5 mL). The combined organic phases were washed with brine (10 mL), dried over Na_2SO_4, filtered, and concentrated. The residue was purified by column chromatography on silica gel (CH_2Cl_2/MeOH = 7:1) to yield Koumine (**40**, 7.8 mg, 98%) as a white solid.

M.p.: 152-153 ºC.

$\mathbf{R}_f$ = 0.57 (CH_2Cl_2/MeOH = 7:1).

$[\alpha]_D^{20}$ = –222.5 (*c* 0.19, MeOH). Lit[17]: $[\alpha]_D^{25}$ = –232.7 (*c* 0.1, MeOH).

FTIR (KBr, thin film) cm^{-1} : 2920, 2852, 1654, 1630, 1492, 1444, 1080, 776.

^{1}H NMR (600 MHz, $CDCl_3$): δ 7.61 (d, *J* = 7.8 Hz, 1H), 7.54 (d, *J* = 7.2 Hz, 1H), 7.35 (td, *J* = 7.8, 1.2 Hz, 1H), 7.25 (td, *J* = 7.2, 1.2 Hz, 1H), 5.02 (br s, 1H), 4.83 (d, *J* = 18.0 Hz, 1H), 4.79 (d, *J* = 10.8 Hz, 1H), 4.68 (dd, *J* = 18.0, 10.8 Hz, 1H), 4.26 (dd, *J* = 12.0, 4.2Hz, 1H), 3.62 (d, *J* = 11.4 Hz, 1H), 3.18 (d, *J* = 11.4 Hz, 1H), 3.13 (d, *J* = 11.4 Hz, 1H), 2.83 (br s, 2H), 2.63 (s, 3H), 2.62–2.59 (m, 1H), 2.42–2.34 (m, 3H), 1.88 (d, *J* = 15.0 Hz, 1H).

^{13}C NMR (150 MHz, MeOD): δ 185.5, 155.1, 143.7, 137.3, 128.4, 126.2, 123.2, 121.3, 116.1, 71.1, 61.4, 58.1, 57.8, 57.2, 45.4, 42.7, 38.9, 33.2, 28.7, 25.4.

HRMS (ESI): Calcd for $C_{20}H_{23}N_2O$ $[M+H]^+$: 307.1805, found: 307.1800.

1.3 Spectral Data Comparison of Natural or/and Synthetic Products with Our Synthetic Products

1.3.1 Akuammidine (1)

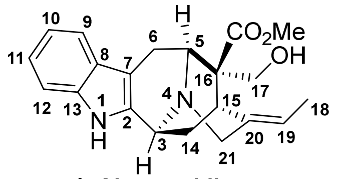

1: **Akuammidine**

Supplementary Table 4 Comparison of NMR spectra of Akuammidine (**1**)

Position	Natural<br>Riley & Rijn[18], **2021**<br>(400 MHz, MeOD)		This work<br>(400 MHz, MeOD)	
	δ_H mult (J in Hz)	δ_C	δ_H mult (J in Hz)	δ_C
2		137.1		137.08*
3	4.25 d (9.7)	50.6	4.22 d (9.6)	50.5
5	3.27 d (3.2)	57.6	3.25 d (3.2)	57.6
6α	3.44 (16.8, 3.3)	23.6	3.41 dd (18.4, 4.4)	23.6
6β	2.88-2.84 m		2.83 dd (18.4, 4.4)	
7		104.7		104.7
8		126.5		126.5
9	7.39 d (7.7)	118.3	7.37 d (7.6)	118.3**
10	6.98 t (7.4)	117.2	6.96 td (7.6, 0.8)	117.2**
11	7.06 t (7.4)	120.7	7.04 td (7.6, 0.8)	120.7
12	7.29 d (8.0)	110.6	7.27 d (8.0)	110.5
13		137.1		137.05*
14α	2.72 ddd (13.1, 4.2, 1.6)	28.7	2.70 ddd (12.8, 4.4, 2.0)	28.7
14β	1.92 t (11.6)		1.89 ddd (12.8, 10.4, 2.0)	
15	2.88-2.84 m	28.9	2.81 (s, 1H)	28.9
16		51.3		51.2
17α	3.80 d (9.7)	67.6	3.77 d (9.6)	67.5
17β	3.68-3.63 m		3.65 d (9.6)	
18	1.71 d (6.8)	11.9	1.69 dt (6.8, 2.0).	11.9
19	5.47 q (6.7)	116.9	5.44 q (6.8)	116.8**
20		136.4		136.4*
21α	3.68-3.63 m	54.7	3.63 dt (16.8, 2.4)	54.7
21β	3.54 d (16.8)		3.51 d (16.8)	
$CO_2\underline{Me}$	2.96 s	50.2	2.94 s	50.2
$\underline{C}O_2Me$		173.3		173.3

*, ** Assigments for these signals within a vertical column may be reversed.

1.3.2 19-(Z)-Akuammidine (4)

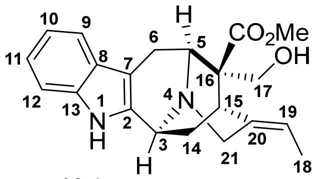

4: 19-(*Z*)-akuammidine

Supplementary Table 5 Comparison of NMR spectra of 19-(Z)-Akuammidine (**4**)

Position	Natural Ponglux[4], **1988** (270 MHz, MeOD)		This work (400 MHz, MeOD)	
	δH mult (*J* in Hz)	δC	δH mult (*J* in Hz)	δC
2		139.1*		139.1*
3	4.20 d (8.3)	51.5	4.20 d (10.0)	51.9
5	nr	59.5	2.76 d (2.4)	59.5
6α	nr	25.1	3.42 dd (15.6, 1.2)	25.2
6β	nr		2.85 dd (15.6, 4.4)	
7		106.0		106.4
8		128.1		128.2
9	nr	119.8**	7.37 d (7.6)	120.0**
10	nr	118.6**	6.97 td (7.2, 0.8)	118.8**
11	nr	122.1	7.04 td (7.2, 0.8)	122.3
12	nr	112.0	7.27 d (8.0)	112.2
13		138.9*		138.9*
14α	nr	31.4	2.68 dd (13.2, 1.6)	31.4
14β	nr		1.93 ddd (12.0, 10.0, 1.2)	
15	2.75 d (4.1, 1.3)	37.4	2.78 d (4.0)	37.2
16		53.2		53.2
17α	3.74 d (10.2)	69.1	3.74 d (10.4)	69.1
17β	3.69 d (10.2)		3.70 d (10.4)	
18	1.62 br d (6.9)	12.5	1.62 d (6.8)	12.8
19	5.44 m	118.2**	5.47-5.43 m	118.6**
20		138.6*		138.7*
21α	3.63 d (13.5)	54.0	3.64 d (17.2)	54.1
21β	3.57 d (13.5)		3.58 d (17.2)	
CO_2<u>Me</u>	2.96 s	51.5	2.97 s	51.8
<u>C</u>O_2Me		174.9		174.9

*, ** Assigments for these signals within a vertical column may be reversed.
nr = no report for these protons

1.3.3 Polyneuridine (2)

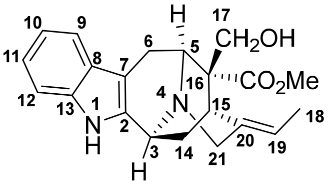

2: **Polyneuridine**

Supplementary Table 6 Comparison of ^{1}H NMR spectra of Polyneuridine (**2**)

Position	Natural Lounasmaa[19], **1996** (400 MHz, $CDCl_3$) δ_H mult (*J* in Hz)	Synthetic Cook[20], **2010** (300 MHz, $CDCl_3$) δ_H mult (*J* in Hz)	This work (400 MHz, $CDCl_3$) δ_H mult (*J* in Hz)
1	7.81 br s	8.48 s	7.86 br s
3	4.06 dd (10.5, 4)	4.18 d (9)	4.03 dd (10.0, 4.0)
5	4.27 br d (6.5)	4.35 d (6.2)	4.27 d (6.4 Hz, 1H)
6α	3.10 dd (16.5, 6.5)	3.12 dd (6.3)	3.09 dd (16.4, 6.4)
6β	2.94 br d (16.5)	3.01 br d (nr)	2.94 br d (16.4)
9	7.48 d (nr)	7.48 d (7.5)	7.48 d (7.6)
10	7.10 t (nr)	7.10 t (nr)	7.10 t (7.2, 0.8)
11	7.15 t (nr)	7.16 t (nr)	7.15 td (7.2, 0.8)
12	7.31 d (nr)	7.34 d (7.8)	7.30 d (8.0)
14α	1.91 ddd (13.5, 4, 3.5)	1.95 ddd (nr)	1.89 ddd (13.2, 9.6, 2.4)
14β	1.85 ddd (13.5, 10.5, 2.5)	1.85 ddd (nr)	1.84 ddd (13.2, 3.6, 3.2)
15	3.21 dd (3.5, 2.5)	3.18 (nr)	3.19 dd (3.5, 2.4)
17α	3.71 d (11.5)	3.65 d (8.2)	3.70 d (11.6)
17β	3.61 d (11.5)	3.50 m	3.60 d (11.2)
18	1.60 br d (6.5)	1.59 d (6.8)	1.60 d (6.8).
19	5.28 br q (6.5)	5.23 br q (6.8)	5.27 br q (6.8)
21α	3.60 m	3.57 m	3.64-3.55 m
21β	3.60 m	3.57 m	3.64-3.55 m
CO_2<u>Me</u>	3.73 s	3.73 s	3.72 s

nr = no report for these *J* values

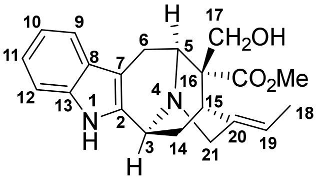

2: **Polyneuridine**

Supplementary Table 7 Comparison of ^{13}C NMR spectra of Polyneuridine (**2**)

Position	Natural<br>Lounasmaa[19], **1996**<br>(100 MHz, $CDCl_3$)<br>δ_C	Synthetic<br>Cook[20], **2010**<br>(125 MHz, $CDCl_3$)<br>δ_C	This work<br>(100 MHz, $CDCl_3$)<br>δ_C
2	136.2*	134.5*	136.2*
3	49.0	48.8	49.0
5	53.6	53.6	53.6
6	22.3	21.9	22.3
7	106.2	105.6	106.2
8	126.5	126.1	126.5
9	118.3	118.2	118.3
10	119.5	119.4	119.5
11	121.6	121.7	121.6
12	110.8	111.1	110.9
13	136.5*	135.8*	136.5*
14	28.9	28.6	28.9
15	30.6	30.4	30.6
16	53.4	53.3	53.4
17	63.2	63.1	63.2
18	12.7	12.6	12.7
19	116.0	116.7	116.0
20	136.9*	136.1*	136.9*
21	55.8	55.1	55.7
$CO_2\underline{Me}$	52.2	52.2	52.2
$\underline{C}O_2Me$	176.4	175.7	176.4

* Assigments for these signals within a vertical column may be reversed.

1.3.4 Vincarine (8)

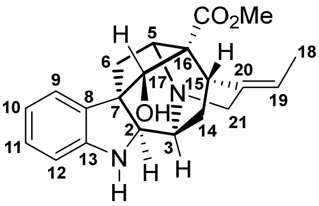

8: **Vincarine**

Supplementary Table 8 Comparison of NMR spectra of Vincarine (**8**)

Position	Natural<br>Yuldashev & Yunusov[6], **1965**		This work<br>(400 MHz, CDCl3)	
	δH mult (*J* in Hz)	δC	δH mult (*J* in Hz)	δC
2	nr		4.00 d (5.2)	70.1
3	nr		3.60 dd (9.6, 4.8)	55.3
5	nr		3.48 d (4.4)	61.7
6α	nr		1.74 d (12.0)	36.4
6β	nr		2.24 dd (12.0, 4.8)	
7				54.3
8				130.1
9	7.25-6.52		7.06 d (6.8)	122.3
10	7.25-6.52		6.84 t (7.6)	120.6
11	7.25-6.52		7.12 td (7.6, 0.8)	128.6
12	7.25-6.52		6.82 d (7.6)	111.8
13				152.1
14α	nr	nr	1.50 dd (13.6, 9.6)	23.7
14β	nr		3.06 dd (13.6, 5.2)	
15	nr		3.42 d (4.8)	30.3
16				54.8
17	nr		3.98 s (1H)	86.5
18	1.55 dt		1.58 d (6.8)	13.1
19	5.10 q		5.23 q (6.8)	116.0
20				137.0
21α	nr		3.45 br s (2H)	55.7
21β	nr		3.45 br s (2H)	
$CO_2\underline{Me}$	nr		3.68 s (3H)	52.1
$\underline{C}O_2Me$				175.5
OH			2.75 br s (1H)	

nr = no report for these protons or carbons

1.3.5 Vincamedine (7)

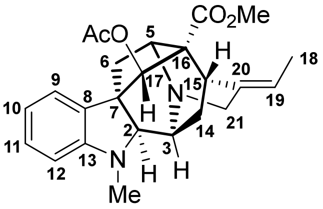

7: **Vincamedine**

Supplementary Table 9 Comparison of NMR spectra of Vincamedine (**7**)

Position	Natural Lounasmaa[21], **1996** (400 MHz, $CDCl_3$)		This work (400 MHz, $CDCl_3$)	
	δ_H mult (*J* in Hz)	δ_C	δ_H mult (*J* in Hz)	δ_C
2	3.21 d (5)	74.7	3.21 d (4.8)	74.8
3	3.57 ddd (9, 5, 1)	53.1	3.56 dd (9.6, 4.8)	53.2
5	3.62 dd (5, 1)	61.5	3.61 d (4.4)	61.6
6α	1.78 dd (12, 1)	36.4	1.78 d (11.2)	36.5
6β	2.55 dd (12, 5)		2.55 dd (11.6, 4.8)	
7		56.1		56.1
8		128.9		128.9
9	6.98 d (nr)	123.3	6.98 d (6.8)	123.3
10	6.72 t (nr)	118.9	6.72 t (7.6)	118.9
11	7.16 t (nr)	128.6	7.17 t (7.6)	128.6
12	6.66 d (nr)	109.3	6.66 d (8.0)	109.3
13		154.3		154.3
14α	1.55 ddd (14, 9, 1-2)	21.7	1.52 d (10.0)	21.8
14β	2.63 ddd (14, 5, 1)		2.62 dd (10.0, 4.8)	
15	3.50 dd (5, 1-2)	30.3	3.50 d (4.8)	30.4
16		58.9		58.9
17	5.67 s	75.5	5.67 s	75.5
18	1.56 dd (7, 2)	12.7	1.55 d (7.6)	12.7
19	5.28 q (7)	116.8	5.28 q (6.8)	116.8
20		136.4		136.6
21α	3.5 def	55.4	3.50-3.41 m	55.5
21β	3.5 def		3.50-3.41 m	
N-<u>Me</u>	2.65 s	34.1	2.65 s	34.1
CO_2<u>Me</u>	3.65 s	51.6	3.66 s	51.6
<u>C</u>O_2Me		172.2		172.3
O-CO<u>Me</u>	1.87 s	20.7	1.87 s	20.7
O-<u>CO</u>Me		168.4		168.4

nr = no report for these *J* values

1.3.6 Vincamajine (6)

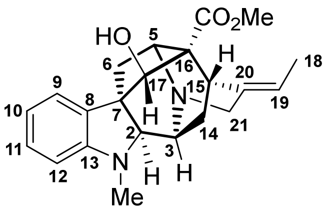

6: **Vincamajine**

Supplementary Table 10 Comparison of NMR spectra of Vincamajine (**6**)

Position	Natural Lounasmaa[21], **1996** (400 MHz, $CDCl_3$)		This work (400 MHz, $CDCl_3$)	
	δ_H mult (*J* in Hz)	δ_C	δ_H mult (*J* in Hz)	δ_C
2	3.22 d (5)	74.7	3.21 d (5.2)	75.0
3	3.52 ddd (10, 5, 1)	53.2	3.50-3.47 m	53.1
5	3.55 dd (5, 1)	61.7	3.53 d (4.8)	61.7
6α	1.71 dd (12, 1)	35.4	1.68 d (11.6)	35.6
6β	2.61 dd (12, 5)		2.61 dd (11.6, 4.8)	
7		57.1		57.1
8		129.9		130.0
9	7.16 d (nr)	124.2	7.15 d (6.8)	124.1
10	6.79 t (nr)	119.2	6.79 t (7.2)	119.1
11	7.18 t (nr)	128.5	7.18 td (7.6, 0.8)	128.5
12	6.65 d (nr)	109.1	6.65 d (8.0)	109.1
13		154.5		154.5
14α	1.50 ddd (13.5, 10, 1)	21.9	1.49 dd (13.6, 10.0)	22.0
14β	2.44 ddd (13.5, 5, 1)		2.43 dd (14.0, 4.8)	
15	3.48 dd (5, 1)	30.1	3.50-3.47 m	30.2
16		59.5		59.5
17	4.23 s	74.7	4.24 s	74.8
18	1.59 br d (7)	12.8	1.59 br d (6.8)	12.8
19	5.26 q (7)	116.7	5.25 q (6.8)	116.4
20		136.5		136.9
21α	3.4 def	55.4	3.42-3.40 m	55.5
21β	3.4 def		3.42-3.40 m	
N-Me	2.63 s	34.3	2.64 s	34.3
CO_2Me	3.69 s	51.6	3.69 s	51.6
CO_2Me		173.0		173.1
OH			1.99 br s	

nr = no report for these *J* values

1.3.7 Quebrachidine (5)

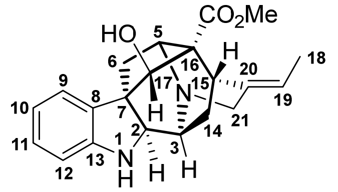

5: Quebrachidine

Supplementary Table 11 Comparison of NMR spectra of Quebrachidine (**5**)

Position	Natural Lounasmaa[22], **1985** (400 MHz, $CDCl_3$)		This work (400 MHz, $CDCl_3$)	
	δH mult (*J* in Hz)	δC	δH mult (*J* in Hz)	δC
2	3.67 d (5)		3.85 d (4.8)	74.5
3	3.30 ddd (10, 5, 1)		3.50-3.45 m	54.8
5	3.44 dd (5, 1)		3.55 d (4.4)	61.8
6α	1.65 dd (12, 1)		1.74 d (11.6)	35.7
6β	2.55 dd (12, 5)		2.62 dd (12.0, 4.8)	
7				57.9
8				129.6
9	7.16		7.17 d (7.2)	124.9
10	6.74		6.80 t (7.6)	119.8
11	7.05		7.11 td (7.6, 1.2)	128.5
12	6.72		6.77 d (7.6)	111.1
13				151.8
14α	1.42 ddd (14, 10, 2)	nr	1.50 dd (13.6, 10.0)	22.4
14β	2.49 ddd (14, 5,1)		2.56 dd (14.0, 4.8)	
15	3.43 dd (5, 2)		3.50-3.45 m	30.5
16				59.6
17	4.18 s		4.27 s	68.5
18	1.58 br d (7)		1.59 d (6.8)	13.0
19	5.22 br q (7)		5.25 q (6.8)	116.9
20				136.6
21α	3.23 def		3.41-3.36 m	55.3
21β	3.25 def		3.41-3.36 m	
$CO_2\underline{Me}$	3.63 s		3.69 s	51.8
$\underline{C}O_2Me$				173.1
OH			2.13 br s	

nr = no report for these carbons

1.3.8 Vincamajinine (9)

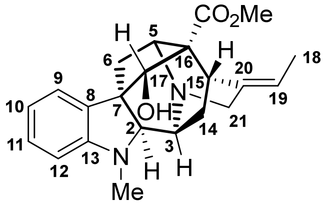

9: **Vincamajinine**

Supplementary Table 12 Comparison of ^{1}H NMR spectra of Vincamajinine (**9**)

Position	Natural Zhukovich[23], **1985** ($CDCl_3$) δ_H mult (*J* in Hz)	Synthetic Cook[11], **2016** (300 MHz, $CDCl_3$) δ_H mult (*J* in Hz)	This work (400 MHz, $CDCl_3$) δ_H mult (*J* in Hz)
2	nr	3.37 d (4.9)	3.37 d (5.2)
3	nr	3.63 dd (9.2, 4.9)	3.64 dd (9.6, 4.8)
5	nr	3.41 d (4.7)	3.43 d (4.4)
6α	nr	1.74 d (11.9)	1.74 d (11.6)
6β	nr	2.54 dd (11.9, 4.7)	2.26 dd (12.0, 4.8)
9	nr	7.04 dd (7.4, 0.8)	7.07 d (7.2)
10	nr	6.83 td (7.4, 0.8)	6.85 t (7.6)
11	nr	7.18 td (7.7, 1.3)	7.20 t (7.6)
12	nr	6.76 d (7.9)	6.74 d (8.0)
14α	nr	1.50 dd (13.7, 10.2)	1.51 dd (13.6, 9.6)
14β	nr	2.90 dd (13.4, 4.9)	2.91 dd (13.6, 5.2)
15	nr	3.48 m	3.50-3.48 m
17	nr	3.98 s	4.00 s
18	1.62 dt	1.56 dt (6.8, 1.6)	1.59 d (6.8)
19	5.31 q	5.23 q (6.7)	5.24 q (6.8)
21α	nr	3.48 m	3.50-3.48 m
21β	nr	3.48 m	3.50-3.48 m
N-<u>Me</u>	2.64 s	2.63 s	2.64 s
CO_2<u>Me</u>	3.69 s	3.68 s	3.69 s
OH	nr		2.64 br s

nr = no report for these protons

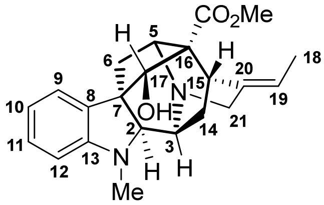

9: **Vincamajinine**

Supplementary Table 13 Comparison of ^{13}C NMR spectra of Vincamajinine (**9**)

Position	Natural<br>Zhukovich[23], **1985**<br>($CDCl_3$)<br>δ_C	Synthetic<br>Cook[11], **2010**<br>(125 MHz, $CDCl_3$)<br>δ_C	This work<br>(100 MHz, $CDCl_3$)<br>δ_C
2	nr	76.2	76.3
3		53.6	53.5
5		61.7	61.7
6		36.1	36.1
7		54.1	54.1
8		130.3	130.4
9		121.6	121.6
10		120.1	120.0
11		128.6	128.5
12		109.8	109.8
13		154.7	154.7
14		23.1	23.1
15		29.9	29.9
16		53.8	53.8
17		86.3	86.2
18		12.9	12.9
19		115.9	115.8
20		136.7	136.9
21		55.6	55.6
N-<u>Me</u>		34.9	34.9
CO_2<u>Me</u>		51.9	51.9
<u>C</u>O_2Me		175.3	175.4

nr = no report for these carbons

1.3.9 Alstiphyllanine J (11)·TFA

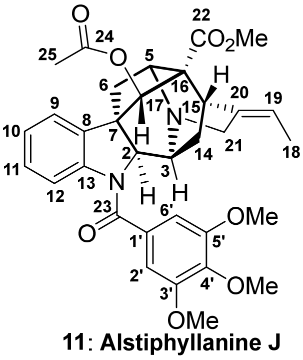

11: Alstiphyllanine J

Supplementary Table 14 Comparison of NMR spectra of Alstiphyllanine J (**11**)·TFA

Position	Natural Morita[12], **2012** (400 MHz, MeOD)		This work (400 MHz, MeOD)	
	δH mult (*J* in Hz)	δC	δH mult (*J* in Hz)	δC
2	4.44 m	68.3	4.37 d (5.2)	68.3
3	5.19 br s	57.1	5.20 dd (10.0, 4.8)	57.2
5	4.44 m	64.8	4.46 d (4.0)	64.8
6α	2.31 d (9.5)	34.2	2.20 d (13.2)	34.2
6β	2.86 d (9.5)		2.86 dd (13.6, 4.4)	
7		57.0		57.1
8		129.6		129.6
9	7.23 br s	125.1	7.23 dd (7.6, 1.6)	125.2
10	7.01 m	125.0	7.05-6.96 m	125.1
11	7.01 m	129.7	7.05-6.96 m	129.8
12	6.23 m	117.1	6.21 d (7.2)	117.2
13		145.3		145.3
14α	2.07 t (14.0)	22.6	2.07 dd (14.8, 10.4)	22.5
14β	2.59 d (14.0)		2.58 dd (15.2, 5.2),	
15	3.37 br s	36.7	3.39 d (4.8)	36.6
16		60.2		60.1
17	6.02 (s)	74.9	6.03 s (1H)	74.8
18	1.61 d (4.5)	12.8	1.60 d (6.8)	12.9
19	5.59 m	123.8	5.59-5.58 m	123.9
20		128.7		128.6
21α	4.08 m	52.3	4.37 d (15.2)	52.2
21β	4.35 d (13.8)		4.12 d (15.2)	
22		170.8		170.8
23		170.5		170.5
24		169.8		169.7
25	1.88 s	20.4	1.88 s	20.5
CO_2<u>Me</u>	3.78 s	53.4	3.76 br s	53.5

3'-O-Me	3.85 s	61.3	3.83 br s	61.3
4'-O-Me	3.78 s	57.0	3.76 br s	56.9
5'-O-Me	3.85 s	61.3	3.83 br s	61.3
1'		130.5		130.5
2'	7.05 s	107.8	7.05-6.96 m	107.5
3'		155.0		155.1
4'		143.4		143.1
5'		155.0		155.1
6'	7.05 s	107.8	7.05-6.96 m	107.5

1.3.10 Koumidine (25c)

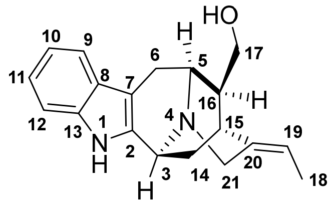

7: Koumidine

Supplementary Table 15 Comparison of NMR spectra of Koumidine (**25c**)

Position	Synthetic Cook[14], **2003** (400 MHz, MeOD)		This work (600 MHz, MeOD)	
	δH mult (*J* in Hz)	δC	δH mult (*J* in Hz)	δC
2		137.27		137.2
3	4.19-4.16 dd (10.1, 3.0)	51.31	4.14 dd (10.2, 1.8)	51.3
5	3.65-3.61 dd (10.8, 6.6)	54.54	3.58 dd (12.0, 6.6)	54.5
6α	2.97 t (16.0)	23.03	2.98 d (16.2)	23.1
6β	2.42 d (2.5)		2.88 dd (16.2, 6.0)	
7		105.7		105.7
8		127.33		127.3
9	7.36-7.33 d (7.7)	118.8	7.35 d (7.2)	118.8
10	7.02-6.90 m	119.97	6.93 t (7.8)	120.0
11	7.02-6.90 m	122.4	7.00 t (7.8)	122.4
12	7.23–7.20 d (7.8)	112.04	7.22 d (7.8)	112.1
13		138.26		138.3
14α	1.92-1.85 m	29.09	1.90-1.86 m	29.1
14β	1.82-1.76 m		1.81-1.79 m	
15	2.93-2.85 dd (15.8, 5.7)	34.83	2.41 br s (1H)	34.8
16	2.20 m	43.90	2.22-2.19 m	43.9
17α	3.46-3.41 dd (10.8, 6.6)	61.01	3.45 dd (10.8, 6.6)	61.0
17β	3.15-3.05 dd (8.7, 2.0)		3.08 dd (10.8, 9.0)	
18	1.54 d (6.8)	12.57	1.54 d (6.6)	12.6
19	5.35-5.33 m	116.20	5.35-5.31 m	116.2
20		140.50		140.4
21α	3.83-3.78 d (16.2)	54.45	3.75 d (17.4)	54.4
21β	3.68-3.63 d (16.5)		3.62 d (17.4)	

1.3.11 19-*Z*-Taberpsychine (13)

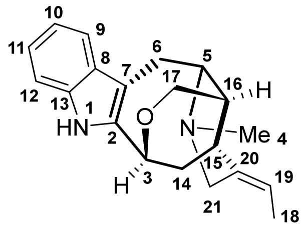

13: 19-*Z*-Taberpsychine

Supplementary Table 16 Comparison of ^{1}H NMR spectra of 19-*Z*-Taberpsychine (**13**)

Position	Natural Ponglux[4], **1988** (270 MHz, $CDCl_3$) δ_H mult (*J* in Hz)	Synthetic Kerr[24], **2018** (600 MHz, $CDCl_3$) δ_H mult (*J* in Hz)	This work (400 MHz, $CDCl_3$) δ_H mult (*J* in Hz)
1	7.98 br s	7.98 br s	8.03 br s
3	5.12 dd (9.8, 1.2)	5.12 dd (10.1, 1.5)	5.13 d (9.2)
5	3.15 m	3.10 ddd (10.7, 7.1, 4.2)	3.17-3.14 m
6α	nr	3.31 dd (15.4, 7.2)	3.37-3.20 m
6β	nr	3.22 dd (15.4, 10.1)	3.37-3.20 m
9	nr	7.63 dd (7.9, 1.0)	7.62 d (7.6)
10	nr	7.14 ddd (8.0, 7.0, 1.1)	7.14 td, (8.0, 1.2)
11	nr	7.19 ddd (8.1, 7.0, 1.2)	7.19 td (7.2, 1.2)
12	nr	7.32 dt (8.1, 0.9)	7.32 d (7.6)
14α	2.43 dt (14.3, 9.8)	2.43 dt (14.4, 9.7)	2.43 dt (14.4, 9.6)
14β	2.12 ddd (14.2, 10.7, 1.2)	2.12 ddd (14.4, 10.7, 1.4)	2.11 dd (14.0, 11.2)
15	2.82 br td (10.0, 6.0)	2.83 td (10.0, 5.5)	2.83 td (9.6, 4.0)
16	nr	2.54 dt (9.2, 4.4)	2.58-2.57 m
17α	3.84 dd (11.6, 10.1)	3.84 dd (11.5, 10.3)	3.85 dd (11.2, 10.4)
17β	3.26 d (11.6)	3.26 dd (11.5, 2.1)	3.37-3.20 m
18	1.61 br d (6.7)	1.61 dd (6.7, 1.4)	1.60 d (6.8)
19	5.43 br q (6.7)	5.42 br q (6.8)	5.44 br q (6.8)
21α	nr	3.39 d (14.9)	3.42 d (14.8)
21β	nr	3.34 d (14.9)	3.37-3.20 m
N-Me	2.59 s	2.59 s	2.61 s

nr = no report for these protons

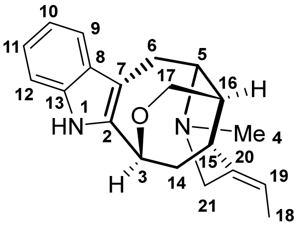

13: 19-*Z*-Taberpsychine

Supplementary Table 17 Comparison of ^{13}C NMR spectra of 19-*Z*-Taberpsychine (**13**)

Position	Natural Ponglux[4], **1988** (50 MHz, CDCl3) δC	Synthetic Kerr[24], **2018** (150 MHz, CDCl3) δC	This work (100 MHz, CDCl3) δC
2	136.2*	136.4	135.9*
3	67.6	67.8	67.8
5	60.5	60.7	60.9
6	18.0	18.2	18.4
7	110.9	111.1	110.8
8	128.3	128.5	128.4
9	119.8**	119.9	120.5**
10	119.3**	119.4	119.5**
11	122.3**	122.4	122.5**
12	110.9	111.2	111.1
13	135.3*	135.5	135.5*
14	29.7	30.6	30.7
15	33.5	33.7	33.4
16	37.5	37.7	37.4
17	61.9	62.1	62.0
18	12.8	12.9	12.9
19	119.2	118.3	118.3
20	131.9	132.2	132.3
21	45.9	46.1	46.2
N-Me	43.0	43.2	43.0

*, ** Assigments for these signals within a vertical column may be reversed.

1.3.12 Dihydrokoumine (40)

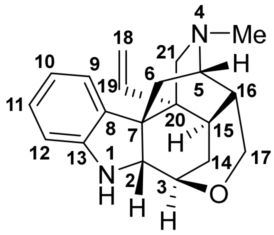

40: Dihydrokoumine

Supplementary Table 18 Comparison of NMR spectra of Dihydrokoumine (**40**)

Position	Natural<br>Ye[25], **2017**<br>(500 MHz, $CDCl_3$)		This work<br>(600 MHz, $CDCl_3$)	
	δ_H mult (*J* in Hz)	δ_C	δ_H mult (*J* in Hz)	δ_C
1			3.77 br s	
2	3.58 br s	75.6	3.57 br s	75.6
3	4.25 dd (4.9, 2.4)	69.1	4.25 d (2.5)	69.1
5	2.74-2.72 m	58.2	2.74-2.72 m	58.2
6α	2.09 dd (14.7, 3.7)	31.4	2.10 dd (15.0, 3.6)	31.5
6β	2.64 m		2.65-2.63 m	
7		46.5		46.5
8		134.1		134.1
9	7.26 d (7.4)	123.6	7.26 d (7.2)	123.6
10	6.85 dd (7.4, 7.4)	119.5	6.85 t (7.2)	119.5
11	7.11 dd (7.4, 7.4)	128.1	7.11 td (7.2, 1.2)	128.1
12	6.72 d (7.4)	110.7	6.72 d (7.2)	110.7
13		151.5		151.6
14α	2.22	20.2	2.24-2.19 m	20.2
14β	2.22		2.24-2.19 m	
15	2.31 d (11.9)	29.5	2.31 d (12.0)	29.5
16	2.64 m	38.5	2.65-2.63 m	38.5
17α	3.63 d (12.0)	61.9	3.63 d (12.0)	61.9
17β	4.18 dd (12.0, 4.9)		4.17 dd (12.0, 4.8)	
18α	4.76-4.66 m	113.7	4.75 d (11.4)	113.7
18β	4.76-4.66 m		4.68 d (18.0)	
19	5.50 dd (17.9, 11.3)	140.9	5.50 dd (18.0, 11.4)	140.9
20		41.9		41.9
21α	2.63 m	58.2	2.65-2.63 m	58.2
21β	2.72 m		2.72 m	
N-Me	2.54 s	42.3	2.54 s	42.3

1.3.13 Koumine (14)

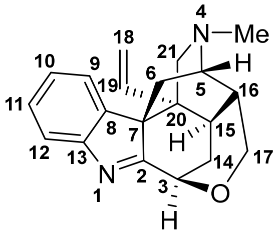

14: Koumine

Supplementary Table 19 Comparison of ^{1}H NMR spectra of Koumine (**14**)

Position	Natural<br>Liu, **1981**[26] & **1986**[27]<br>(200 MHz, $CDCl_3$)<br>δ_H mult (*J* in Hz)	Synthetic<br>Zhang[17], **2021**<br>(400 MHz, $CDCl_3$)<br>δ_H mult (*J* in Hz)	This work<br>(600 MHz, $CDCl_3$)<br>δ_H mult (*J* in Hz)
3	5.016 ddd (3.5, 2.4, 1.0)	5.02 br s	5.02 br s
5	2.795 m	2.87–2.78 m	2.83 br s
6α	2.386 br s	2.42–2.32 m	2.42–2.34 m
6β	2.386 br s	2.42–2.32 m	2.42–2.34 m
9	7.55 d (7.4)	7.55 dd (7.4, 1.3)	7.54 d (7.2)
10	7.25 td (7.4, 1.3)	7.25 t (7.4)	7.25 td (7.2, 1.2)
11	7.36 td (7.4, 1.3)	7.35 t (7.6)	7.35 td (7.8, 1.2)
12	7.61 d (7.6)	7.61 dd (7.4, 1.3)	7.61 d (7.8)
14α	2.610 dt (14.0, 3.5 H)	2.61–2.58 m	2.62–2.59 m
14β	1.878 dd (14.0, 2.4)	1.88 dd (14.6)	1.88 dd (15.0)
15	2.346 br d (10.0)	2.42–2.32 m	2.42–2.34 m
16	2.785 (m, 1H)	2.87–2.78 m	2.83 br s
17α	4.258 dd (12.2, 4.4)	4.26 dd (11.9, 3.3)	4.26 dd (12.0, 4.2)
17β	3.619 d (12.2)	3.62 d (12.0)	3.62 d (11.4)
18α	4.90–4.60 m	4.83 d (17.4)	4.83 d (18.0)
18β	4.90–4.60 m	4.79 d (11.2)	4.79 d (10.8)
19	4.90–4.60 m	4.68 dd (17.4, 11.2)	4.68 dd (18.0, 10.8)
21α	3.173 d	3.17 d (11.4)	3.18 d (11.4)
21β	3.106 d	3.09 d (12.0)	3.13 d (11.4)
N-Me	2.615 s	2.61 s	2.63 s

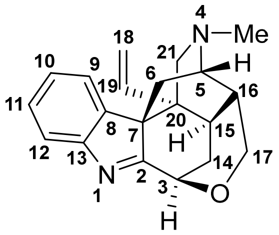

14: Koumine

Supplementary Table 20 Comparison of ^{13}C NMR spectra of Koumine (**14**)

Position	Natural<br>Liu, **1981**[26] & **1986**[27]<br>(50 MHz, CDCl3)<br>δC	Synthetic<br>Zhang[17], **2021**<br>(100 MHz, CDCl3)<br>δC	This work<br>(150 MHz, CDCl3)<br>δC
2	185.7	185.5	185.5
3	71.0	71.0	71.1
5	56.9	56.9	57.2
6	28.6	28.6	28.7
7	58.0	58.0	58.1
8	143.7	143.7	143.7
9	123.1	123.0	123.2
10	126.0	126.0	126.2
11	128.2	128.2	128.4
12	121.1	121.1	121.3
13	154.9	154.9	155.1
14	25.3	25.3	25.4
15	38.9	38.9	38.9
16	33.1	33.1	33.2
17	61.4	61.4	61.4
18	115.9	115.8	116.1
19	137.3	137.3	137.3
20	45.3	45.3	45.4
21	57.8	57.8	57.8
N-Me	42.7	42.7	42.7

1.5 Copies of ^{1}H and ^{13}C NMR Spectra

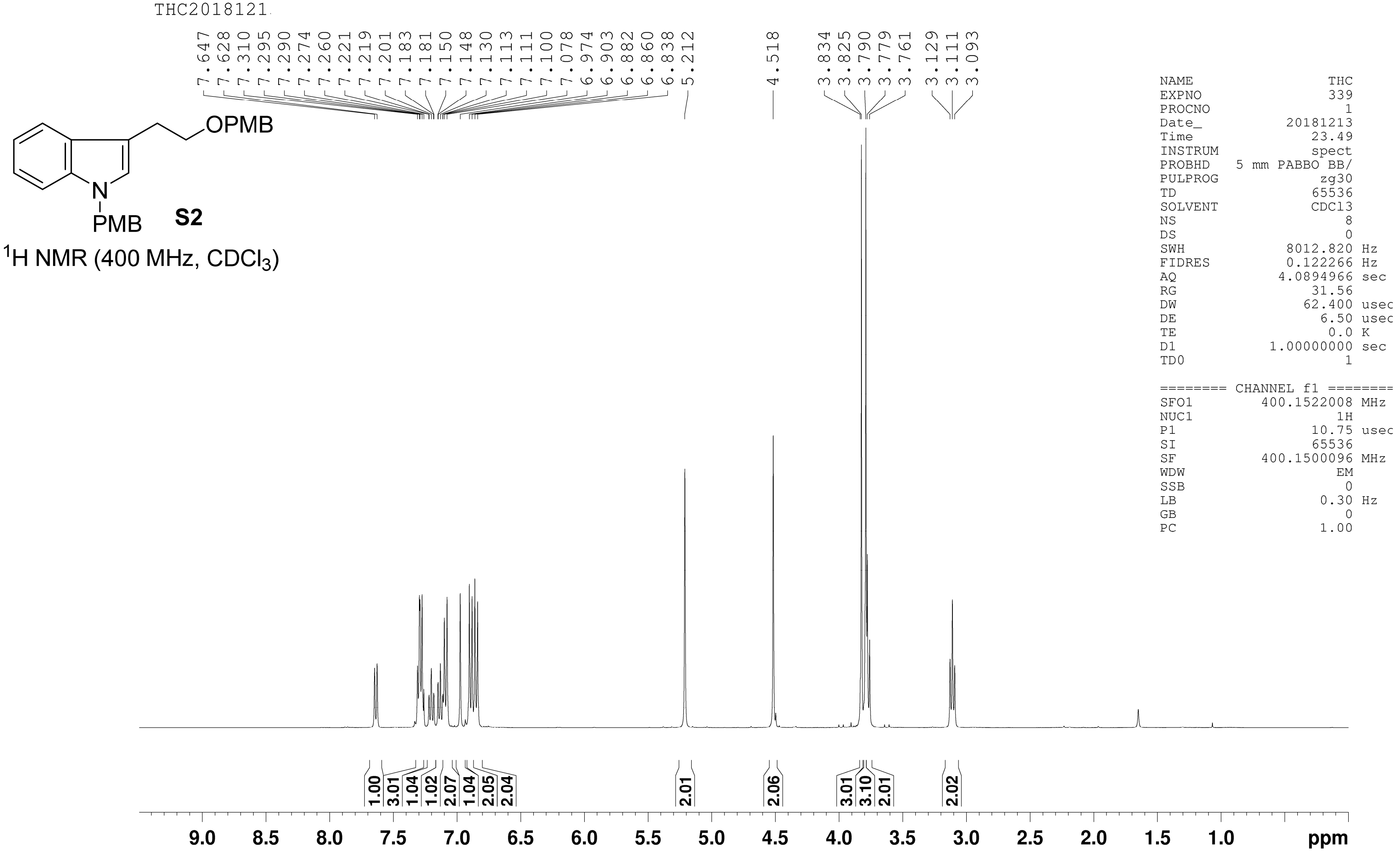

Supplementary Figure 1 ^{1}H-NMR (400 MHz, $CDCl_3$) spectra of **S2**

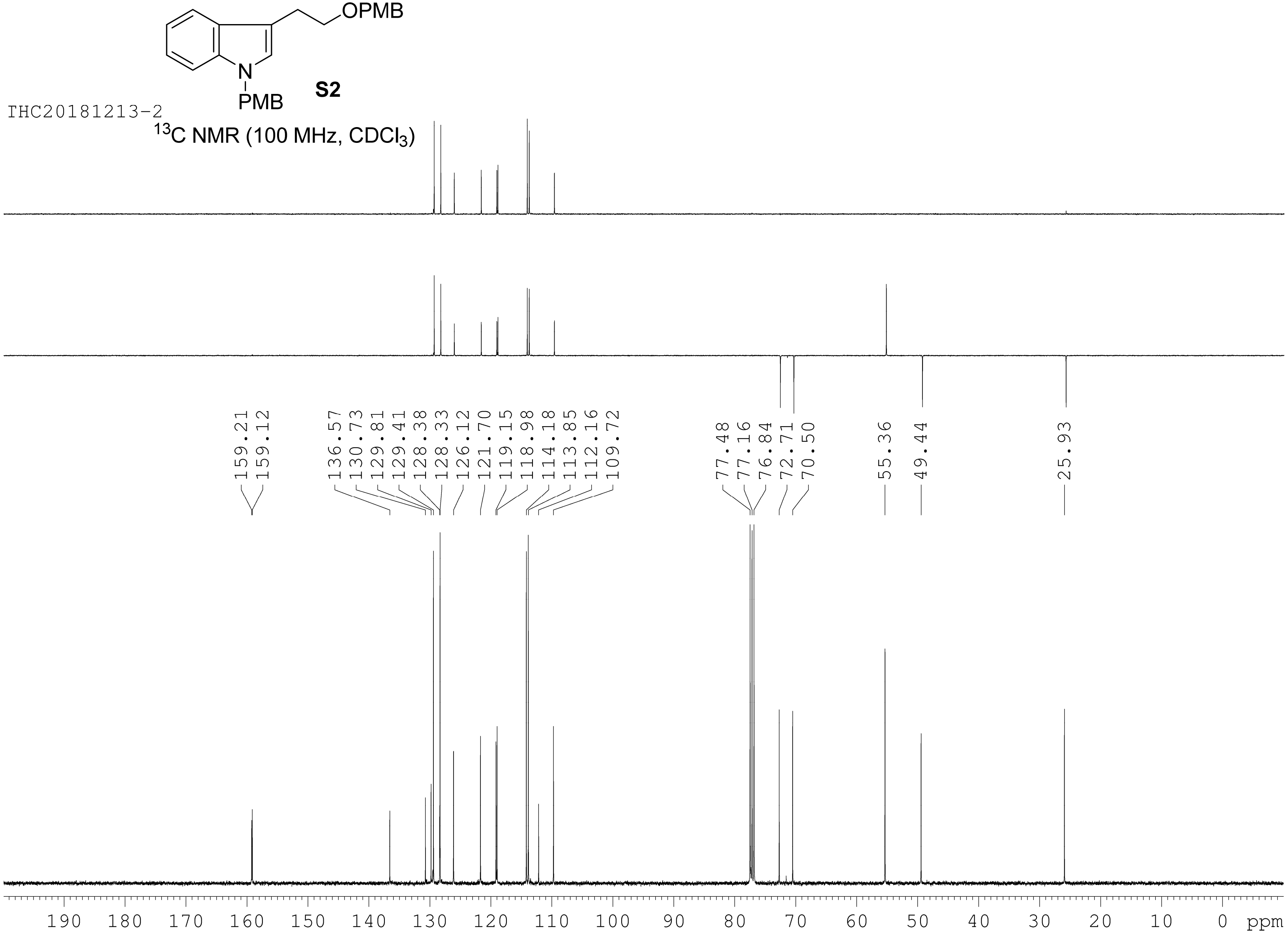

Supplementary Figure 2 ^{13}C-NMR (100 MHz, $CDCl_3$) spectra of **S2**

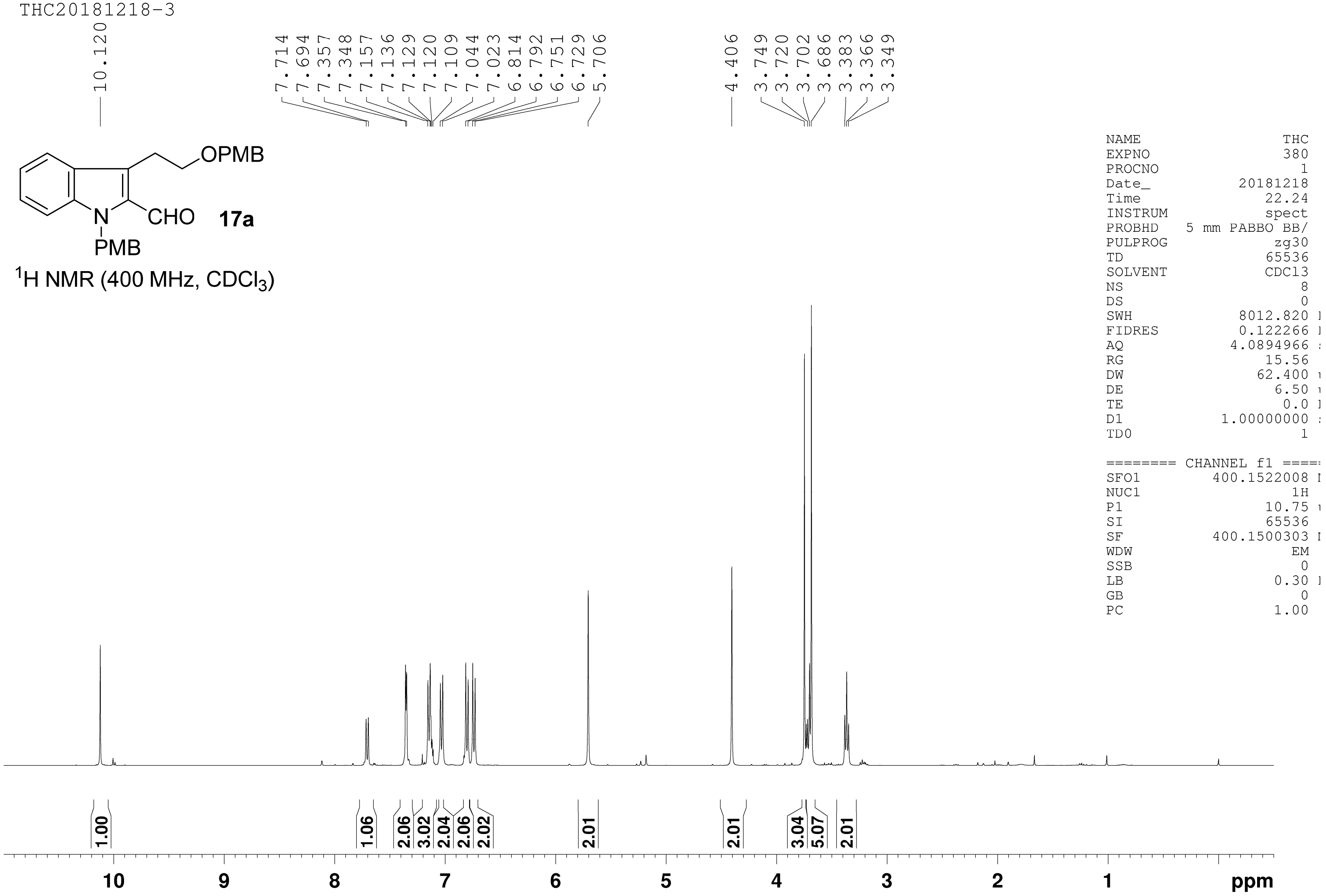

Supplementary Figure 3 ^{1}H-NMR (400 MHz, $CDCl_3$) spectra of **17a**

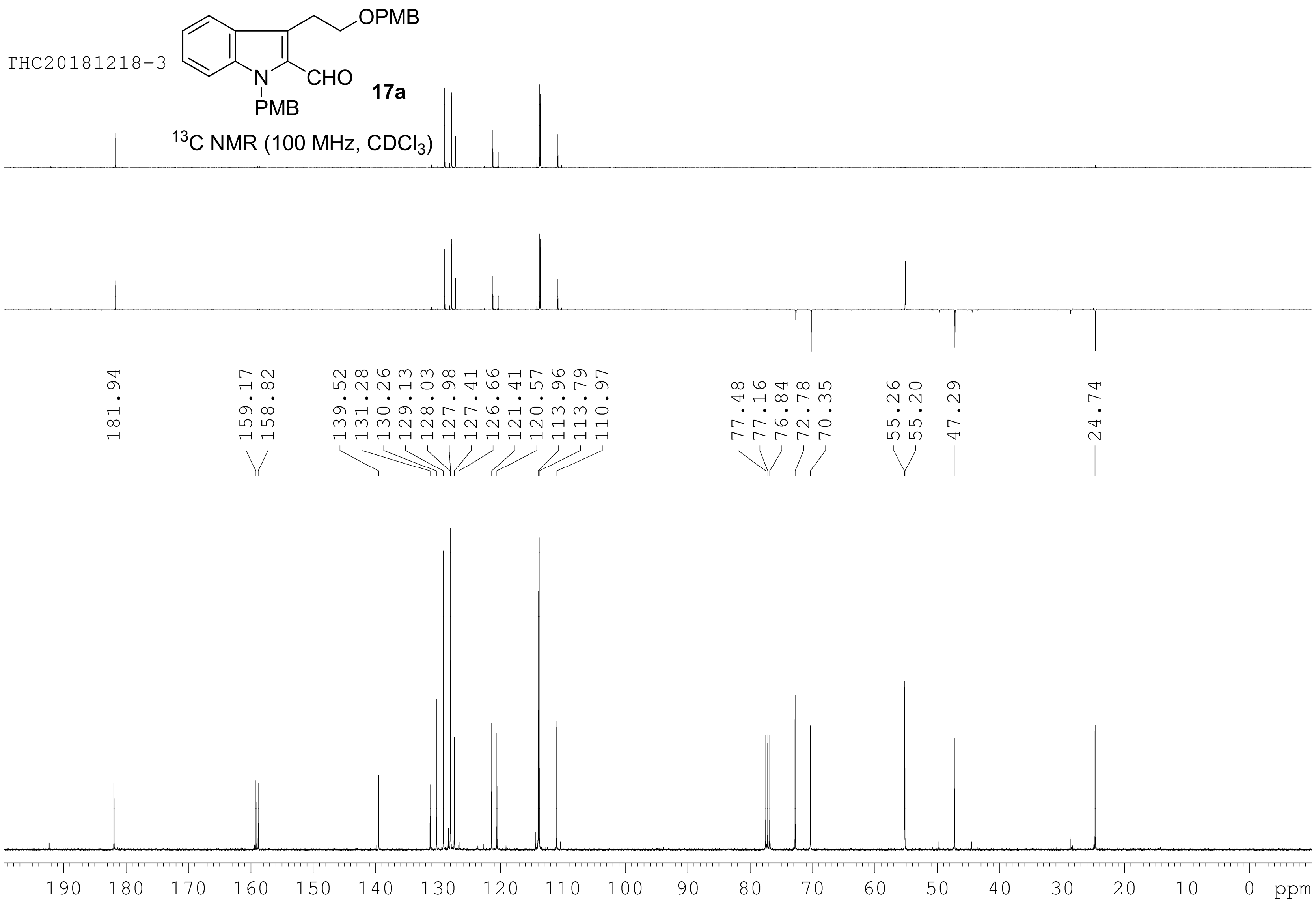

Supplementary Figure 4 ^{13}C-NMR (100 MHz, $CDCl_3$) spectra of **17a**

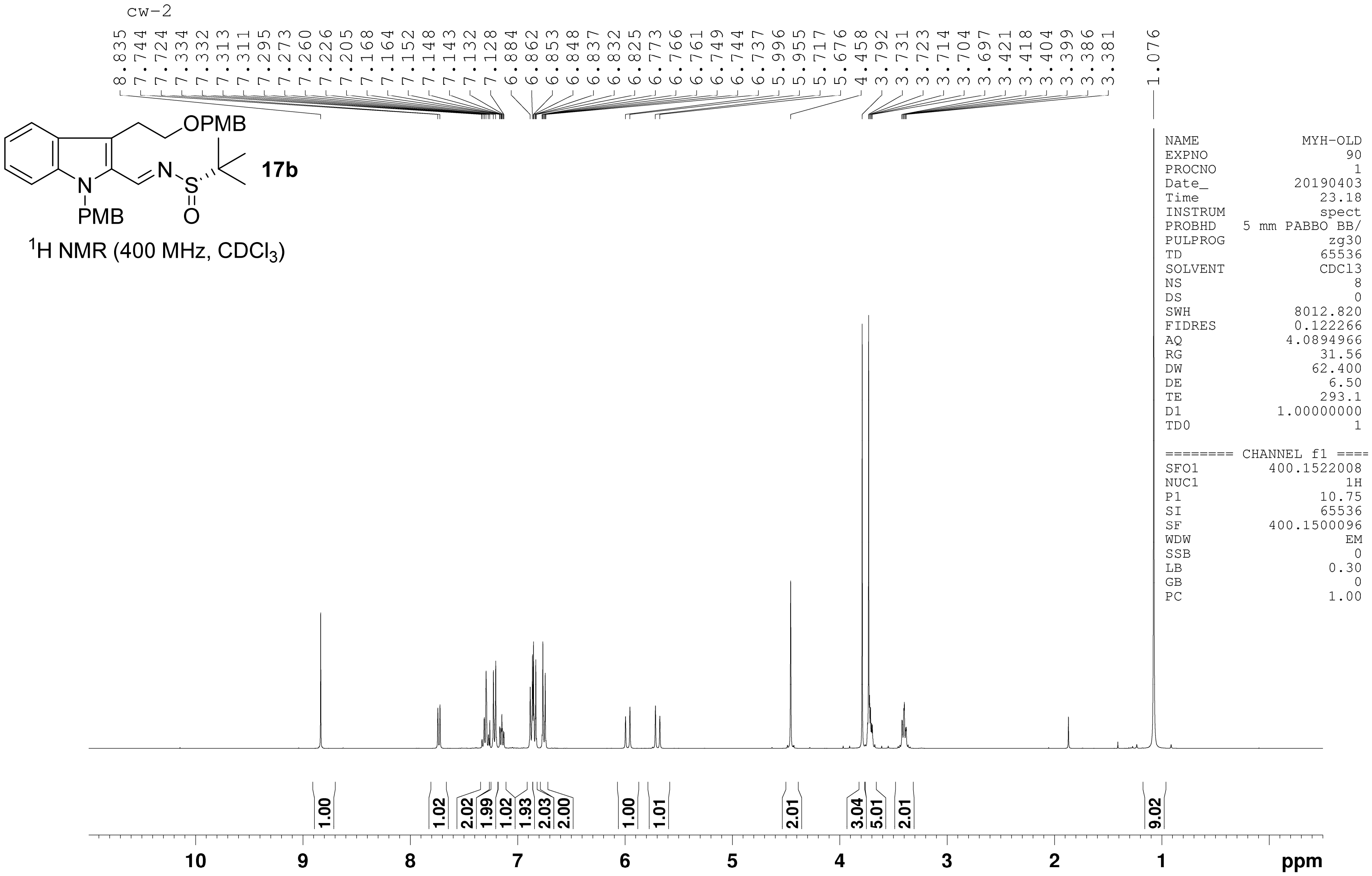

Supplementary Figure 5 ¹H-NMR (400 MHz, $CDCl_3$) spectra of **17b**

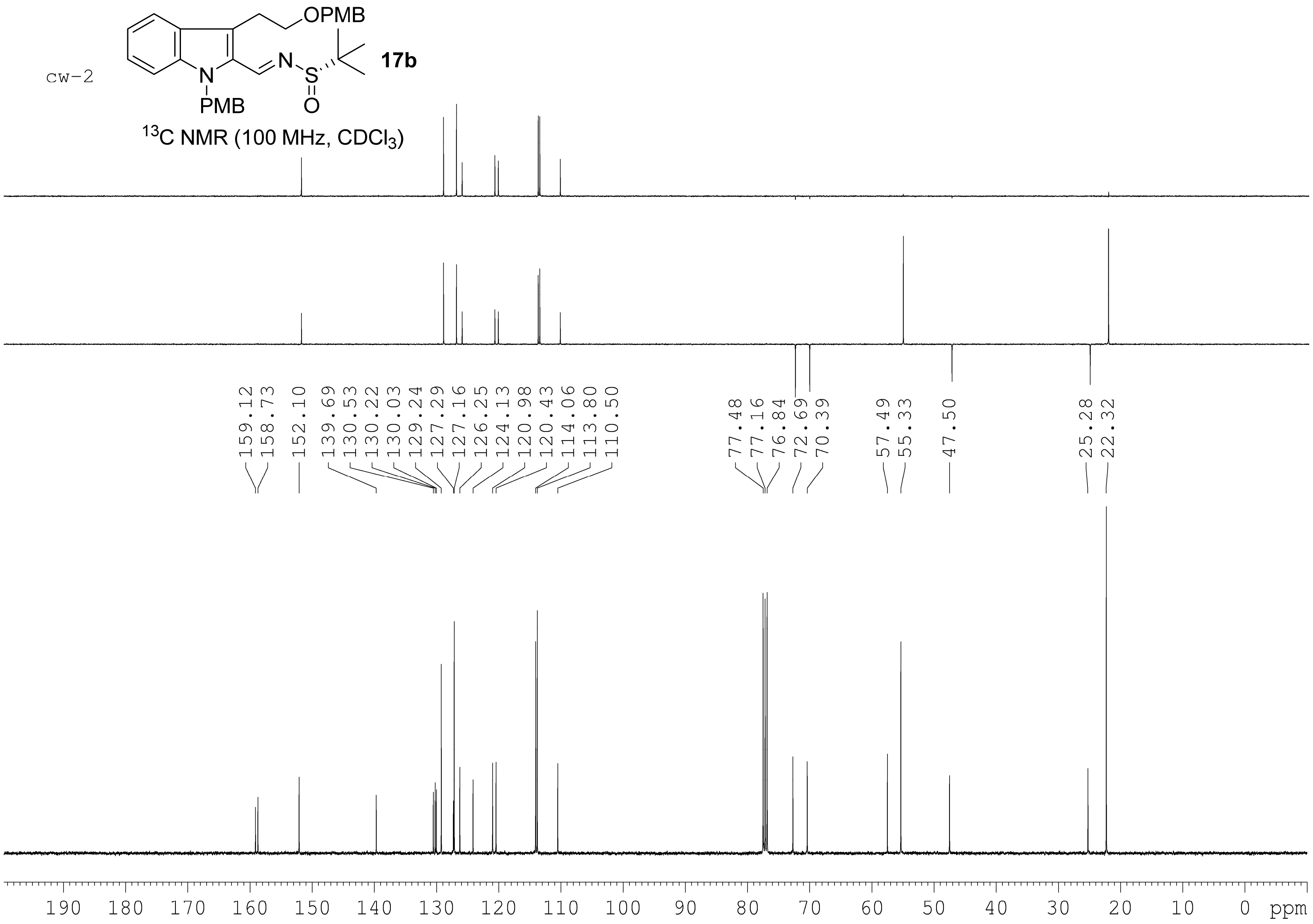

Supplementary Figure 6 ^{13}C-NMR (100 MHz, $CDCl_3$) spectra of **17b**

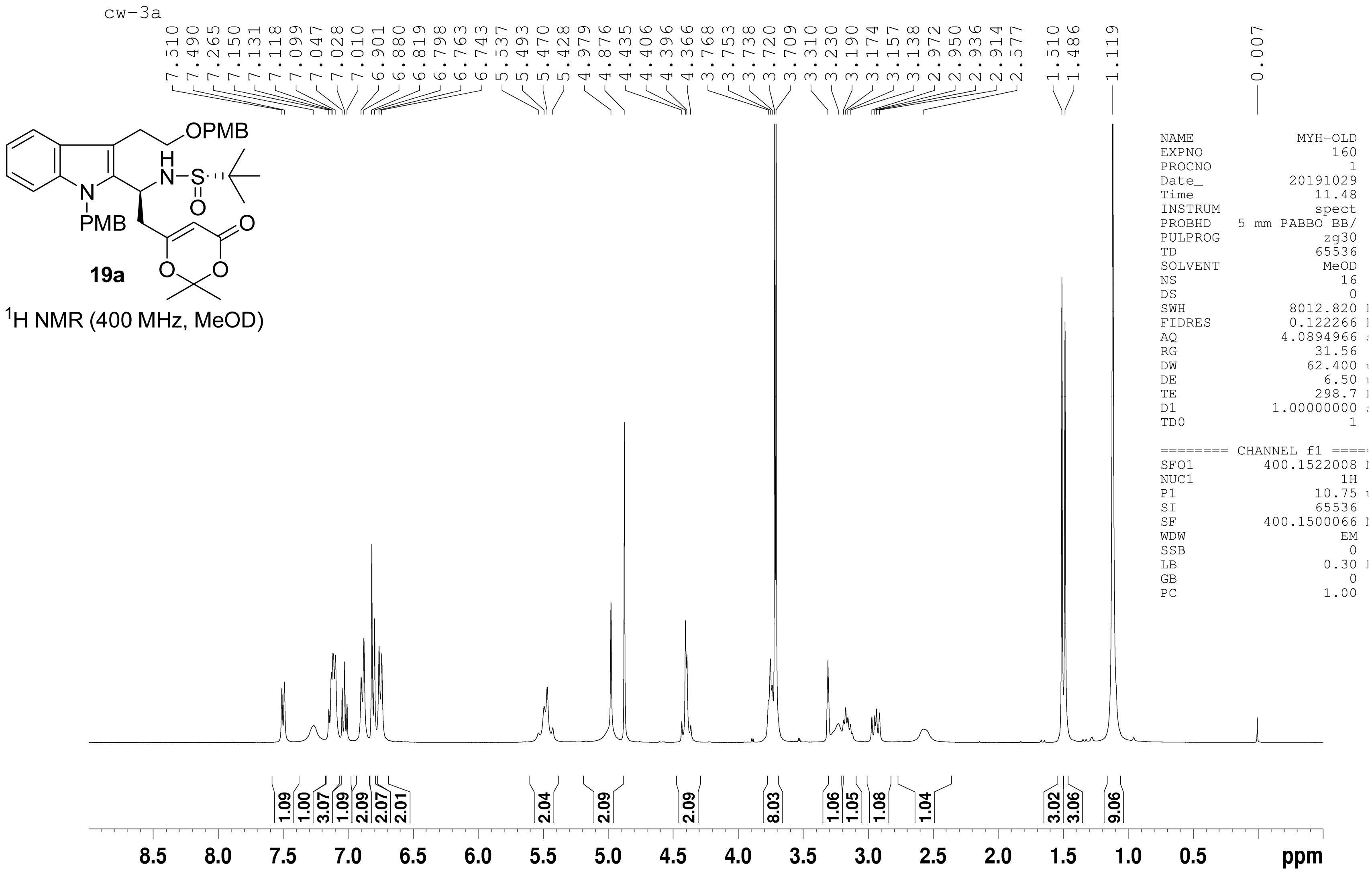

Supplementary Figure 7 ^{1}H-NMR (400 MHz, MeOD) spectra of **19a**

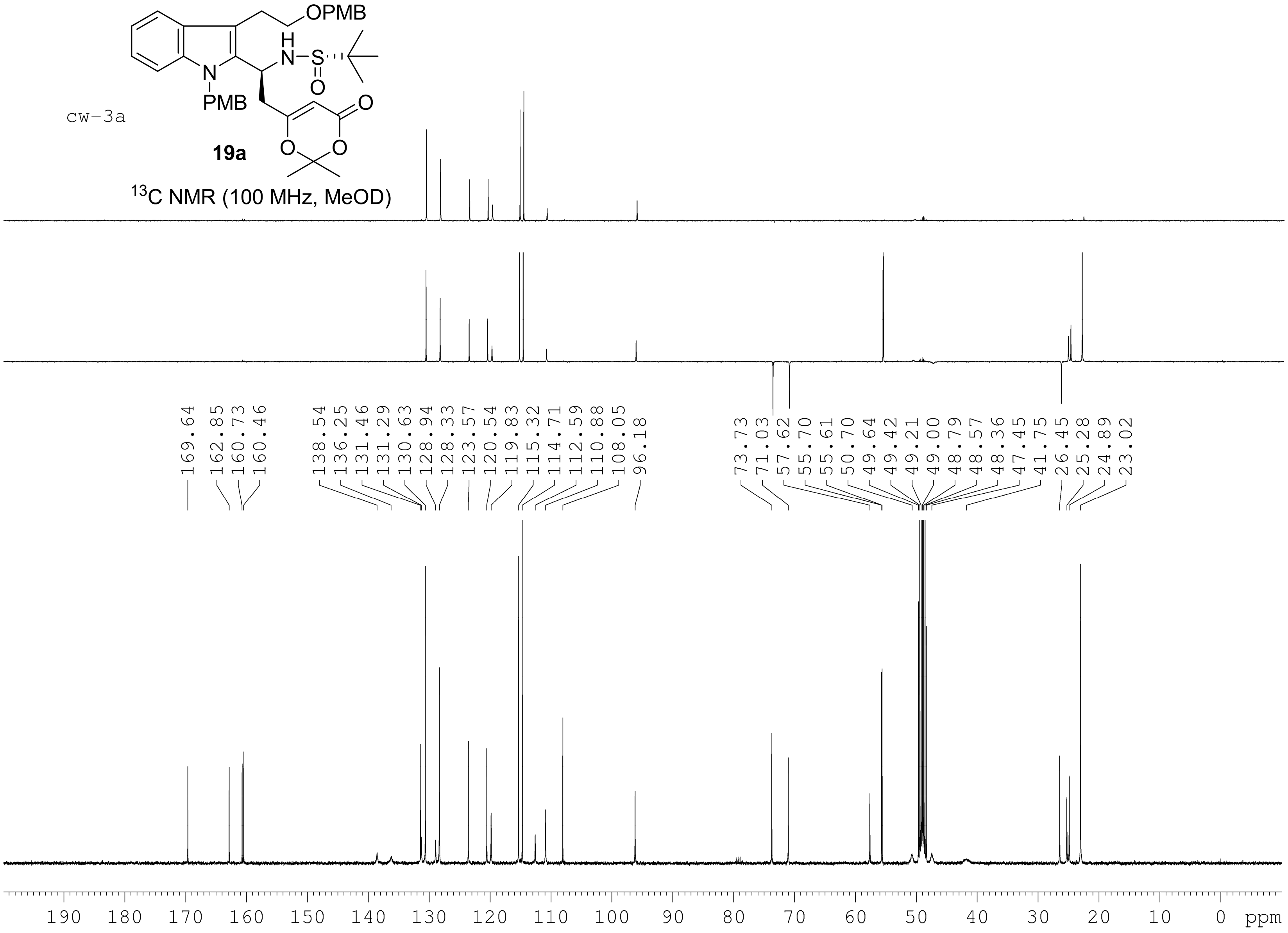

Supplementary Figure 8 ^{13}C-NMR (100 MHz, $CDCl_3$) spectra of **19a**

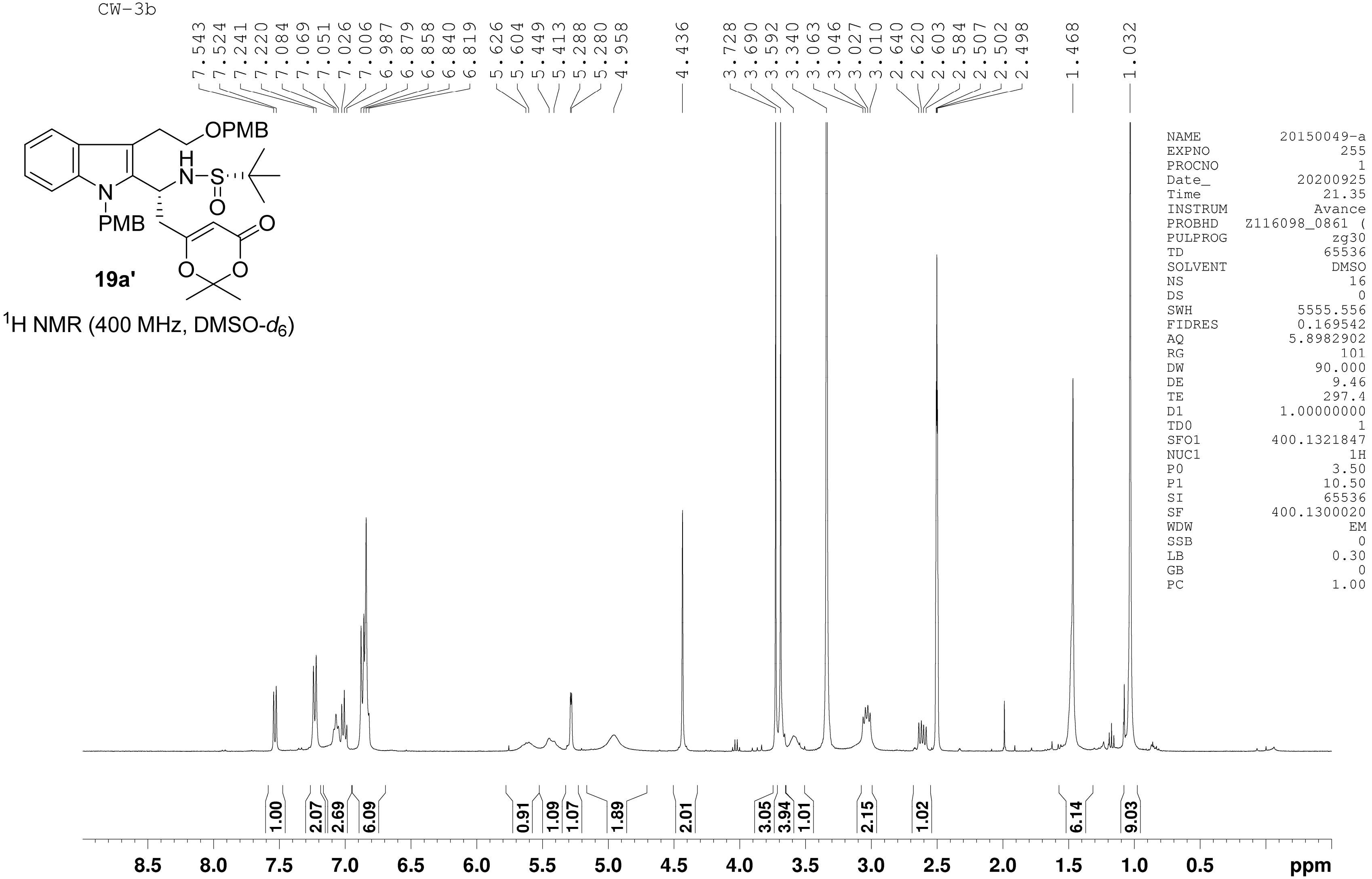

Supplementary Figure 9 ^{1}H-NMR (400 MHz, DMSO-d_6) spectra of **19a'**

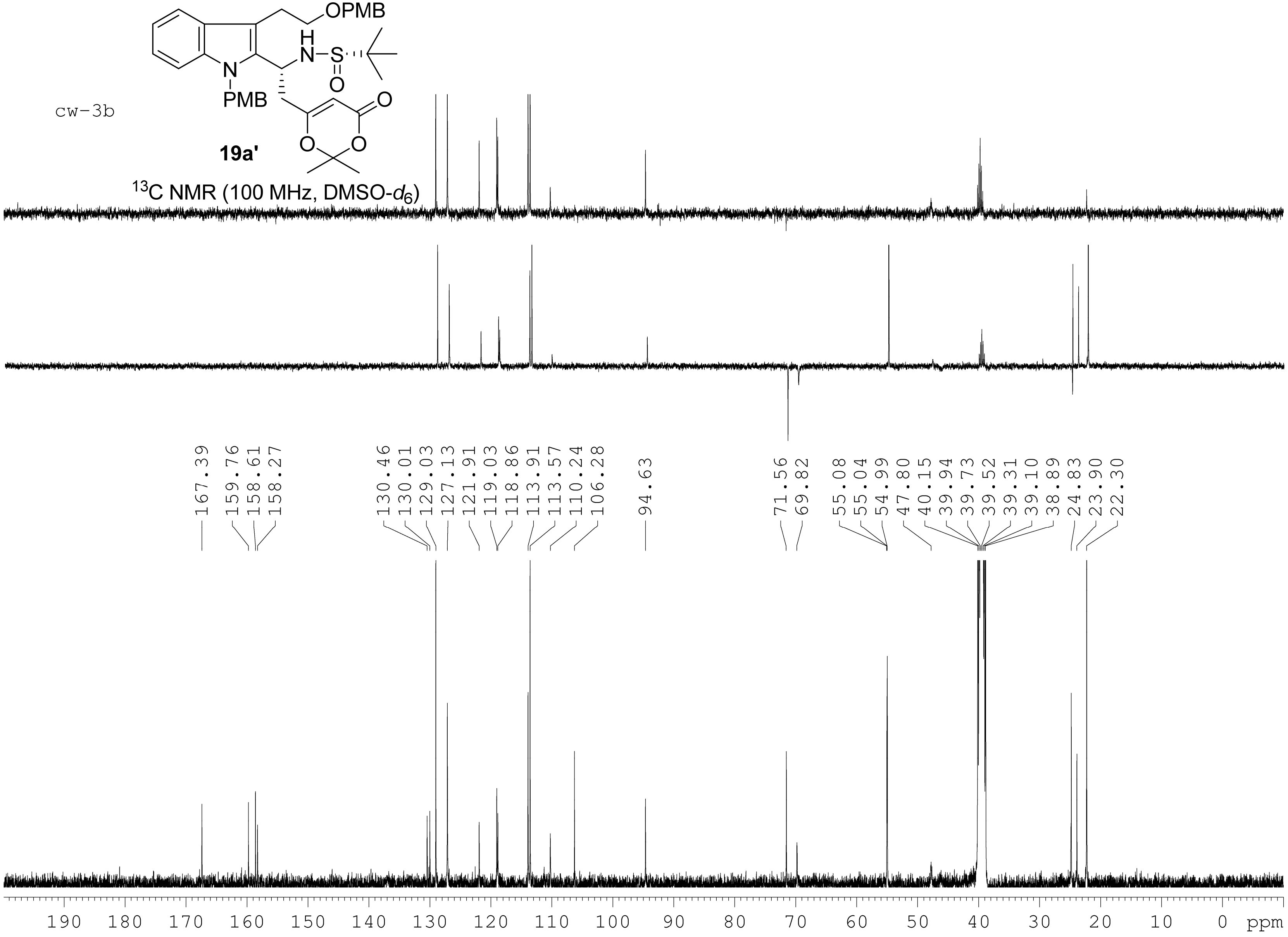

Supplementary Figure 10 ^{13}C-NMR (100 MHz, DMSO-d_6) spectra of **19a'**

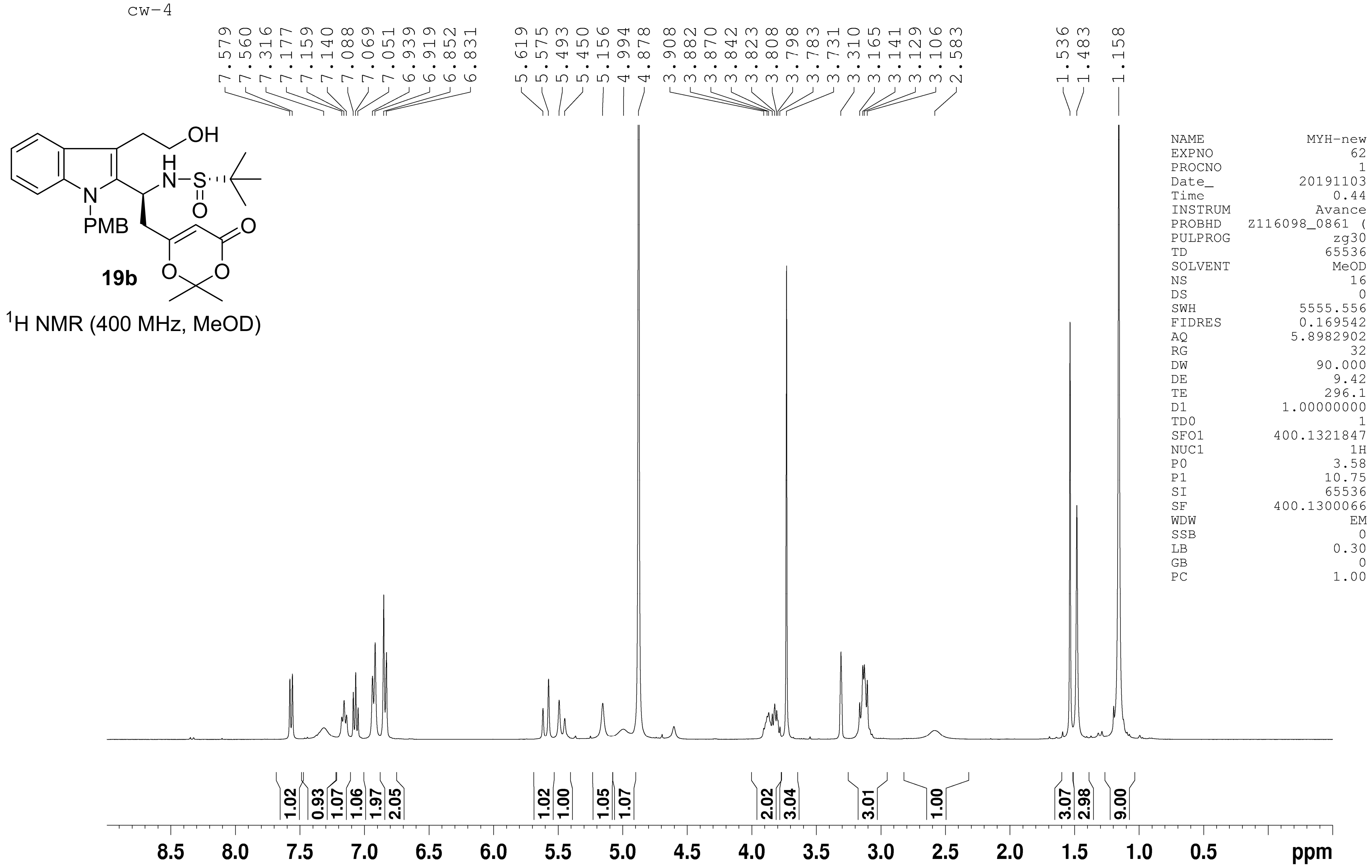

Supplementary Figure 11 ^{1}H-NMR (400 MHz, MeOD) spectra of **19b**

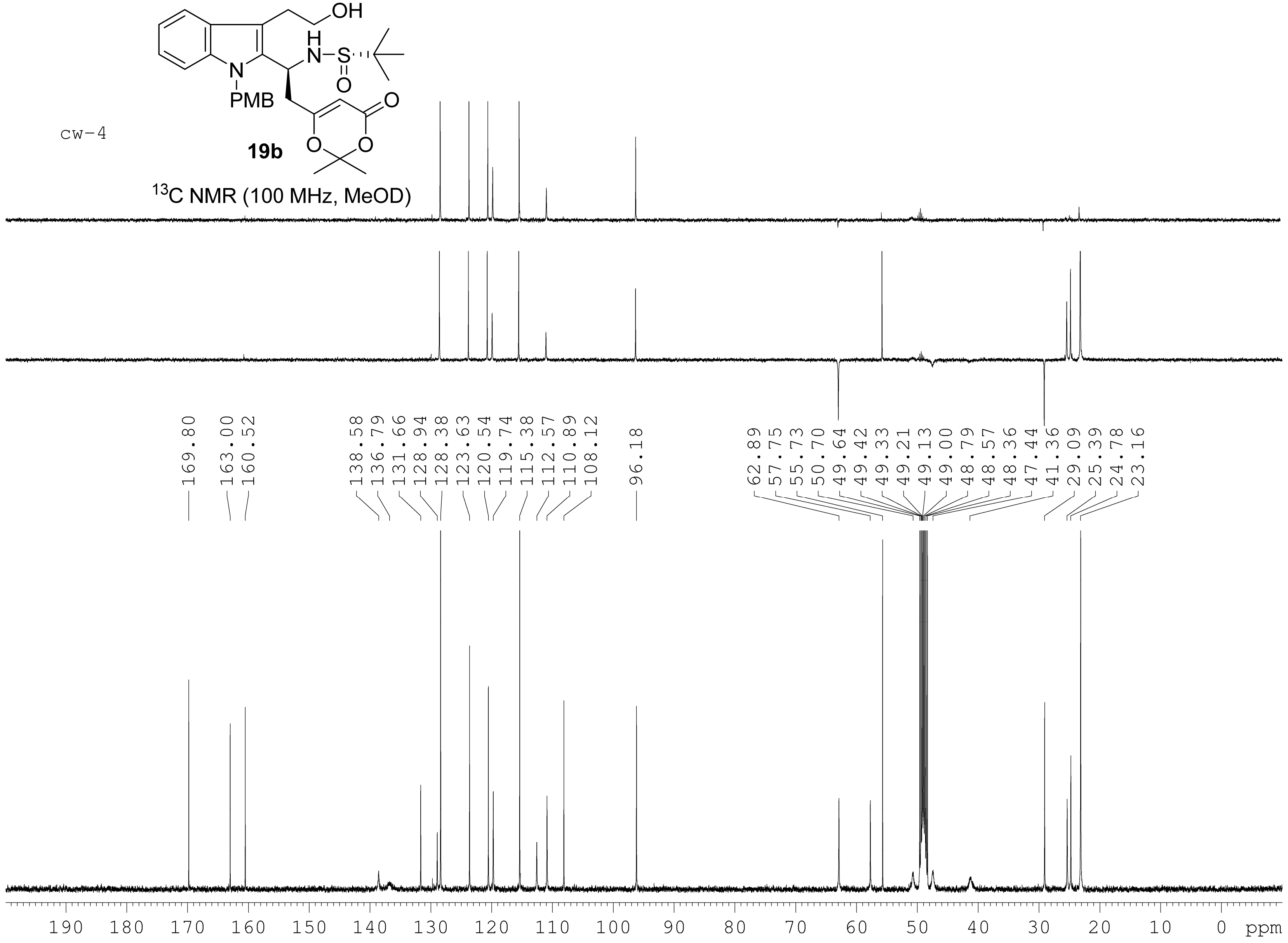

Supplementary Figure 12 ^{13}C-NMR (100 MHz, MeOD) spectra of **19b**

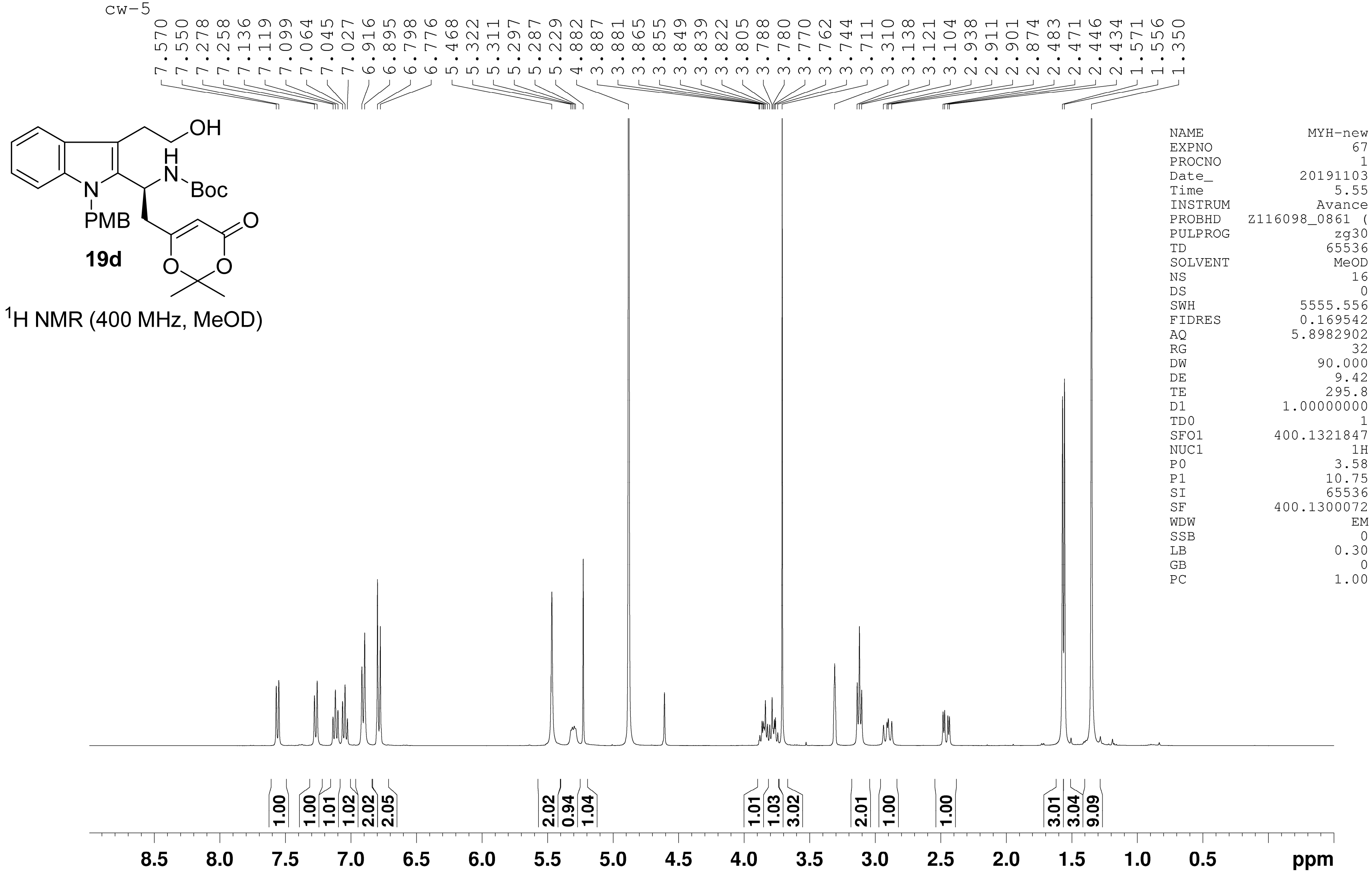

Supplementary Figure 13 ^{1}H-NMR (400 MHz, MeOD) spectra of **19d**

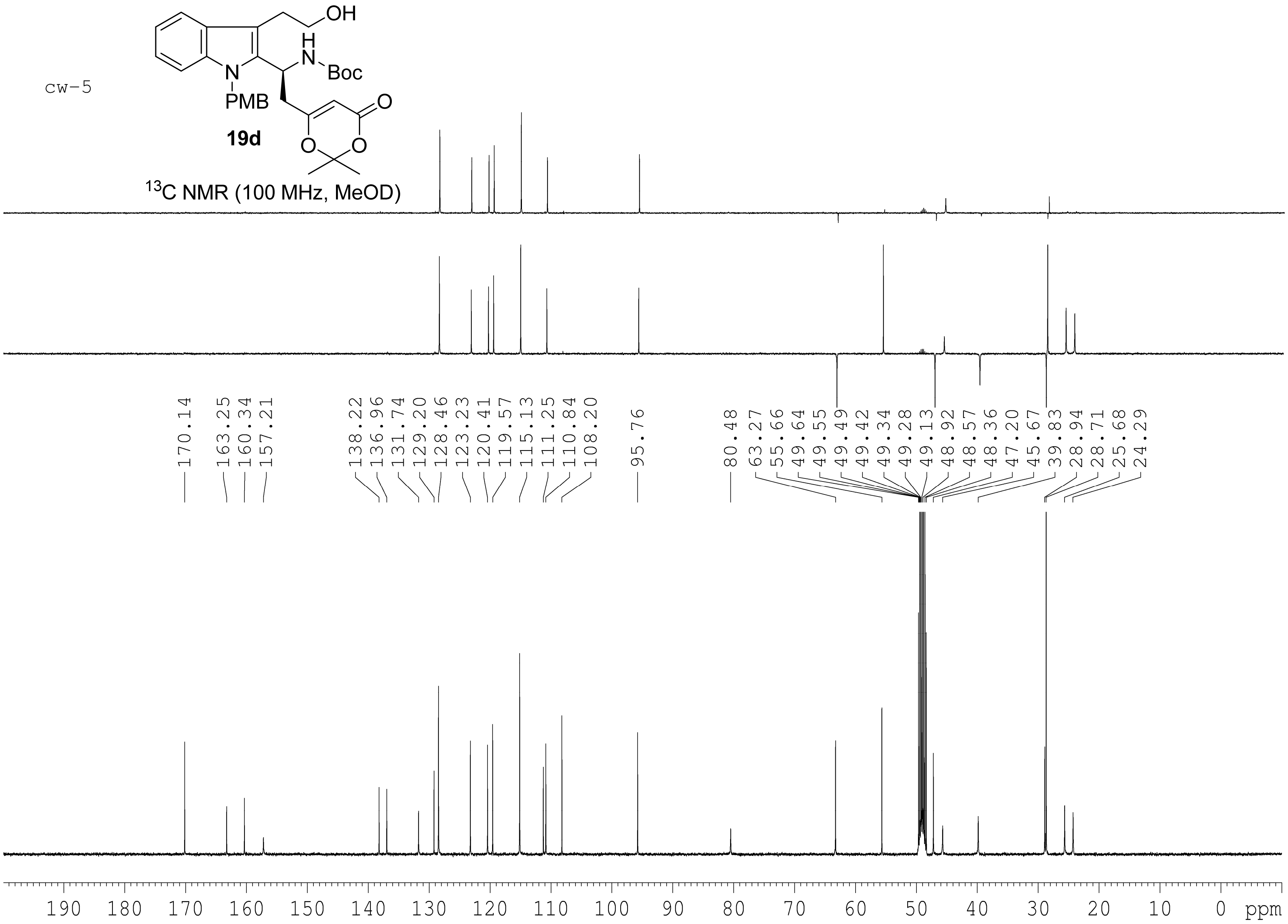

Supplementary Figure 14 ^{13}C-NMR (100 MHz, MeOD) spectra of **19d**

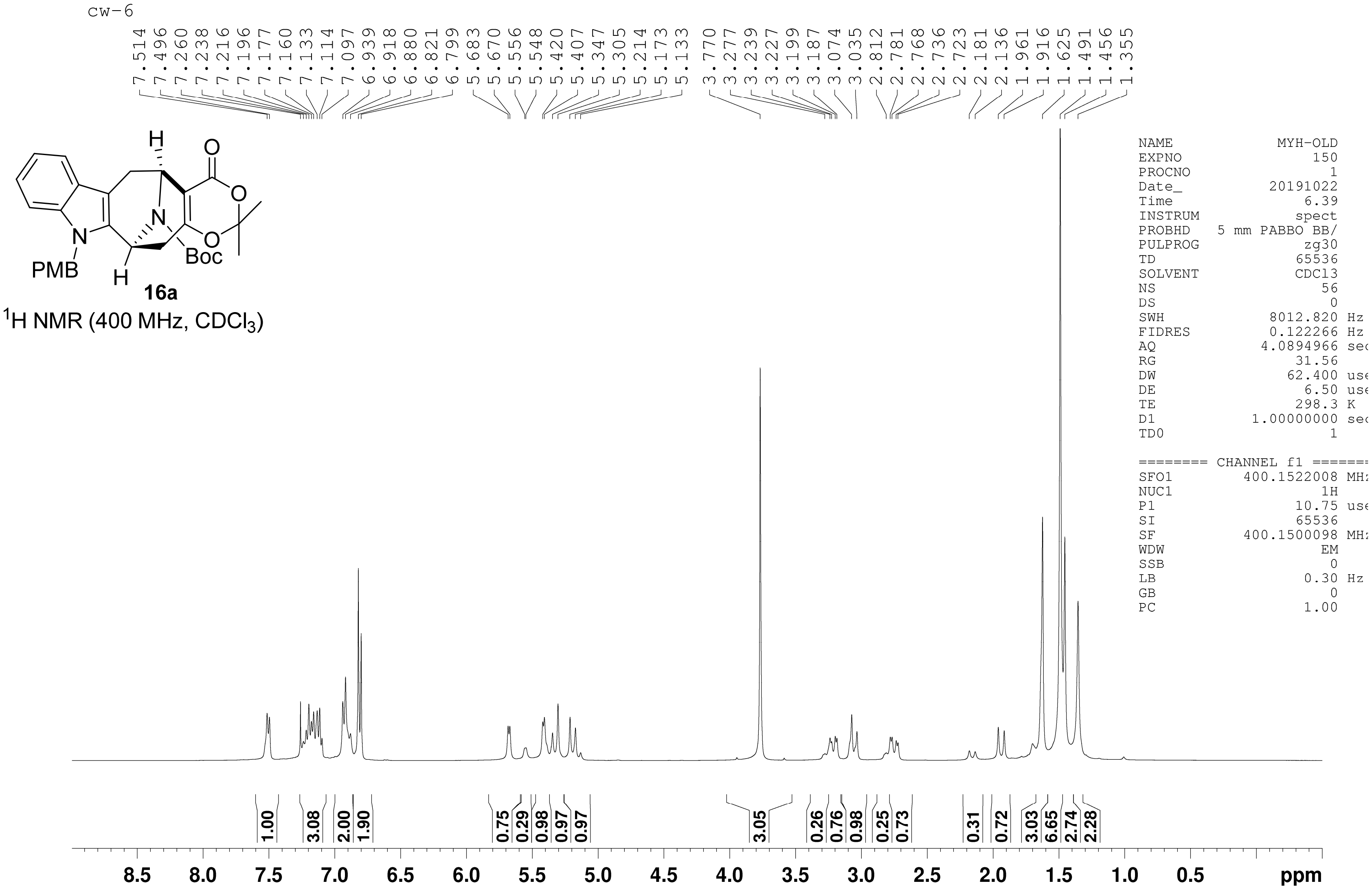

Supplementary Figure 15 ^{1}H-NMR (400 MHz, $CDCl_3$) spectra of **16a**

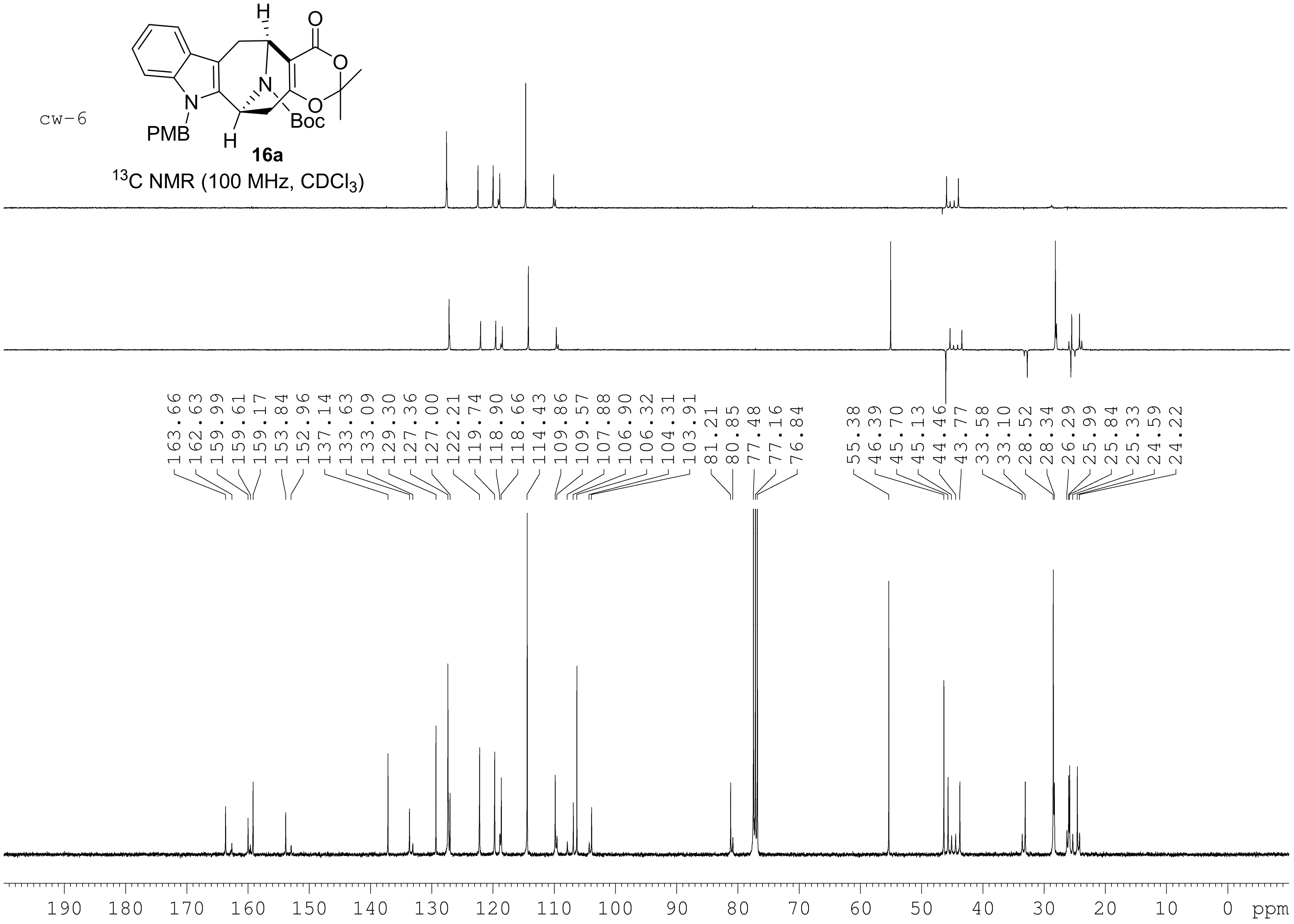

Supplementary Figure 16 ^{13}C-NMR (100 MHz, CDCl$_3$) spectra of **16a**

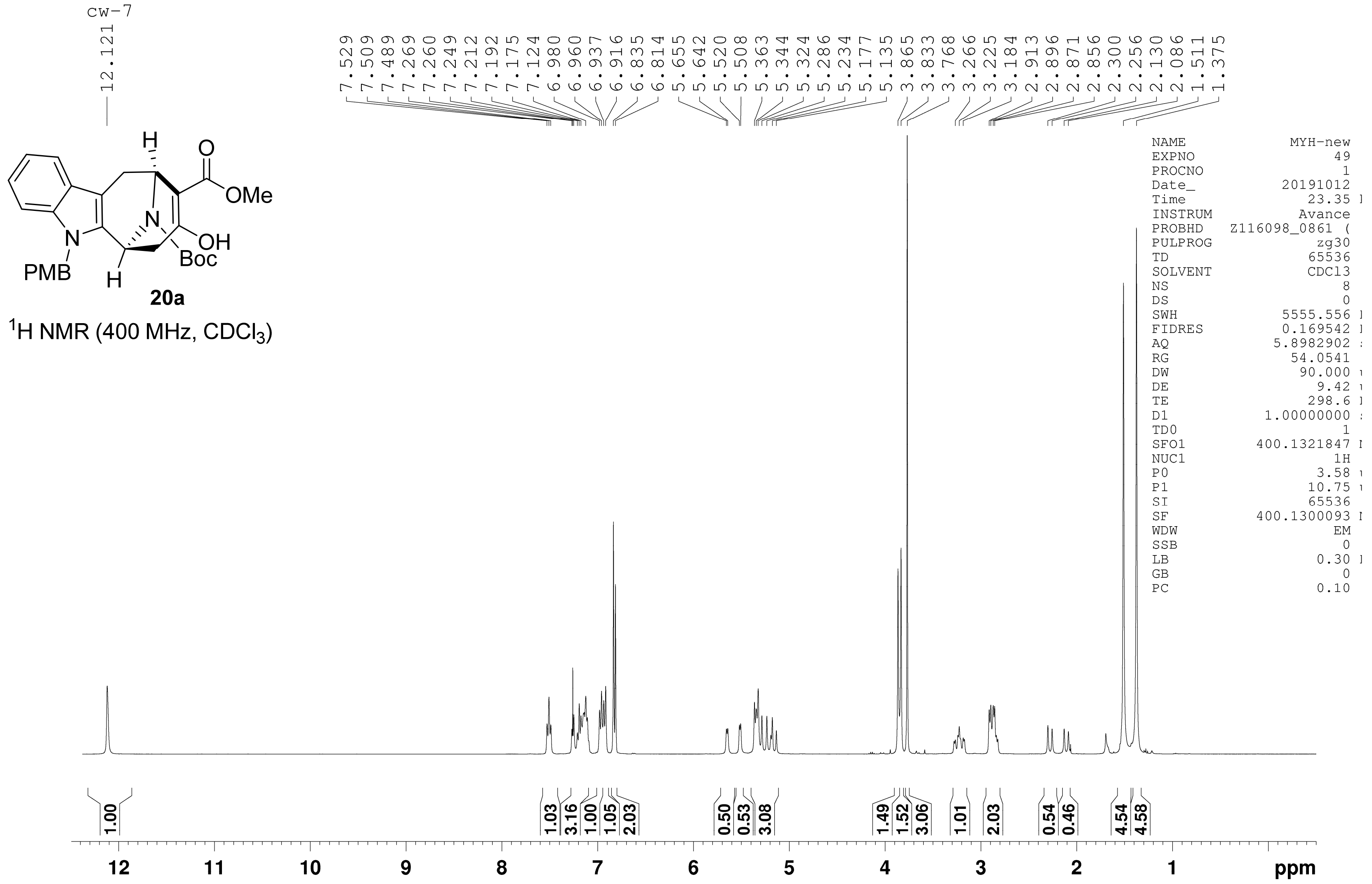

Supplementary Figure 17 ^{1}H-NMR (400 MHz, $CDCl_3$) spectra of **20a**

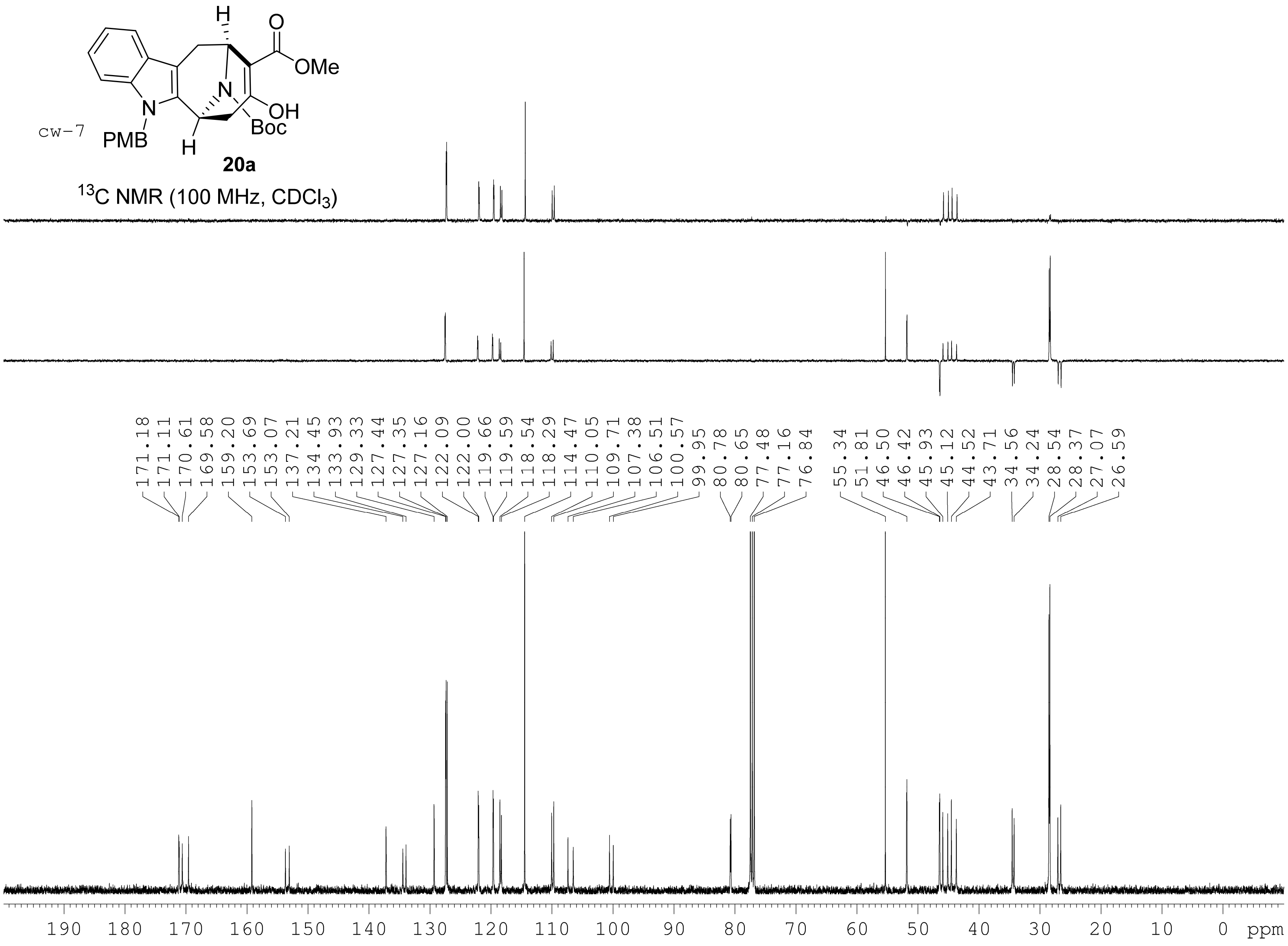

Supplementary Figure 18 ^{13}C-NMR (100 MHz, $CDCl_3$) spectra of **20a**

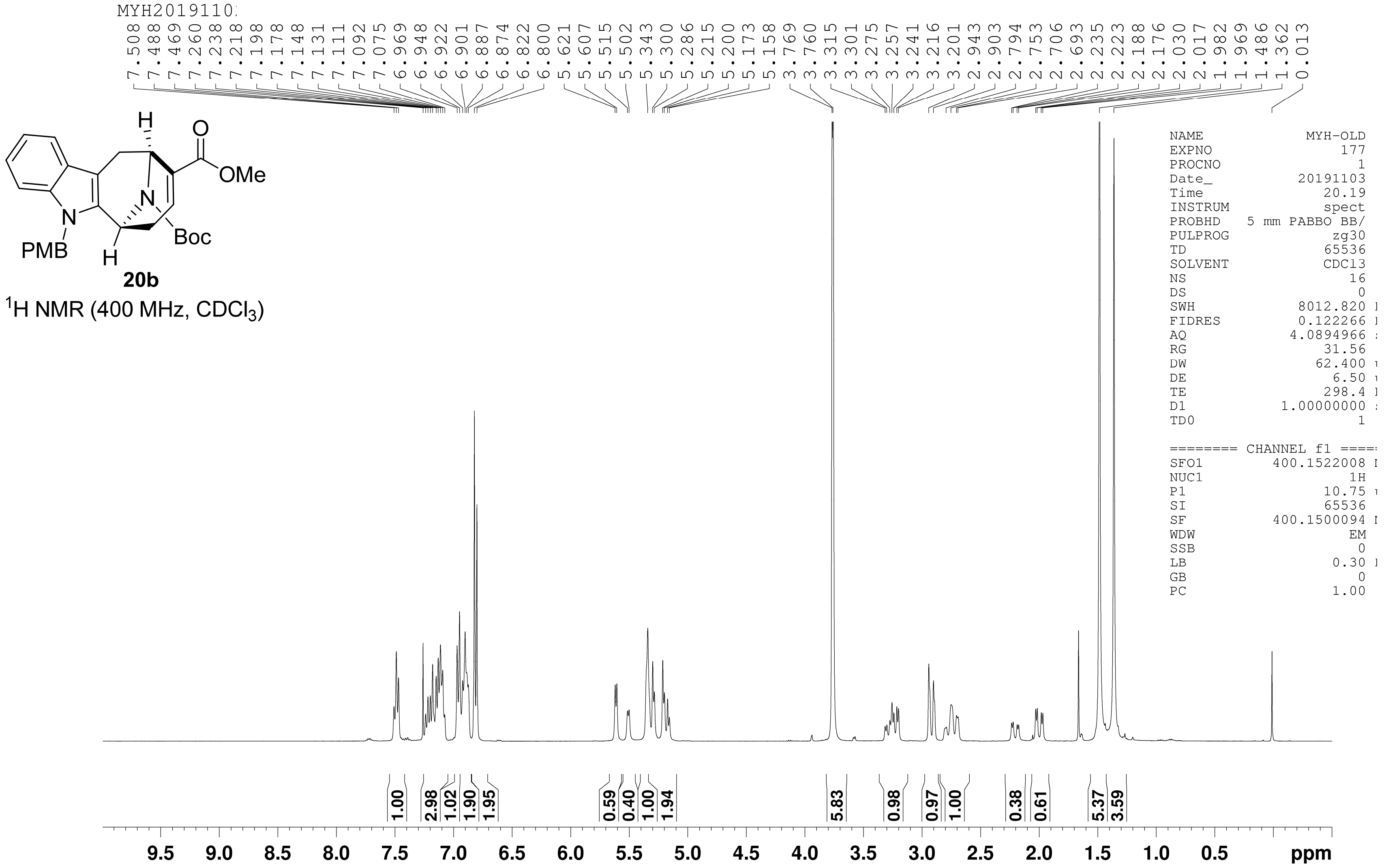

Supplementary Figure 19 ^{1}H-NMR (400 MHz, $CDCl_3$) spectra of **20b**

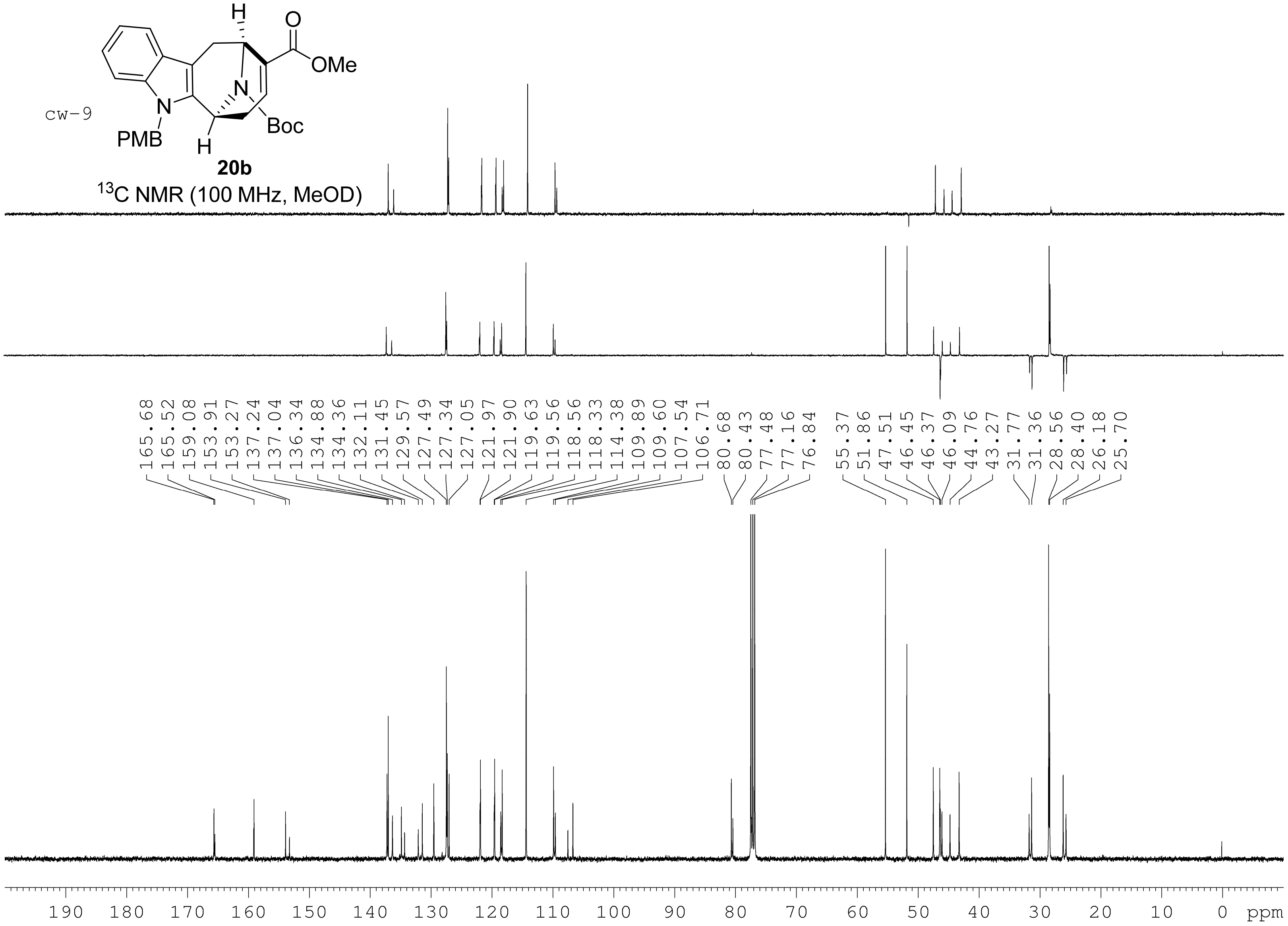

Supplementary Figure 20 ^{13}C-NMR (100 MHz, CDCl$_3$) spectra of **20b**

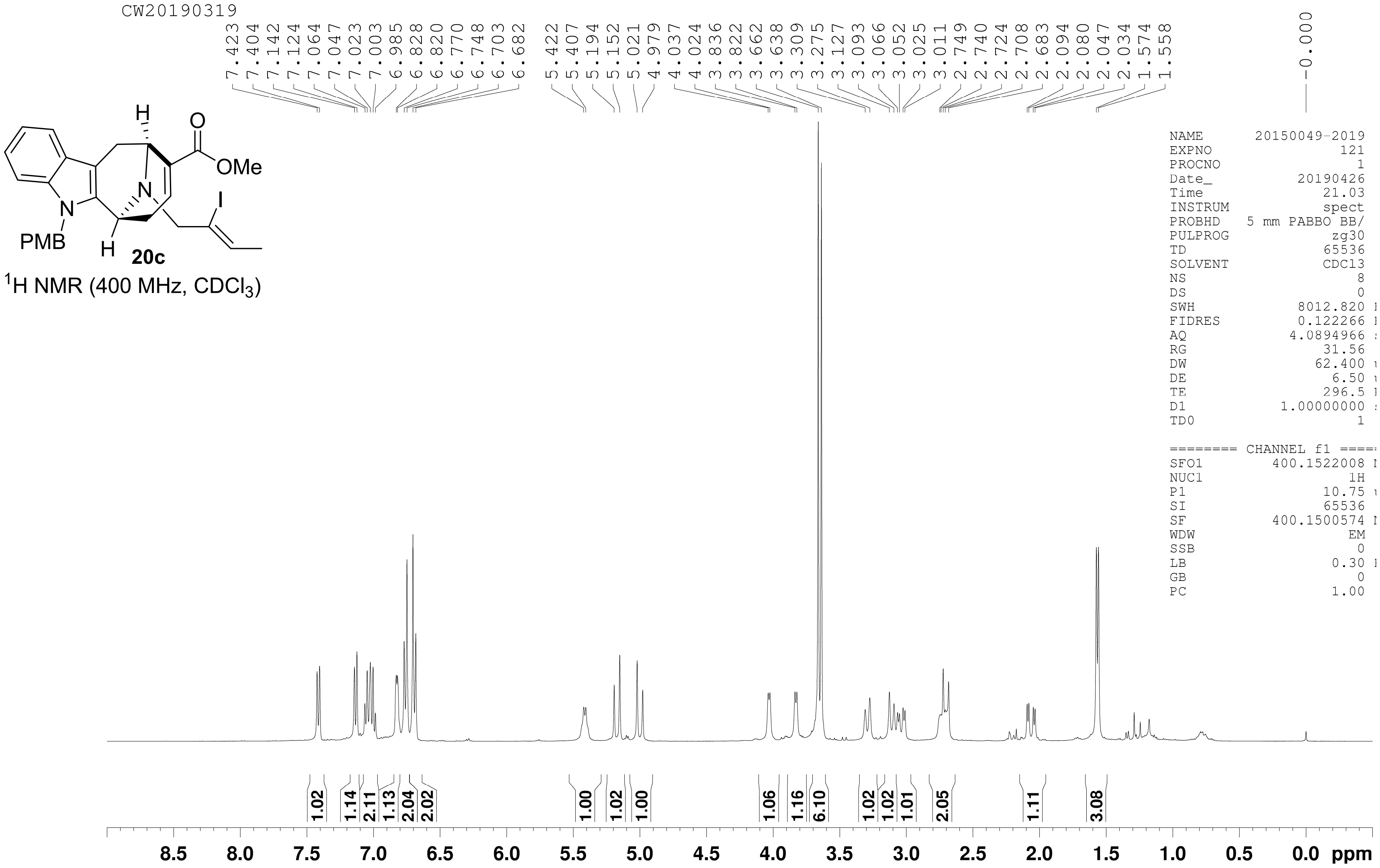

Supplementary Figure 21 ^{1}H-NMR (400 MHz, $CDCl_3$) spectra of **20c**

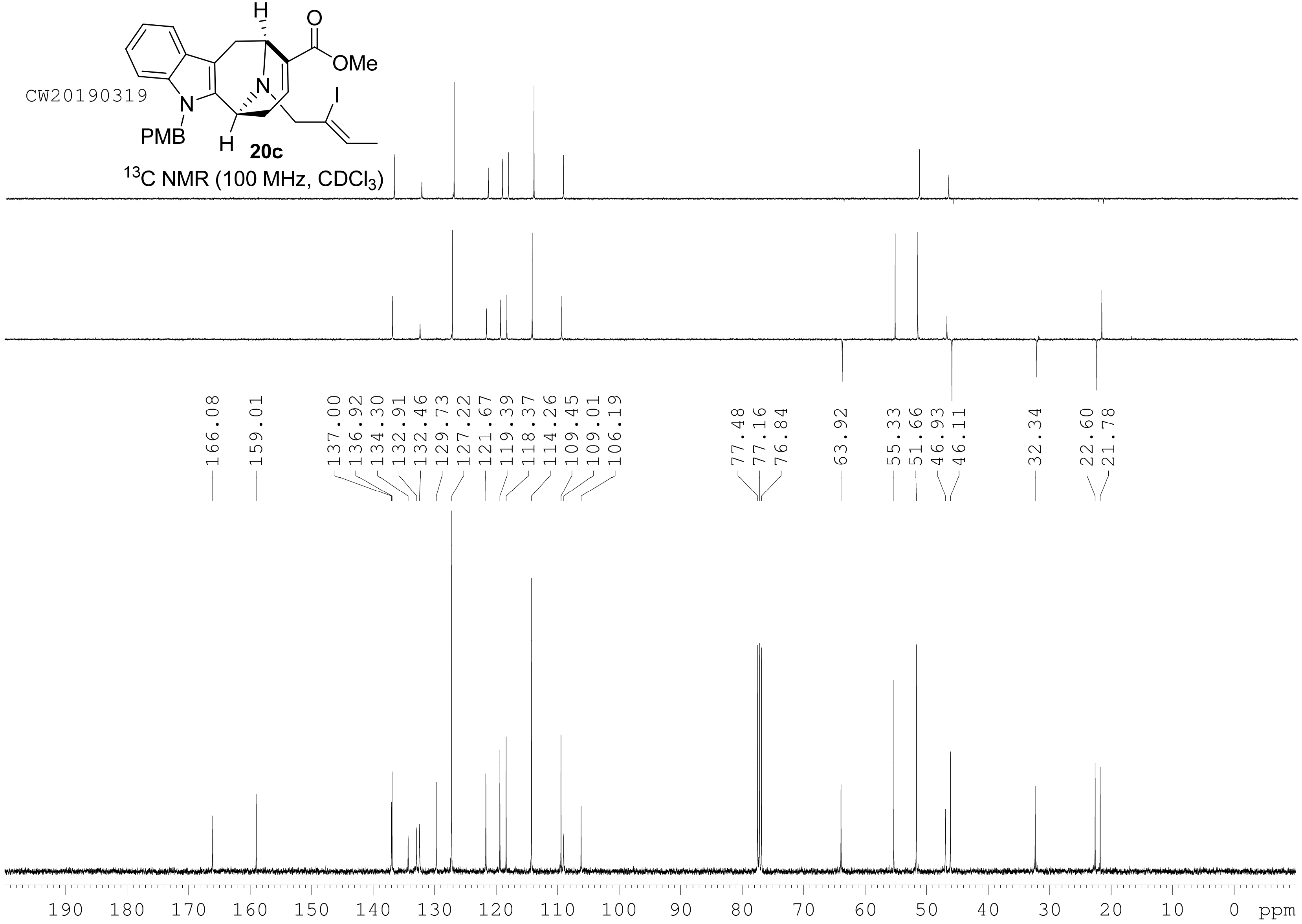

Supplementary Figure 22 ^{13}C-NMR (100 MHz, CDCl$_3$) spectra of **20c**

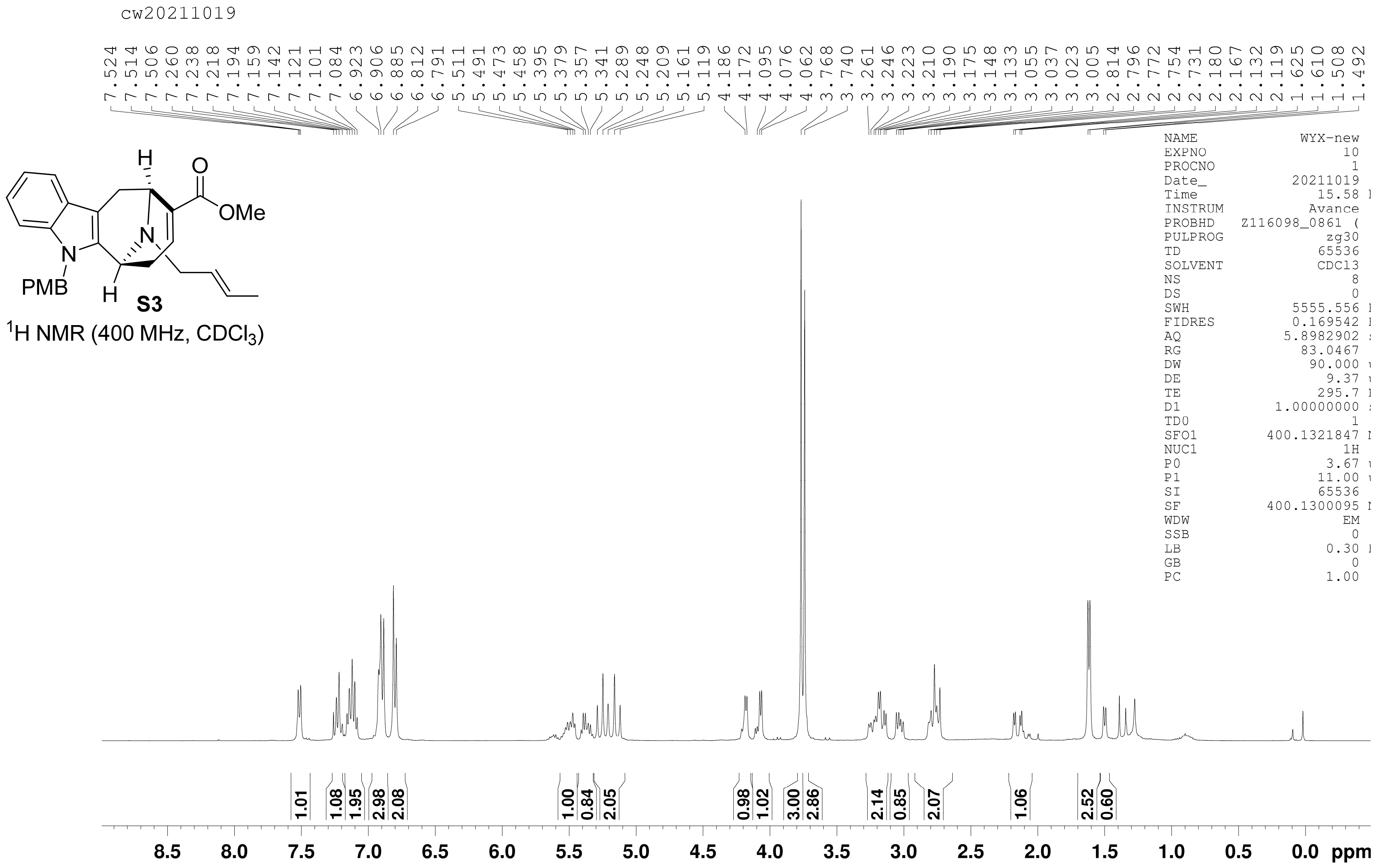

Supplementary Figure 23 ^{1}H-NMR (400 MHz, CDCl$_3$) spectra of **S3**

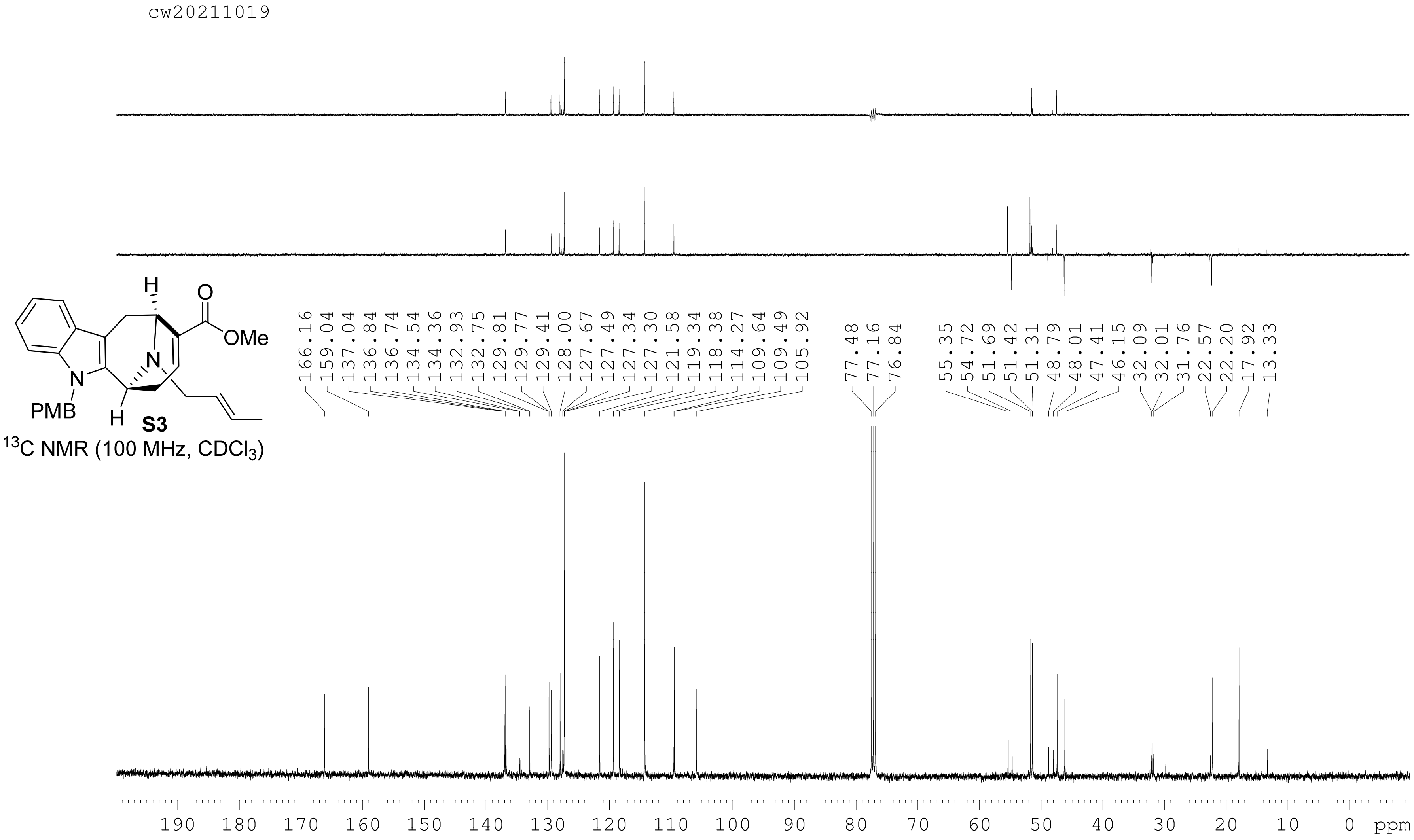

Supplementary Figure 24 ^{13}C-NMR (100 MHz, CDCl$_3$) spectra of **S3**

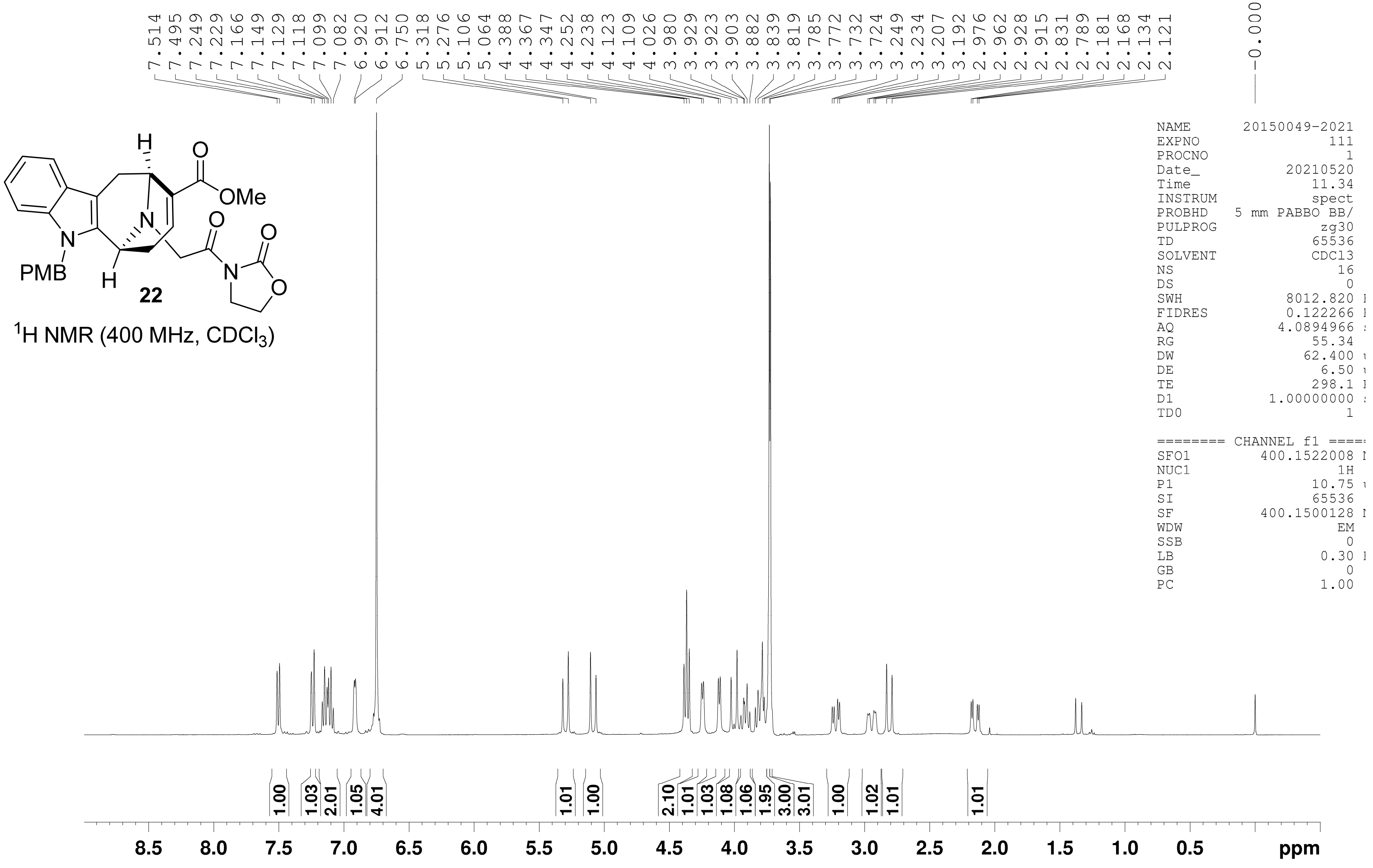

Supplementary Figure 25 ^{1}H-NMR (400 MHz, $CDCl_3$) spectra of **22**

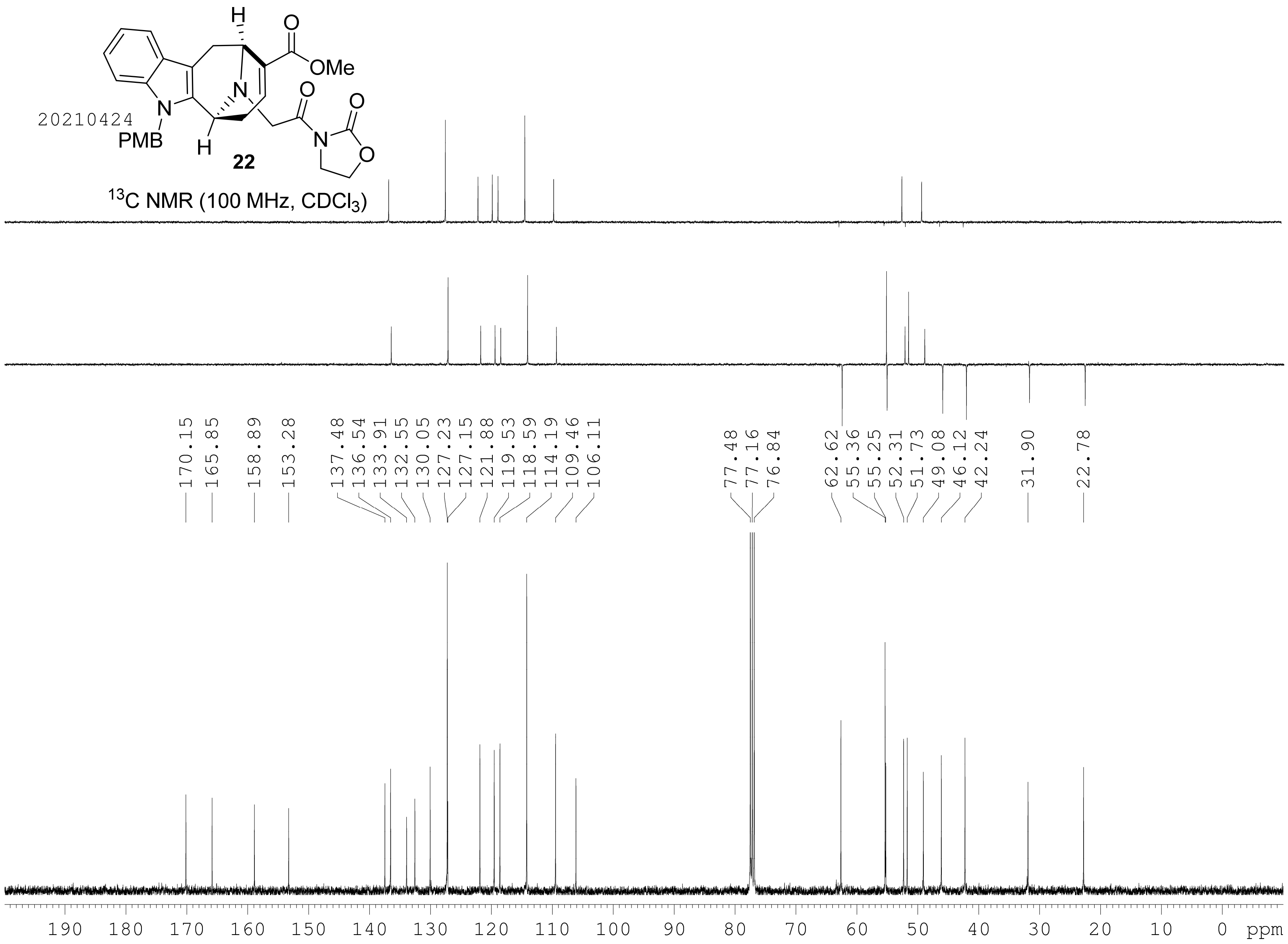

Supplementary Figure 26 ^{13}C-NMR (100 MHz, CDCl$_3$) spectra of **22**

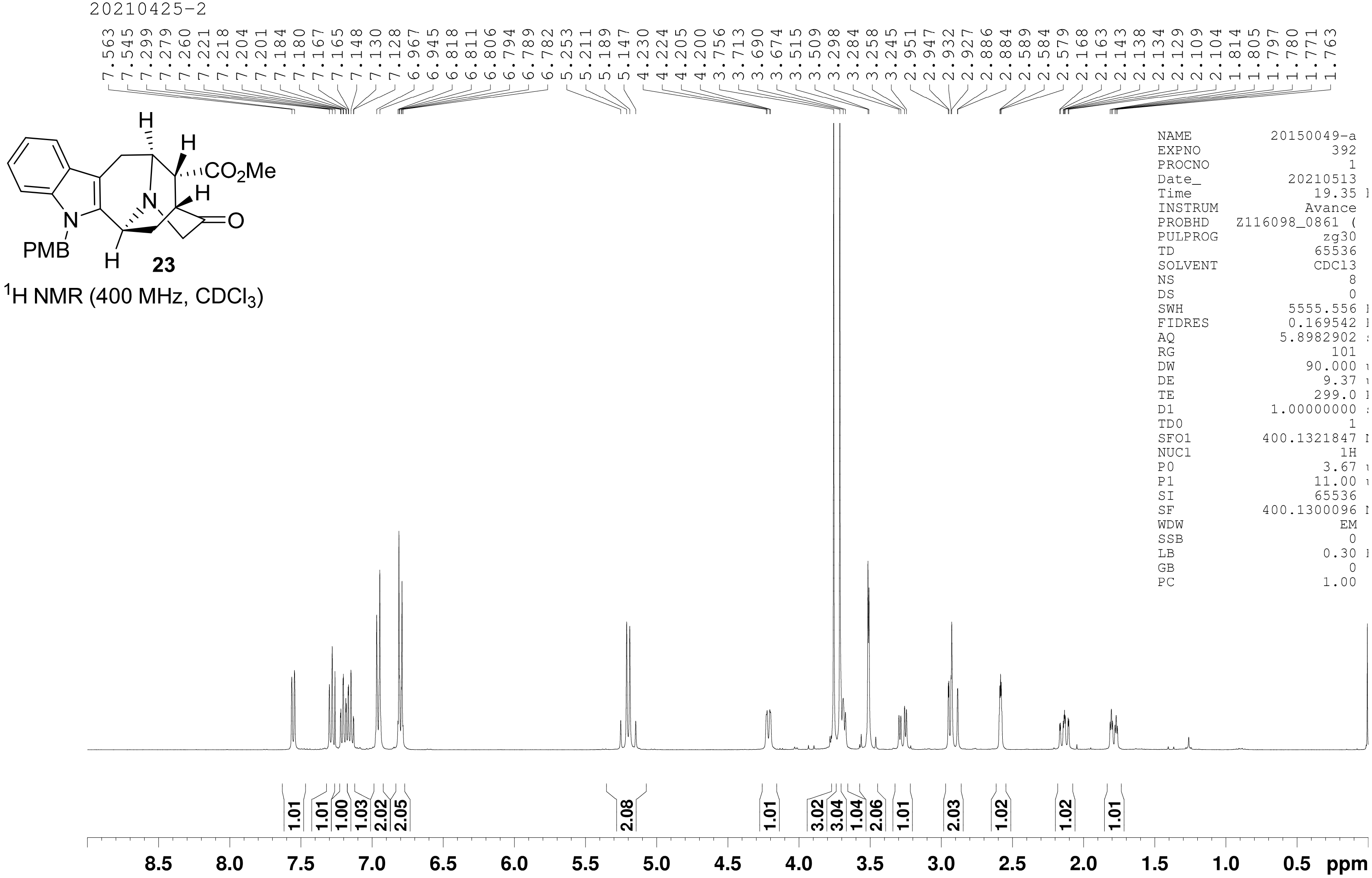

Supplementary Figure 27 ^{1}H-NMR (400 MHz, $CDCl_3$) spectra of **23**

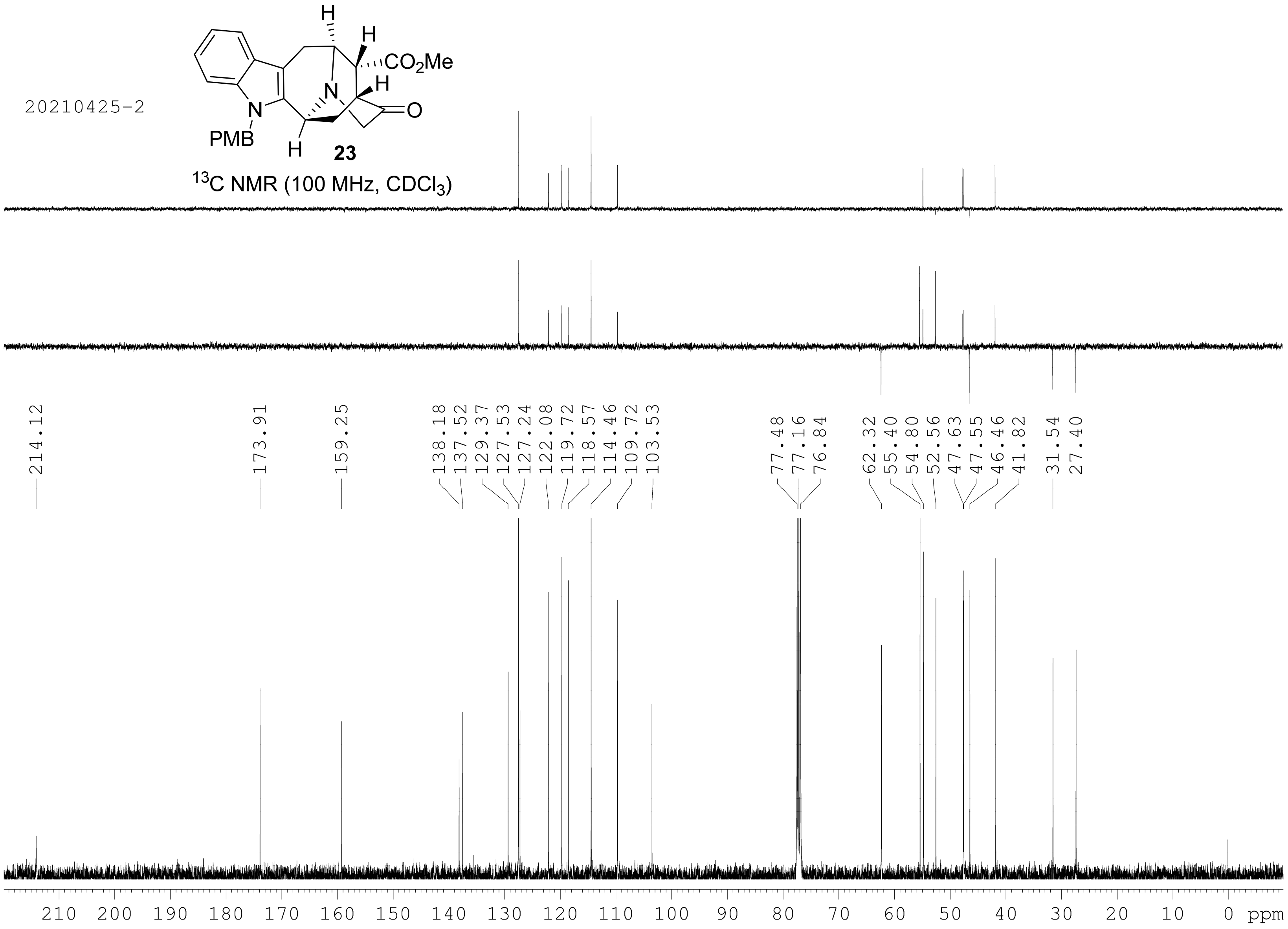

Supplementary Figure 28 ^{13}C-NMR (100 MHz, $CDCl_3$) spectra of **23**

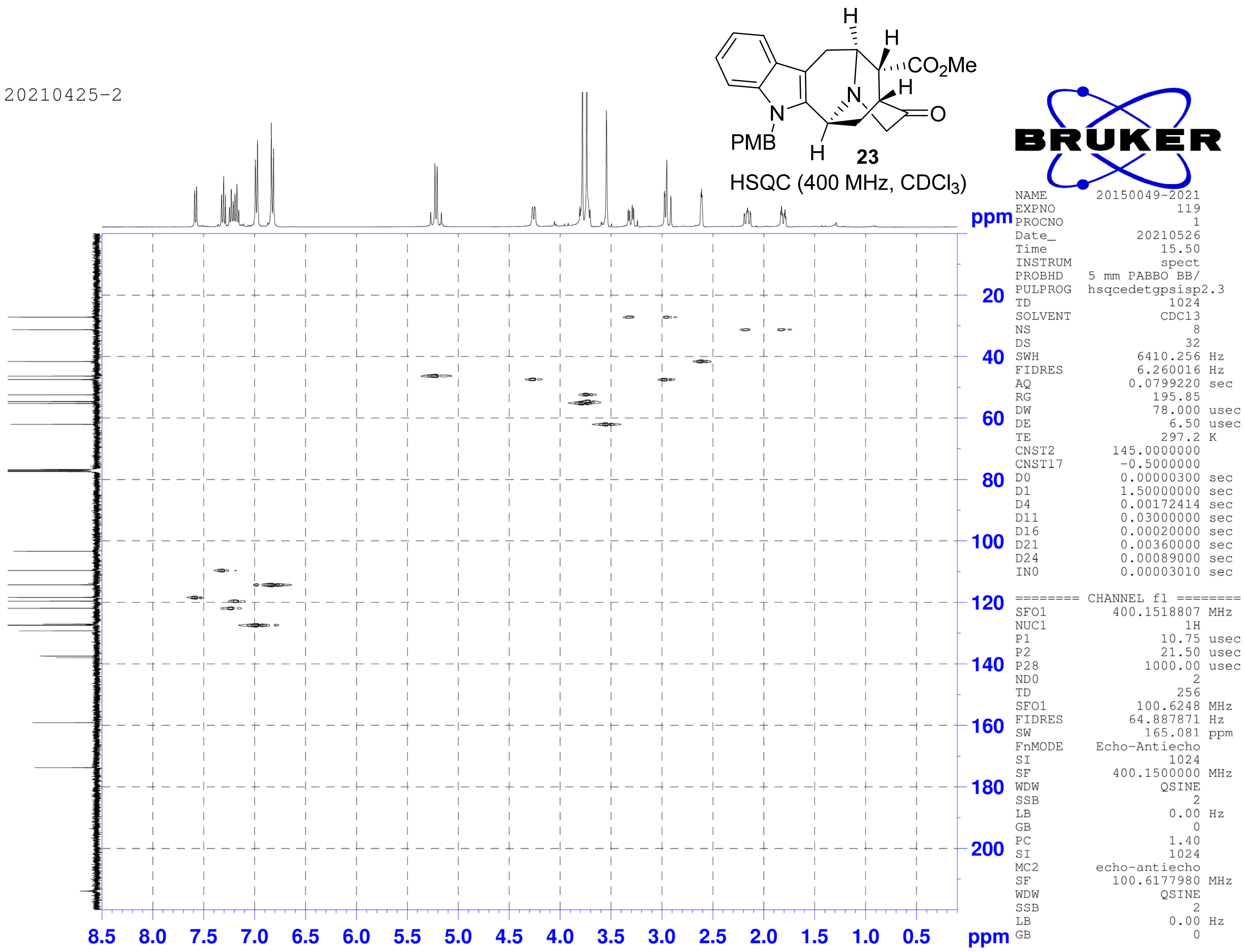

Supplementary Figure 29 HSQC (400 MHz, CDCl$_3$) spectra of **23**

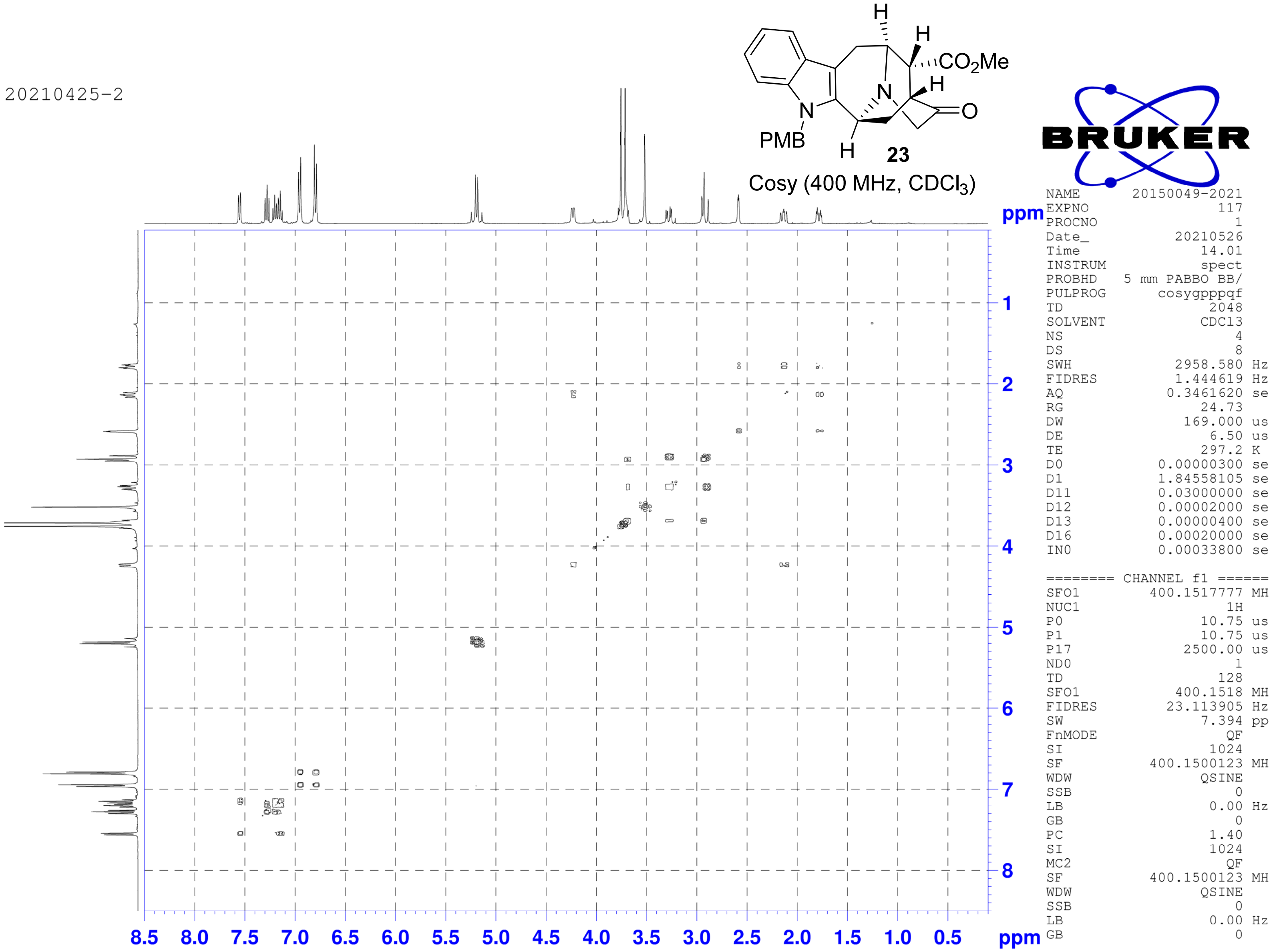

Supplementary Figure 30 Cosy (400 MHz, CDCl3) spectra of **23**

20210425-2

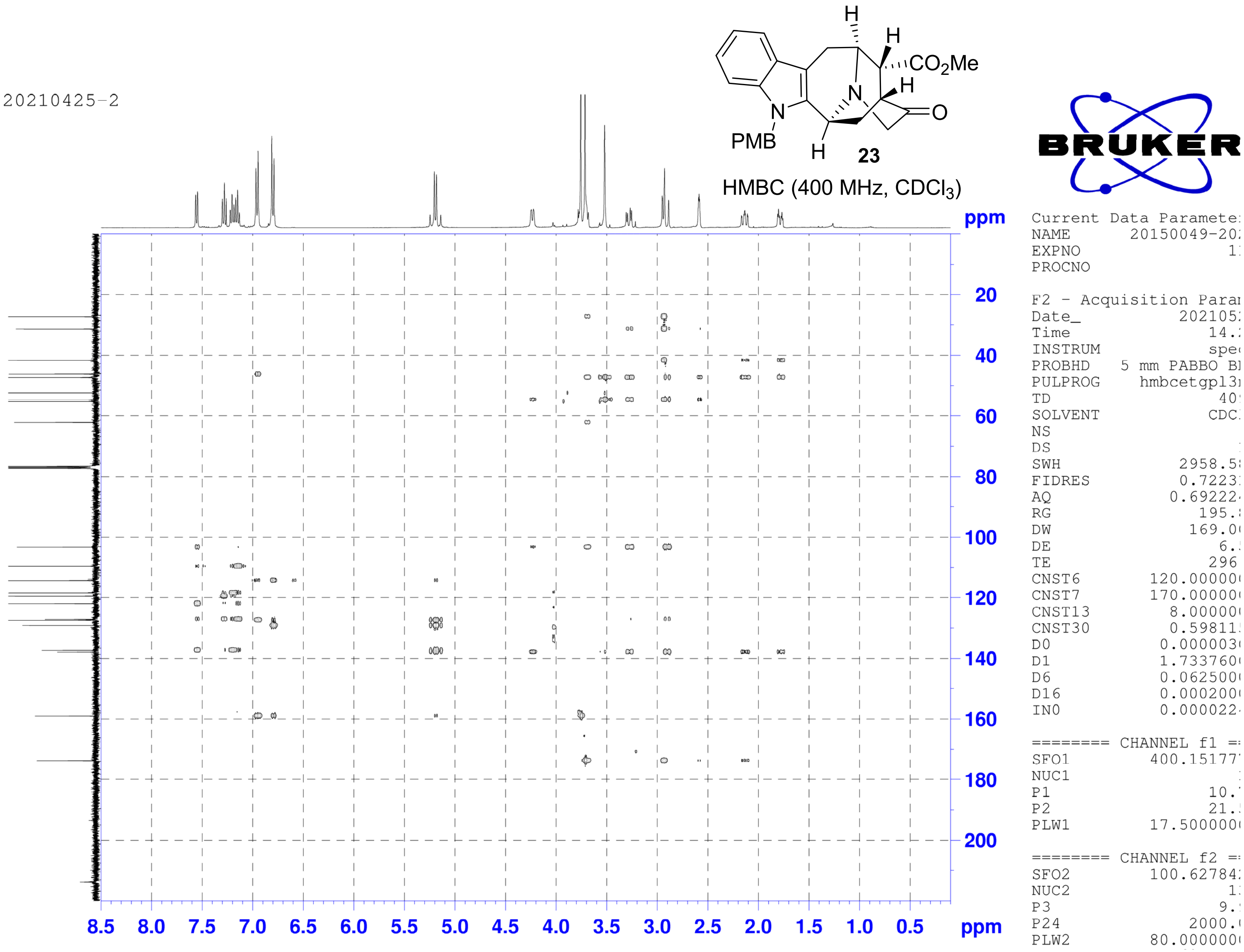

Supplementary Figure 31 HMBC (400 MHz, $CDCl_3$) spectra of **23**

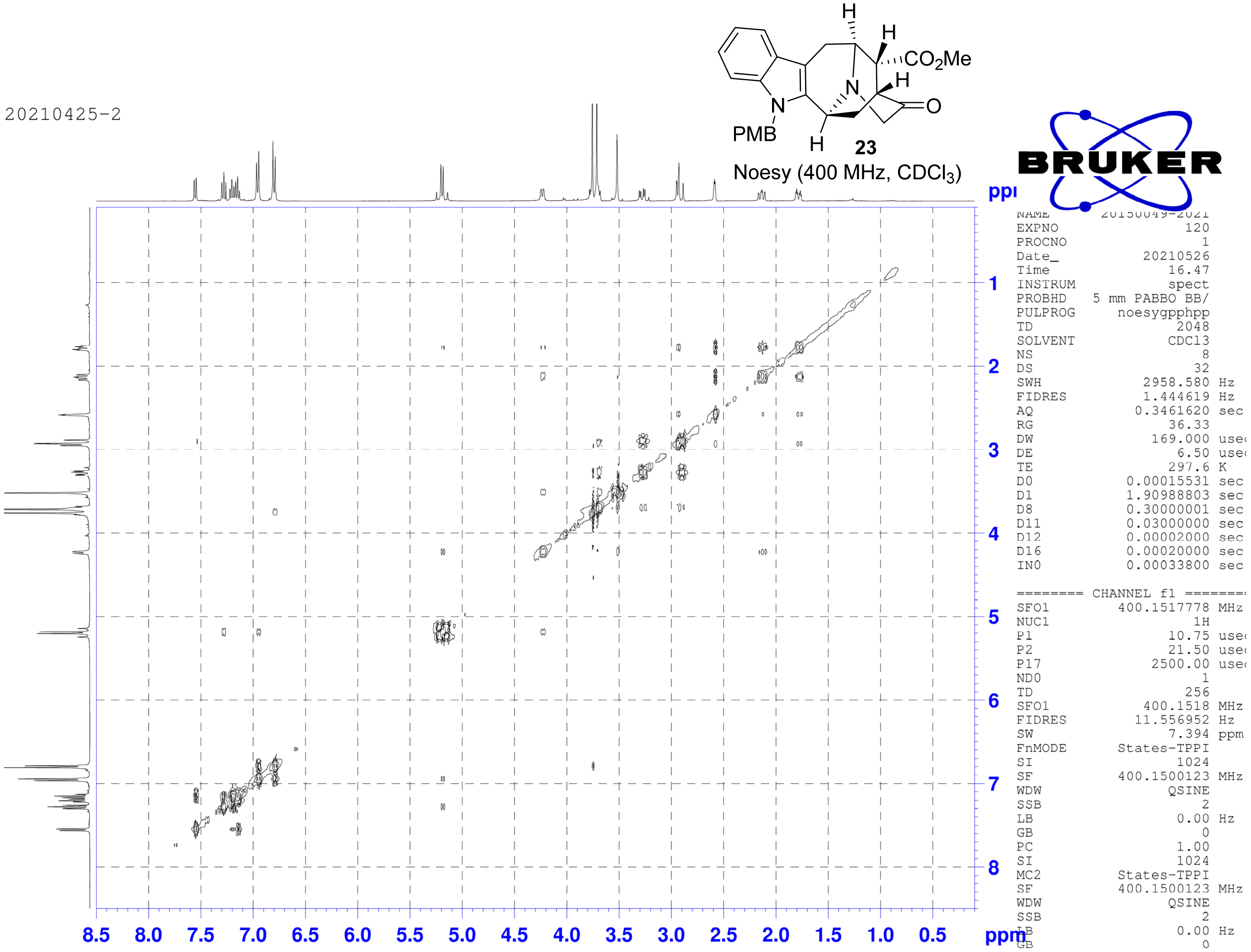

Supplementary Figure 32 Noesy (400 MHz, $CDCl_3$) spectra of **23**

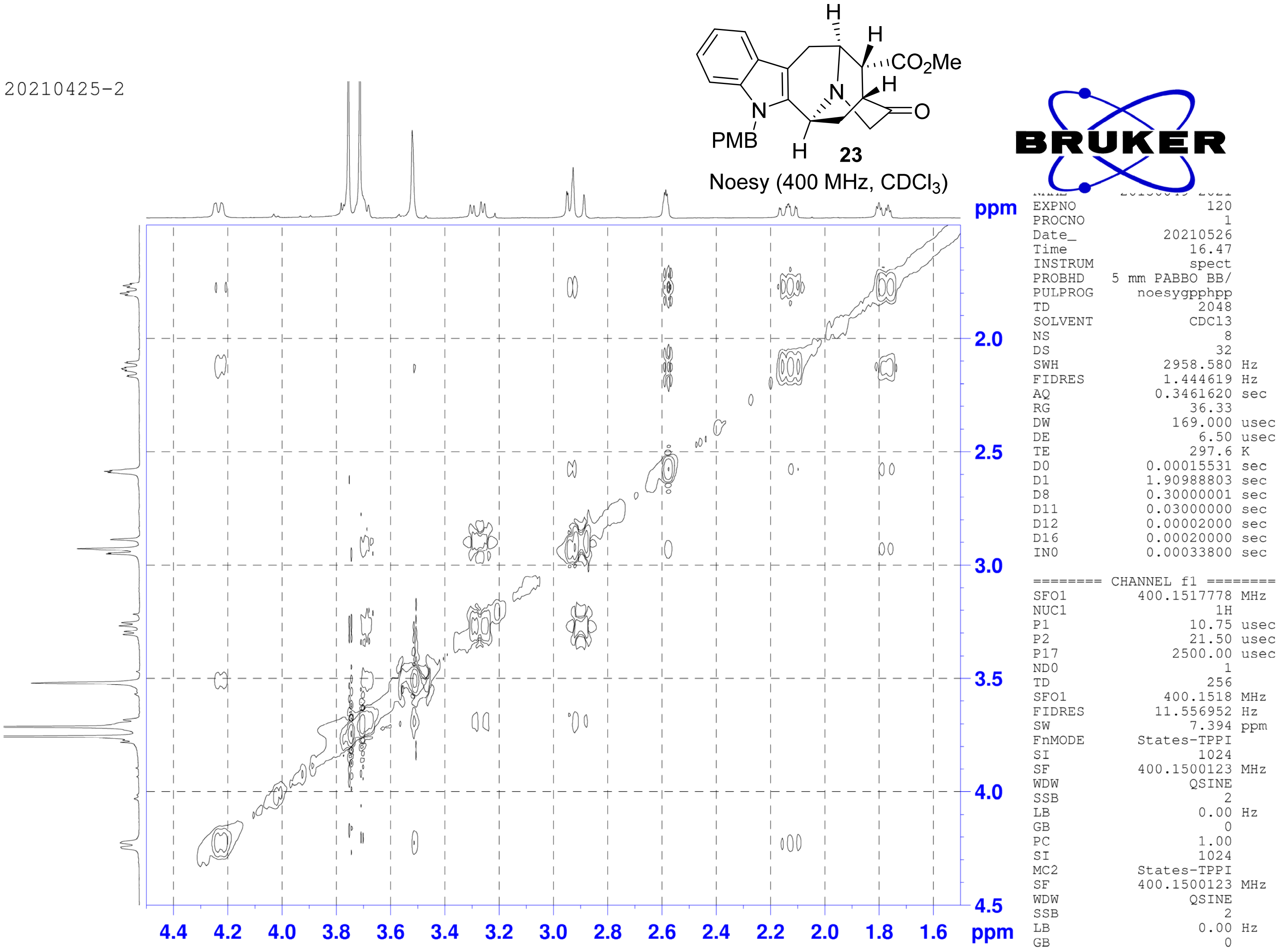

Supplementary Figure 33 Noesy (400 MHz, $CDCl_3$) spectra (δ: 1.5 to 4.5 ppm) of **23**

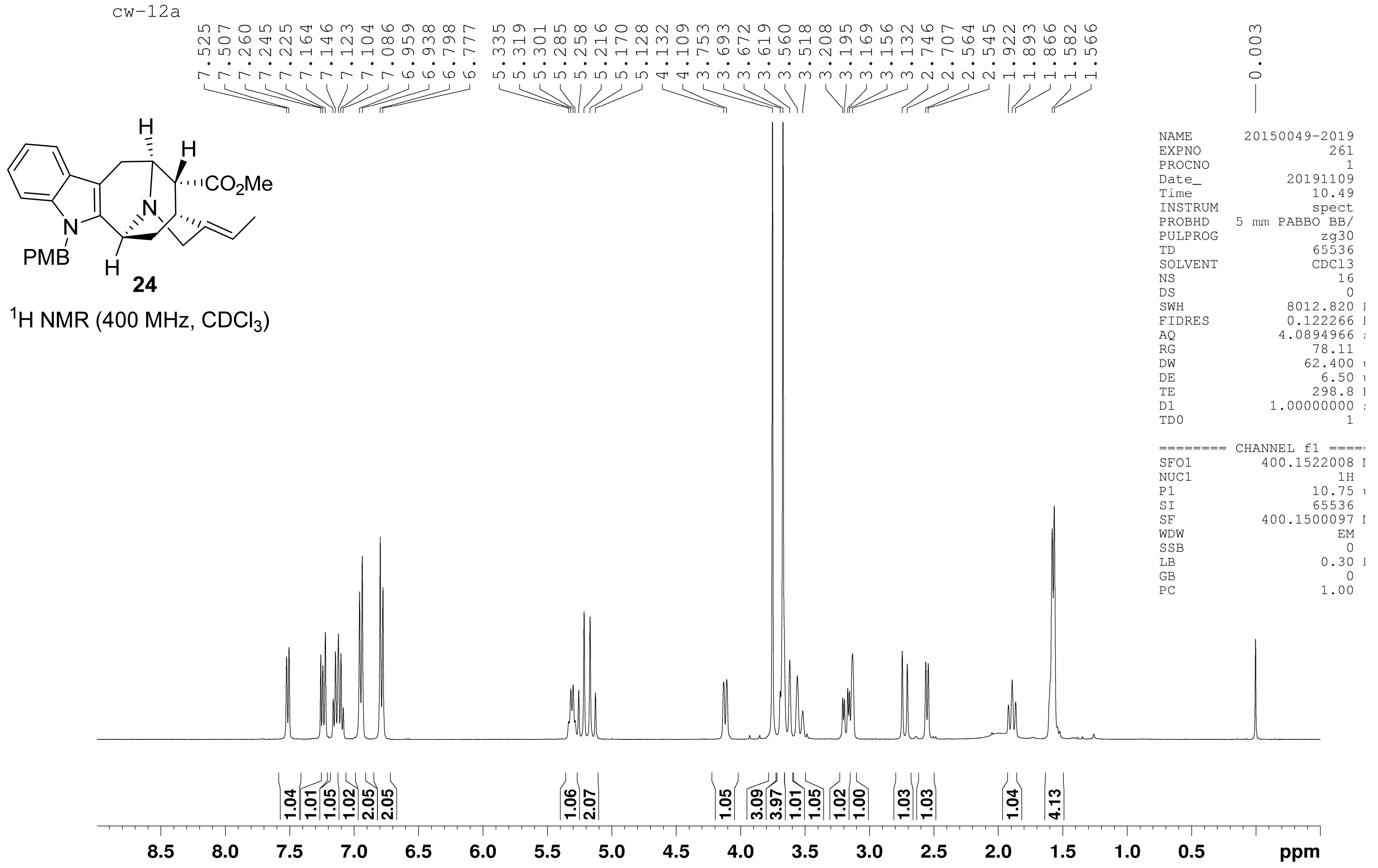

Supplementary Figure 34 ^{1}H-NMR (400 MHz, $CDCl_3$) spectra of **24**

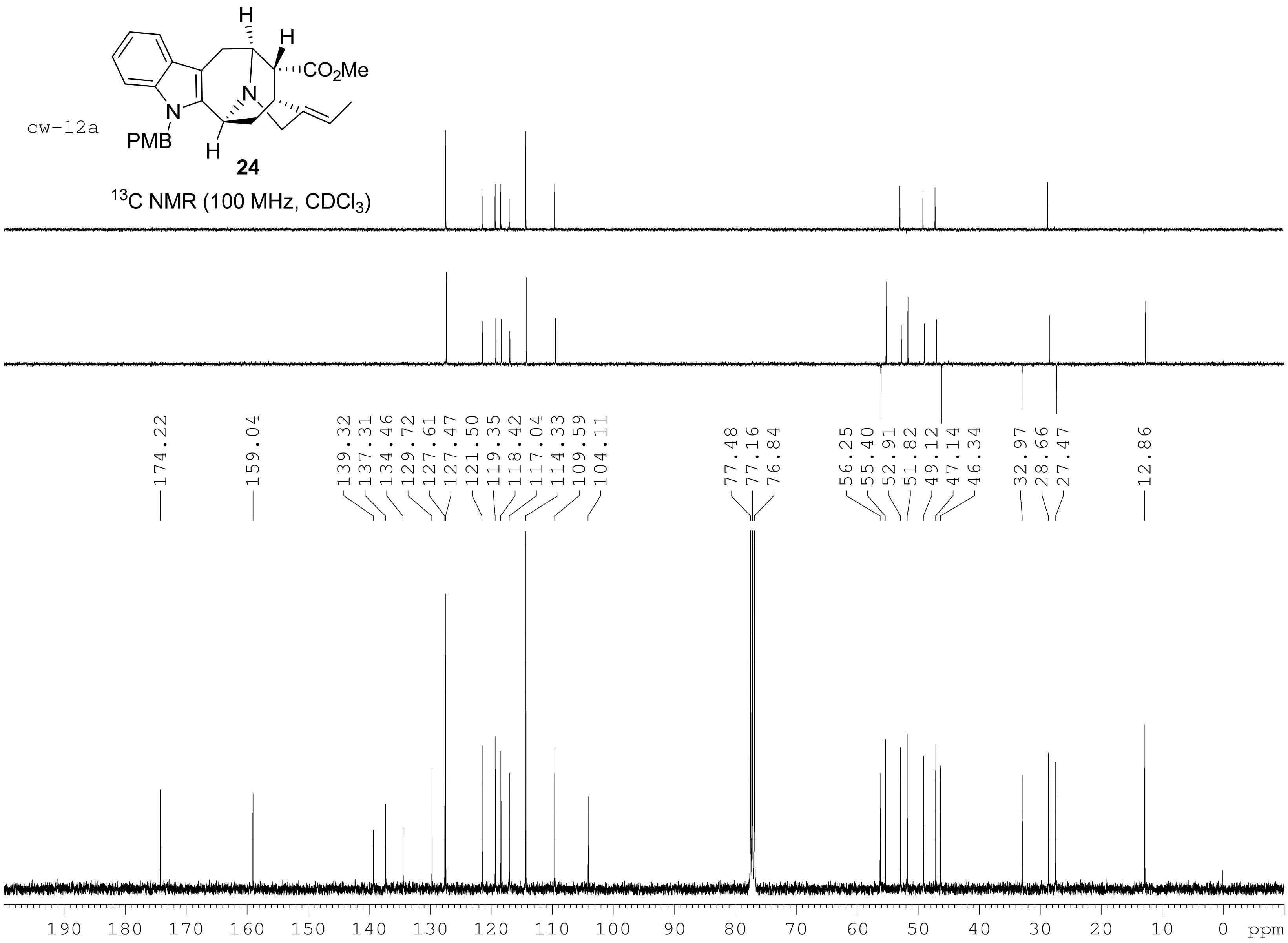

Supplementary Figure 35 ^{13}C-NMR (100 MHz, $CDCl_3$) spectra of **24**

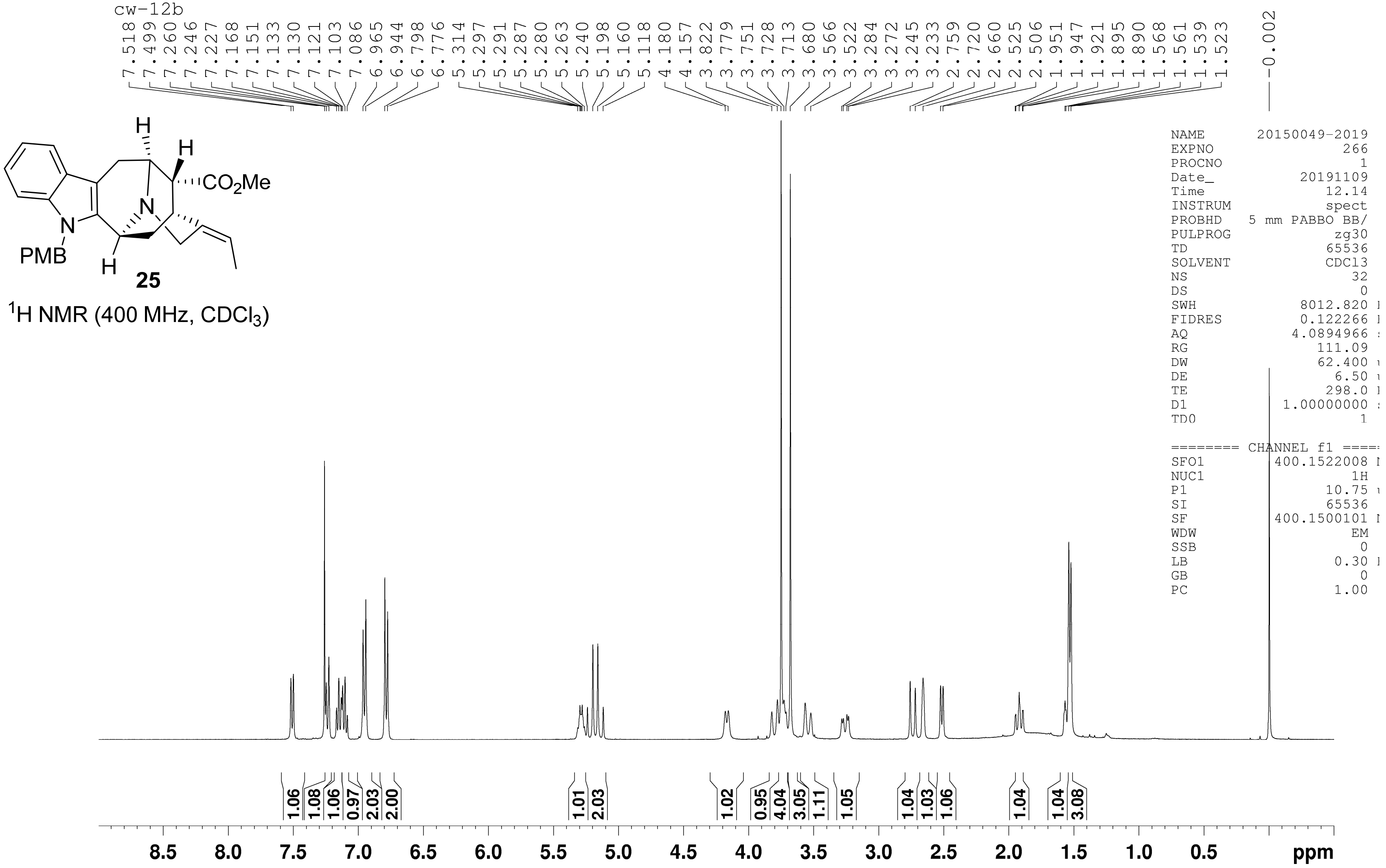

Supplementary Figure 36 ^{1}H-NMR (400 MHz, $CDCl_3$) spectra of **25**

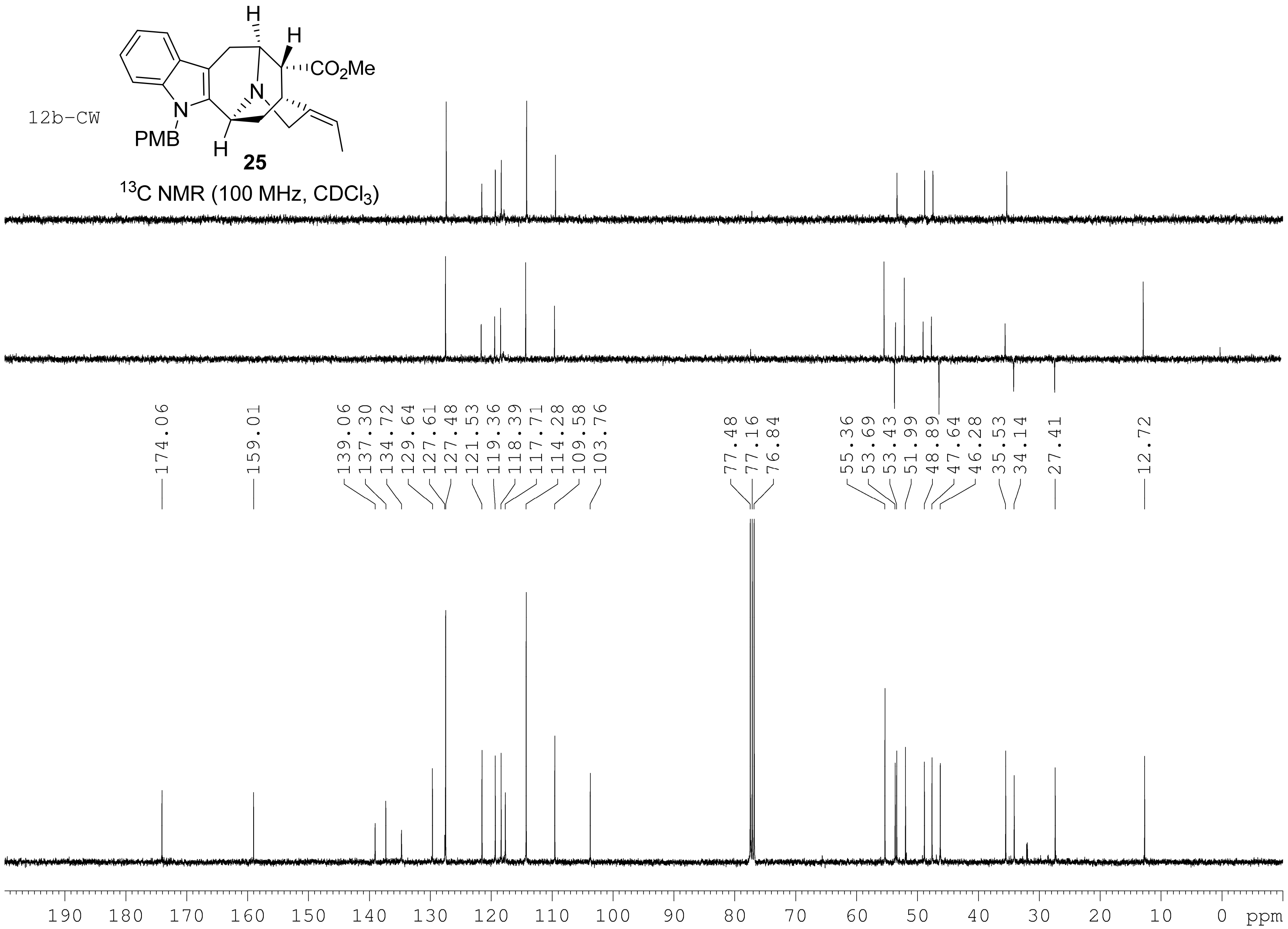

Supplementary Figure 37 ^{13}C-NMR (100 MHz, $CDCl_3$) spectra of **25**

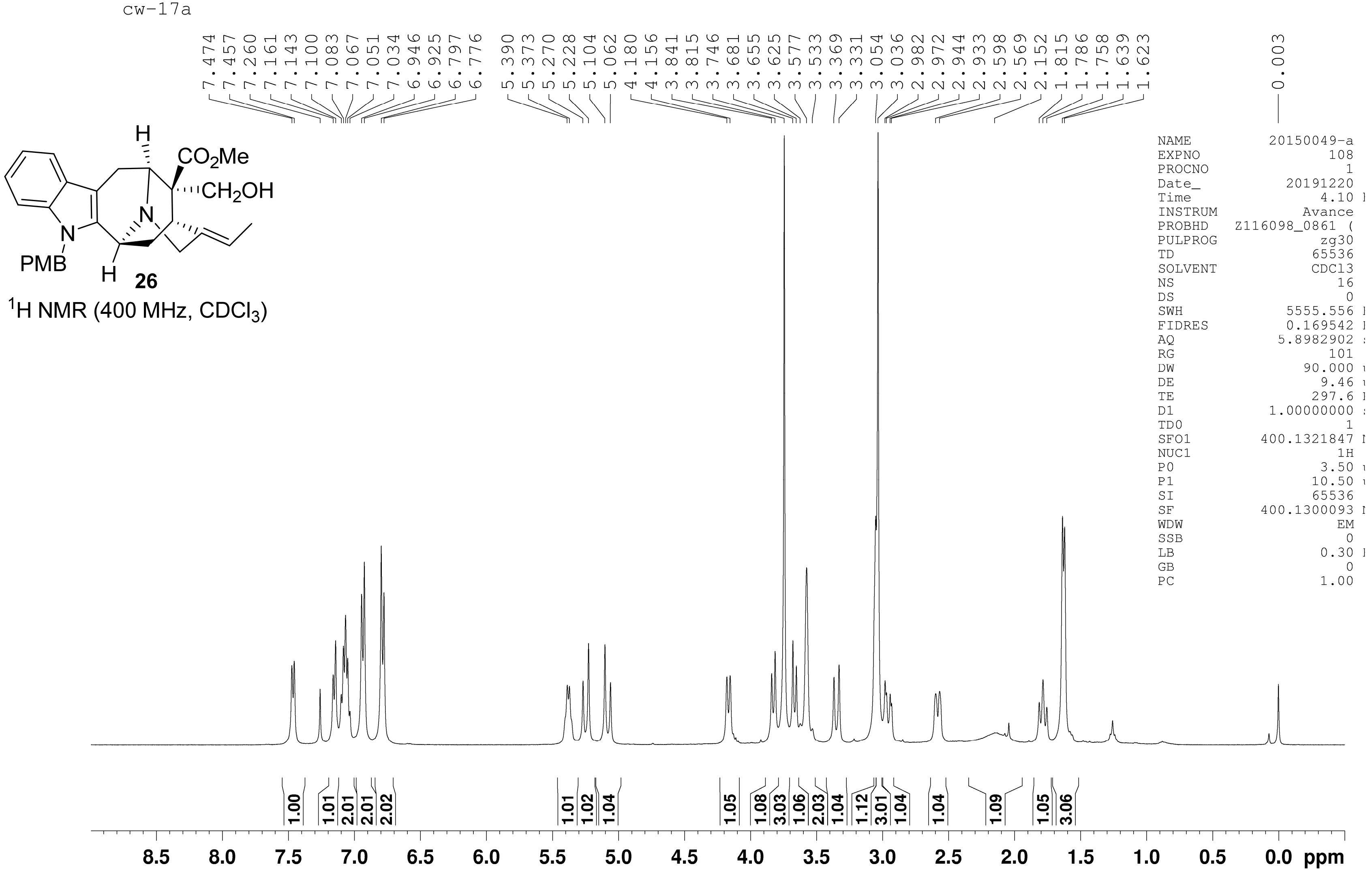

Supplementary Figure 38 ^{1}H-NMR (400 MHz, $CDCl_3$) spectra of **26**

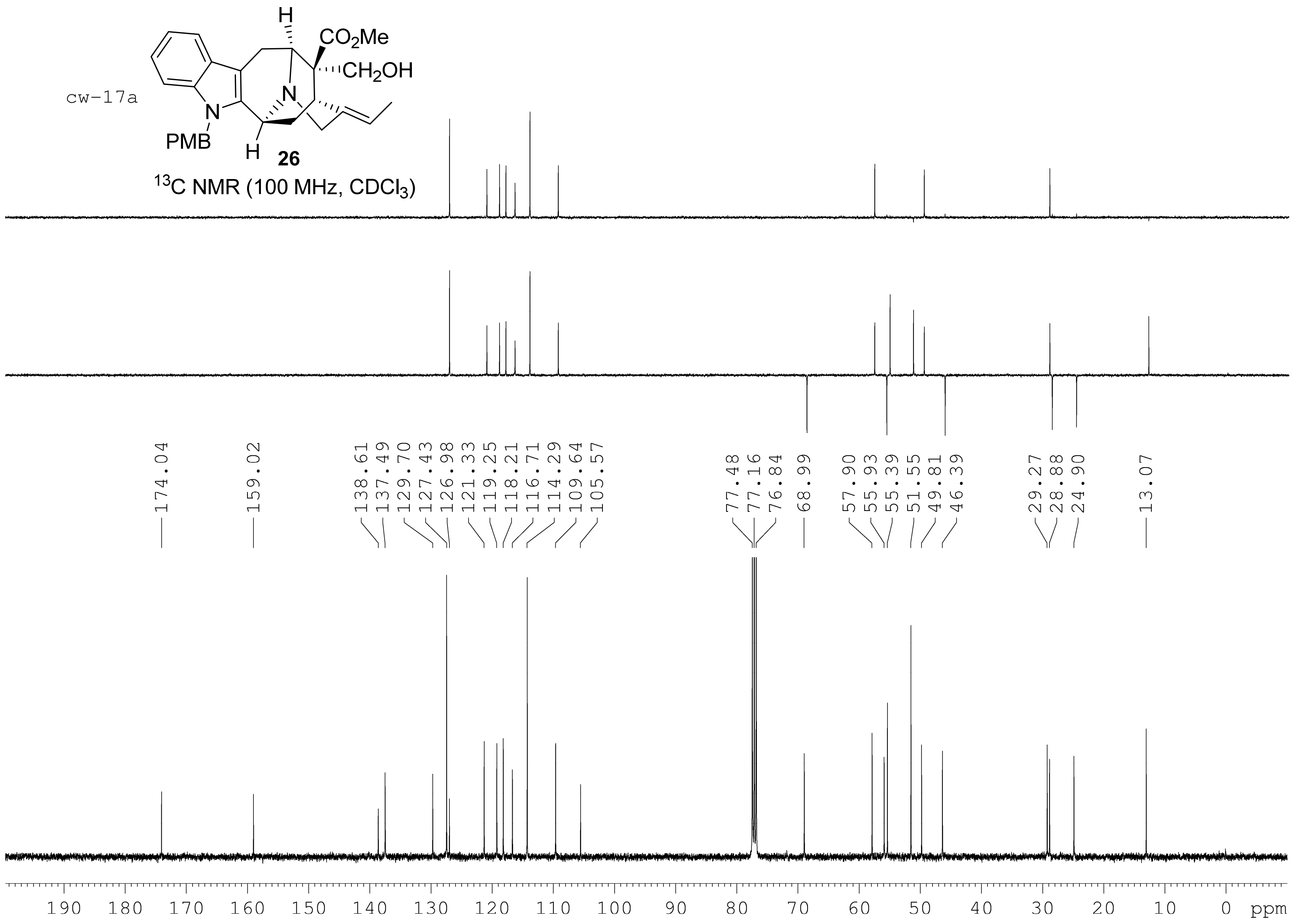

Supplementary Figure 39 ^{13}C-NMR (100 MHz, $CDCl_3$) spectra of **26**

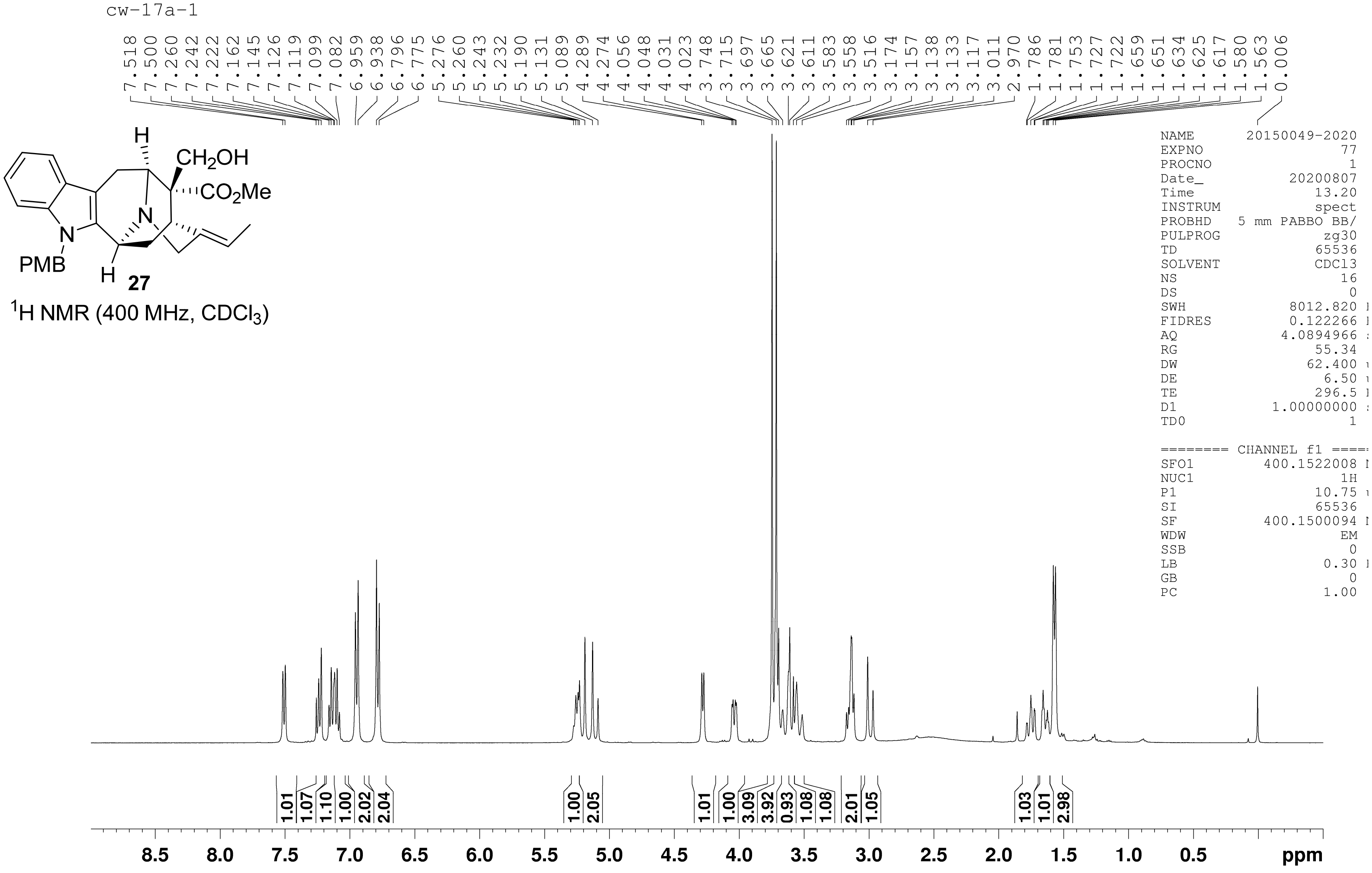

Supplementary Figure 40 ^{1}H-NMR (400 MHz, $CDCl_3$) spectra of **27**

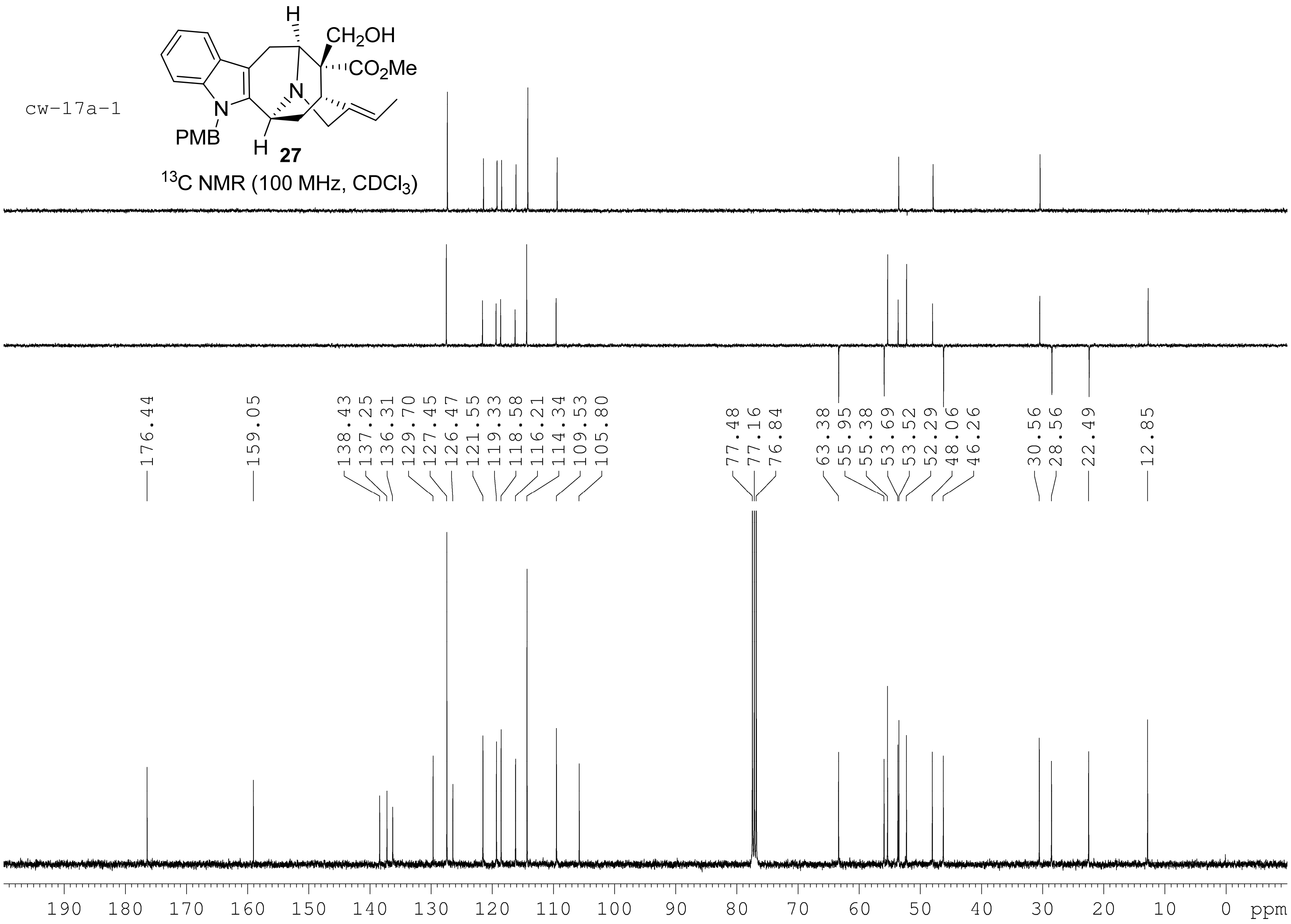

Supplementary Figure 41 ^{13}C-NMR (100 MHz, $CDCl_3$) spectra of **27**

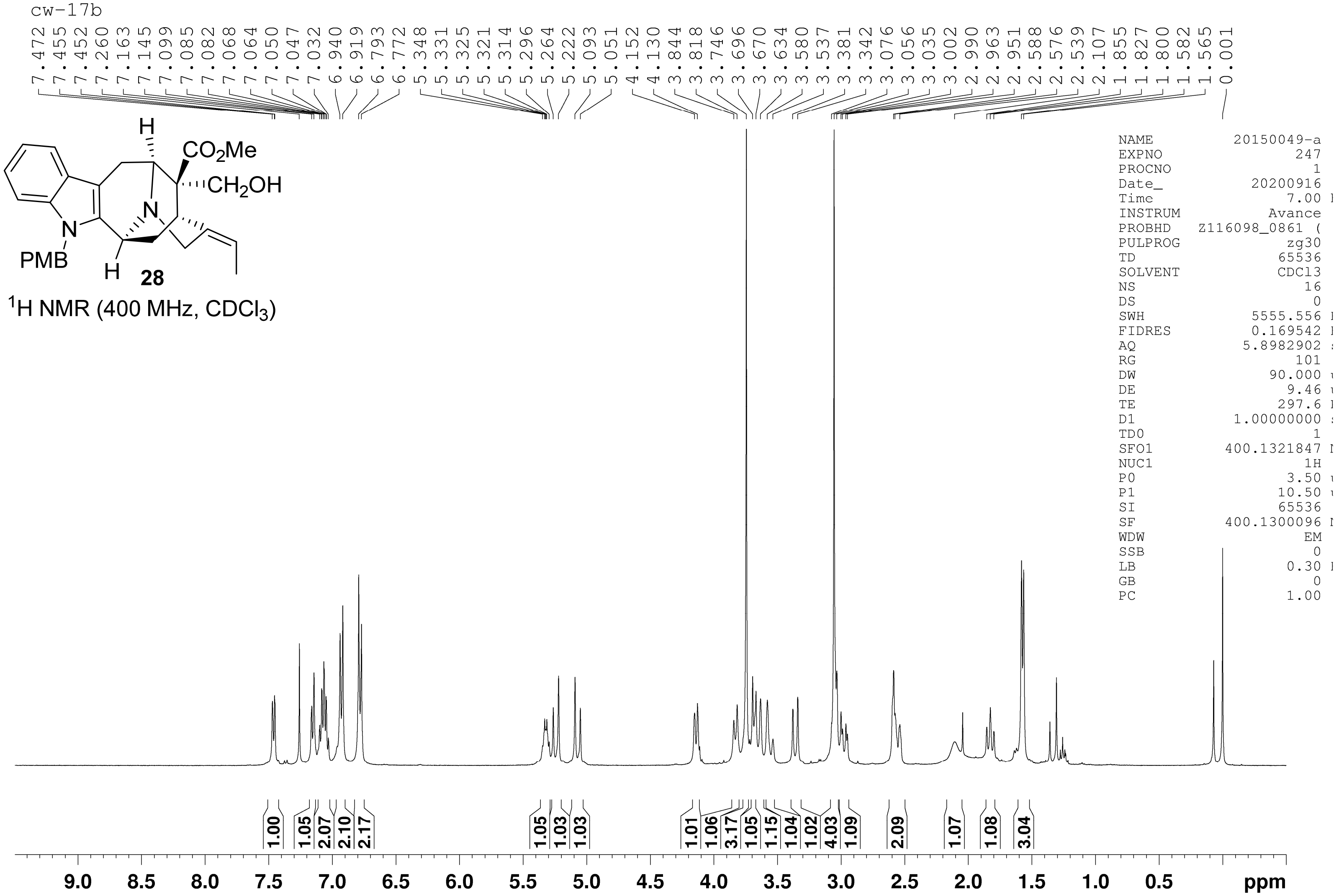

Supplementary Figure 42 ^{1}H-NMR (400 MHz, $CDCl_3$) spectra of **28**

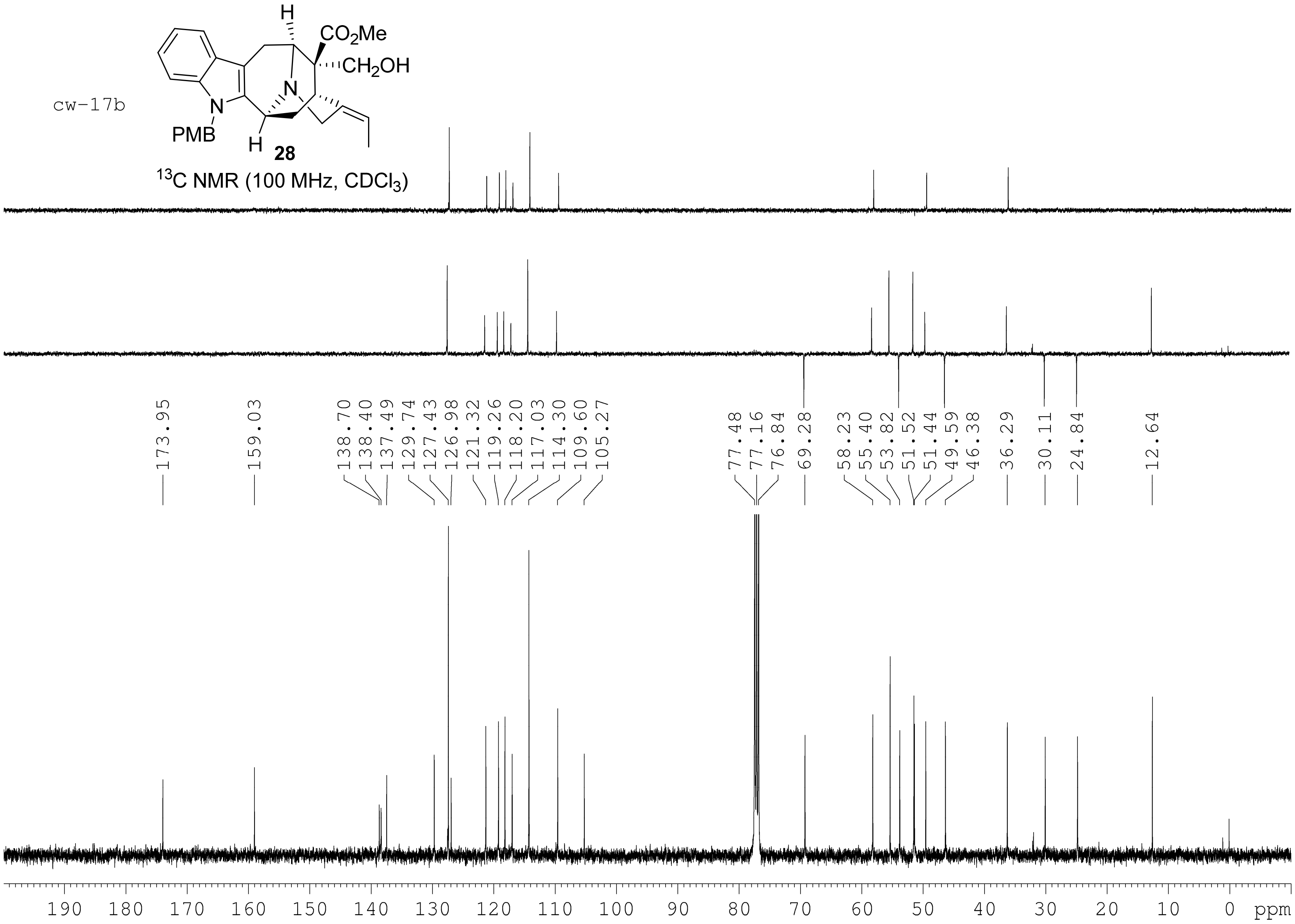

Supplementary Figure 43 ^{13}C-NMR (100 MHz, CDCl$_3$) spectra of **28**

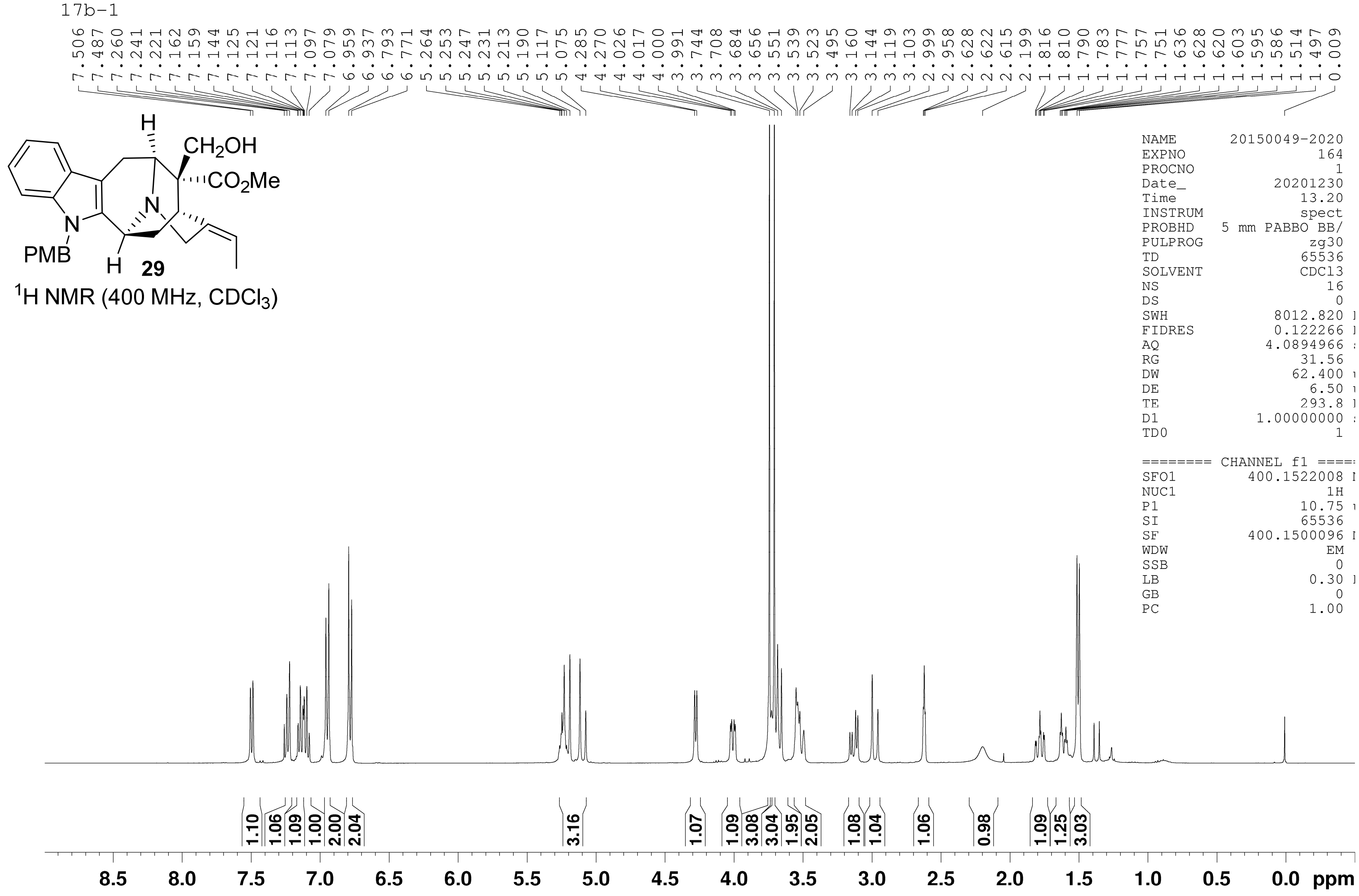

Supplementary Figure 44 ^{1}H-NMR (400 MHz, $CDCl_3$) spectra of **29**

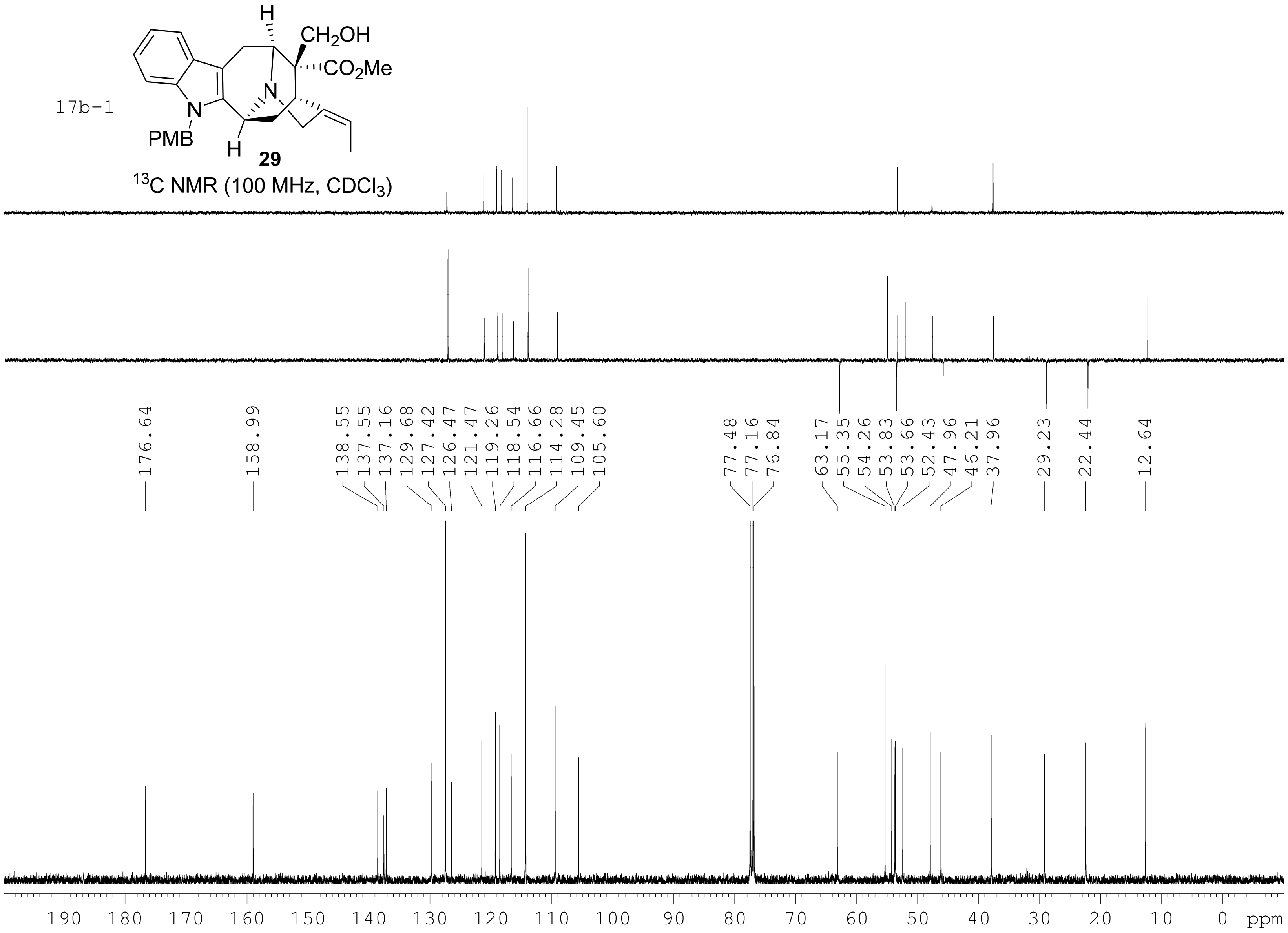

Supplementary Figure 45 ^{13}C-NMR (100 MHz, CDCl$_3$) spectra of **29**

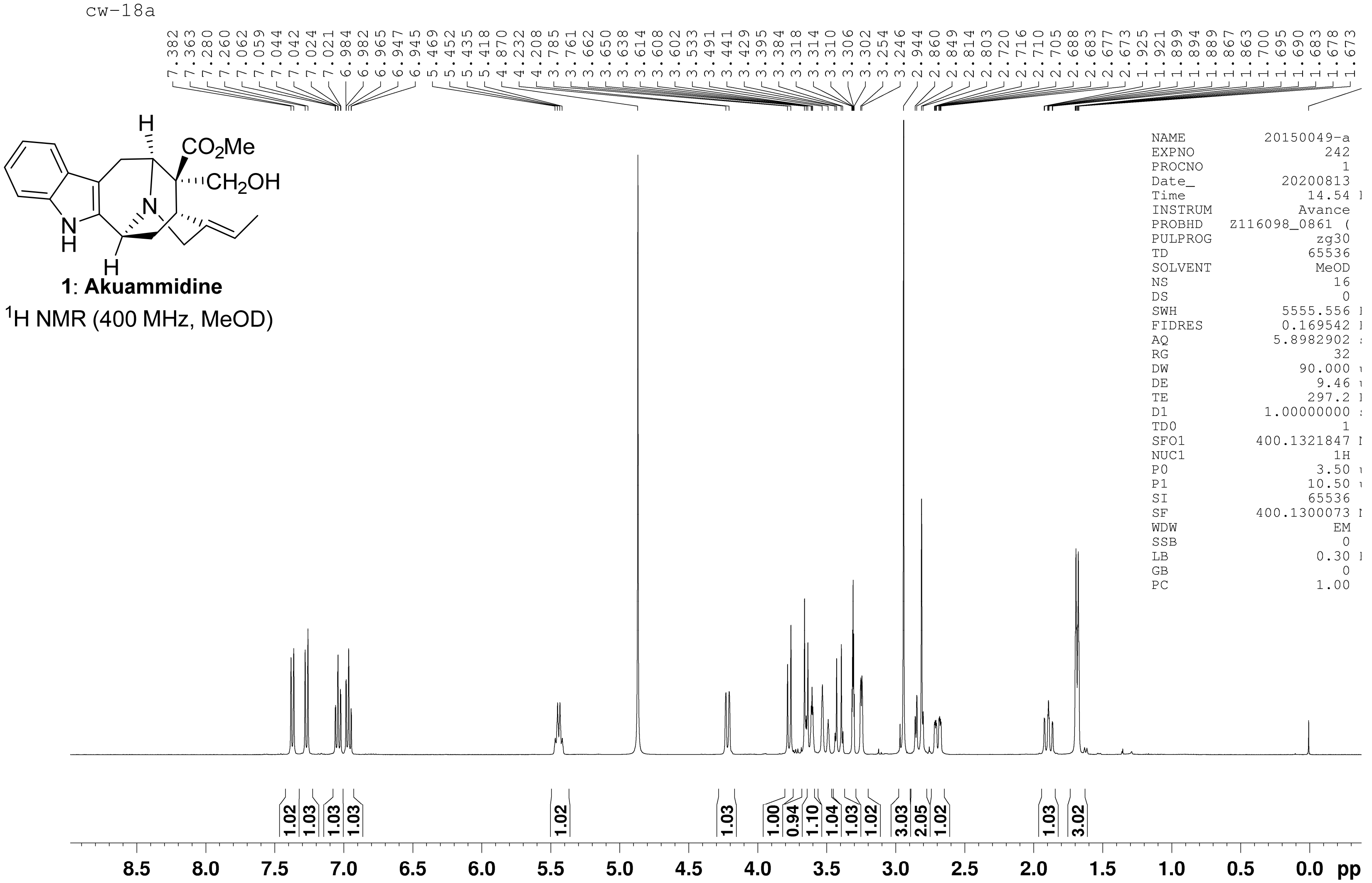

Supplementary Figure 46 ^{1}H-NMR (400 MHz, MeOD) spectra of Akuammidine (**1**)

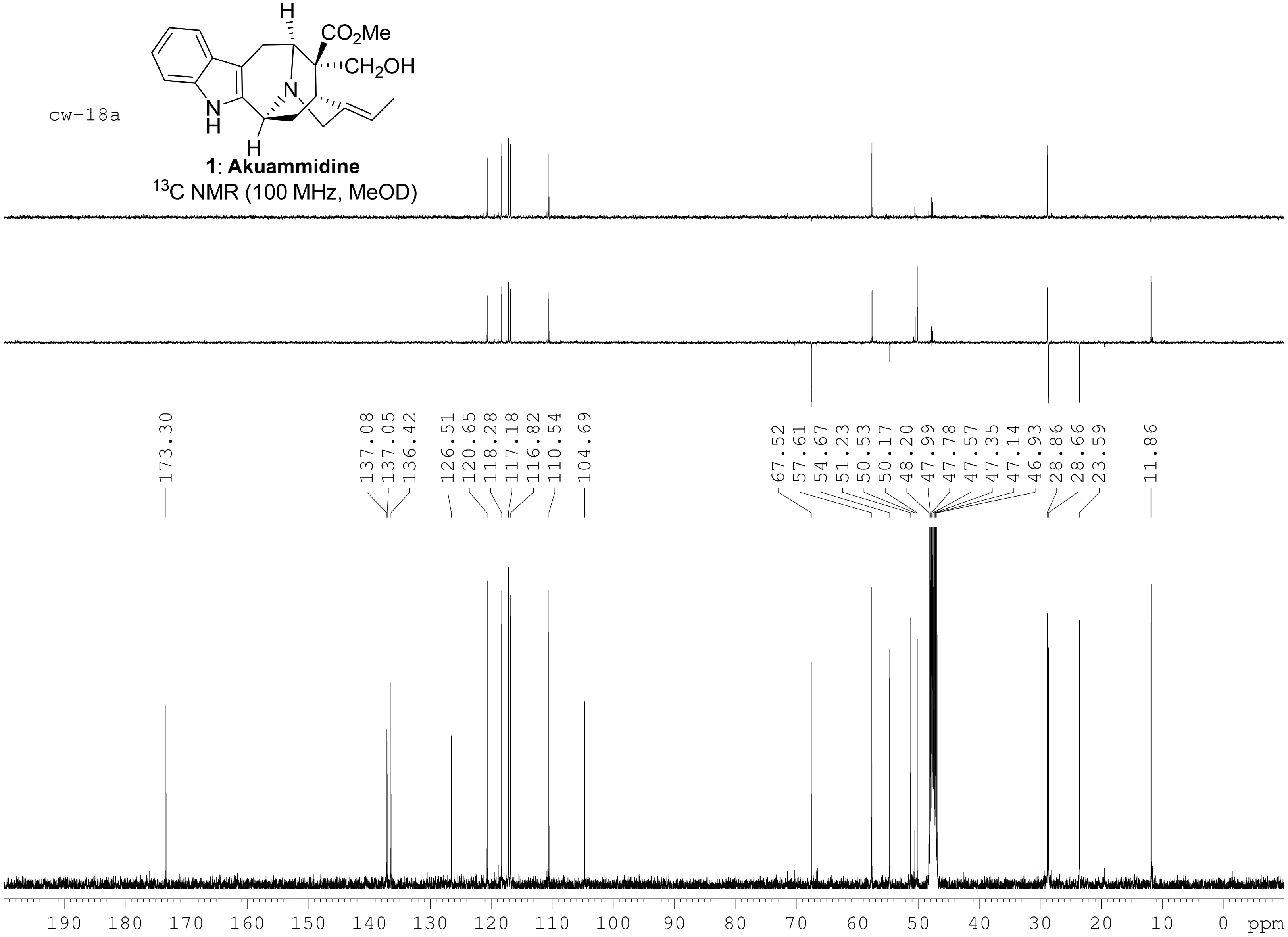

Supplementary Figure 47 ^{13}C-NMR (100 MHz, MeOD) spectra of Akuammidine (**1**)

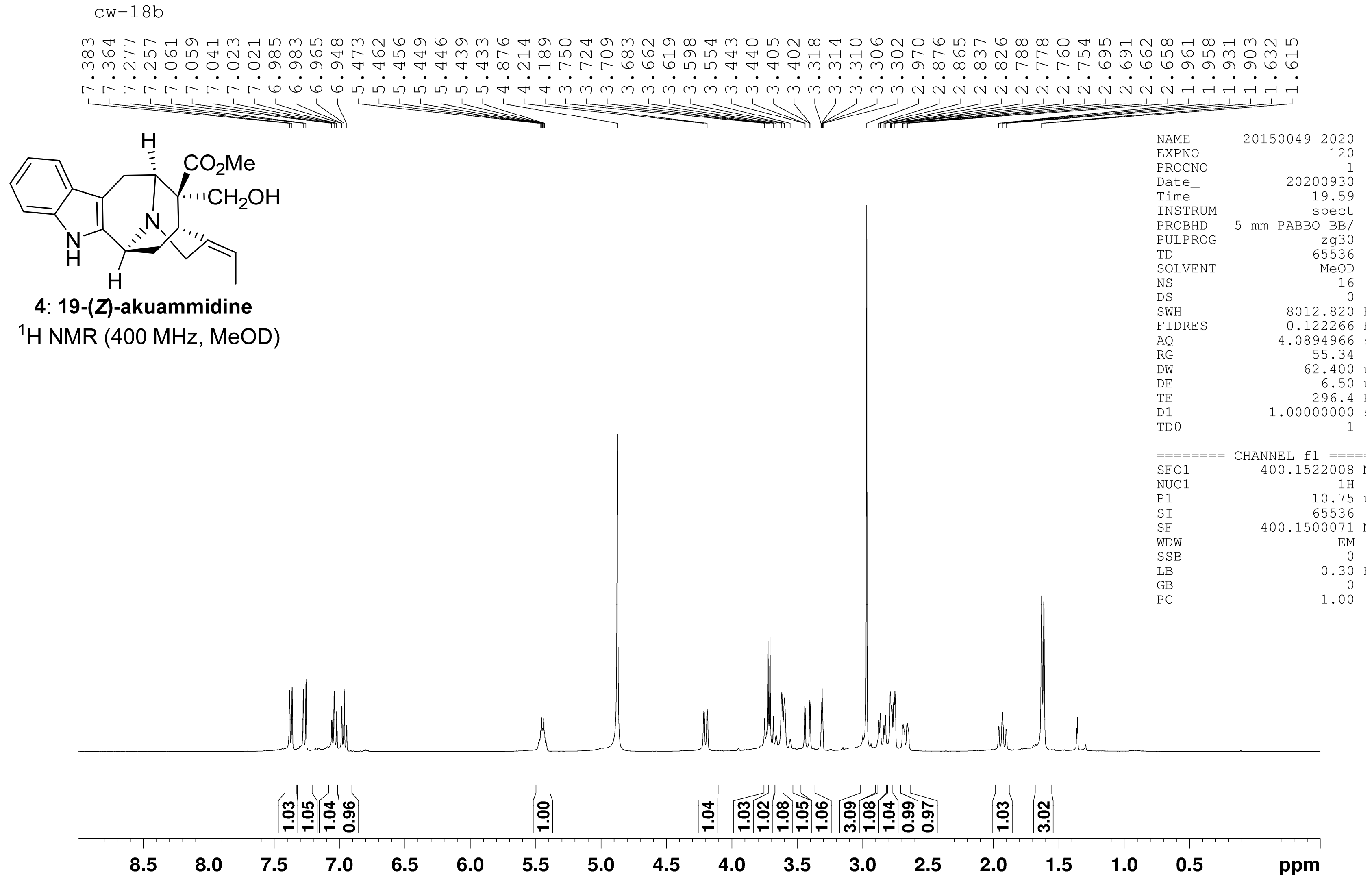

Supplementary Figure 48 ^{1}H-NMR (400 MHz, MeOD) spectra of 19-(*Z*)-Akuammidine (**4**)

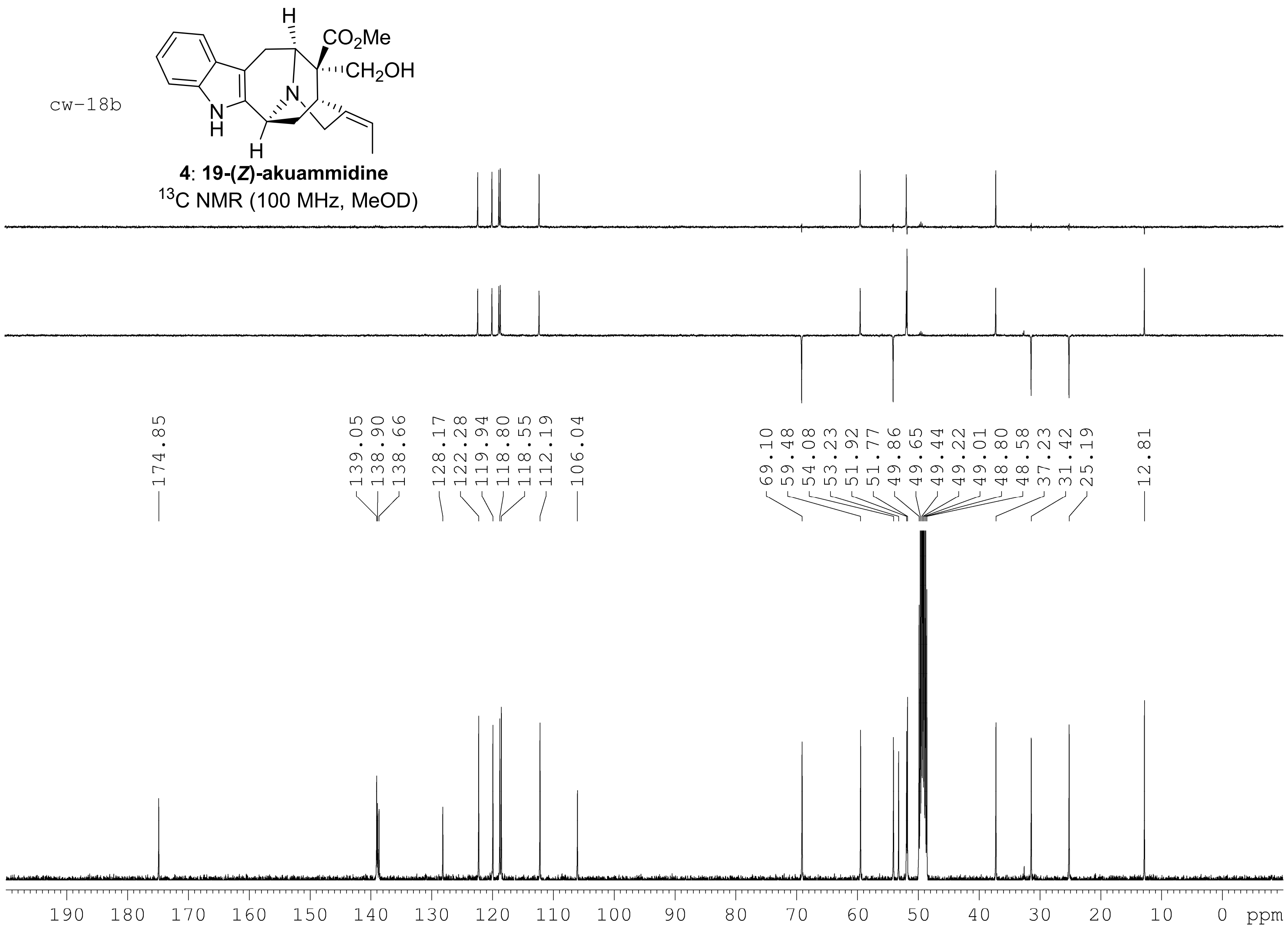

Supplementary Figure 49 ^{13}C-NMR (100 MHz, MeOD) spectra of 19-(*Z*)-Akuammidine (**4**)

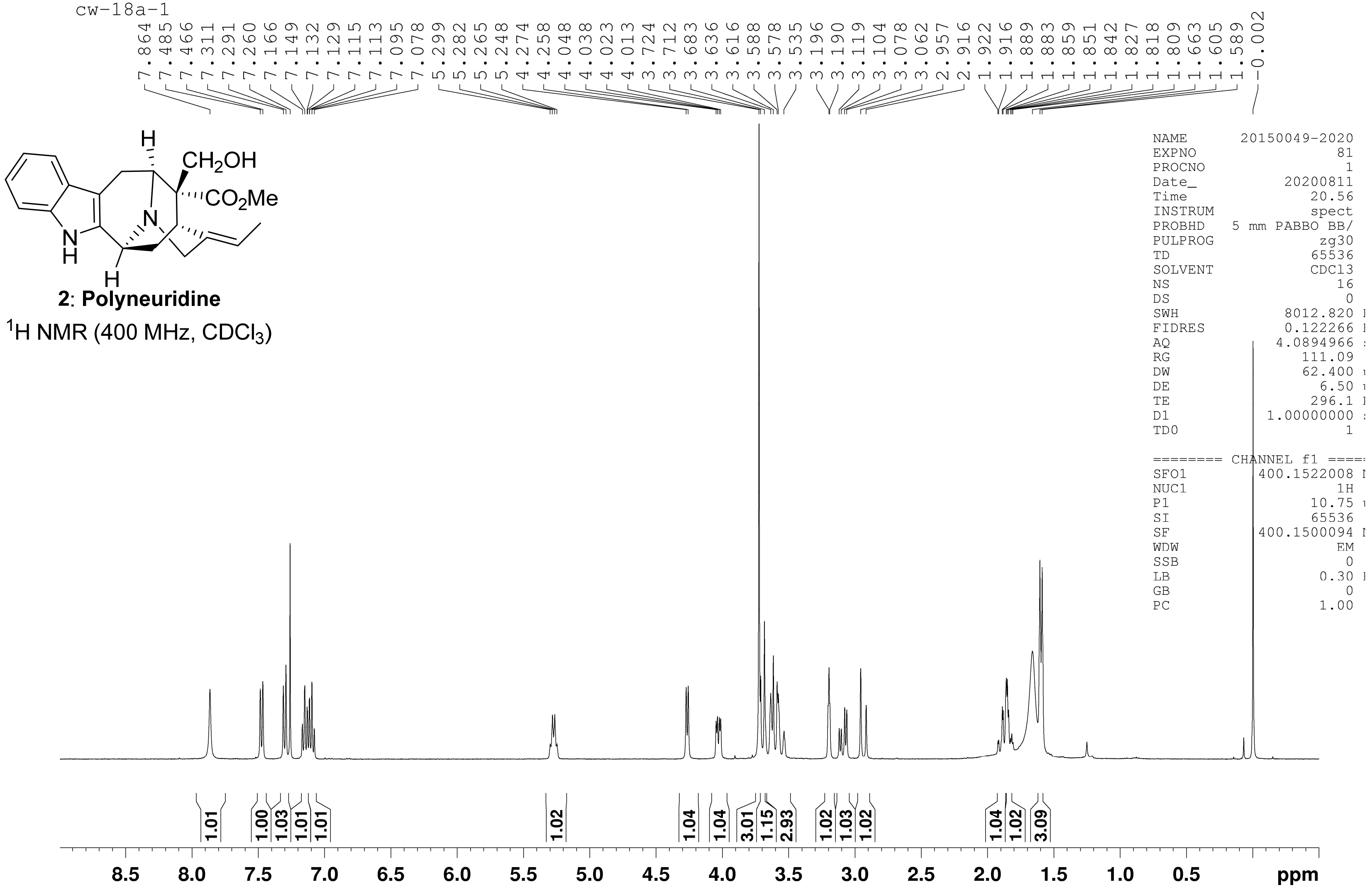

Supplementary Figure 50 ^{1}H-NMR (400 MHz, $CDCl_3$) spectra of Polyneuridine (**2**)

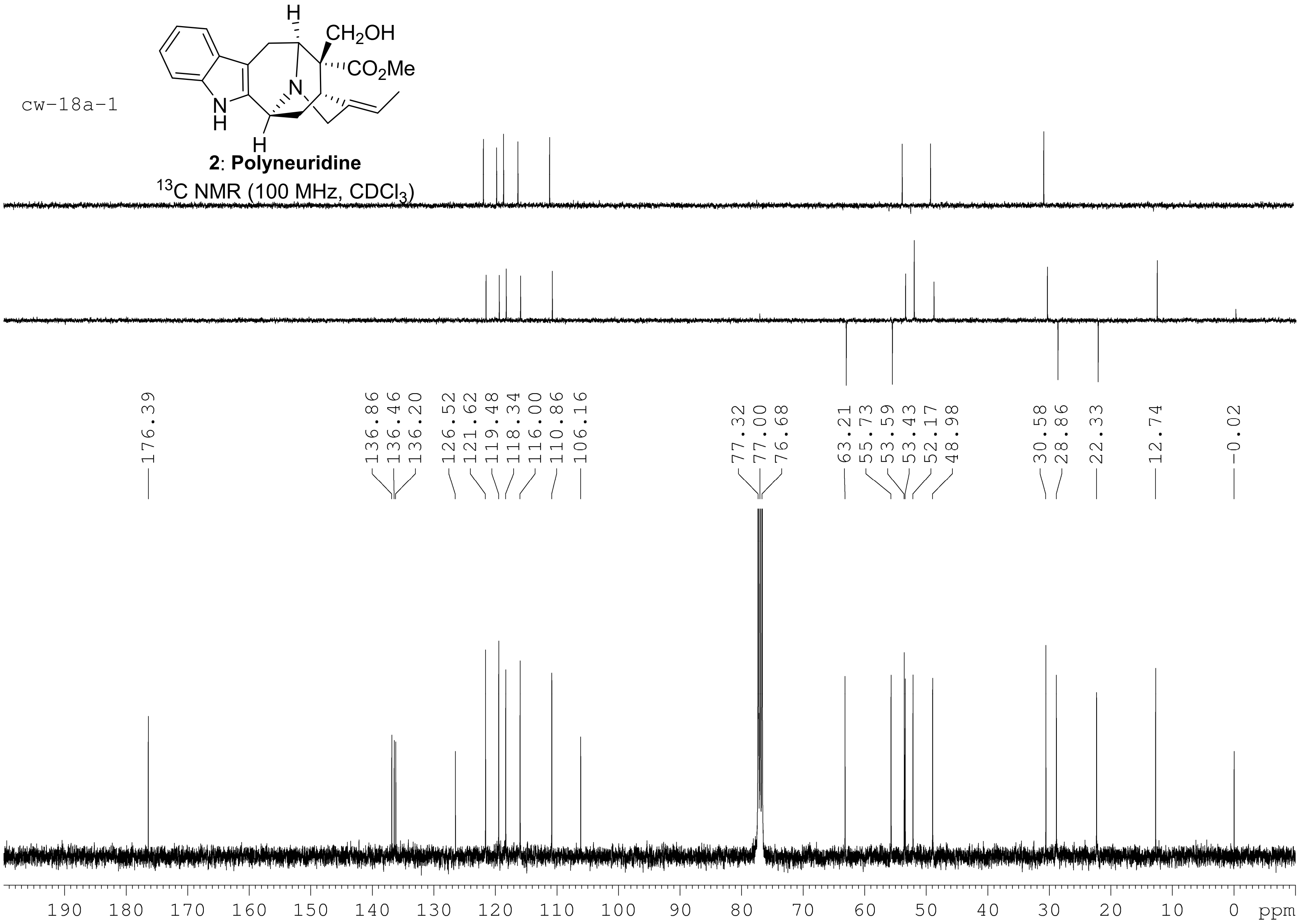

Supplementary Figure 51 ^{13}C-NMR (100 MHz, $CDCl_3$) spectra of Polyneuridine (**2**)

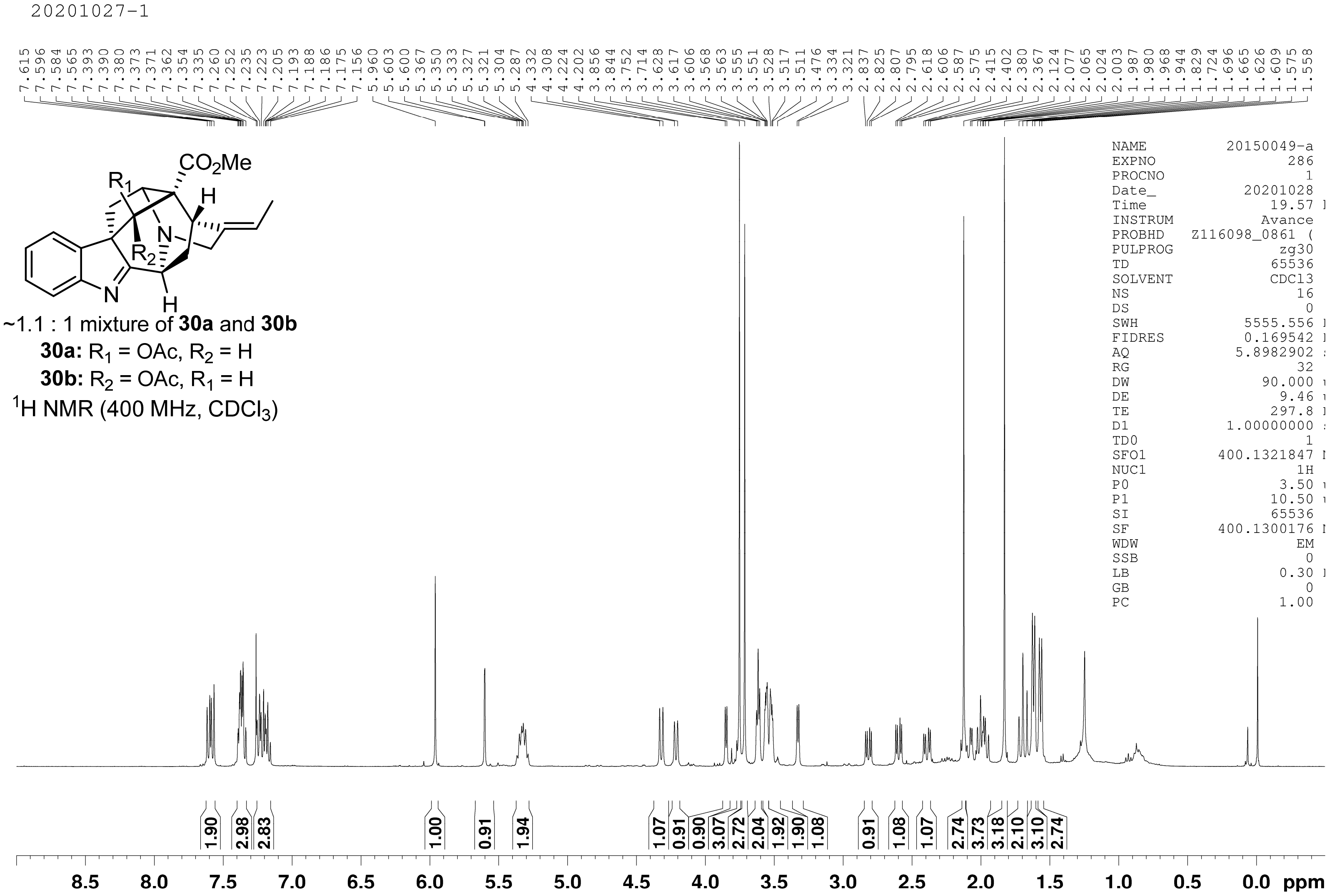

Supplementary Figure 52 ^{1}H-NMR (400 MHz, $CDCl_3$) spectra of the mixture of **30a** and **30b**

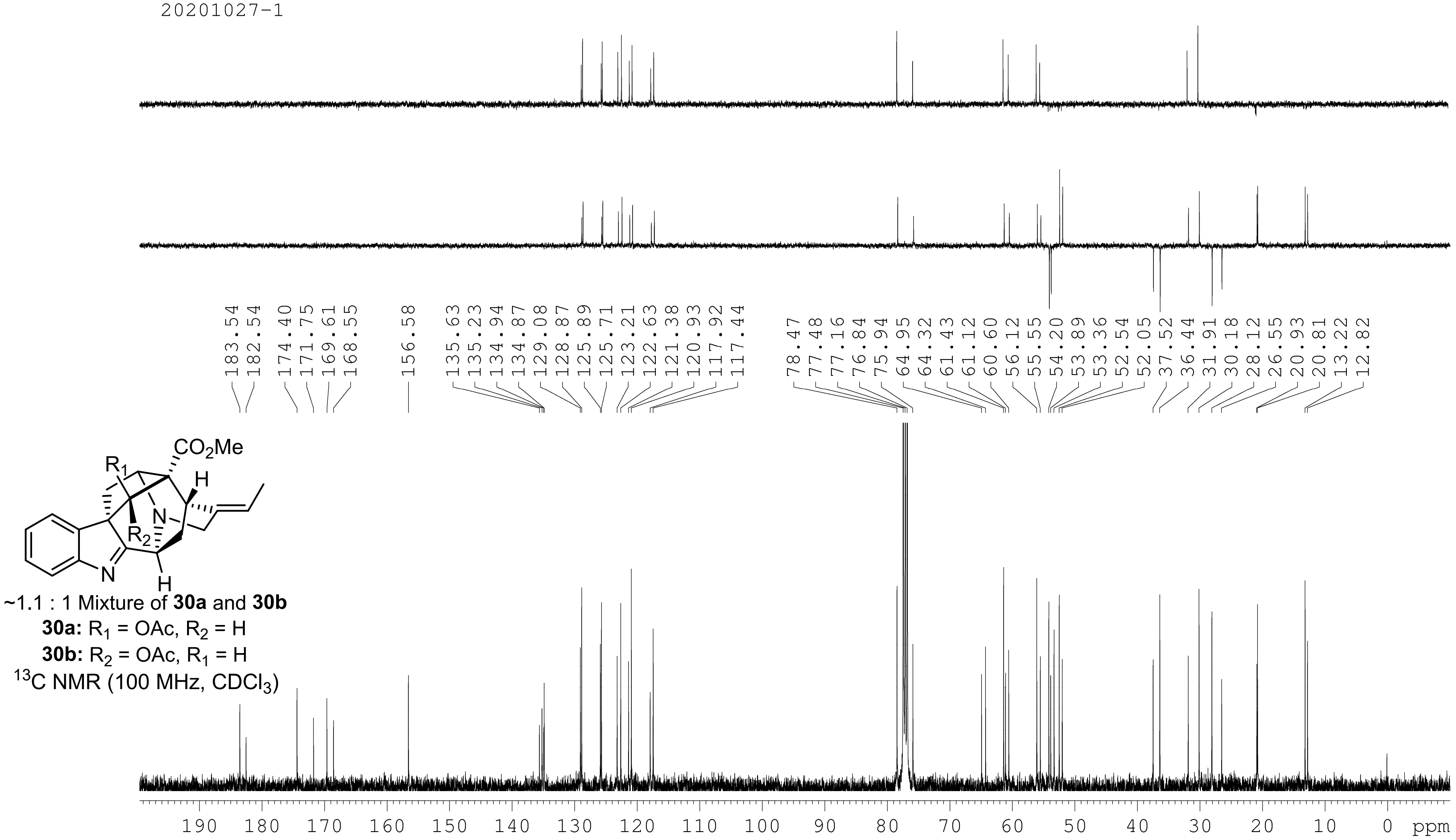

Supplementary Figure 53 ^{13}C-NMR (100 MHz, $CDCl_3$) spectra of the mixture of **30a** and **30b**

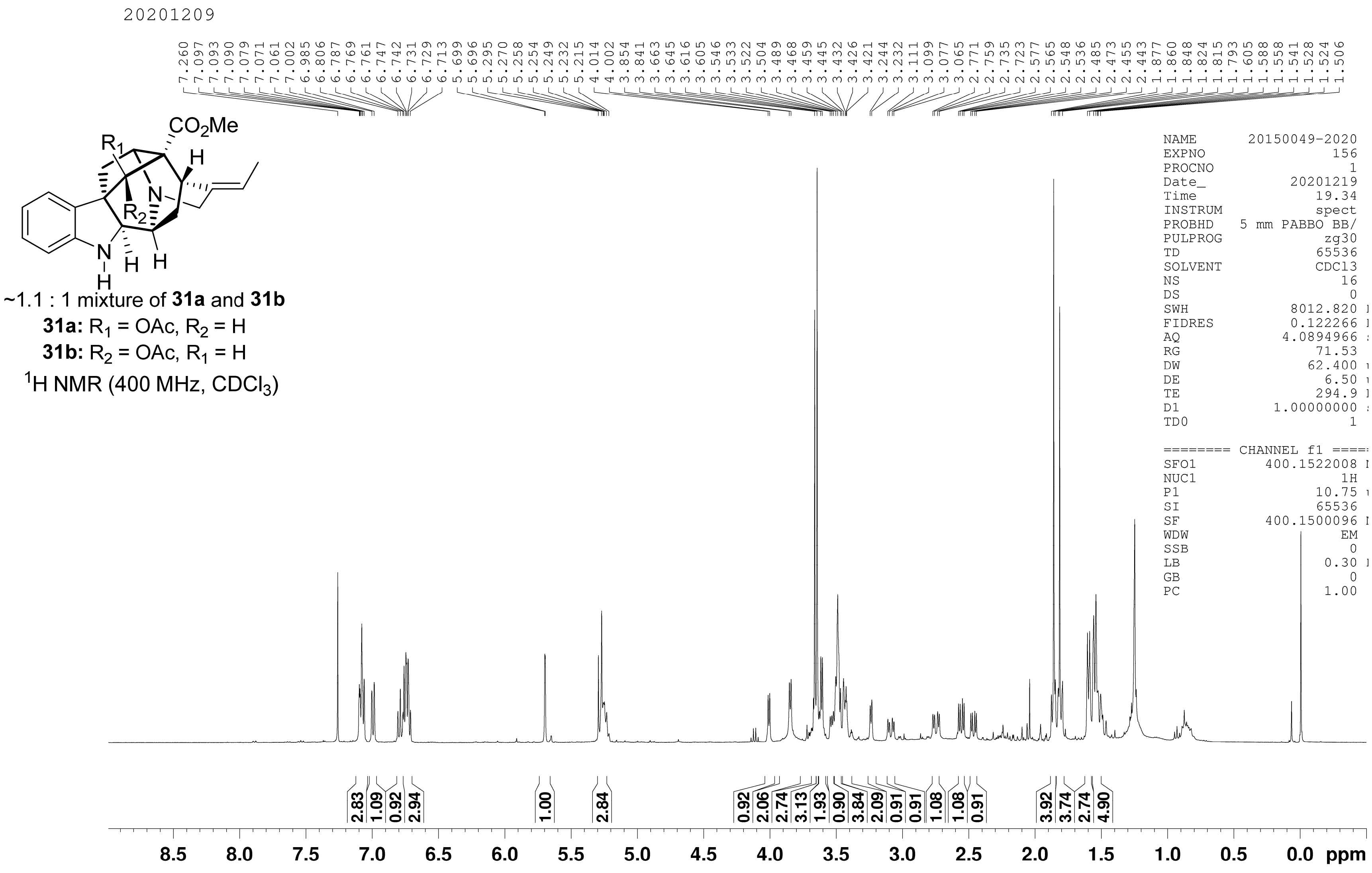

Supplementary Figure 54 ^{1}H-NMR (400 MHz, $CDCl_3$) spectra of the mixture of **31a** and **31b**

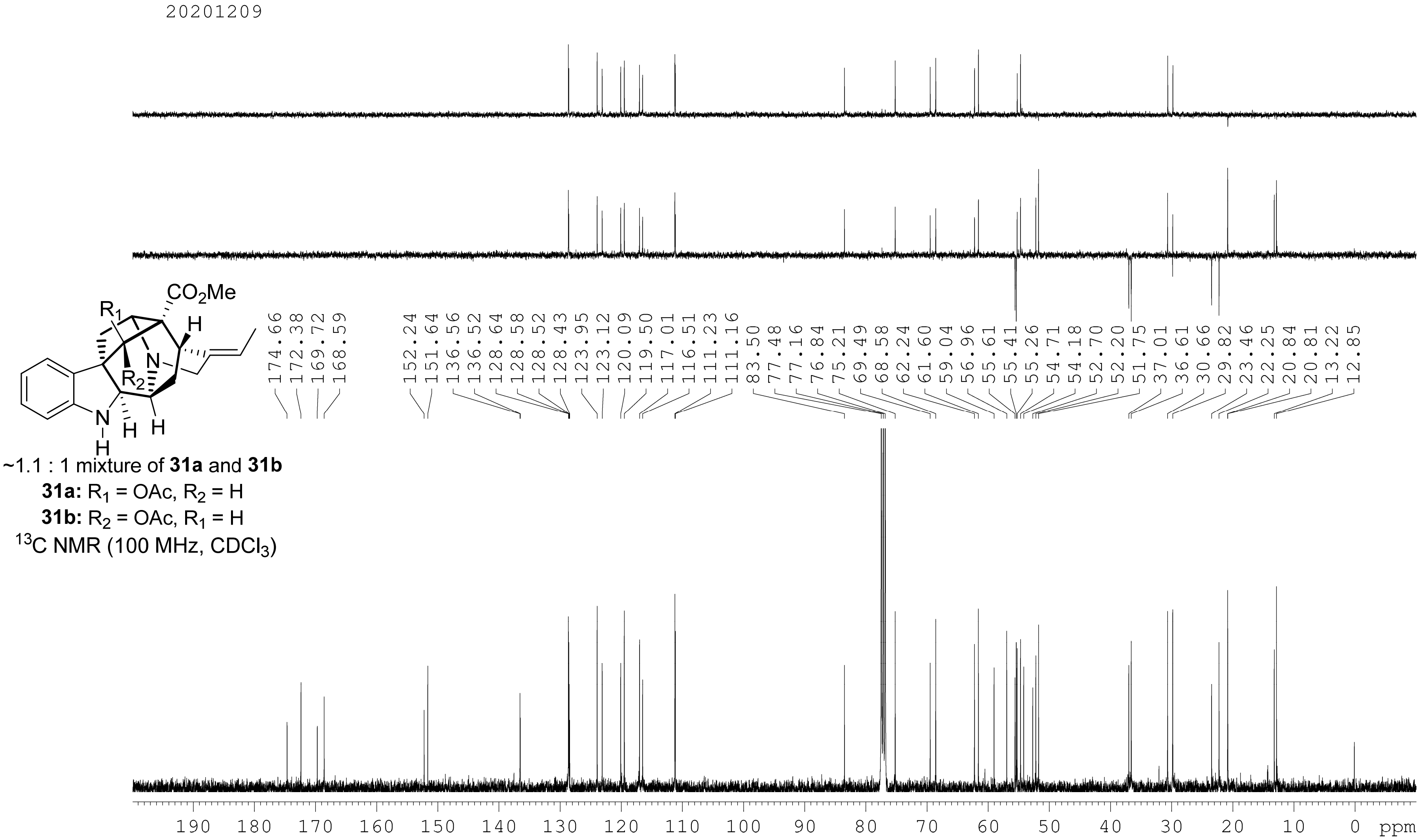

Supplementary Figure 55 ^{13}C-NMR (100 MHz, $CDCl_3$) spectra of the mixture of **31a** and **31b**

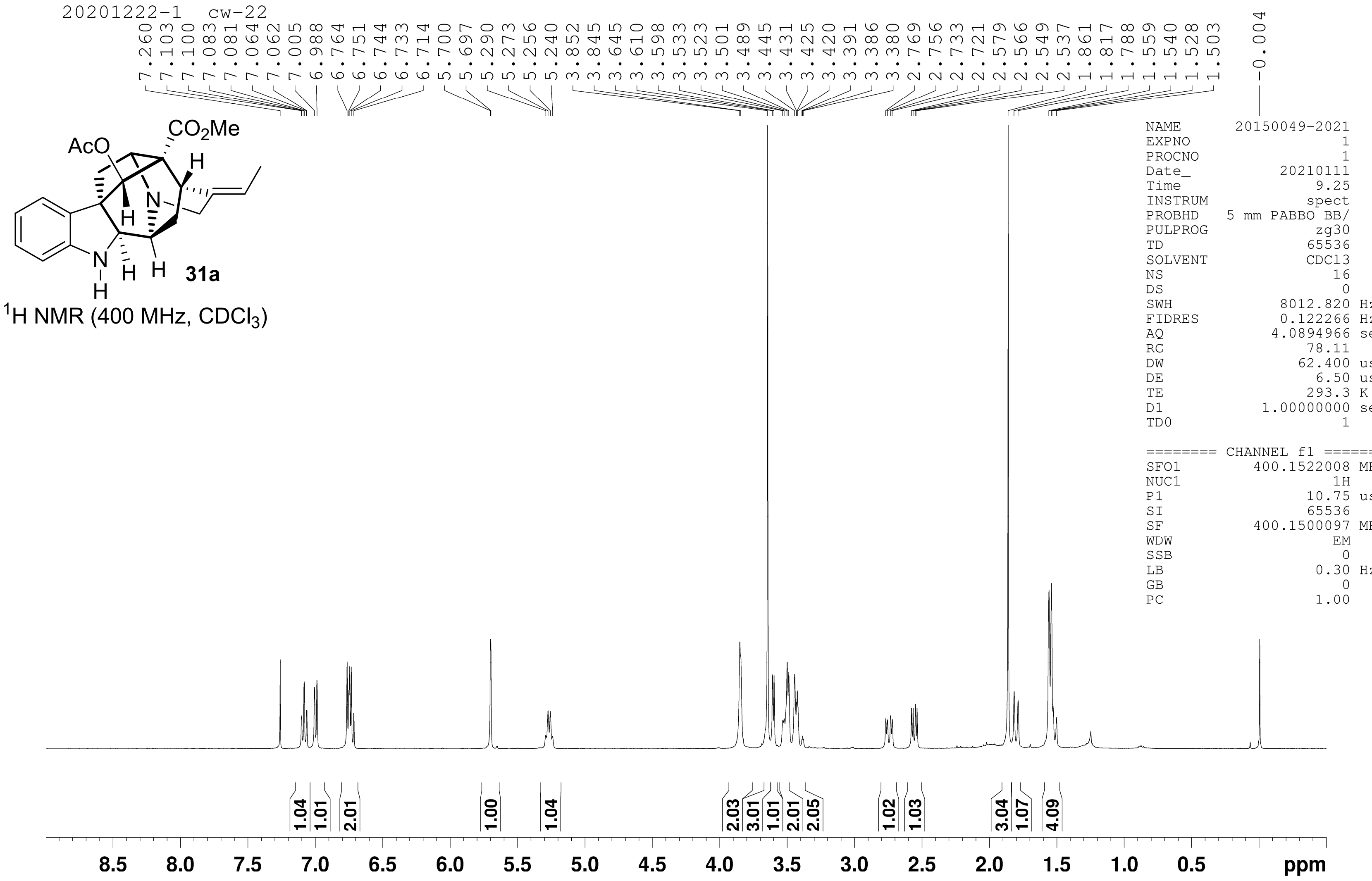

Supplementary Figure 56 ^{1}H-NMR (400 MHz, $CDCl_3$) spectra of **31a**

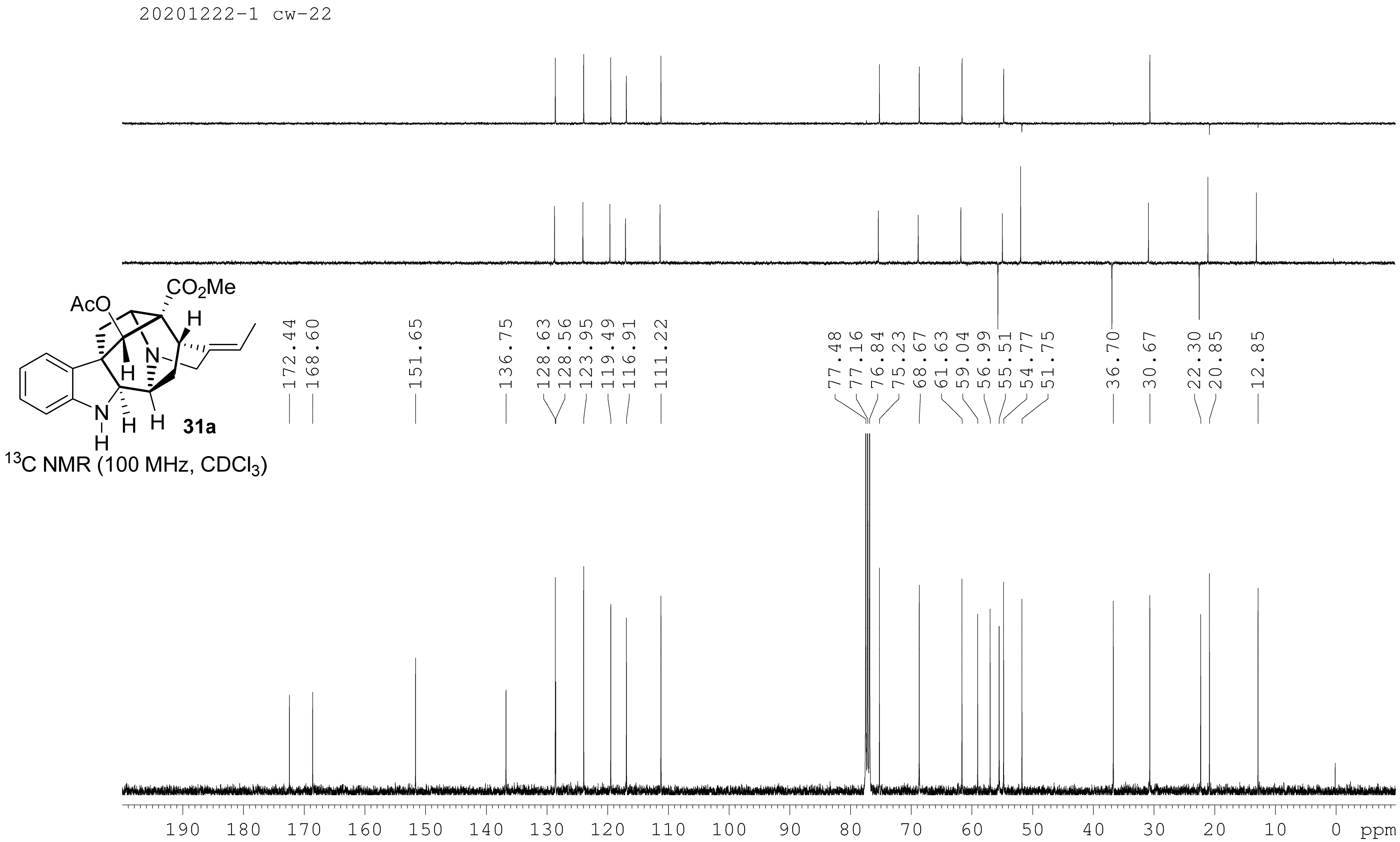

Supplementary Figure 57 ^{13}C-NMR (100 MHz, $CDCl_3$) spectra of **31a**

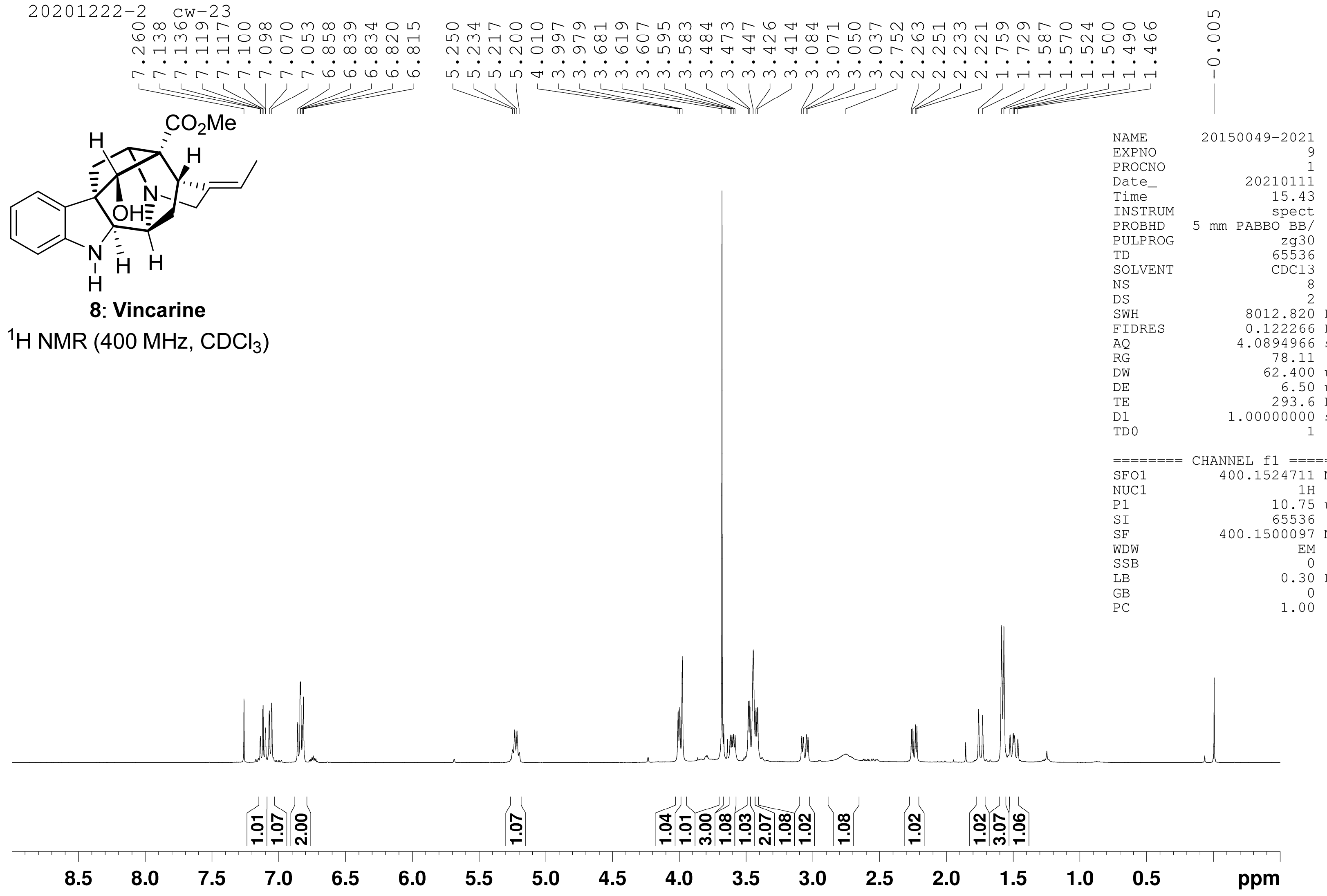

Supplementary Figure 58 ^{1}H-NMR (400 MHz, $CDCl_3$) spectra of Vincarine (**8**)

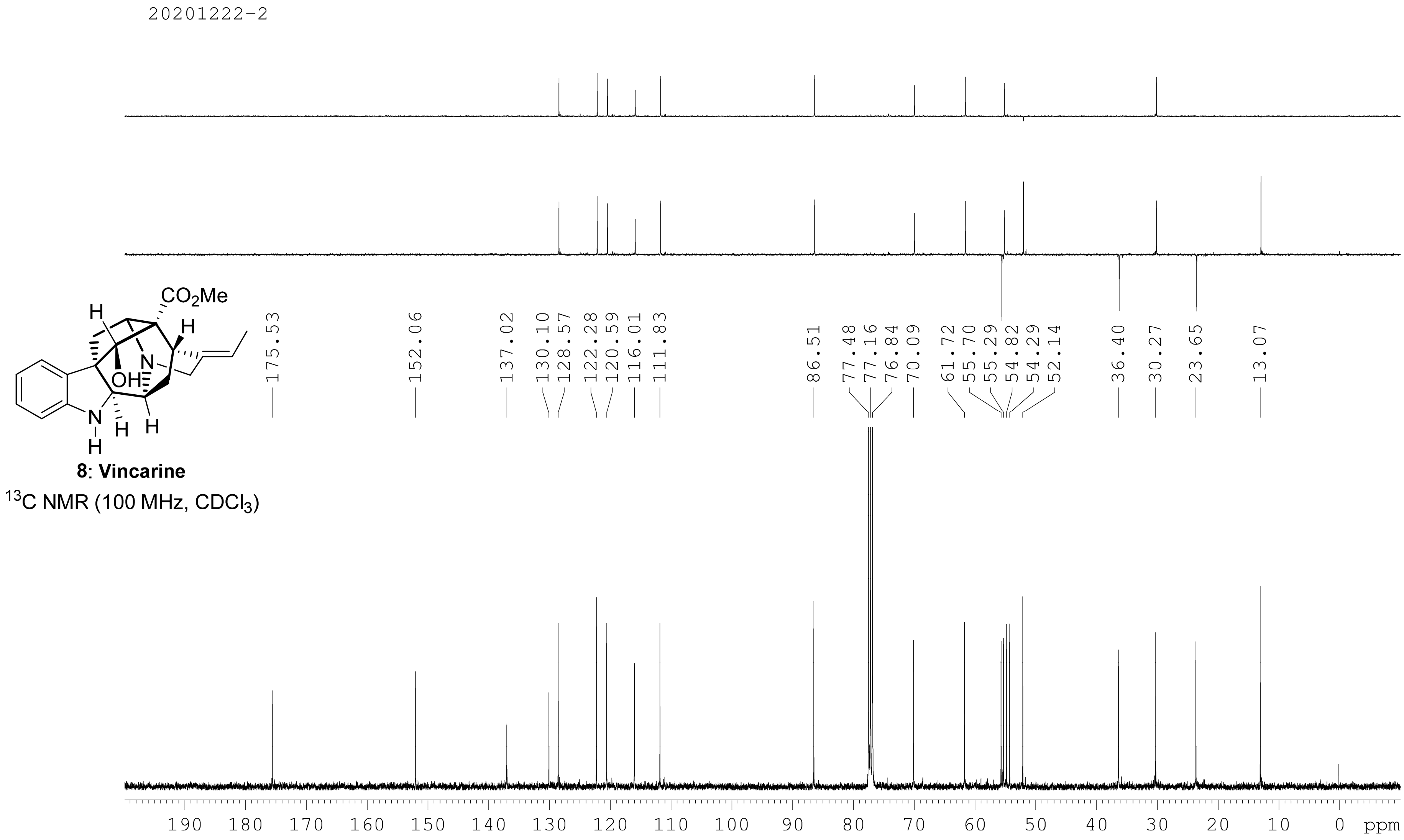

Supplementary Figure 59 ^{13}C-NMR (100 MHz, $CDCl_3$) spectra of Vincarine (**8**)

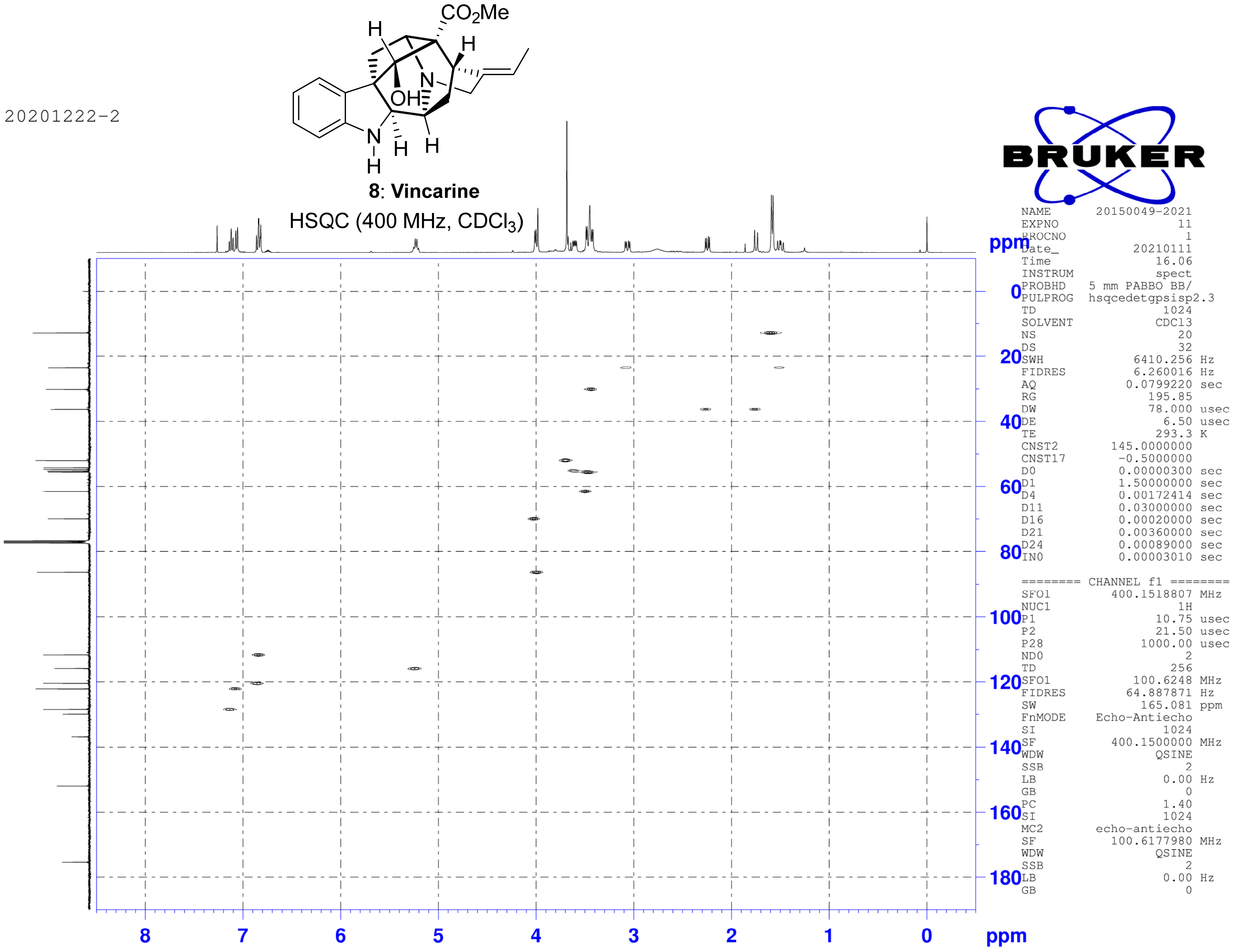

Supplementary Figure 60 HSQC (400 MHz, $CDCl_3$) spectra of Vincarine (**8**)

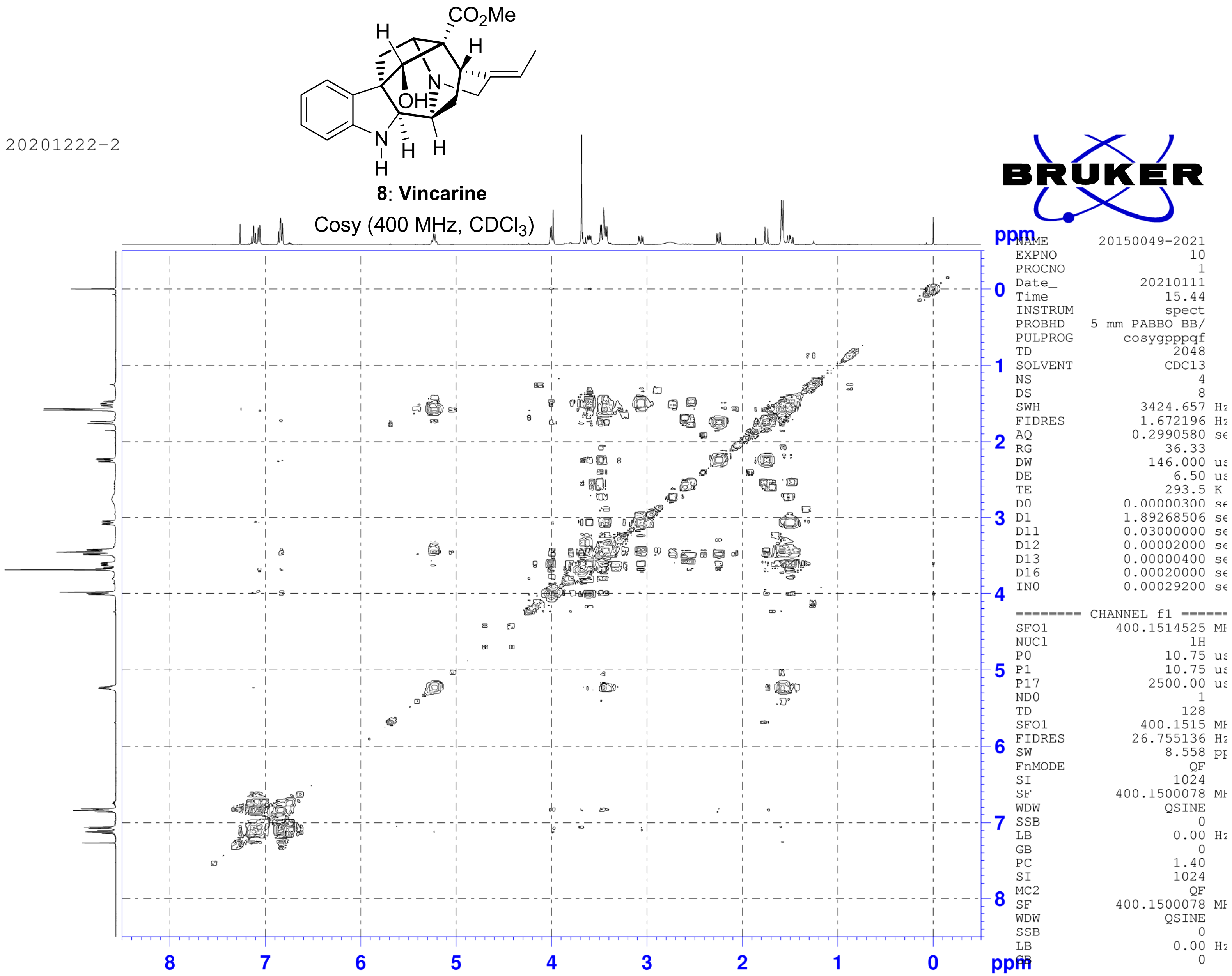

Supplementary Figure 61 Cosy (400 MHz, CDCl3) spectra of Vincarine (**8**)

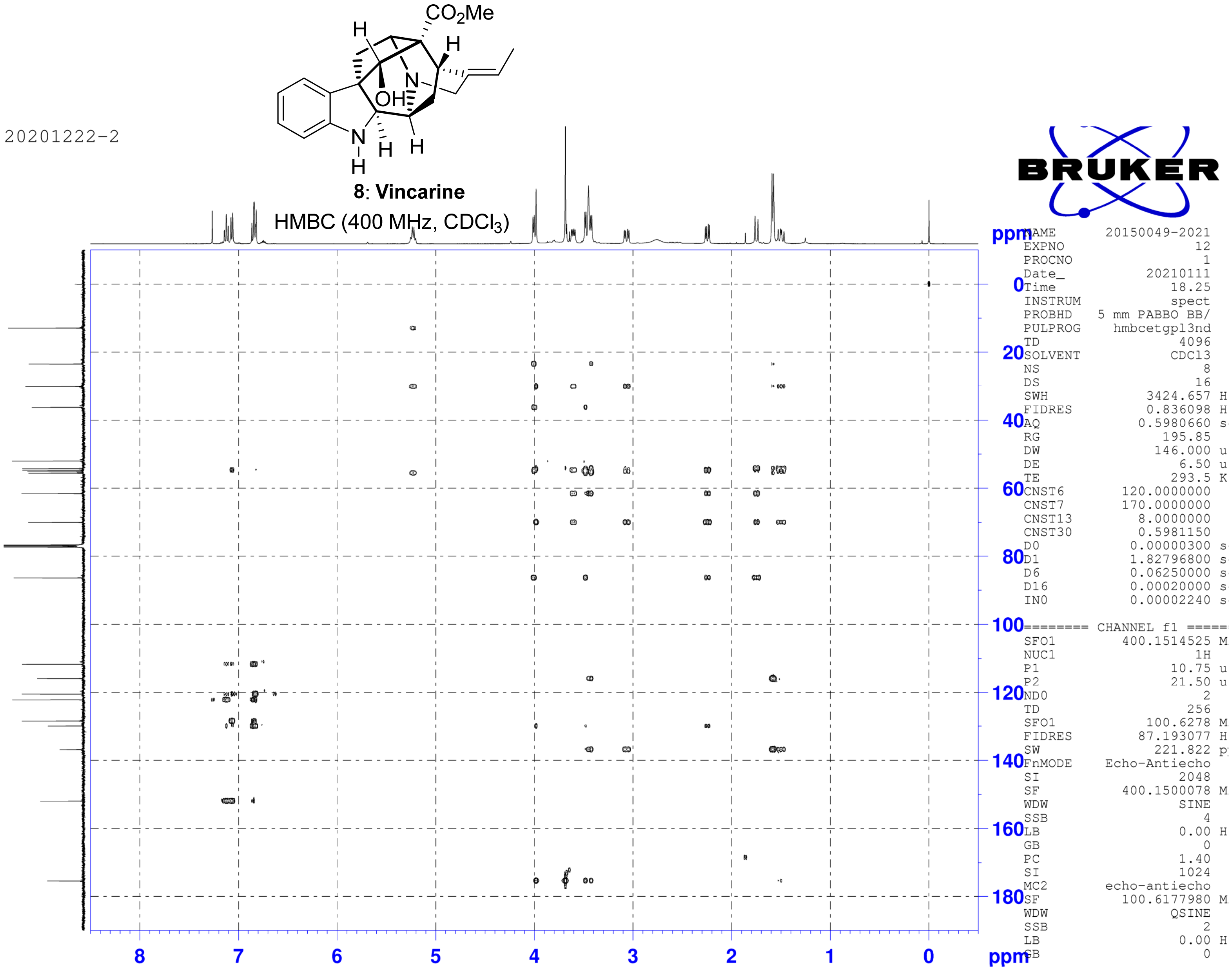

Supplementary Figure 62 HMBC (400 MHz, $CDCl_3$) spectra of Vincarine (**8**)

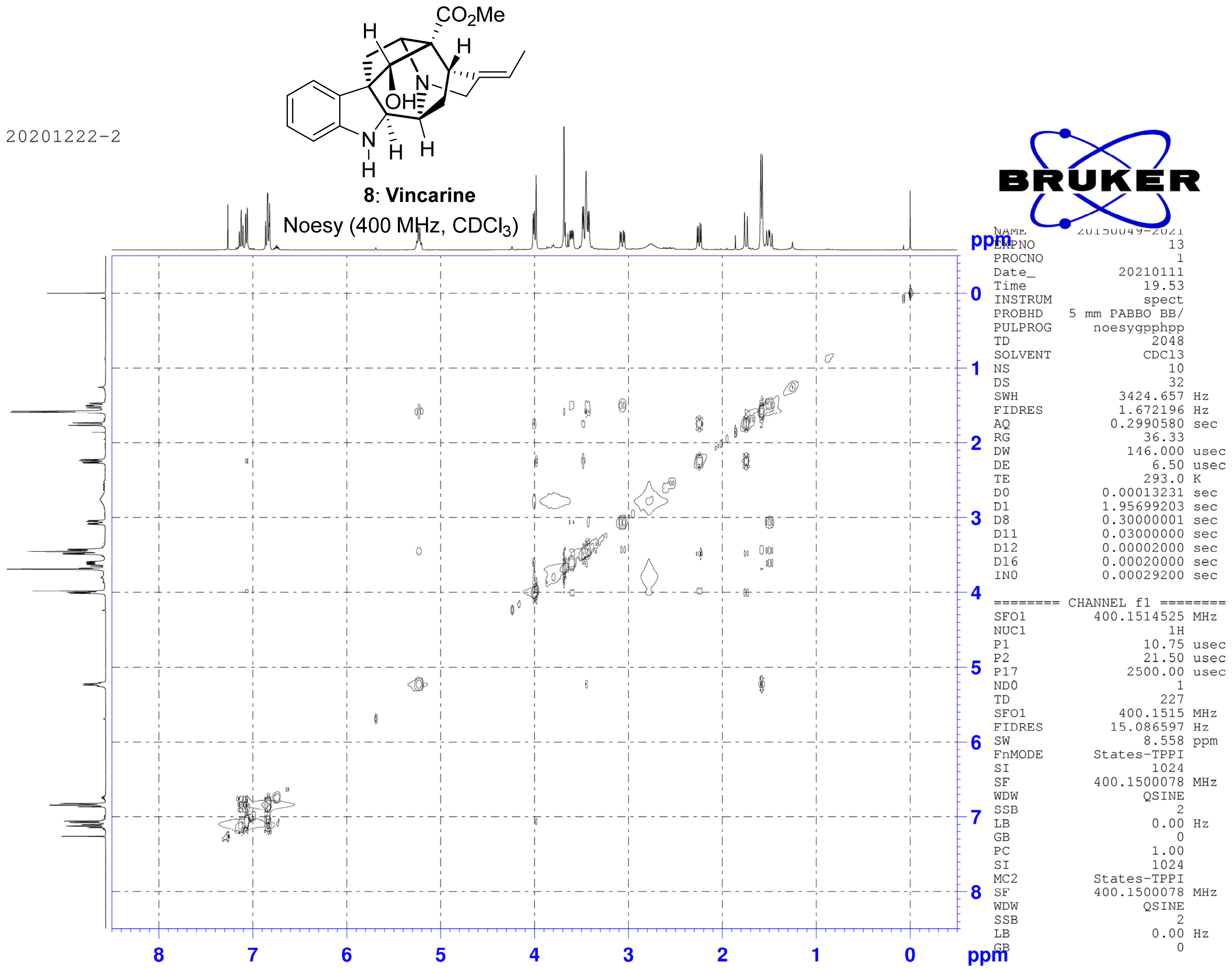

Supplementary Figure 63 Noesy (400 MHz, $CDCl_3$) spectra of Vincarine (**8**)

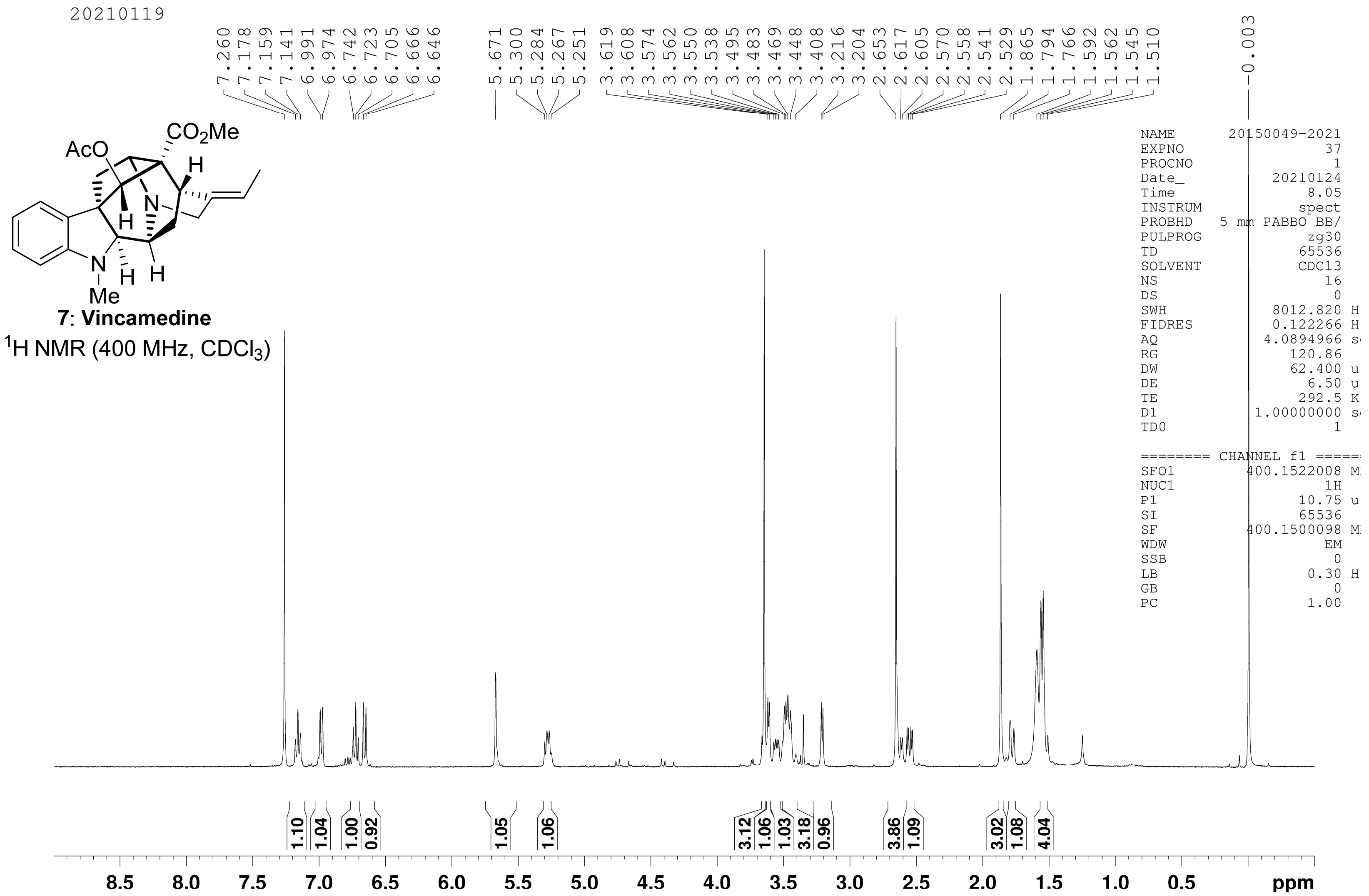

Supplementary Figure 64 ^{1}H-NMR (400 MHz, $CDCl_3$) spectra of Vincamedine (**7**)

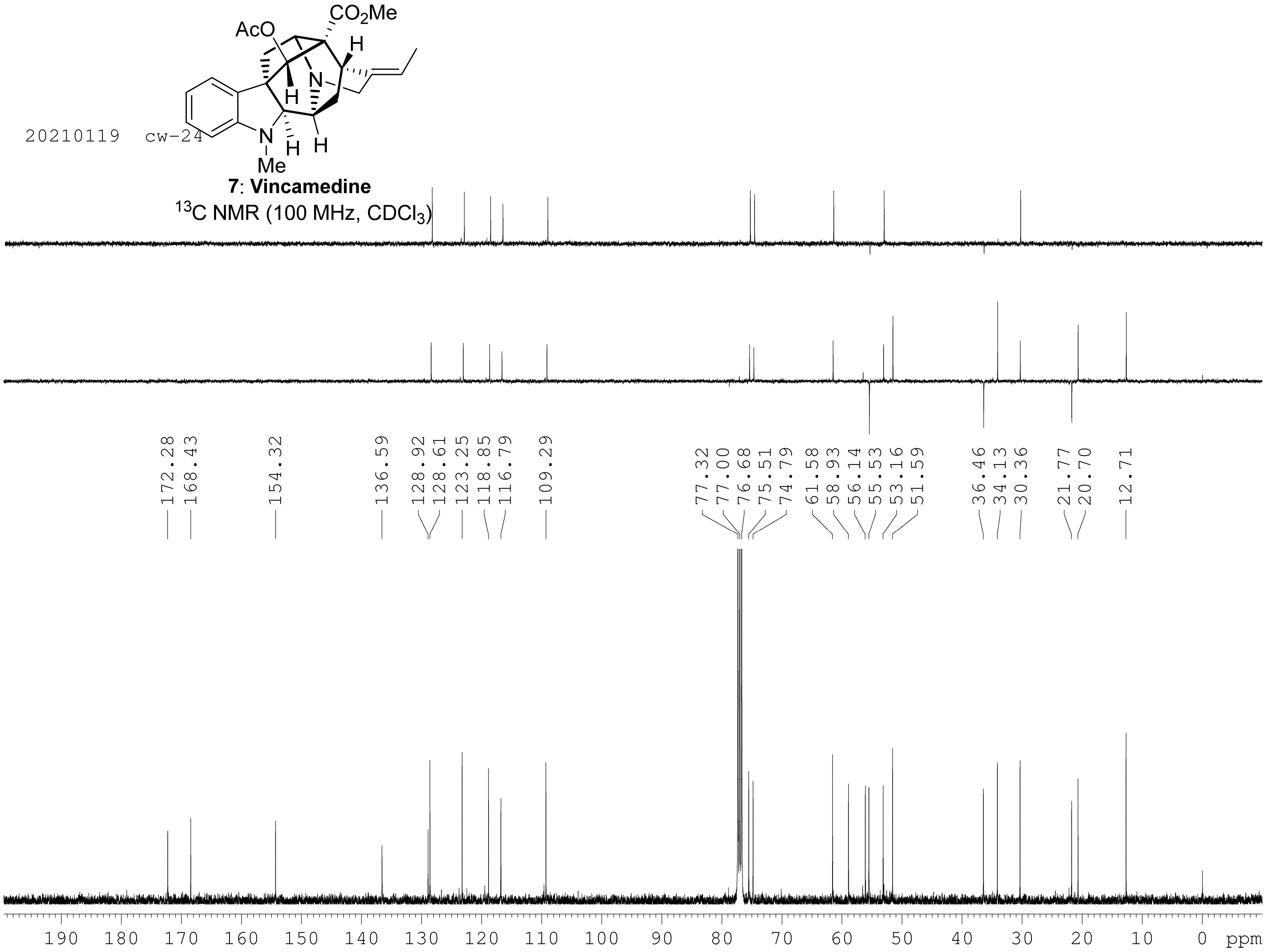

Supplementary Figure 65 ^{13}C-NMR (100 MHz, $CDCl_3$) spectra of Vincamedine (**7**)

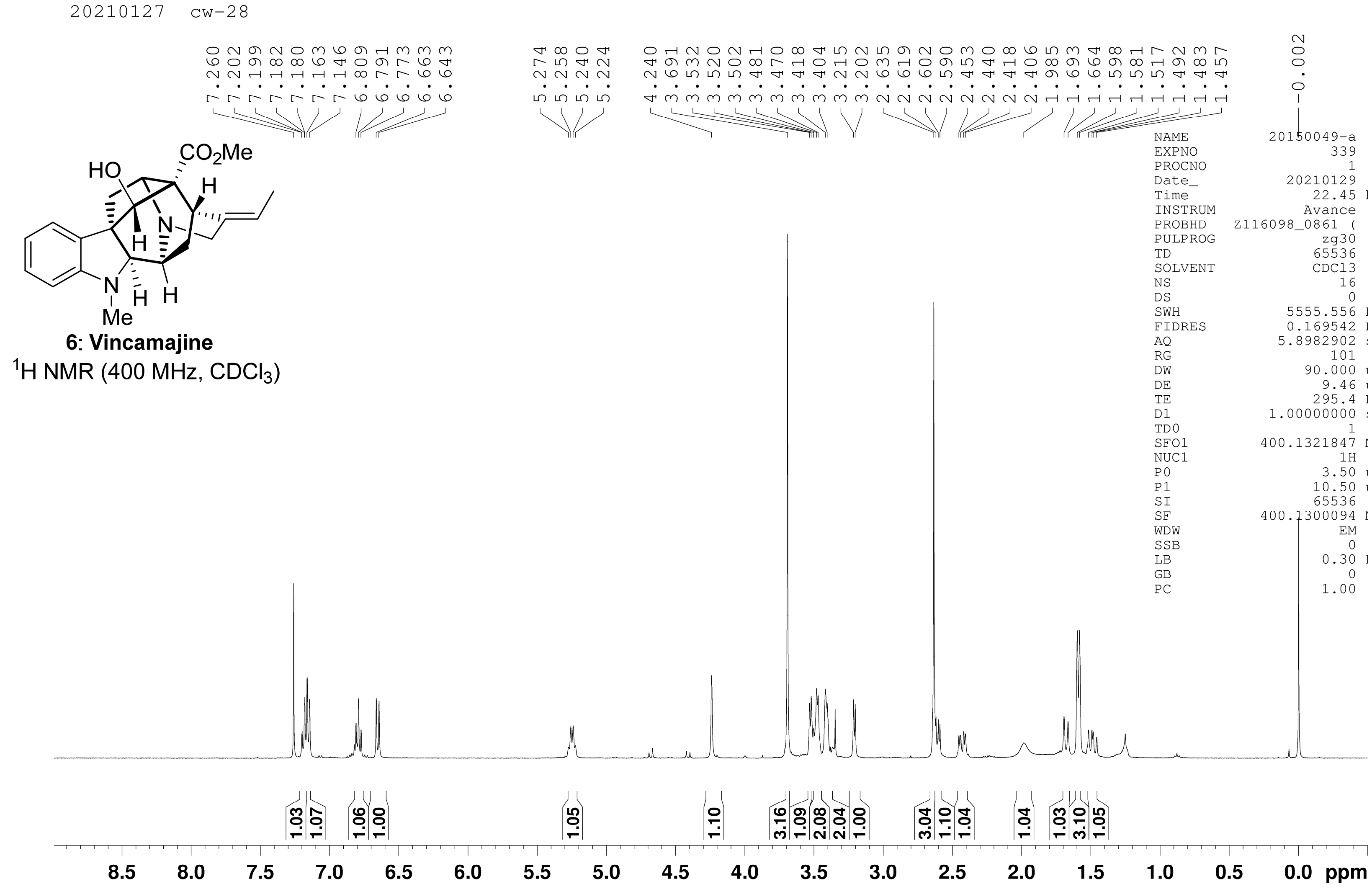

Supplementary Figure 66 ^{1}H-NMR (400 MHz, $CDCl_3$) spectra of Vincamajine (**6**)

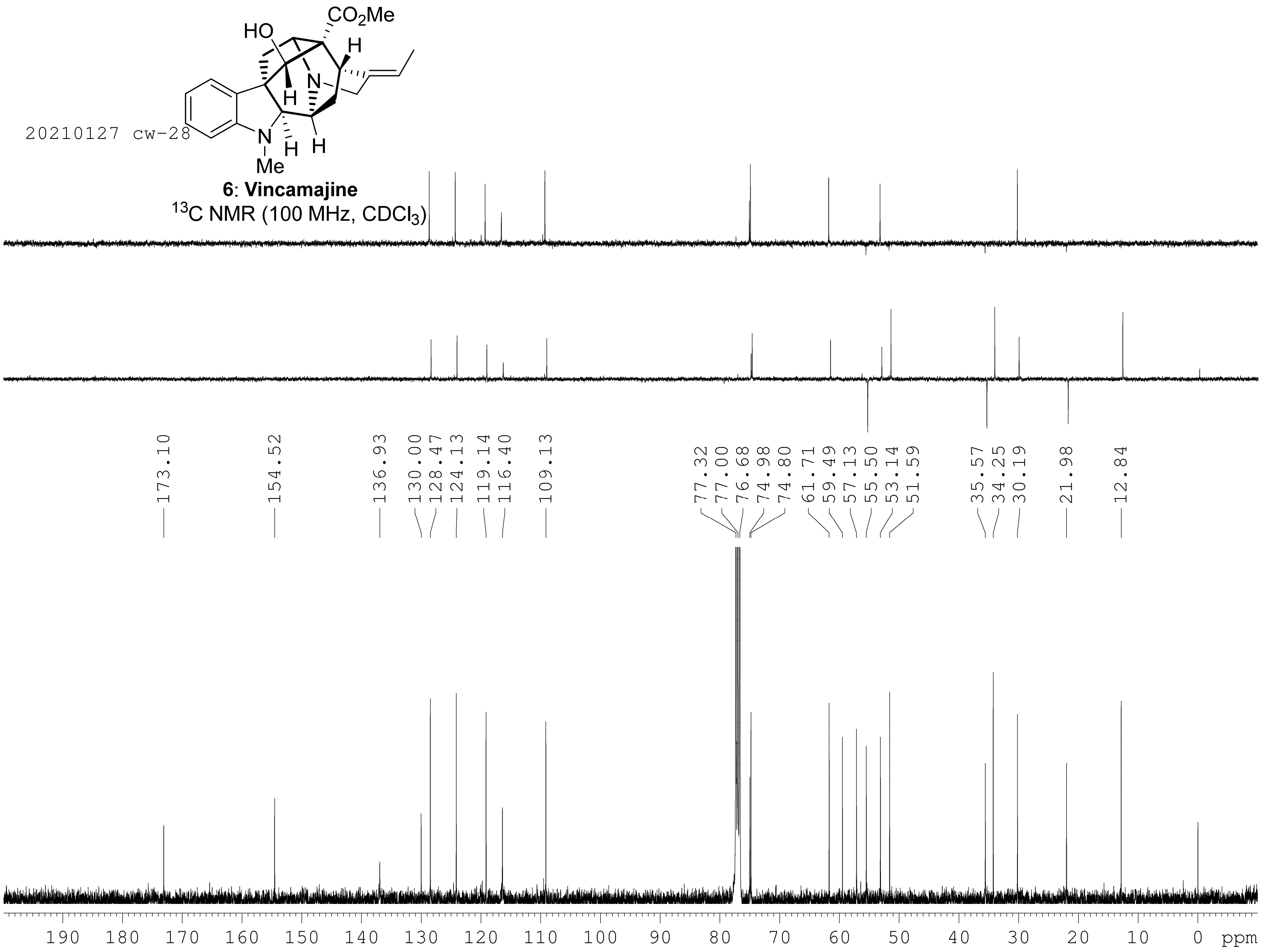

Supplementary Figure 67 ^{13}C-NMR (100 MHz, CDCl₃) spectra of Vincamajine (**6**)

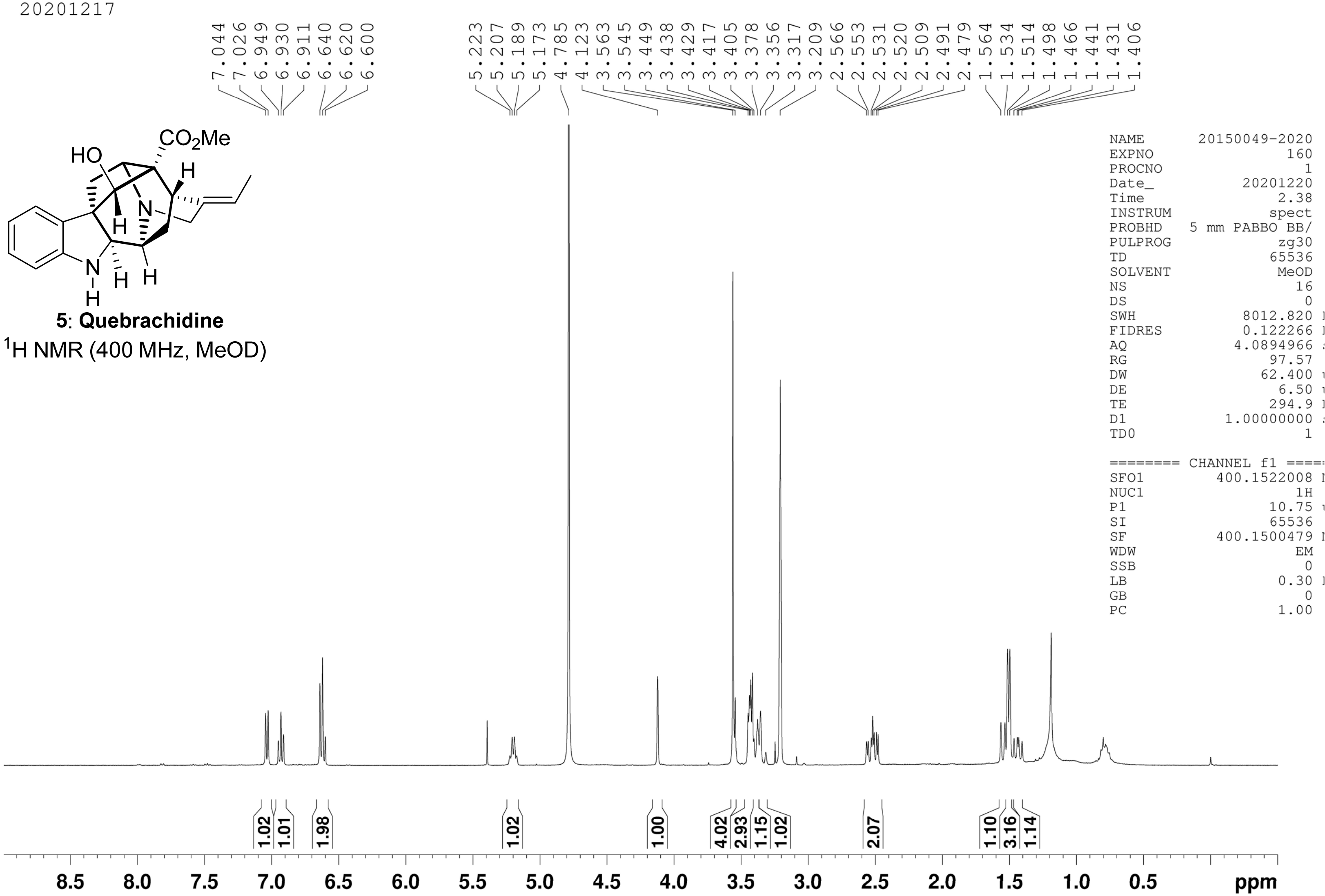

Supplementary Figure 68 ^{1}H-NMR (400 MHz, MeOD) spectra of Quebrachidine (**5**)

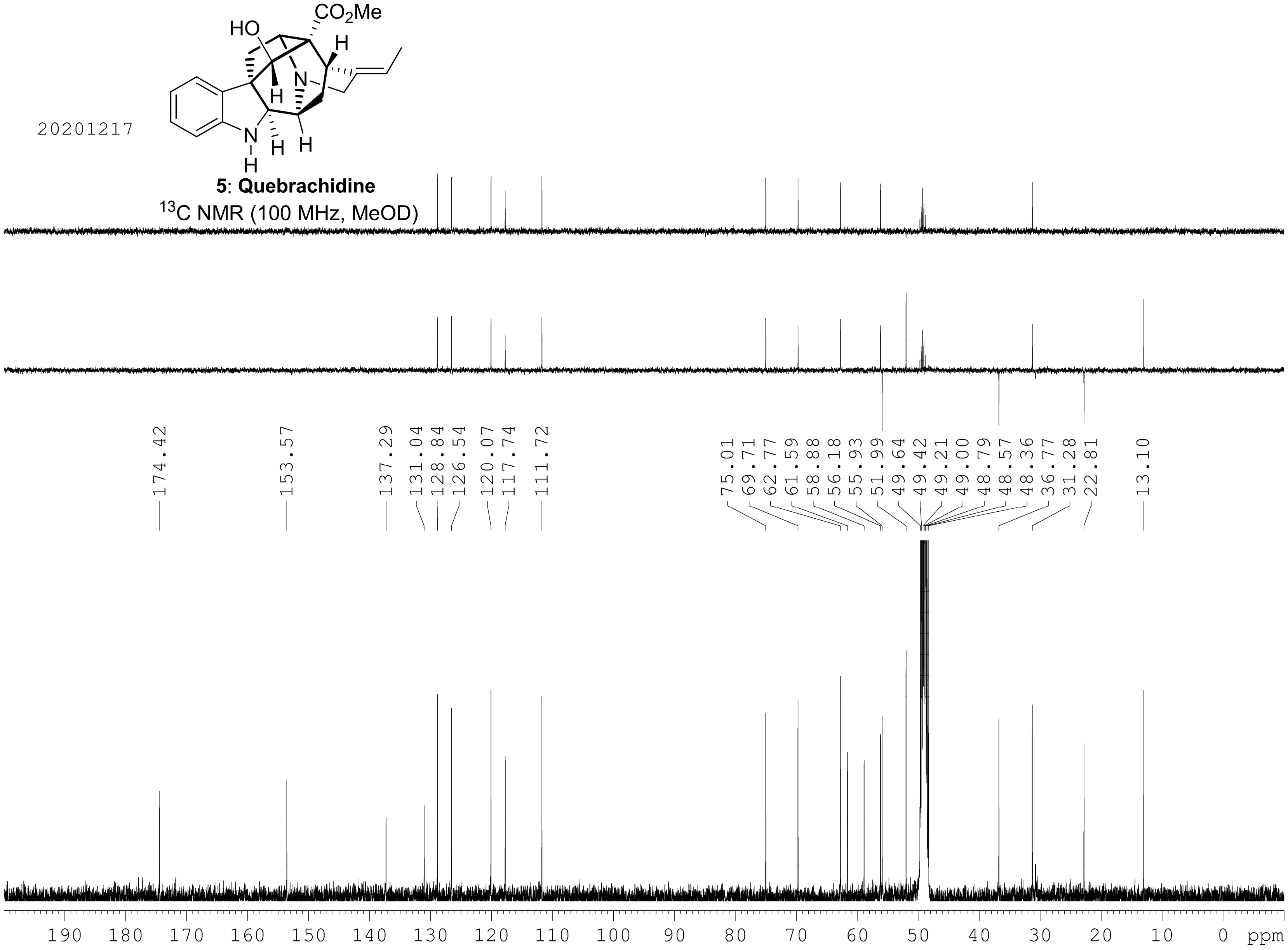

Supplementary Figure 69 ^{13}C-NMR (100 MHz, MeOD) spectra of Quebrachidine (**5**)

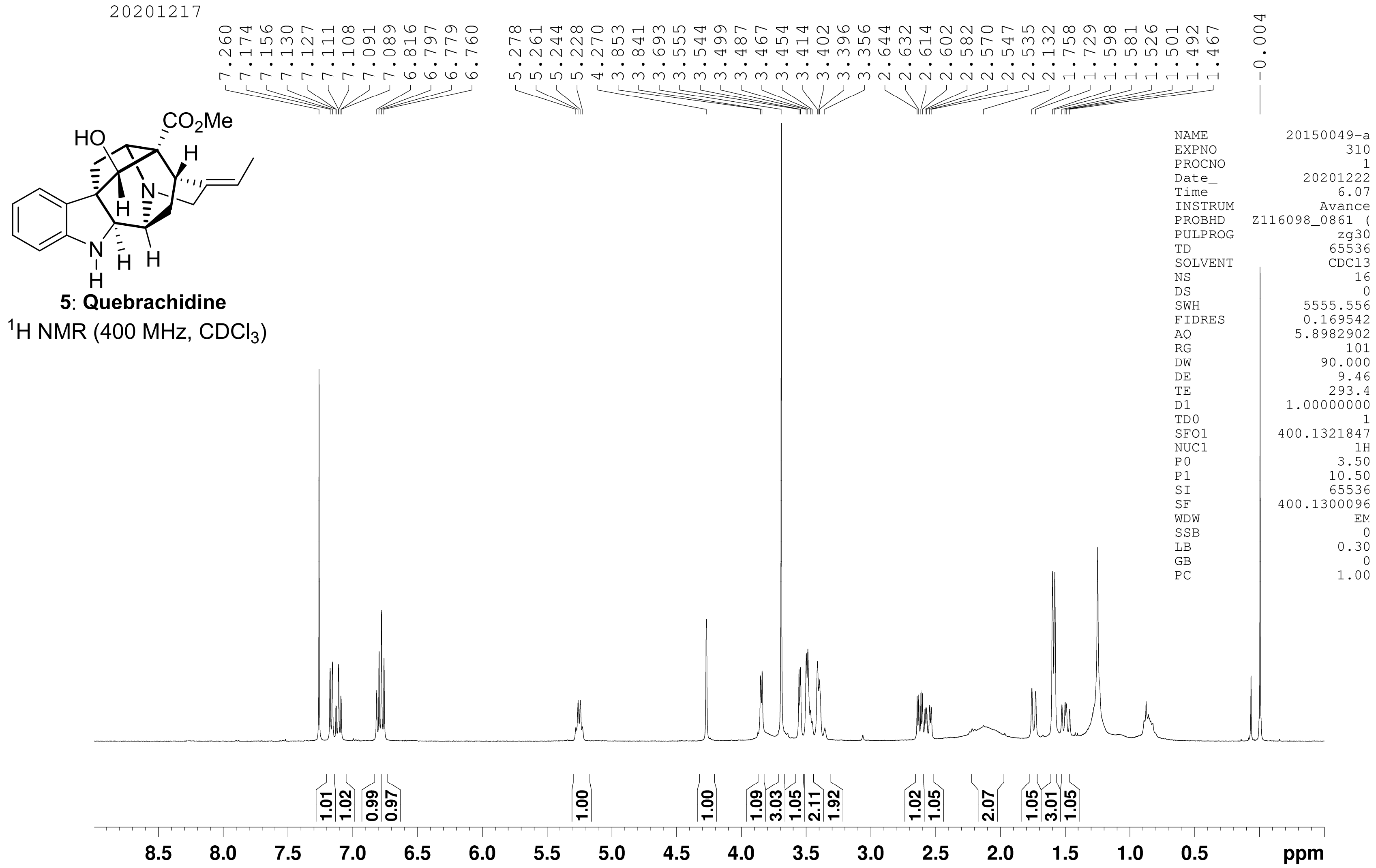

Supplementary Figure 70 ^{1}H-NMR (400 MHz, $CDCl_3$) spectra of Quebrachidine (**5**)

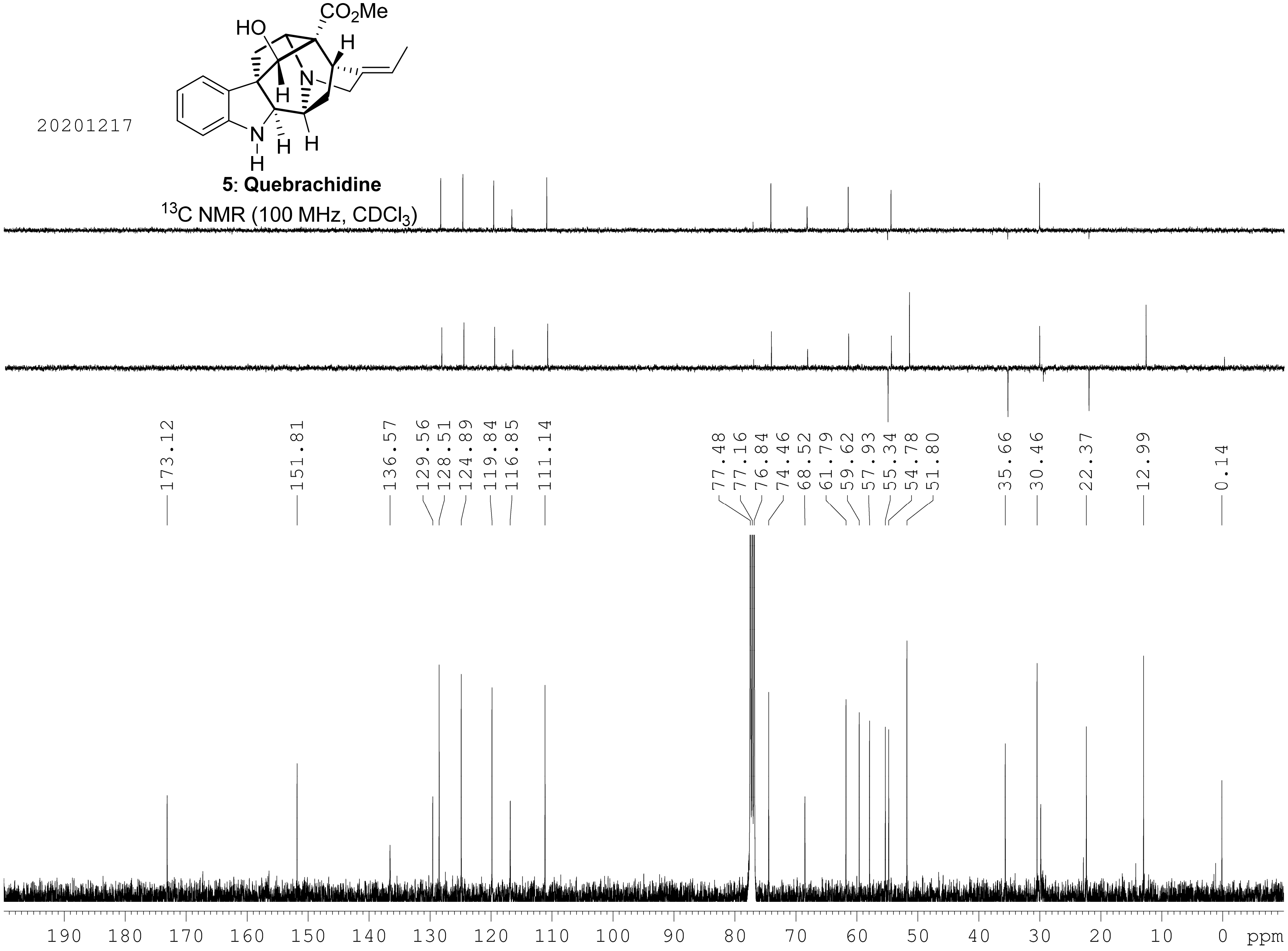

Supplementary Figure 71 ^{13}C-NMR (100 MHz, $CDCl_3$) spectra of Quebrachidine (**5**)

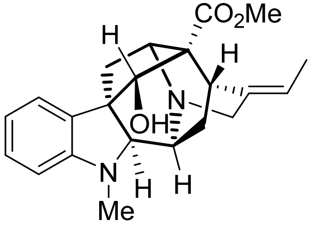

9: **Vincamajinine**

^{1}H NMR (400 MHz, $CDCl_3$)

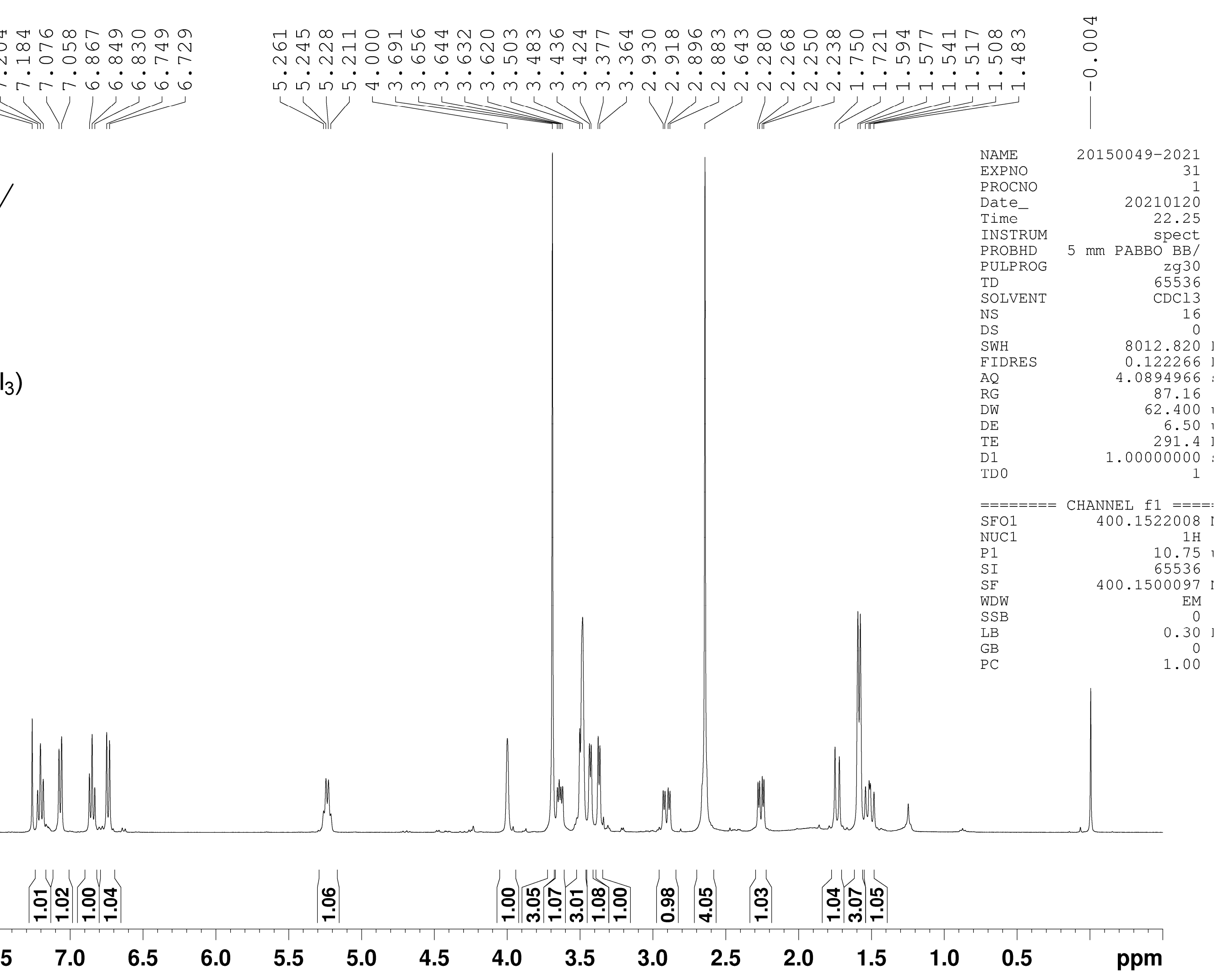

Supplementary Figure 72 ^{1}H-NMR (400 MHz, $CDCl_3$) spectra of Vincamajinine (**9**)

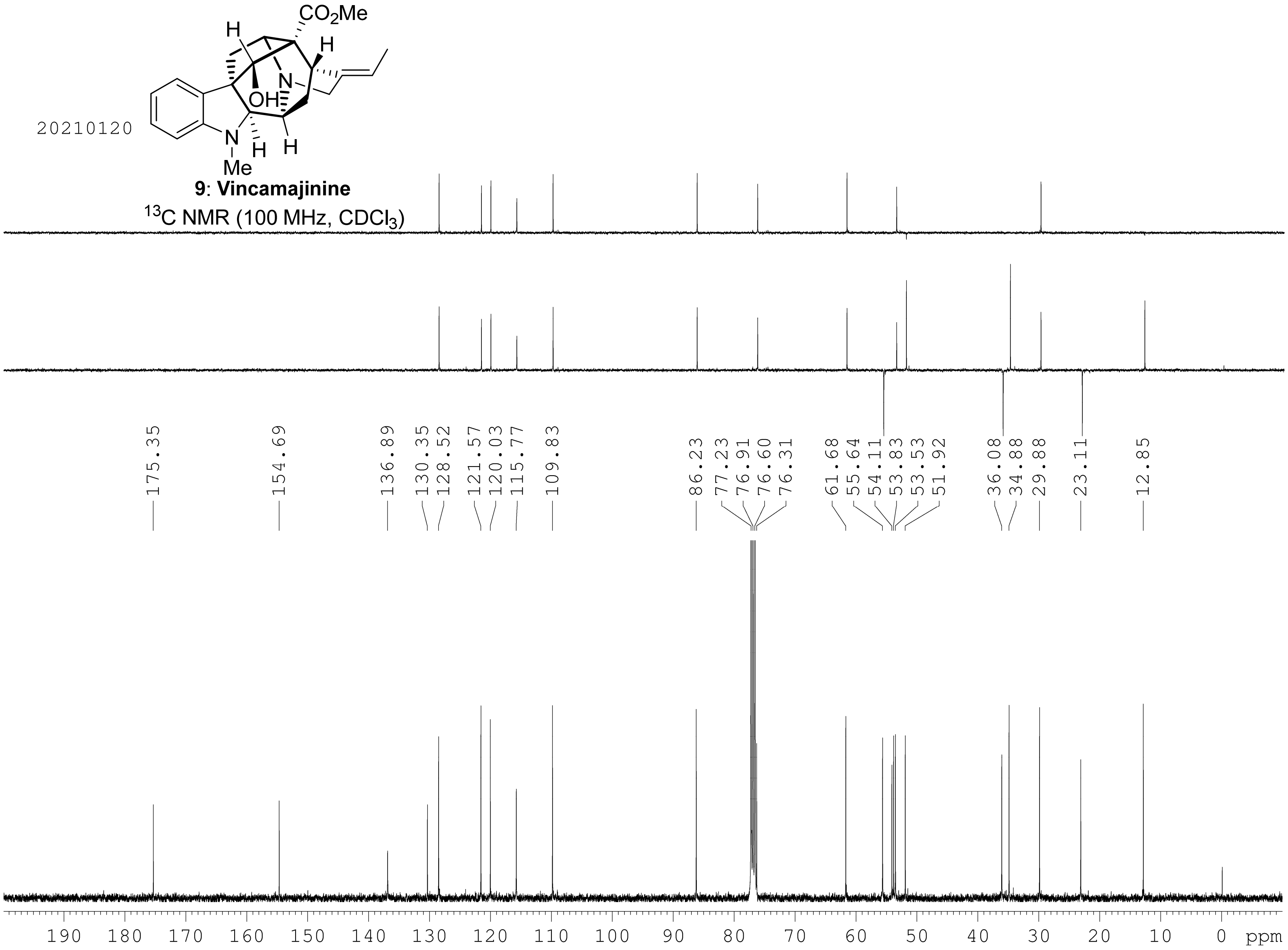

Supplementary Figure 73 ^{13}C-NMR (100 MHz, $CDCl_3$) spectra of Vincamajinine (**9**)

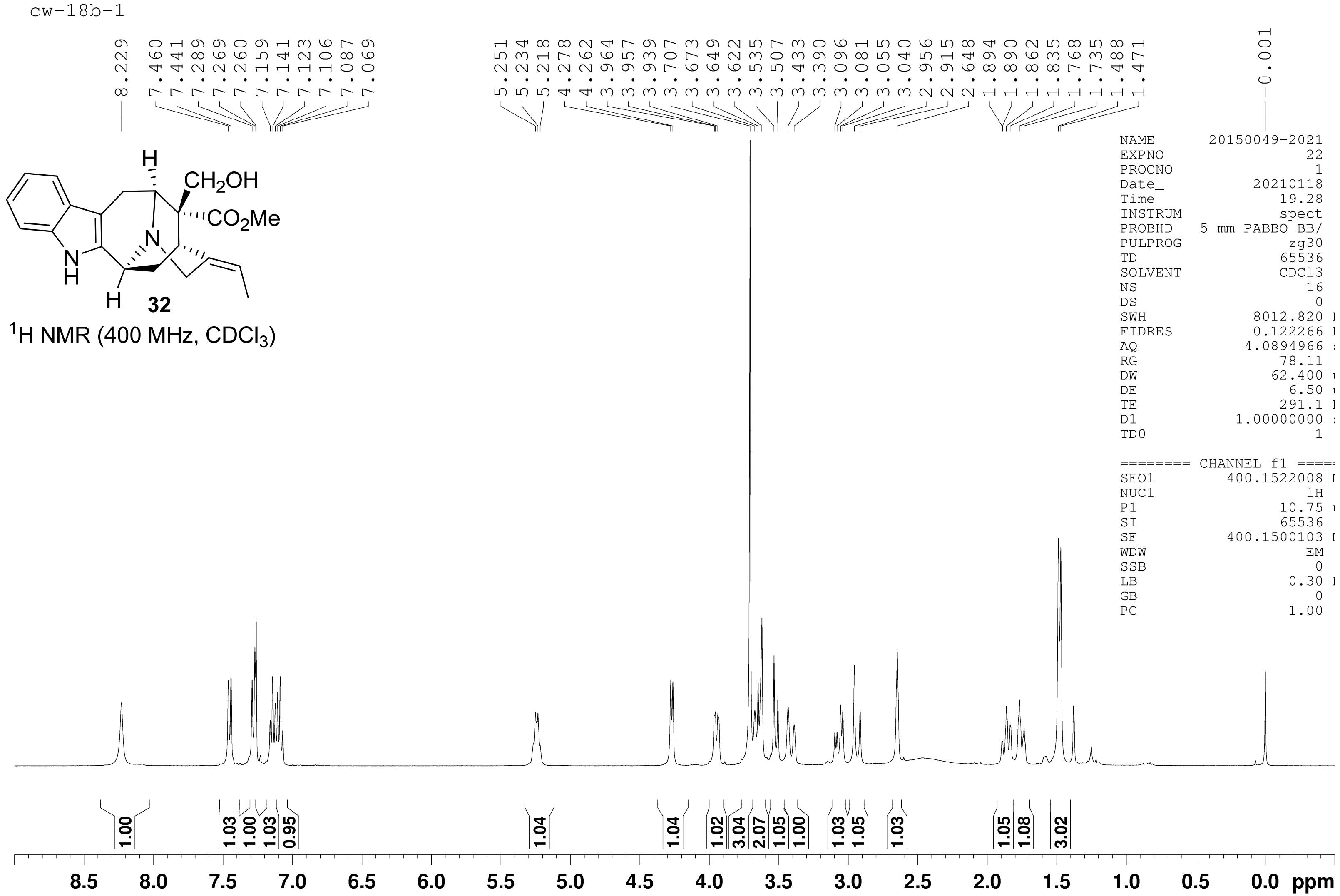

Supplementary Figure 74 ^{1}H-NMR (400 MHz, $CDCl_3$) spectra of **32**

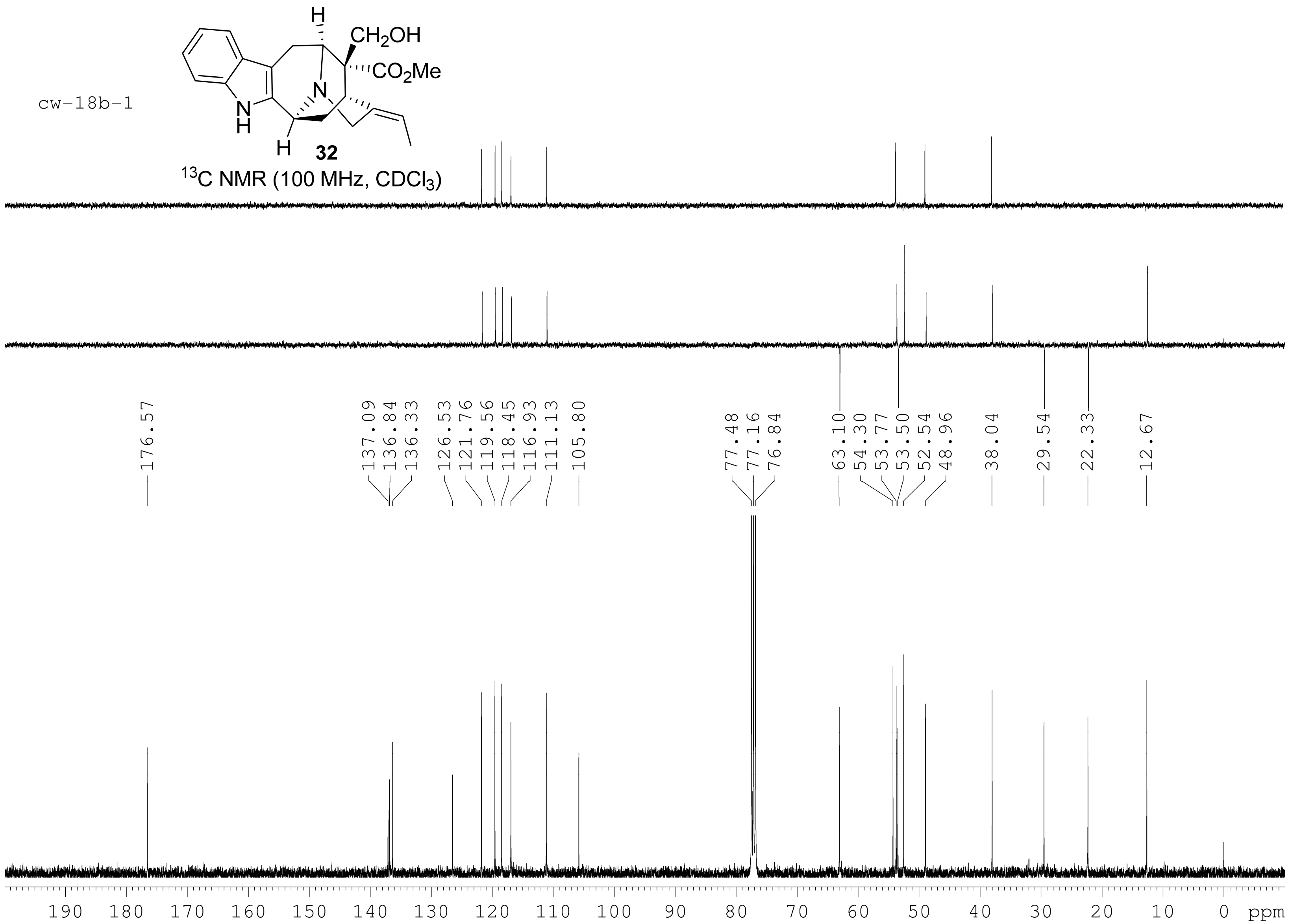

Supplementary Figure 75 ^{13}C-NMR (100 MHz, $CDCl_3$) spectra of **32**

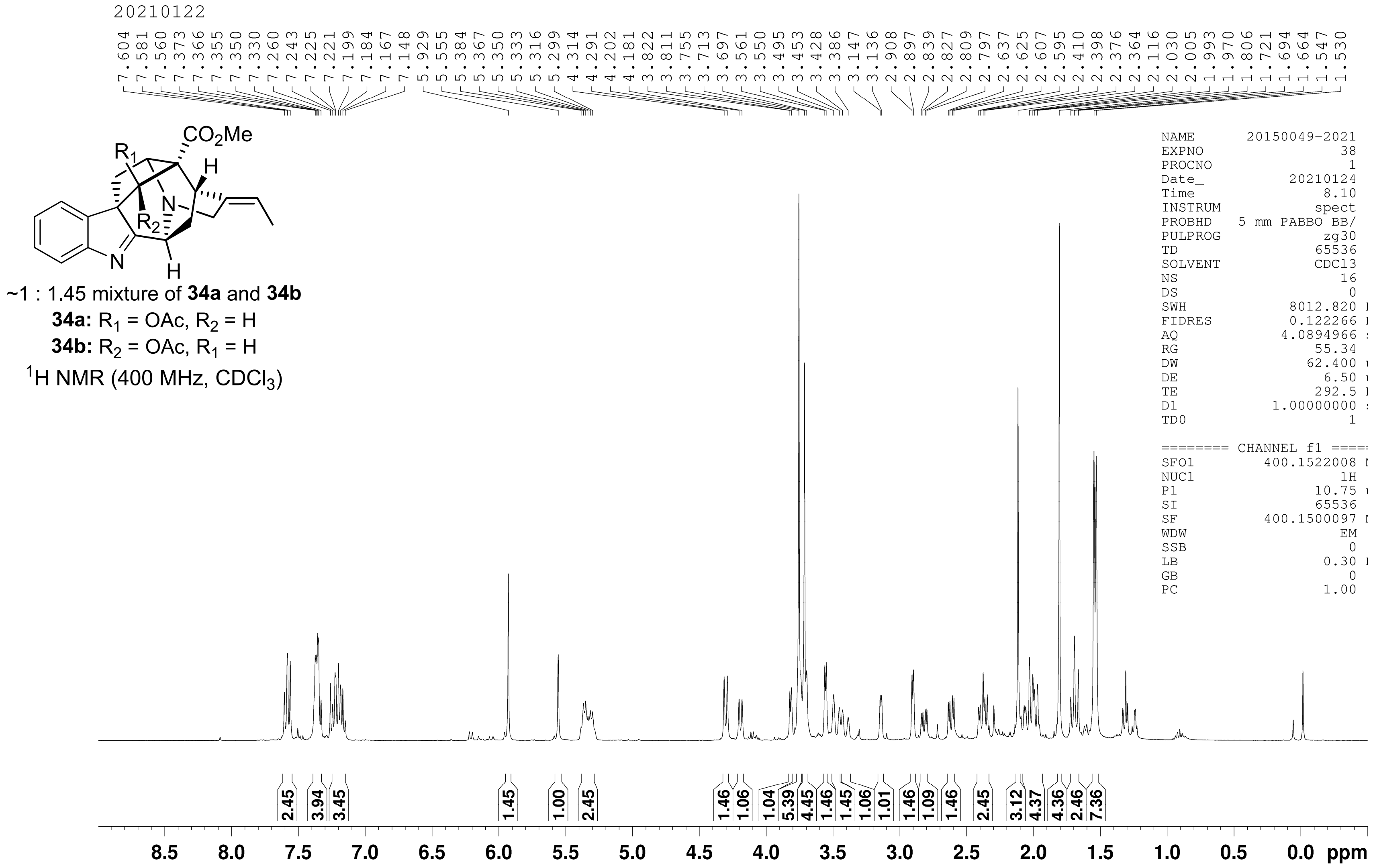

Supplementary Figure 76 ^{1}H-NMR (400 MHz, $CDCl_3$) spectra of the mixture of **34a** and **34b**

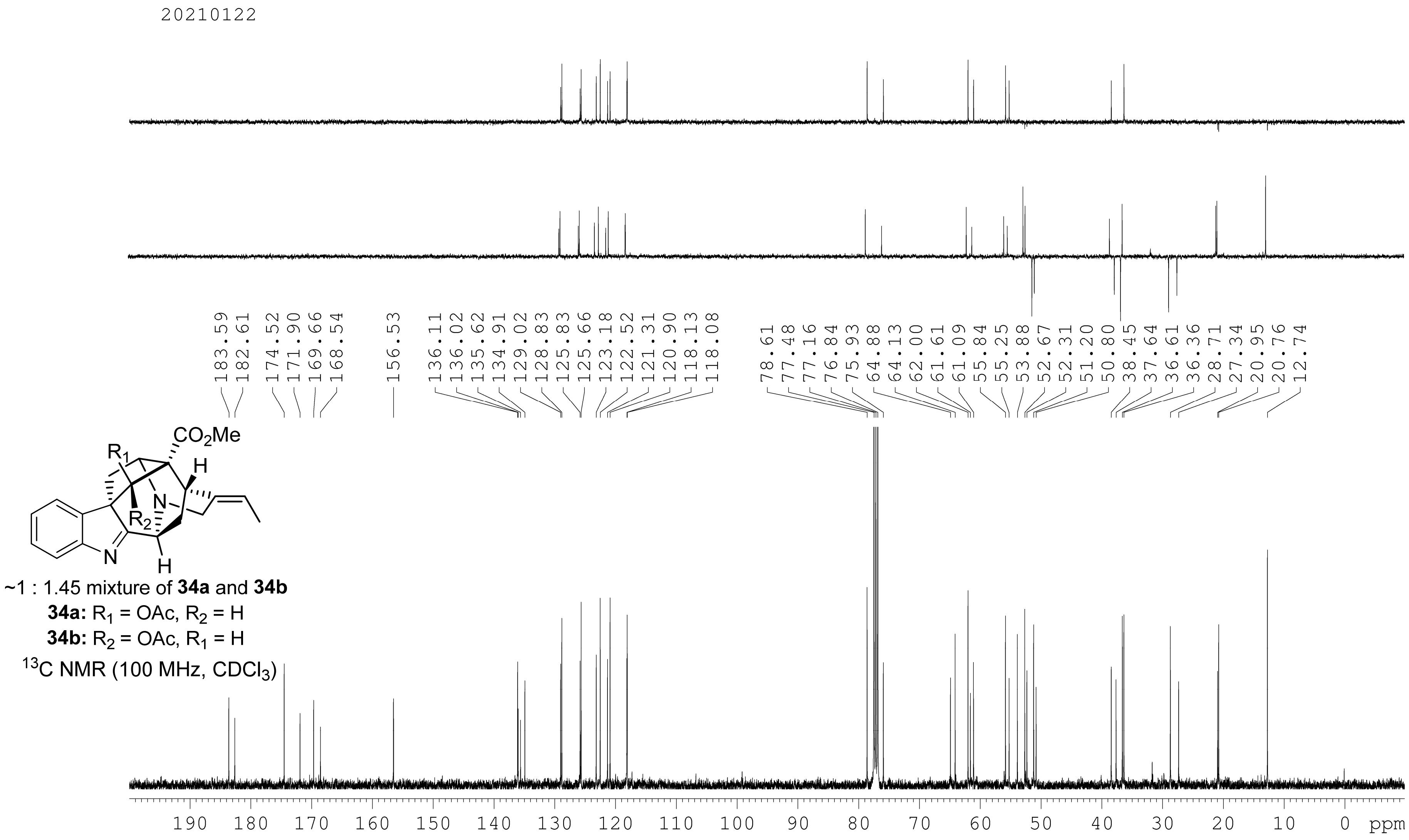

Supplementary Figure 77 ^{13}C-NMR (100 MHz, $CDCl_3$) spectra of the mixture of **34a** and **34b**

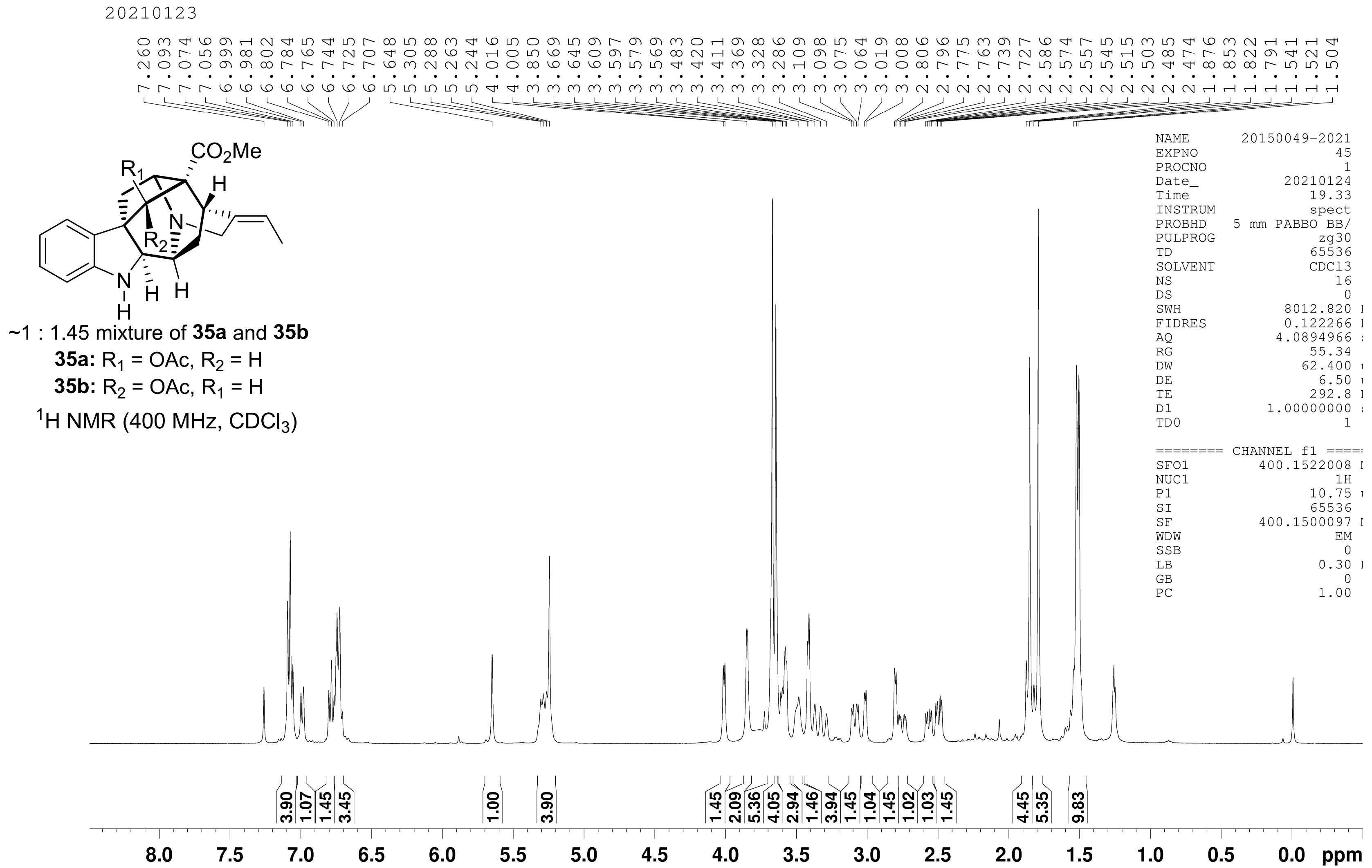

Supplementary Figure 78 ^{1}H-NMR (400 MHz, $CDCl_3$) spectra of the mixture of **35a** and **35b**

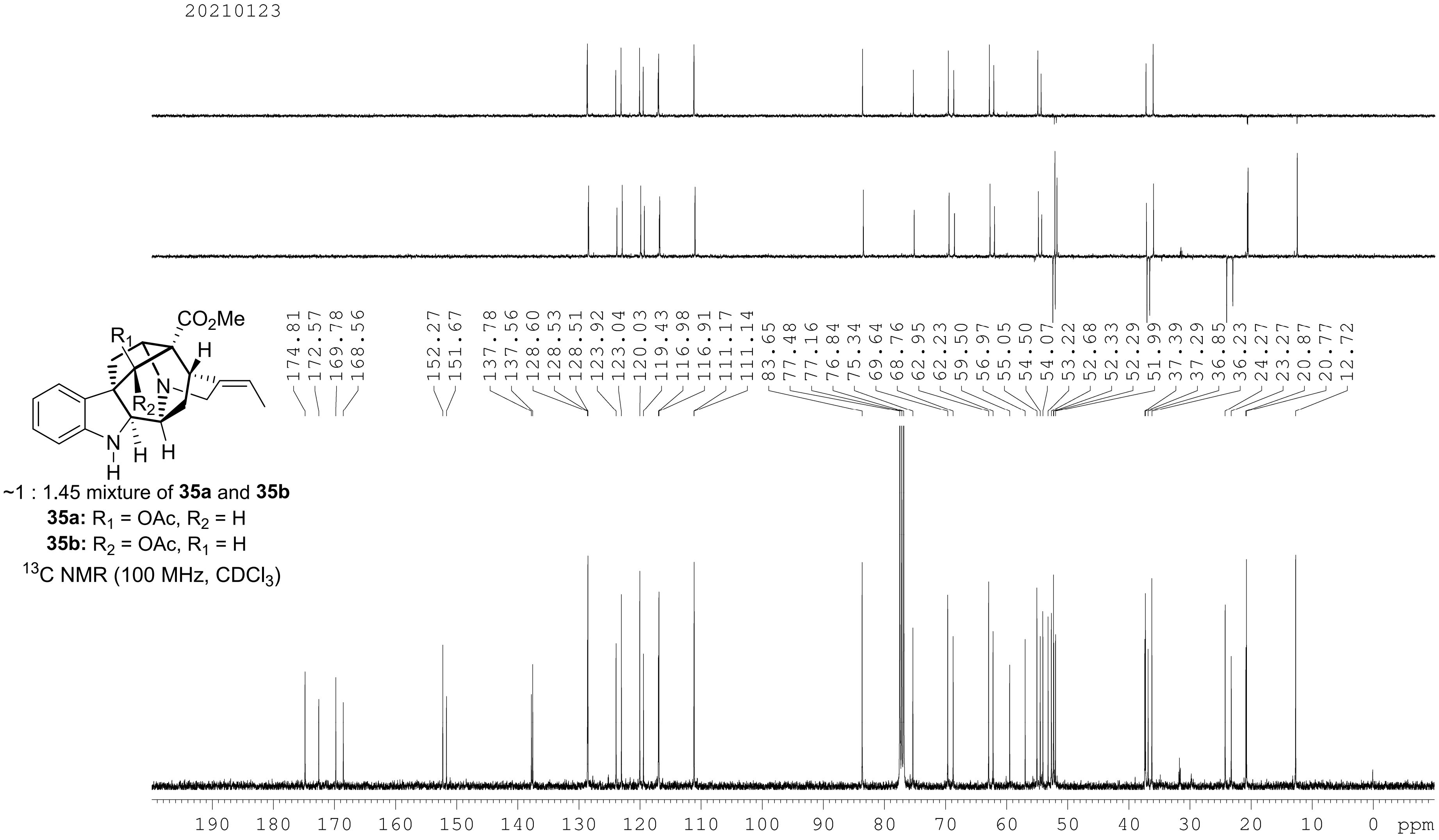

Supplementary Figure 79 ^{13}C-NMR (100 MHz, $CDCl_3$) spectra of the mixture of **35a** and **35b**

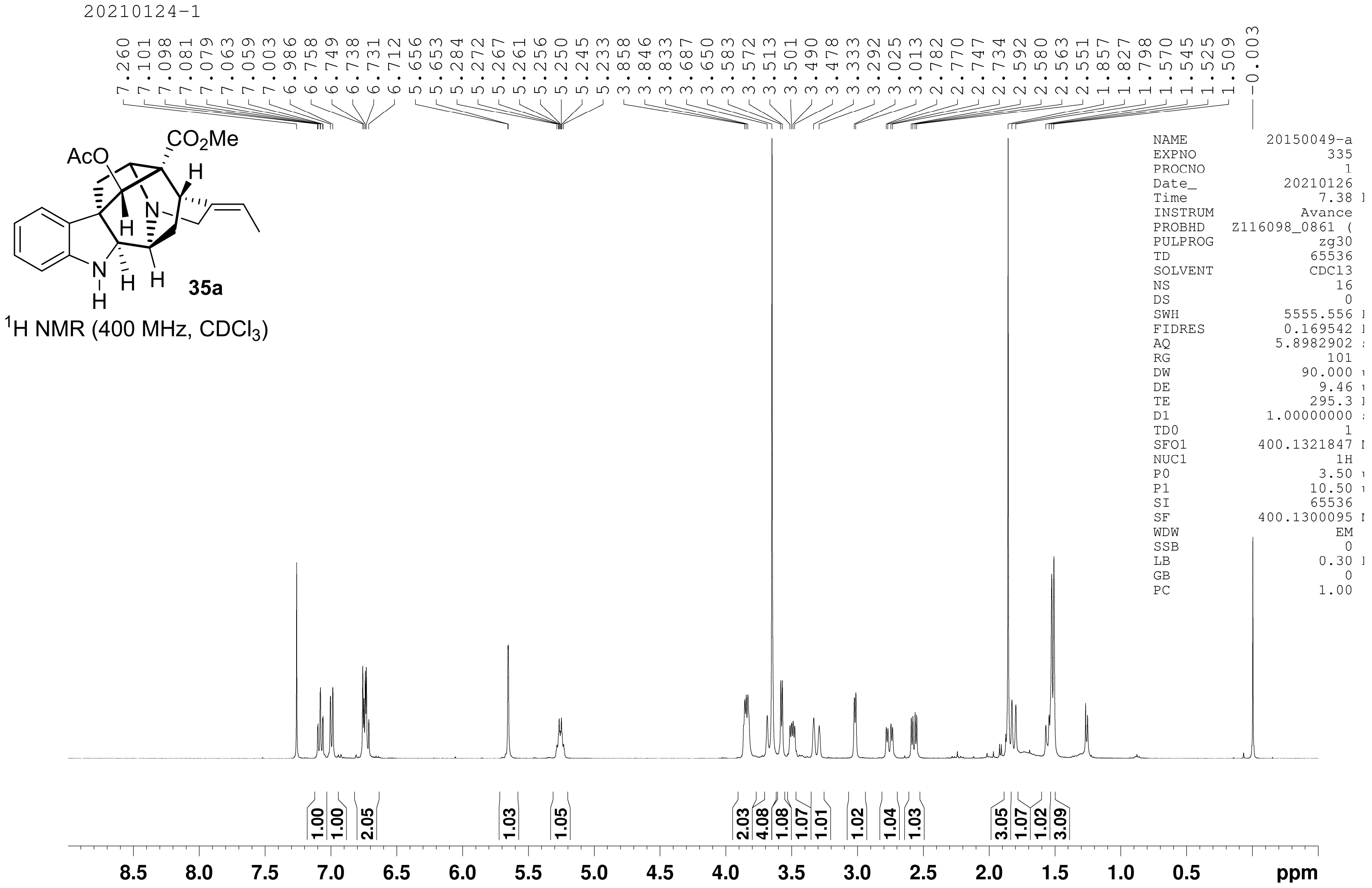

Supplementary Figure 80 ^{1}H-NMR (400 MHz, $CDCl_3$) spectra of **35a**

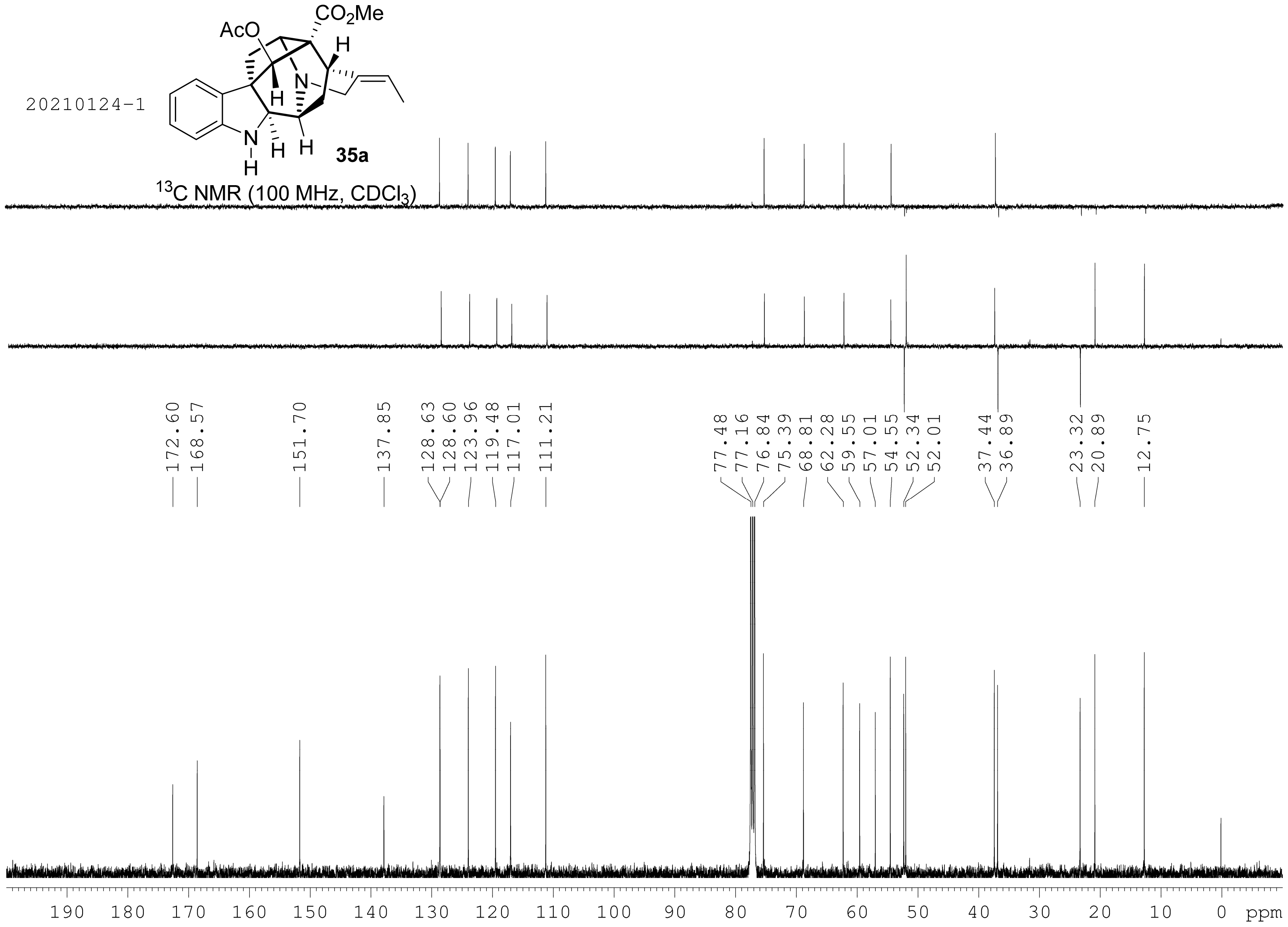

Supplementary Figure 81 ^{13}C-NMR (100 MHz, $CDCl_3$) spectra of **35a**

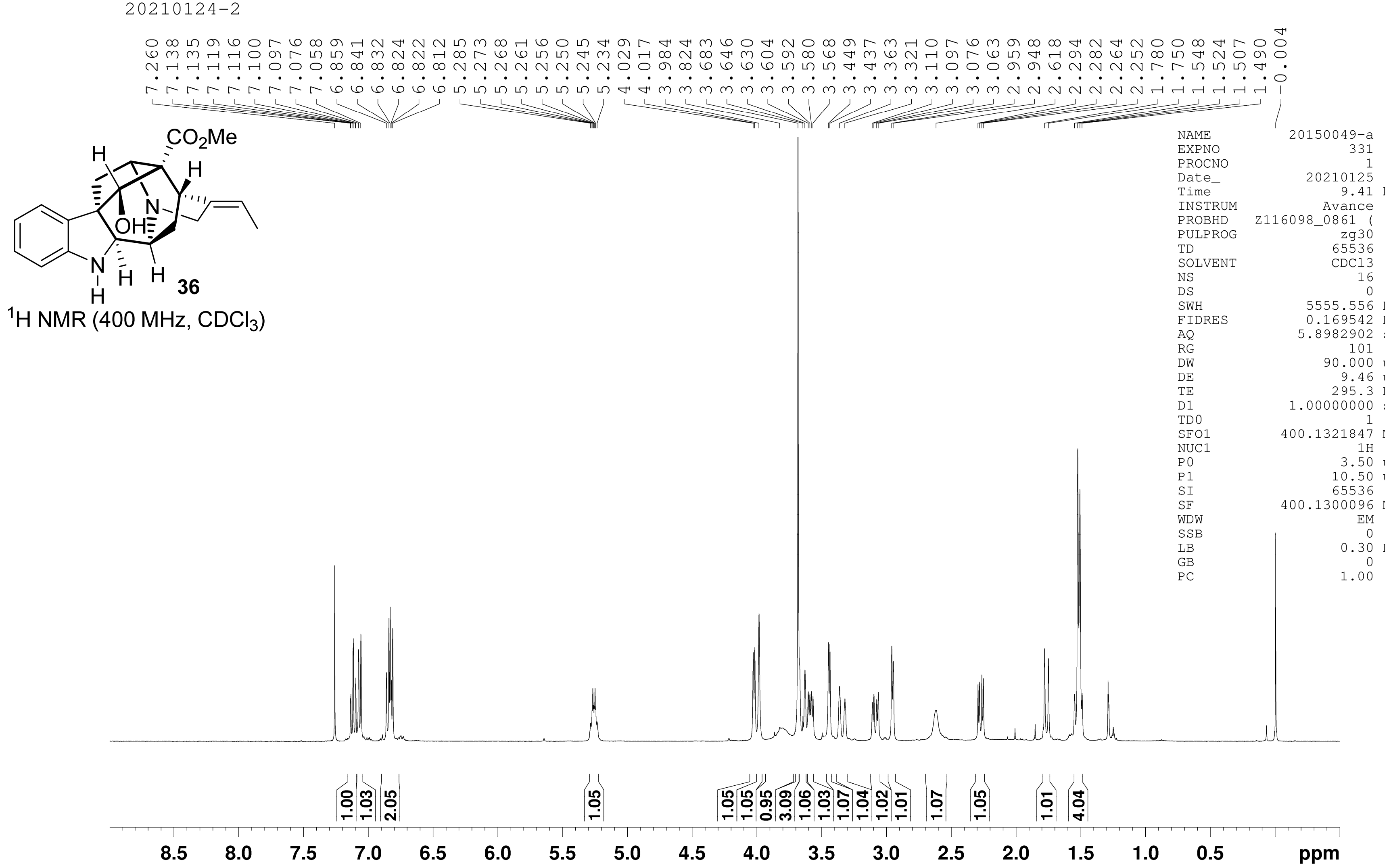

Supplementary Figure 82 ^{1}H-NMR (400 MHz, $CDCl_3$) spectra of **36**

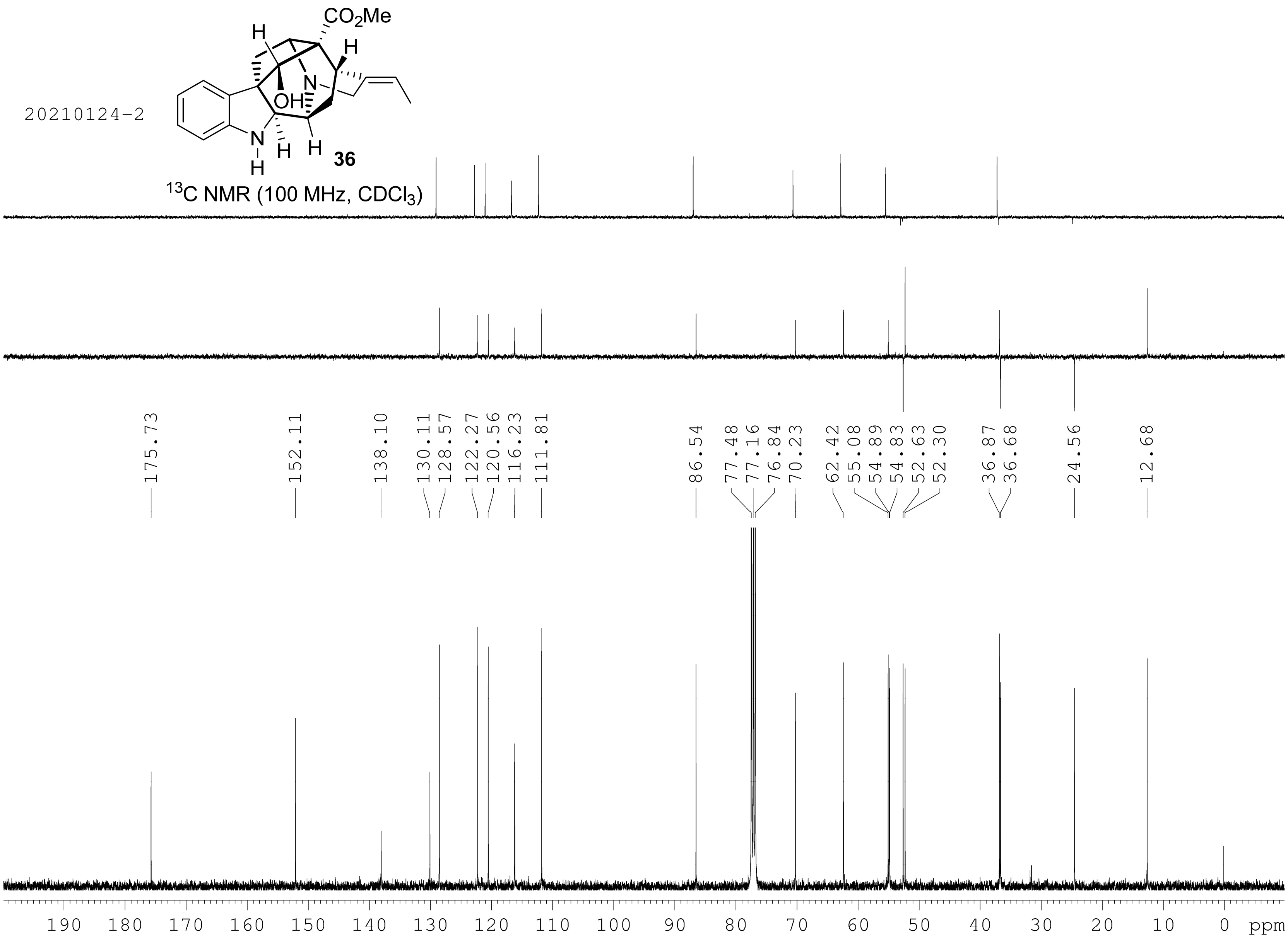

Supplementary Figure 83 ^{13}C-NMR (100 MHz, $CDCl_3$) spectra of **36**

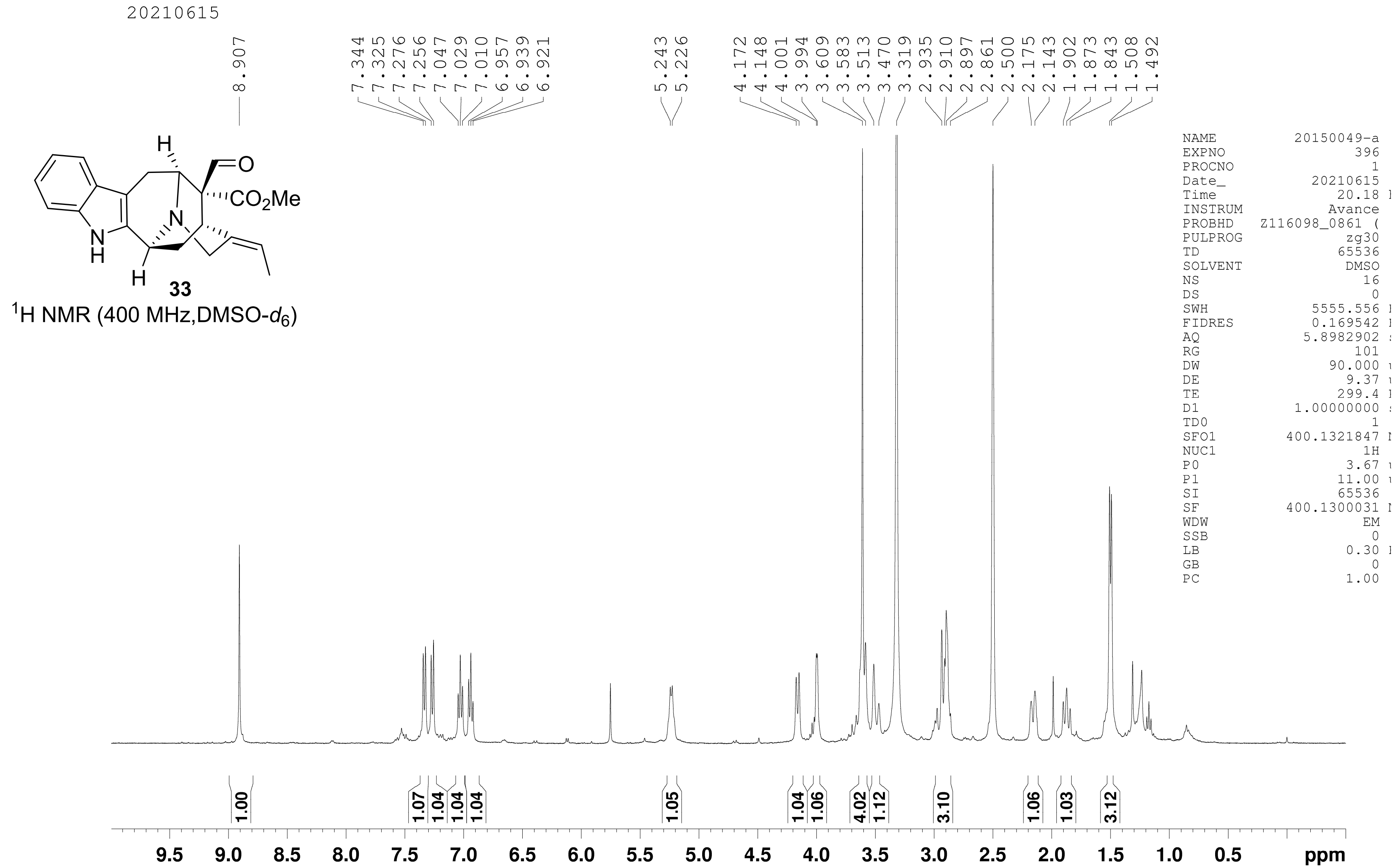

Supplementary Figure 84 ^{1}H-NMR (400 MHz, DMSO-d_6) spectra of crude **33**

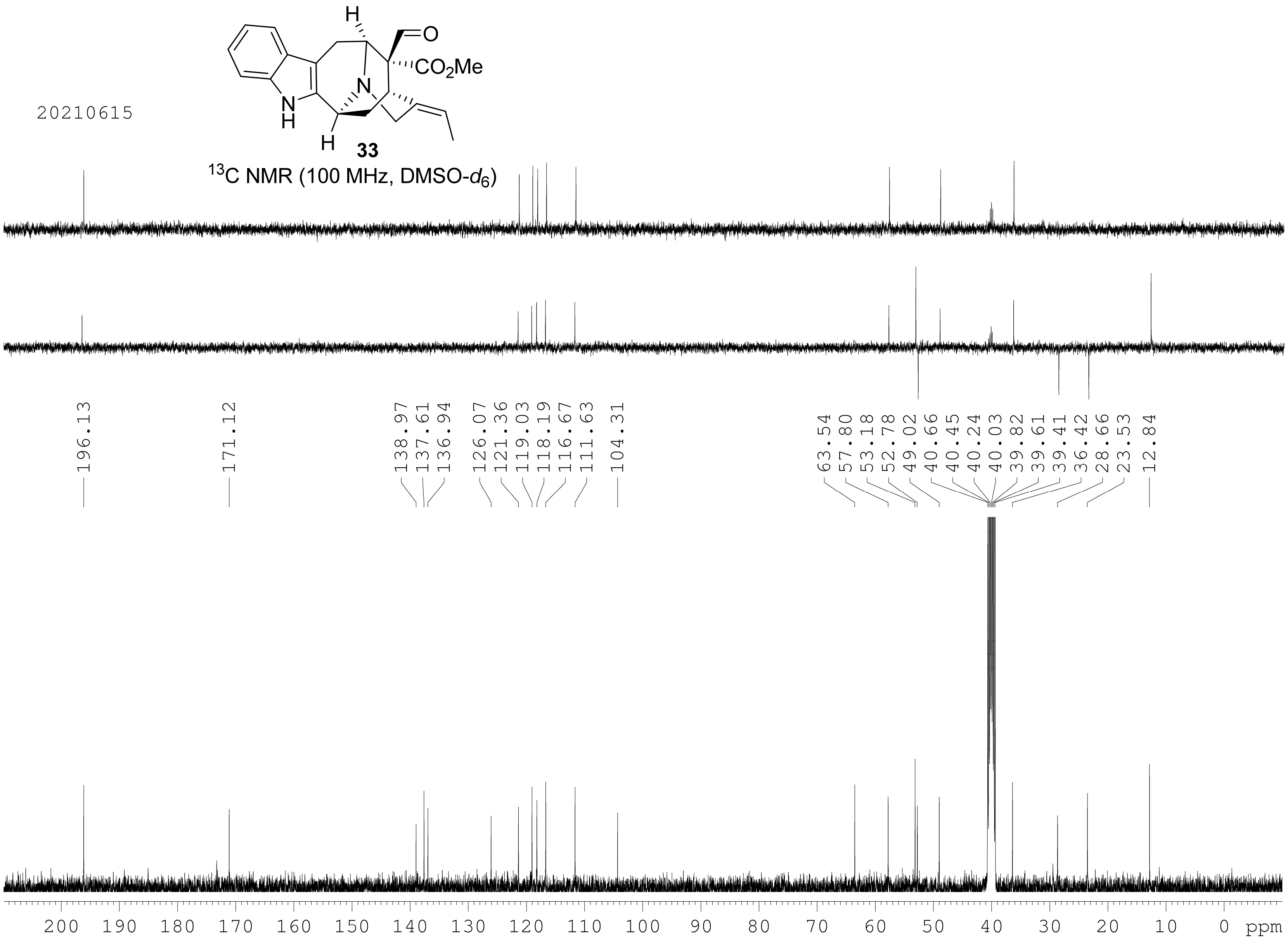

Supplementary Figure 85 ^{13}C-NMR (100 MHz, DMSO-d_6) spectra of crude **33**

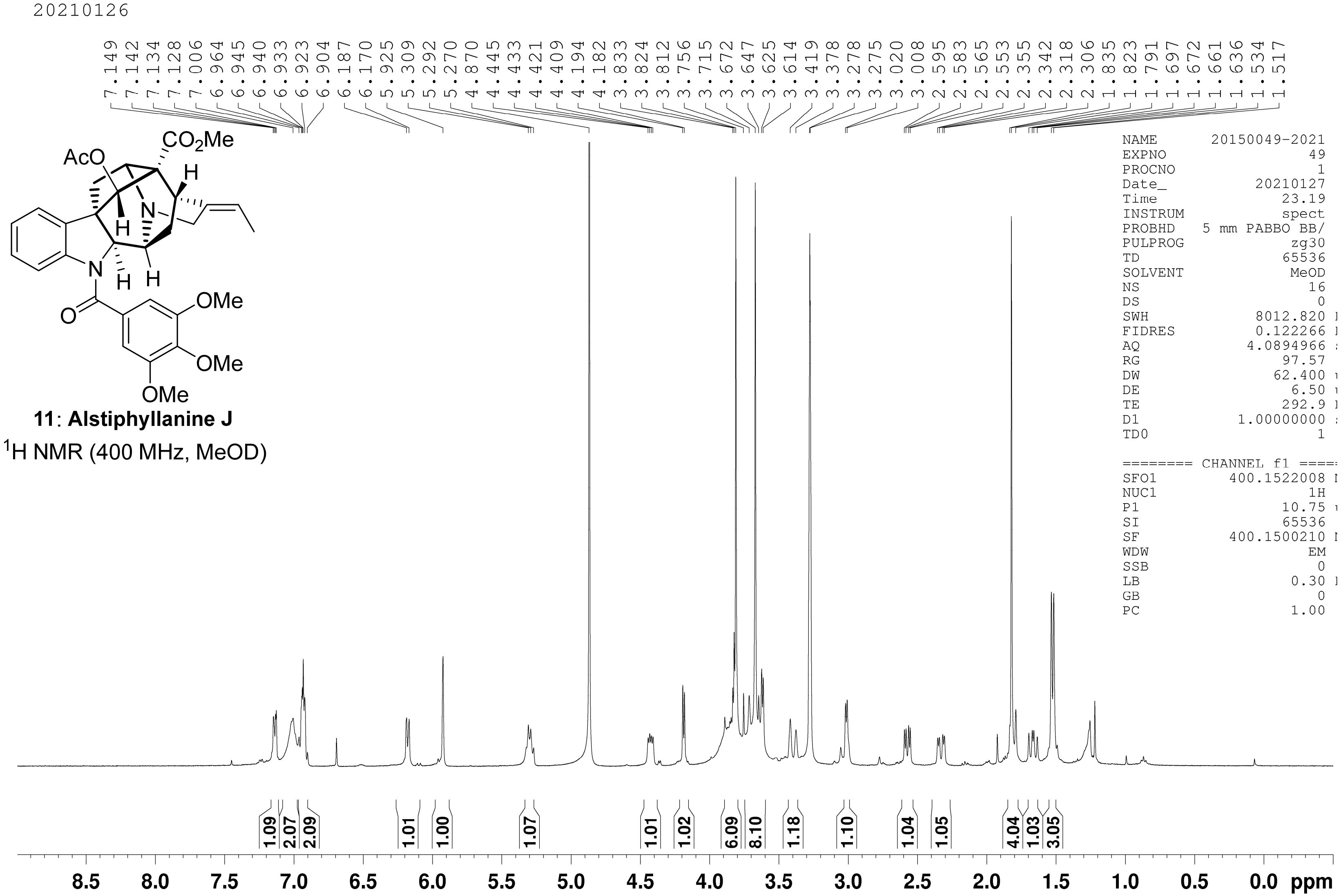

Supplementary Figure 86 ^{1}H-NMR (400 MHz, MeOD) spectra of Alstiphyllanine J (**11**)

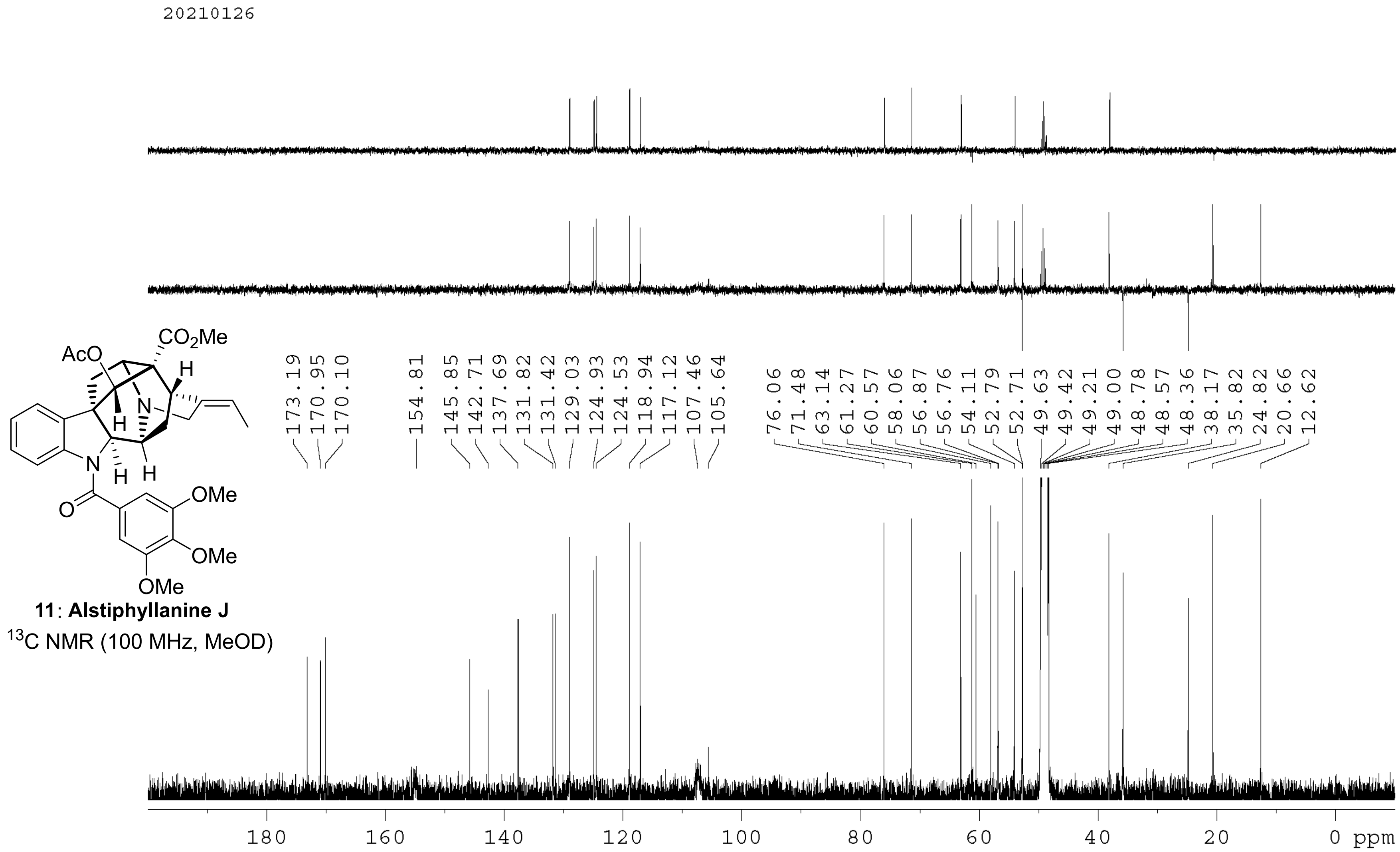

Supplementary Figure 87 ^{13}C-NMR (100 MHz, MeOD) spectra of Alstiphyllanine J (**11**)

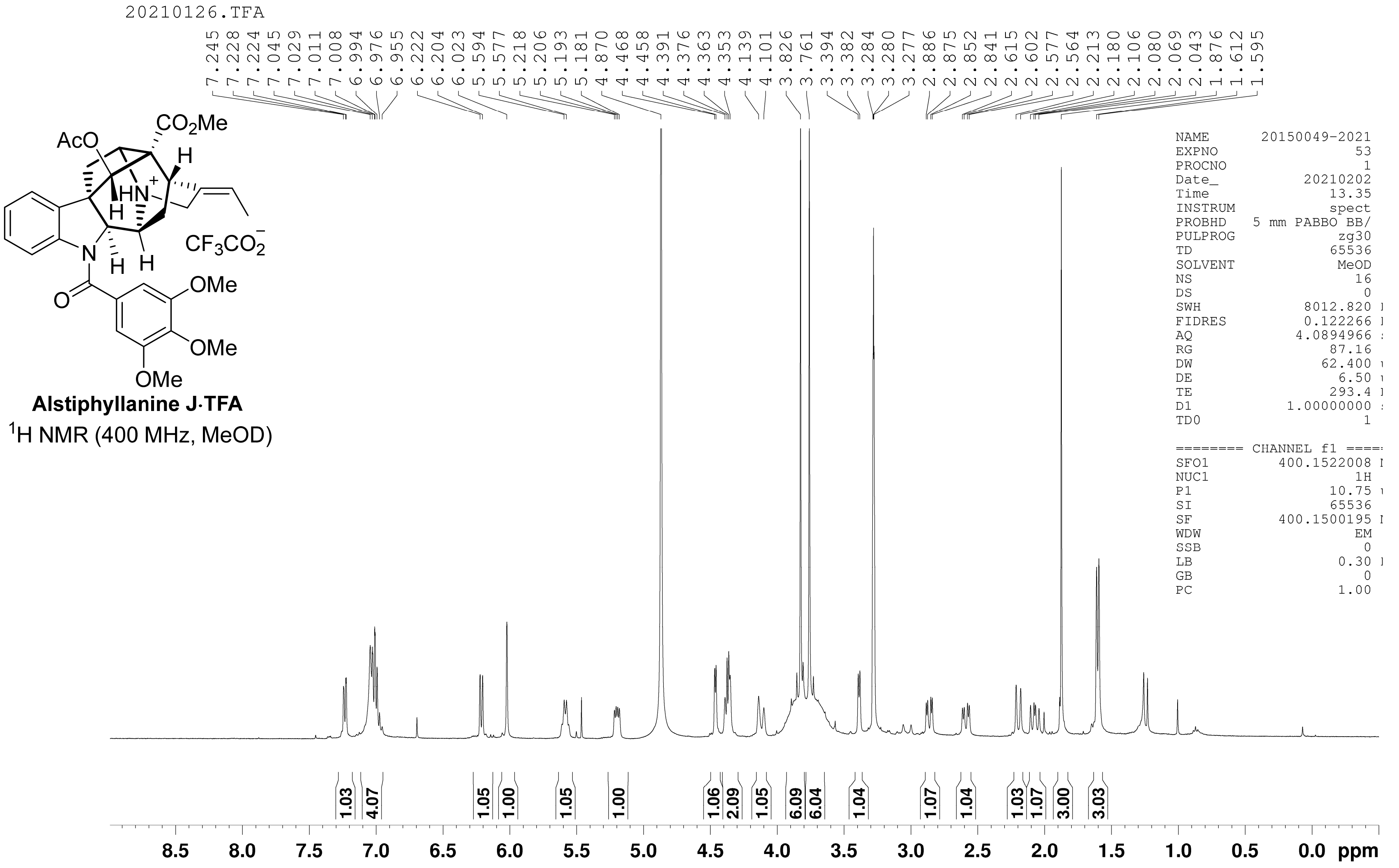

Supplementary Figure 88 ^{1}H-NMR (400 MHz, MeOD) spectra of Alstiphyllanine J·TFA

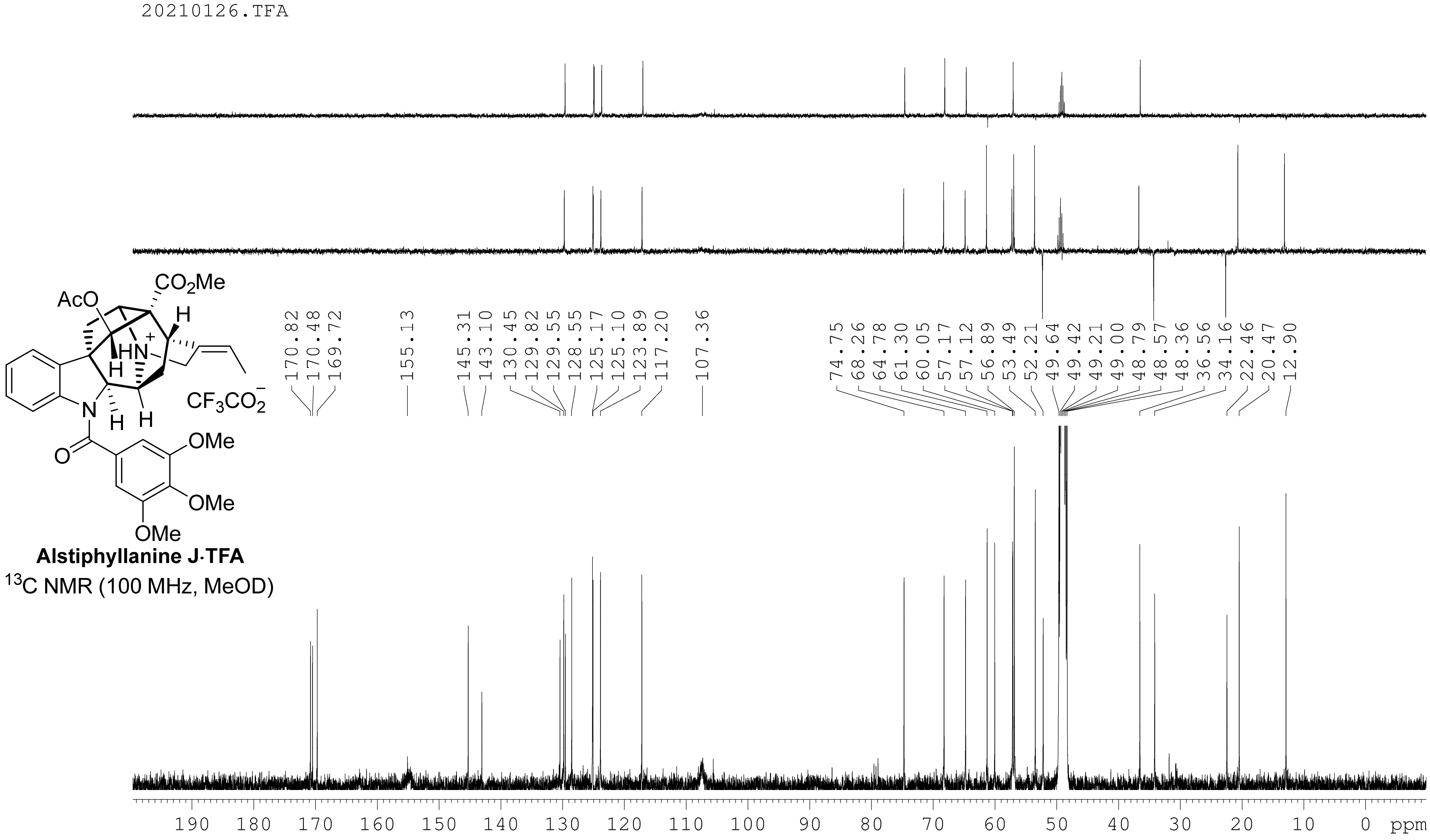

Supplementary Figure 89 ^{13}C-NMR (100 MHz, MeOD) spectra of Alstiphyllanine J·TFA

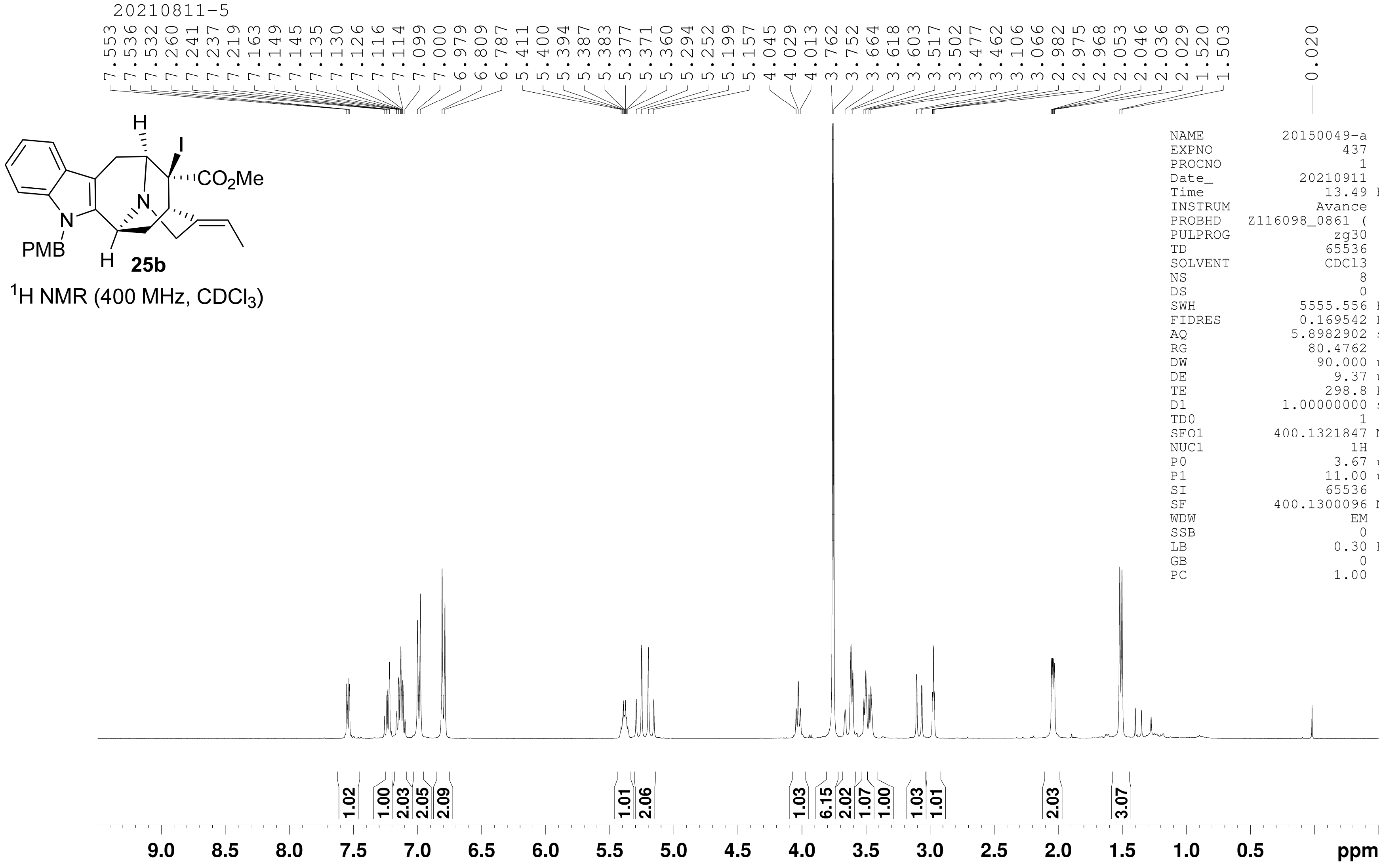

Supplementary Figure 90 ^{1}H-NMR (400 MHz, $CDCl_3$) spectra of **25b**

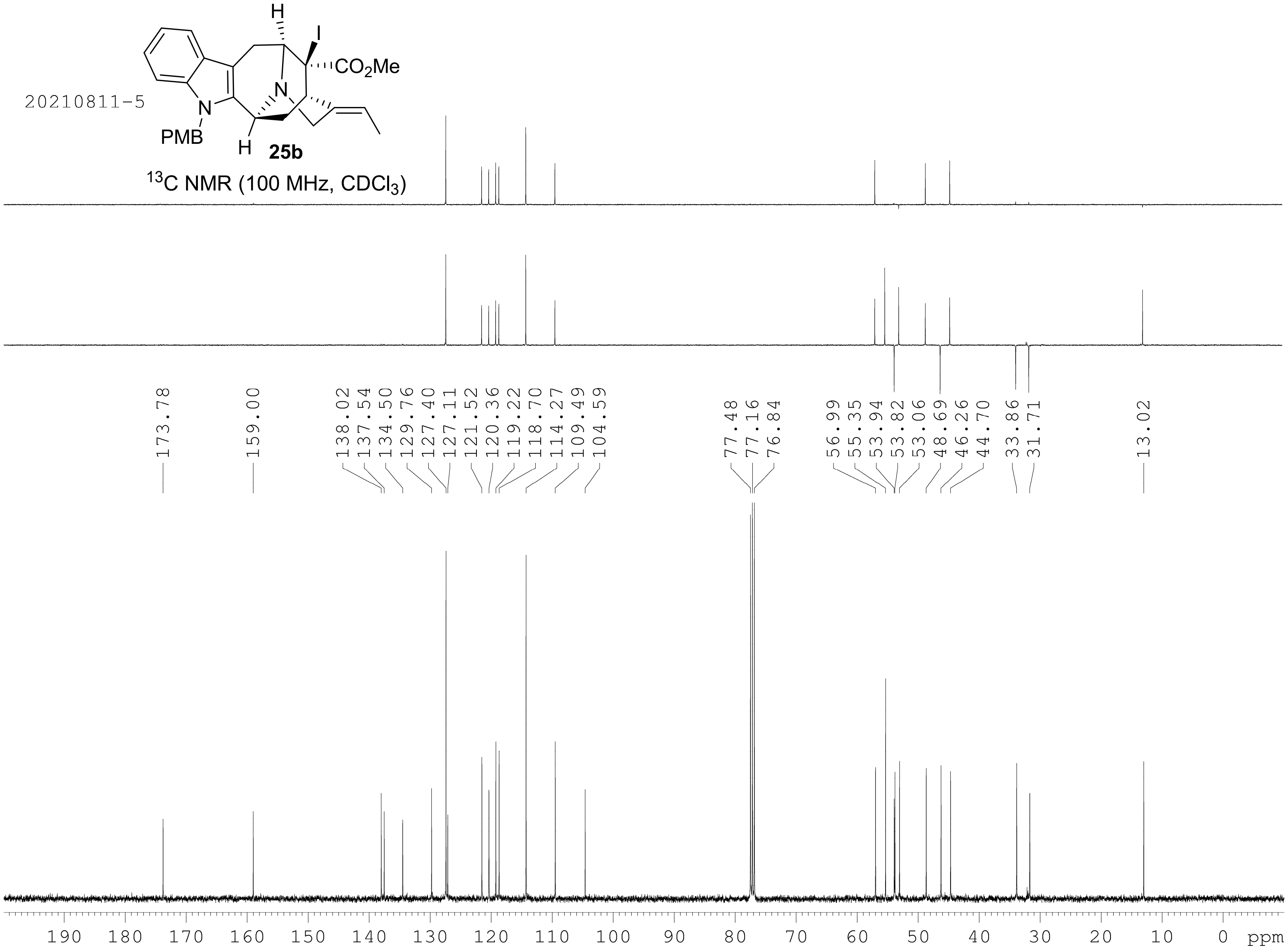

Supplementary Figure 91 ^{13}C-NMR (100 MHz, CDCl$_3$) spectra of **25b**

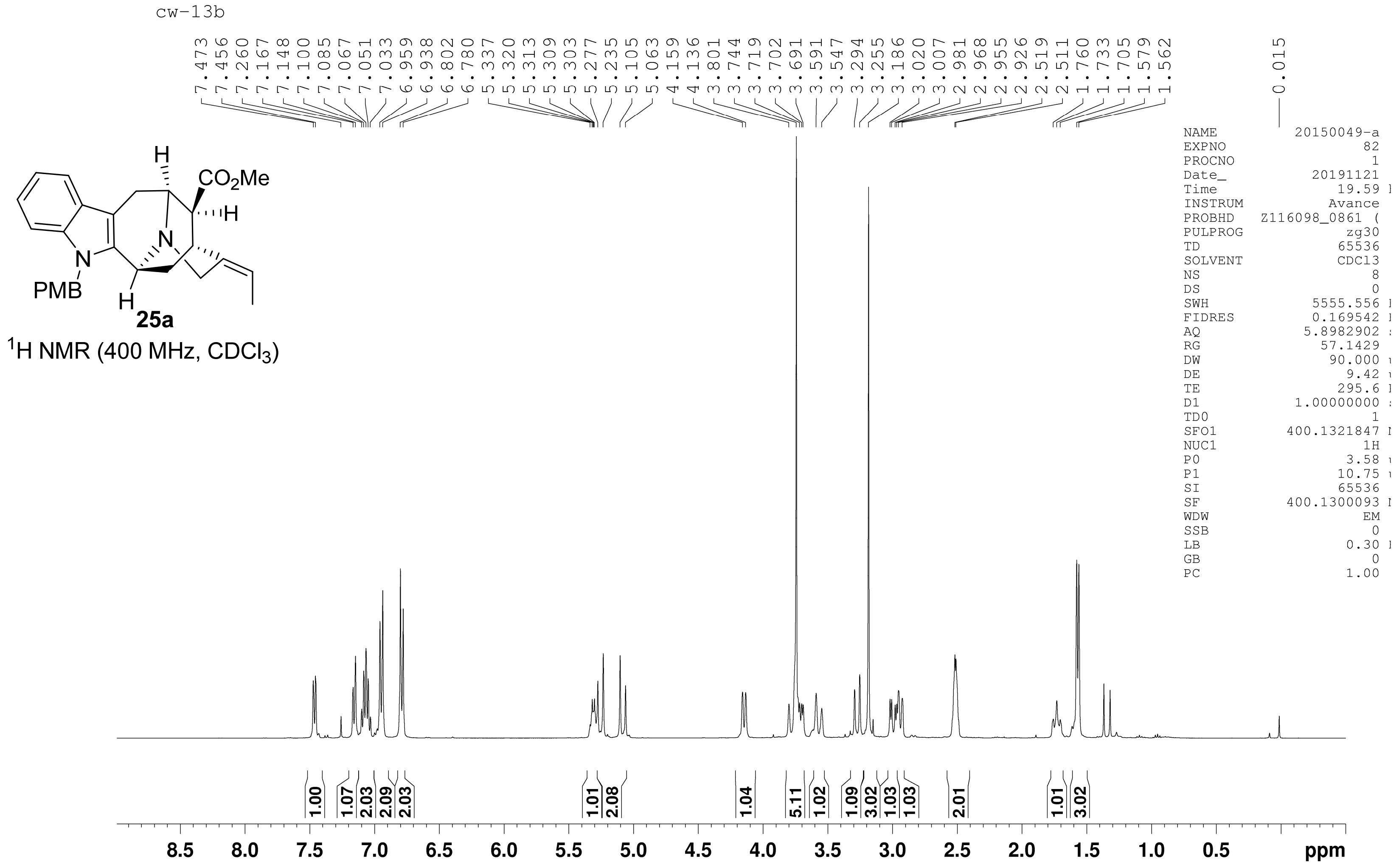

Supplementary Figure 92 ^{1}H-NMR (400 MHz, $CDCl_3$) spectra of **25a**

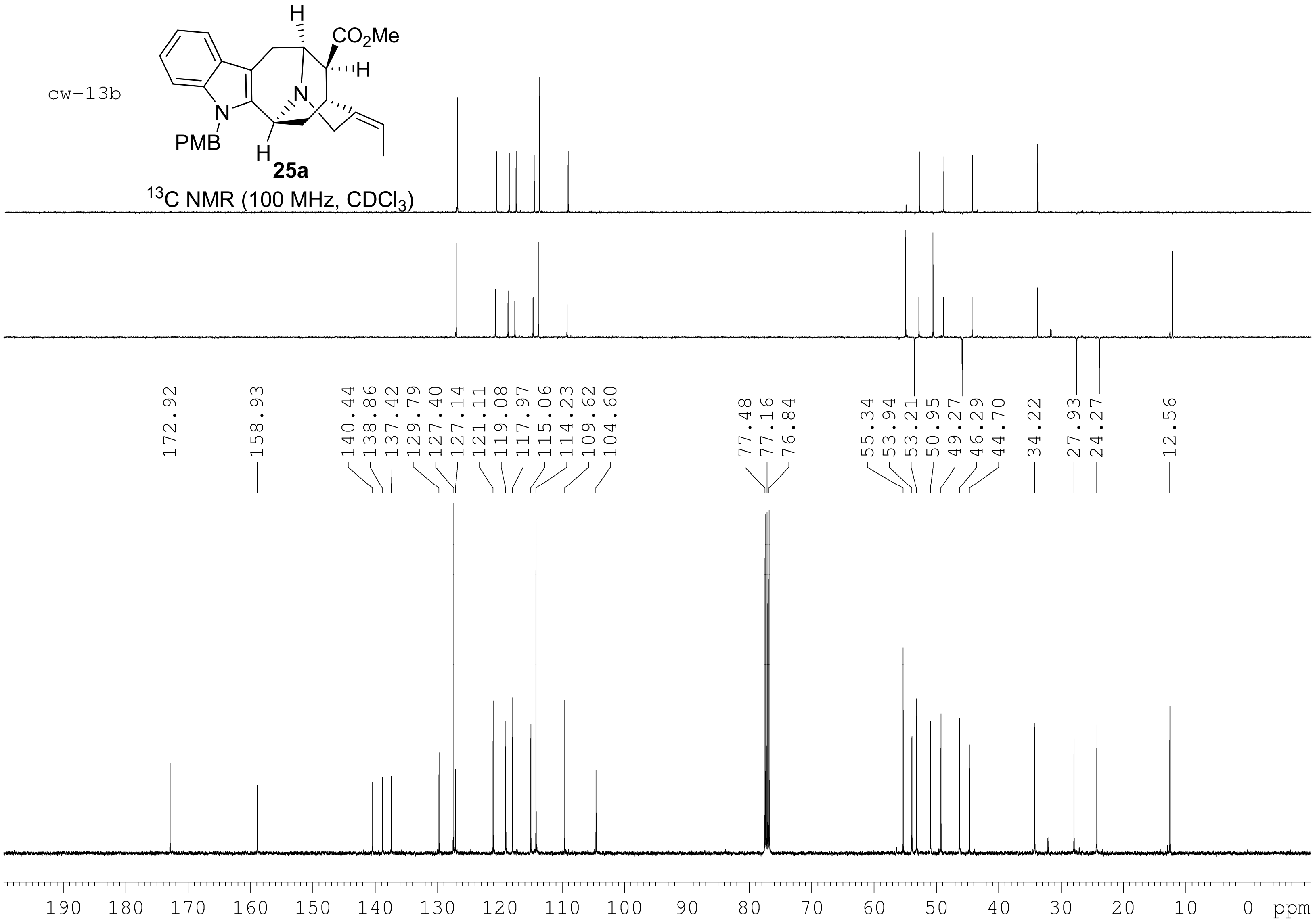

Supplementary Figure 93 ^{13}C-NMR (100 MHz, $CDCl_3$) spectra of **25a**

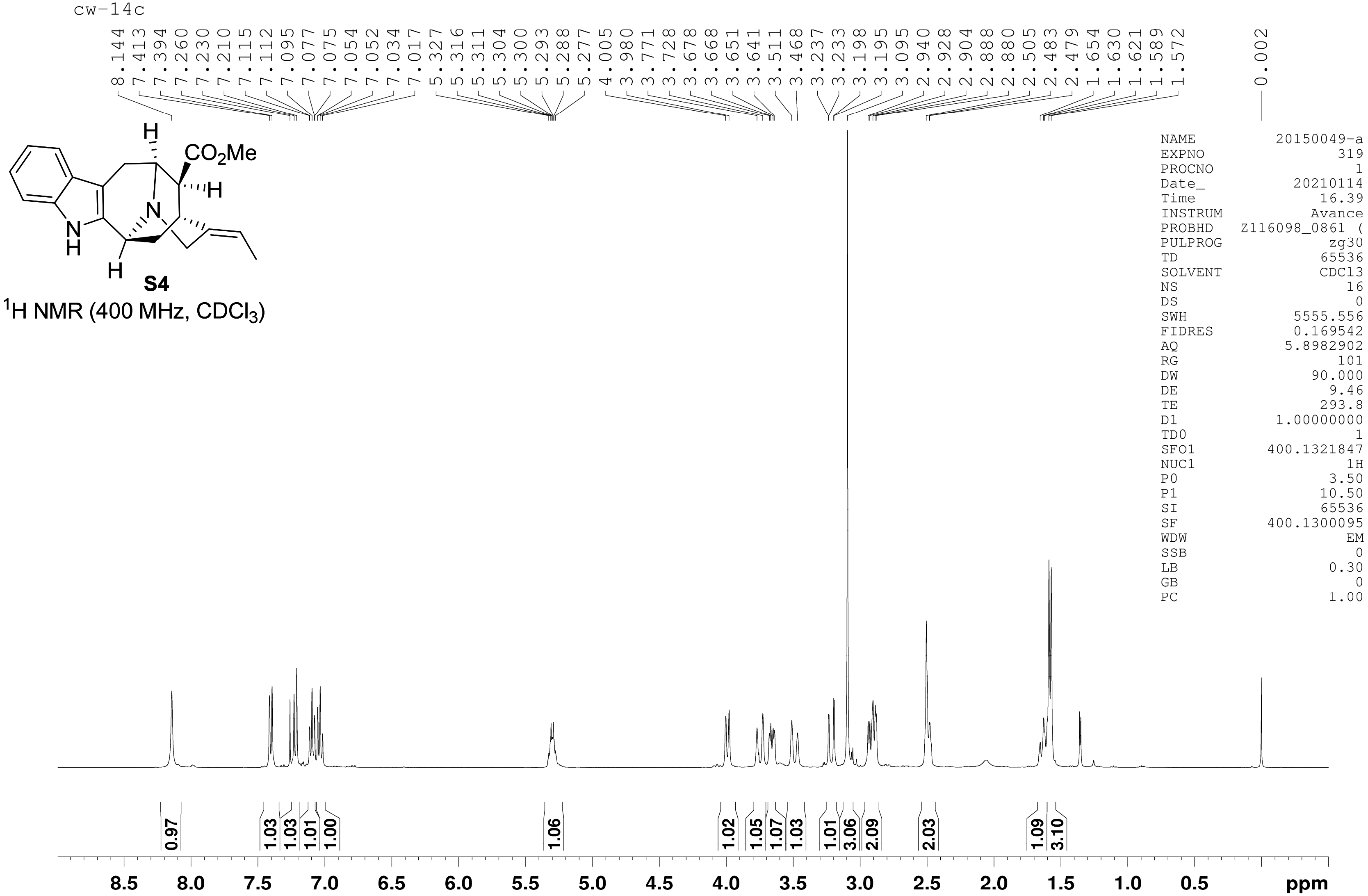

Supplementary Figure 94 ^{1}H-NMR (400 MHz, $CDCl_3$) spectra of **S4**

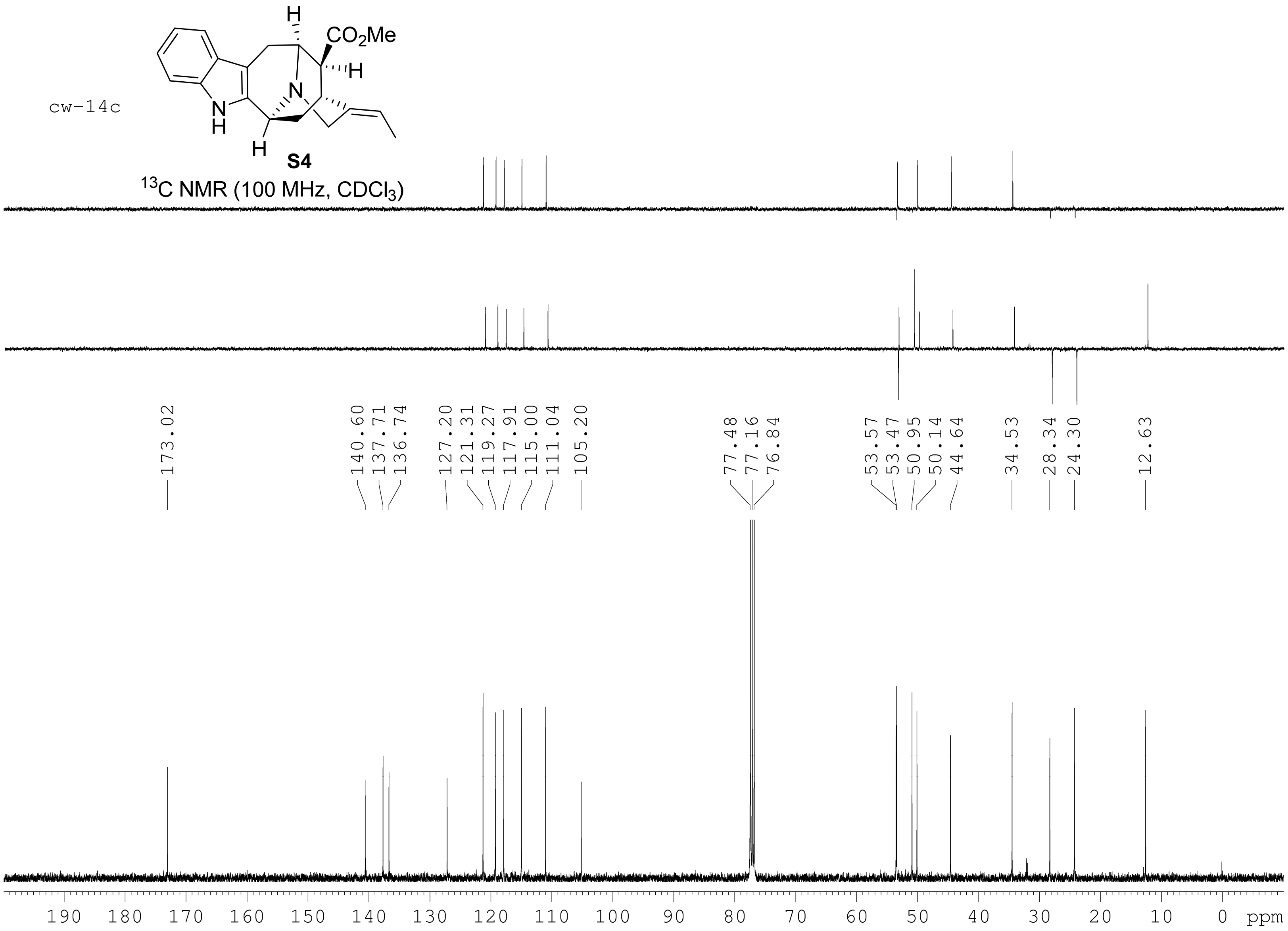

Supplementary Figure 95 ^{13}C-NMR (100 MHz, $CDCl_3$) spectra of **S4**

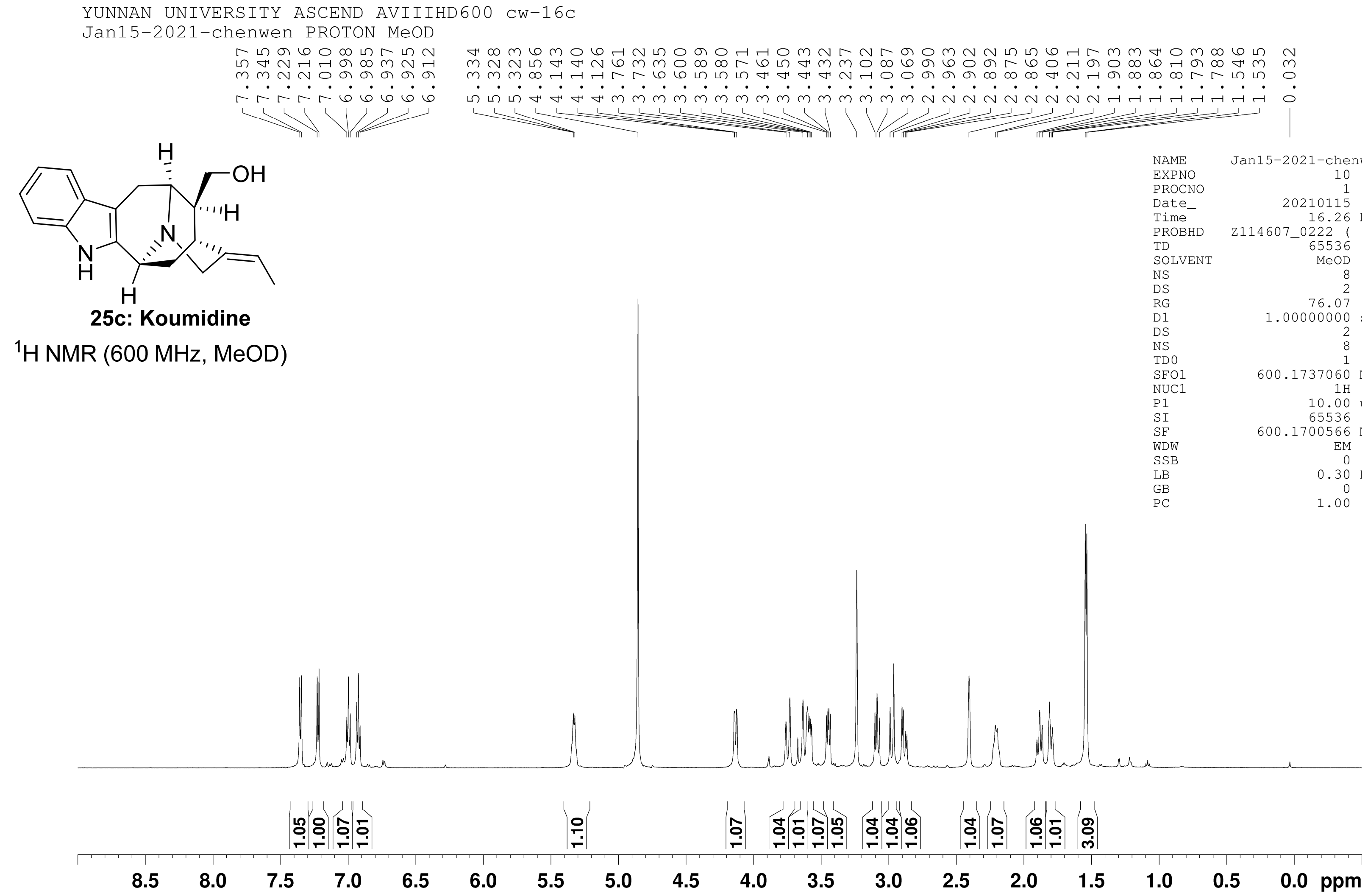

Supplementary Figure 96 ^{1}H-NMR (400 MHz, MeOD) spectra of Koumidine (**25c**)

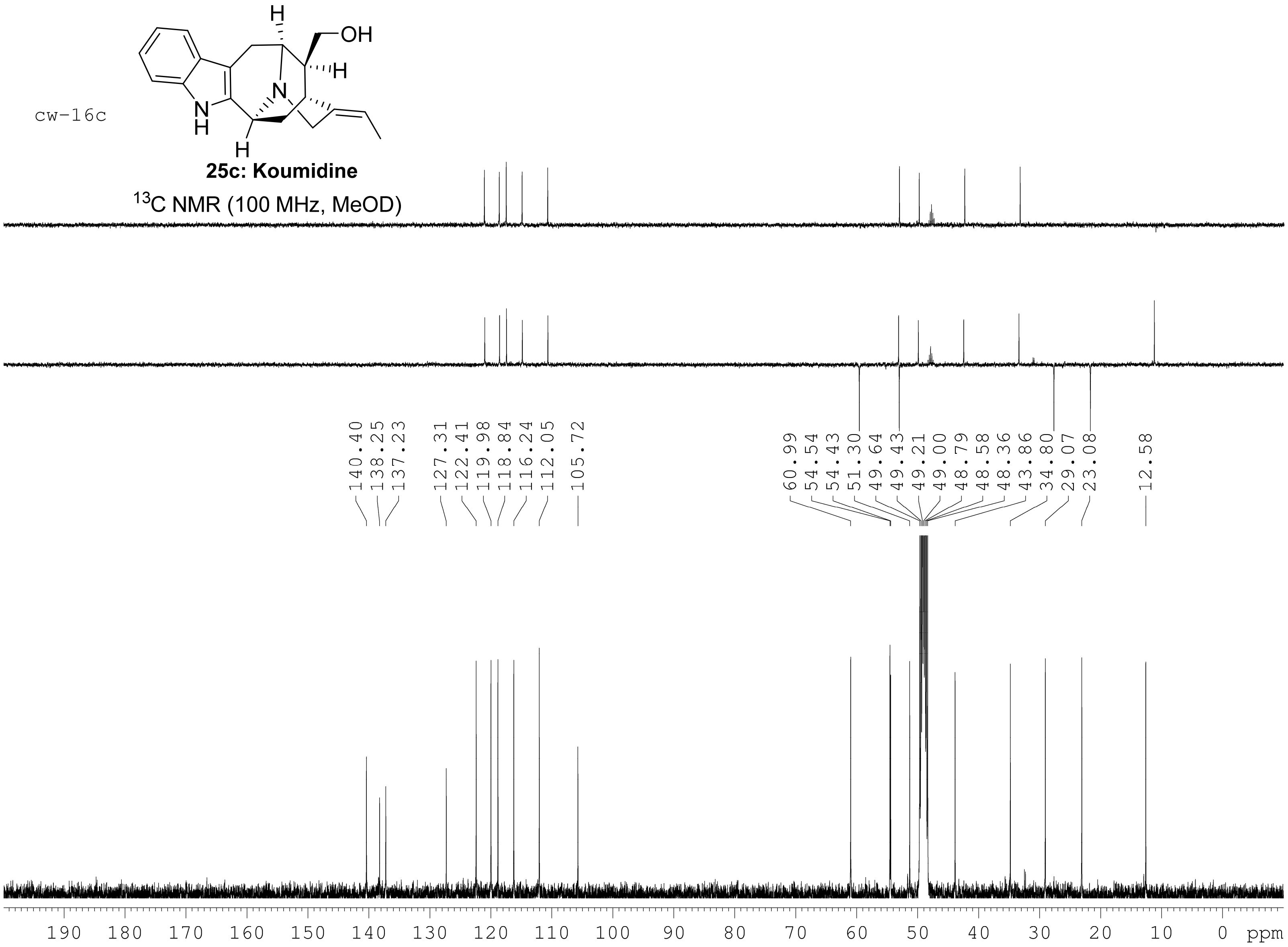

Supplementary Figure 97 ^{13}C-NMR (100 MHz, MeOD) spectra of Koumidine (**25c**)

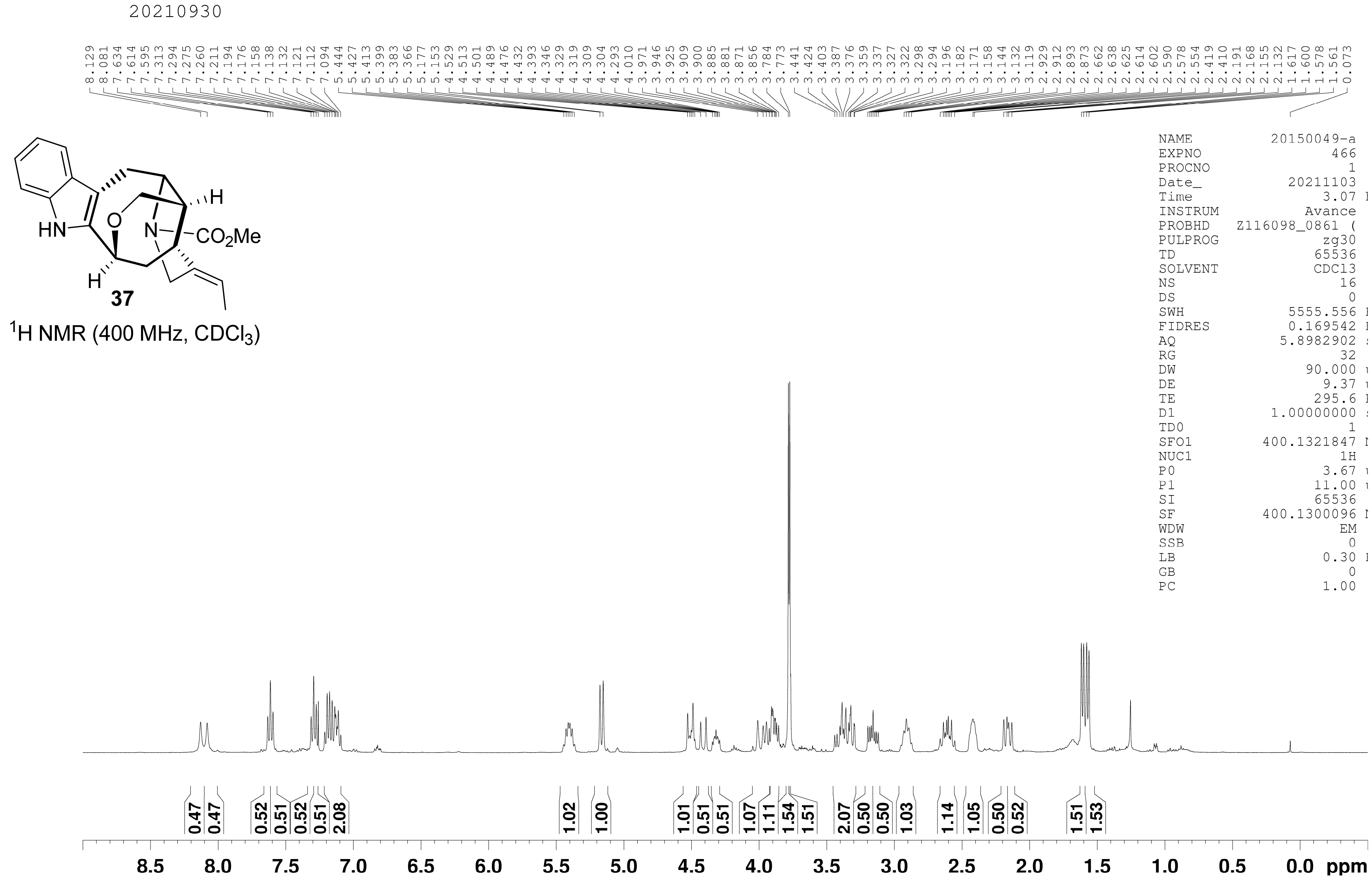

Supplementary Figure 98 ^{1}H-NMR (400 MHz, $CDCl_3$) spectra of **37**

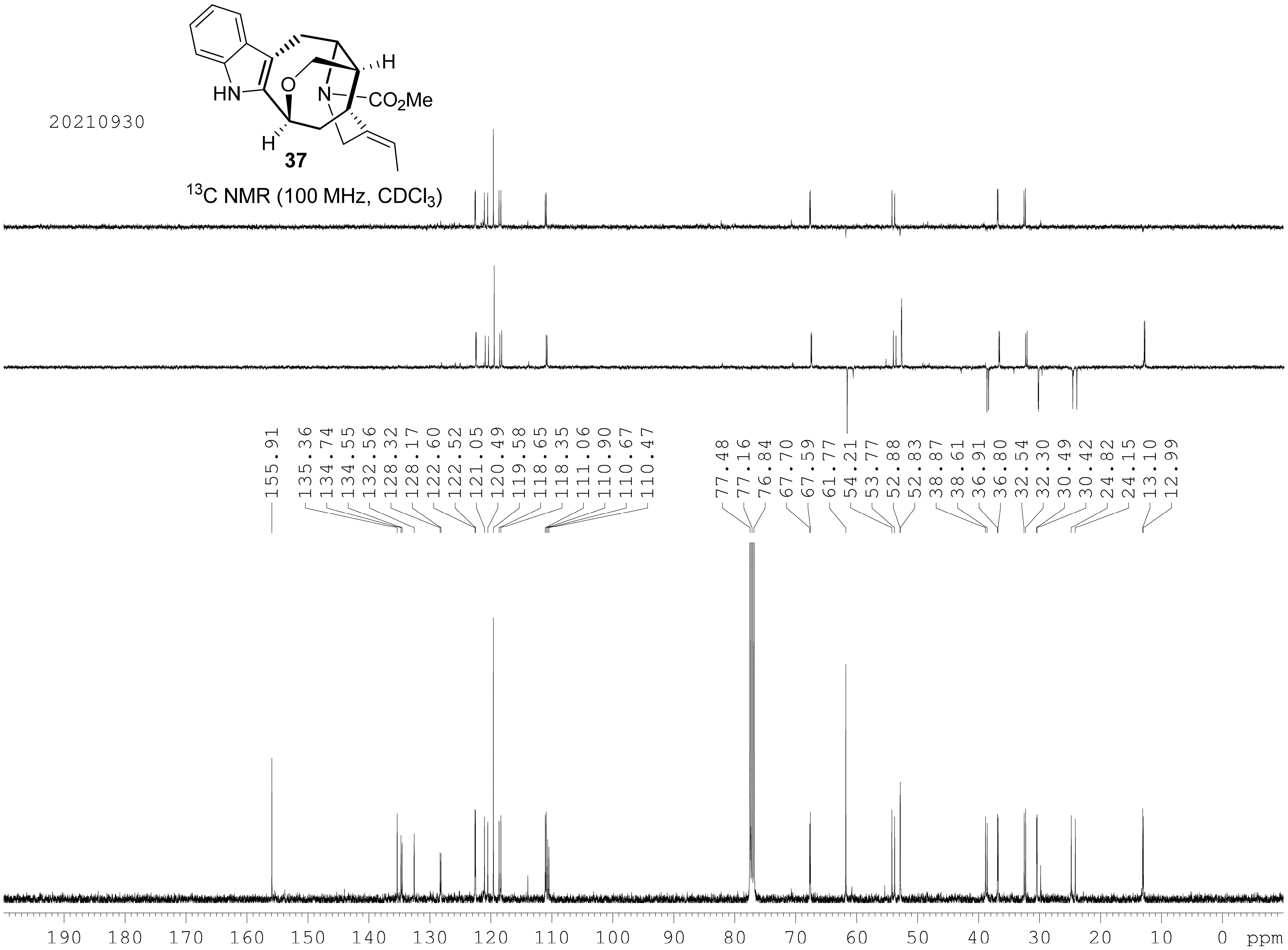

Supplementary Figure 99 ^{13}C-NMR (100 MHz, CDCl$_3$) spectra of **37**

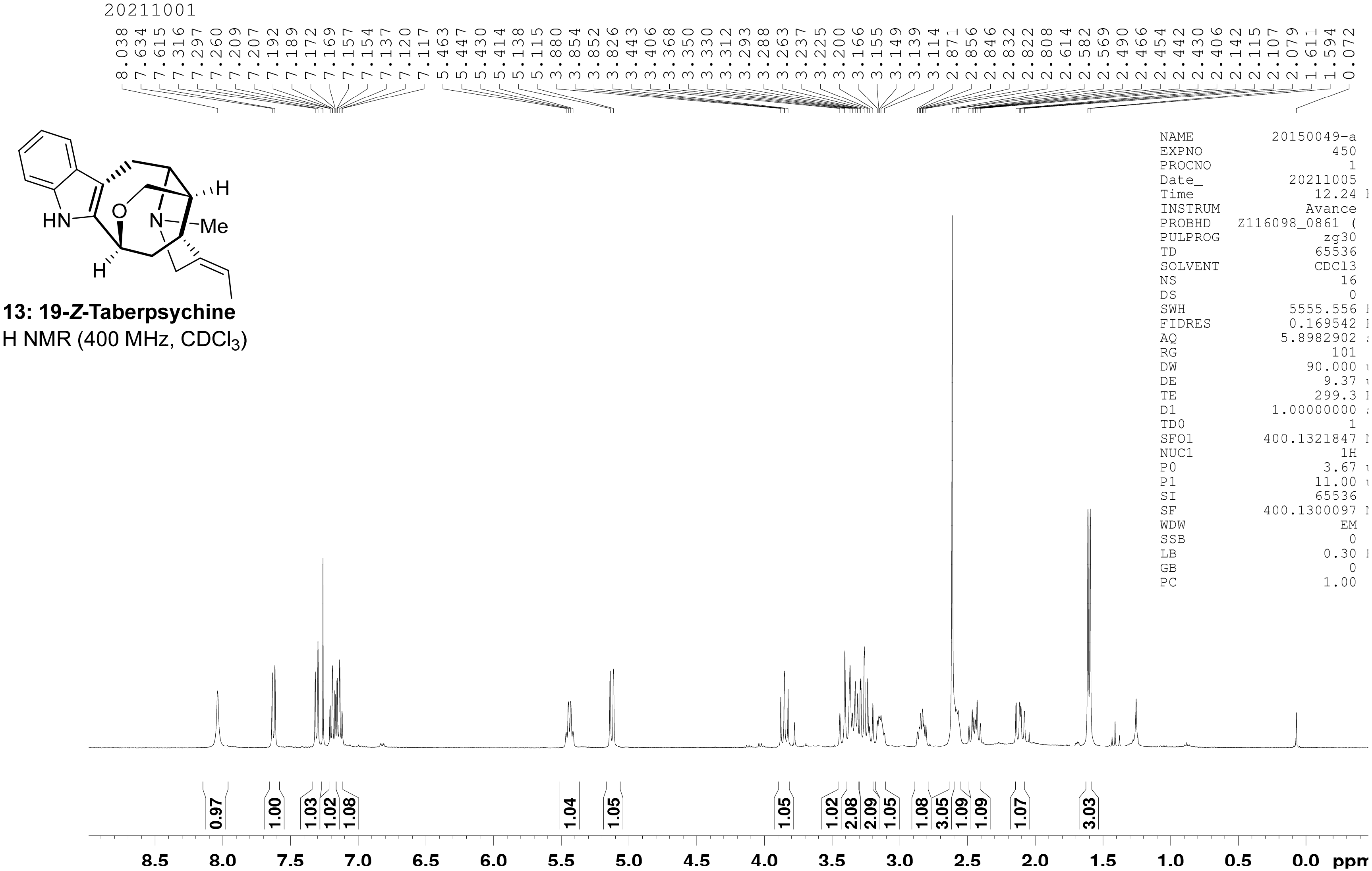

13: 19-*Z*-Taberpsychine
^{1}H NMR (400 MHz, $CDCl_3$)

Supplementary Figure 100 ^{1}H-NMR (400 MHz, $CDCl_3$) spectra of 19-*Z*-Taberpsychine (**13**)

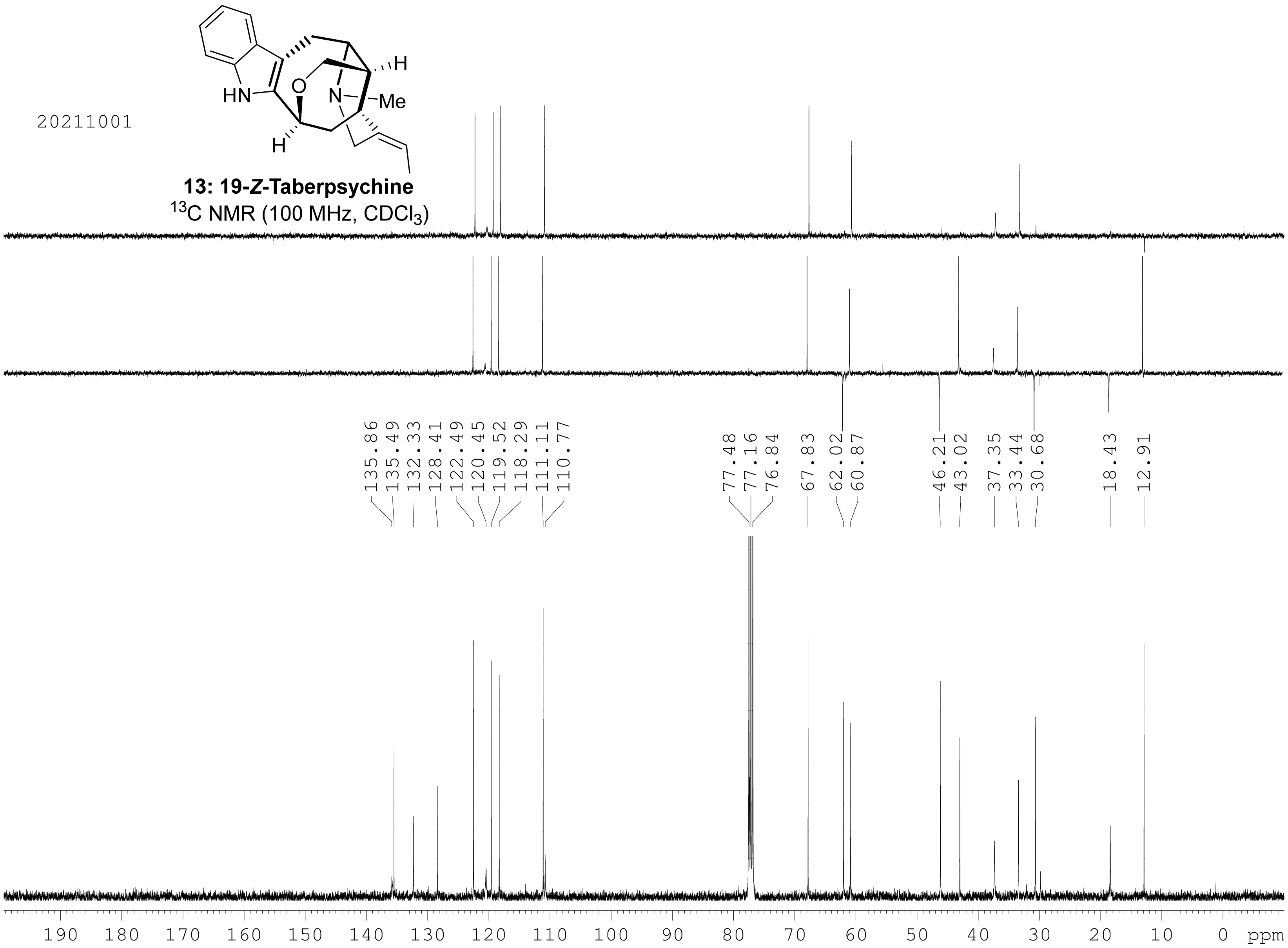

Supplementary Figure 101 ^{13}C-NMR (100 MHz, $CDCl_3$) spectra of 19-*Z*-Taberpsychine (**13**)

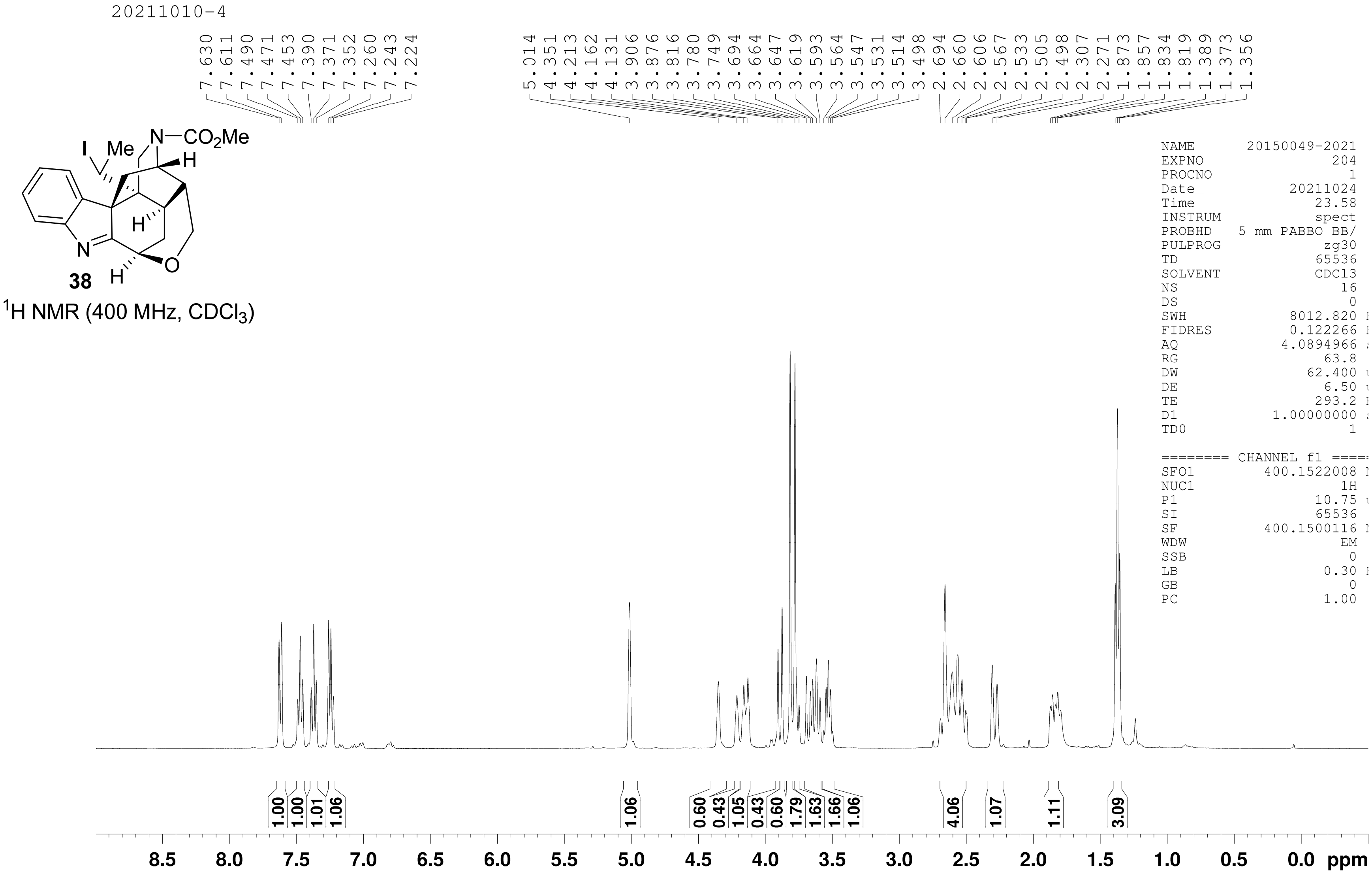

Supplementary Figure 102 ^{1}H-NMR (400 MHz, $CDCl_3$) spectra of **38**

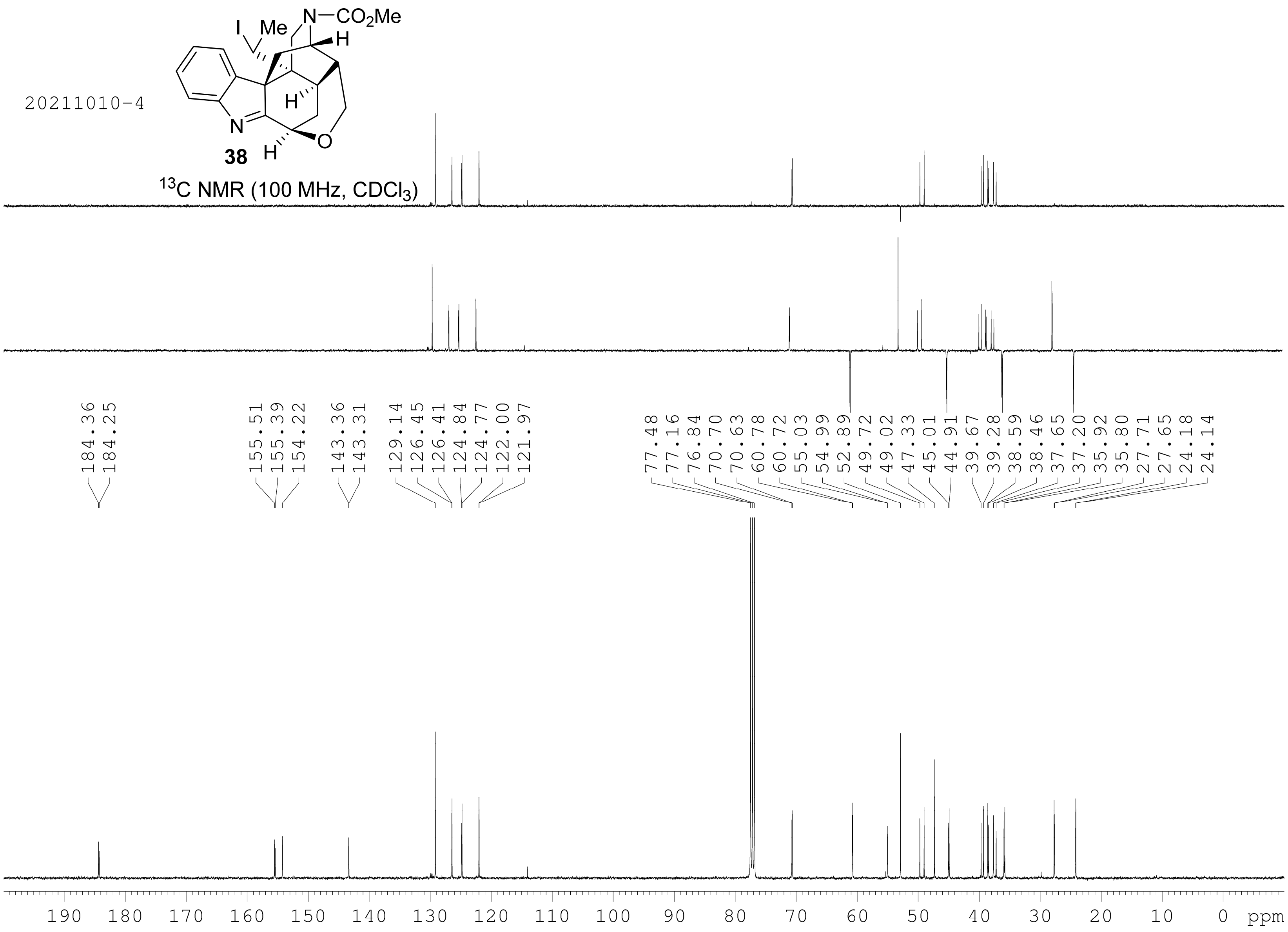

Supplementary Figure 103 ^{13}C-NMR (100 MHz, $CDCl_3$) spectra of **38**

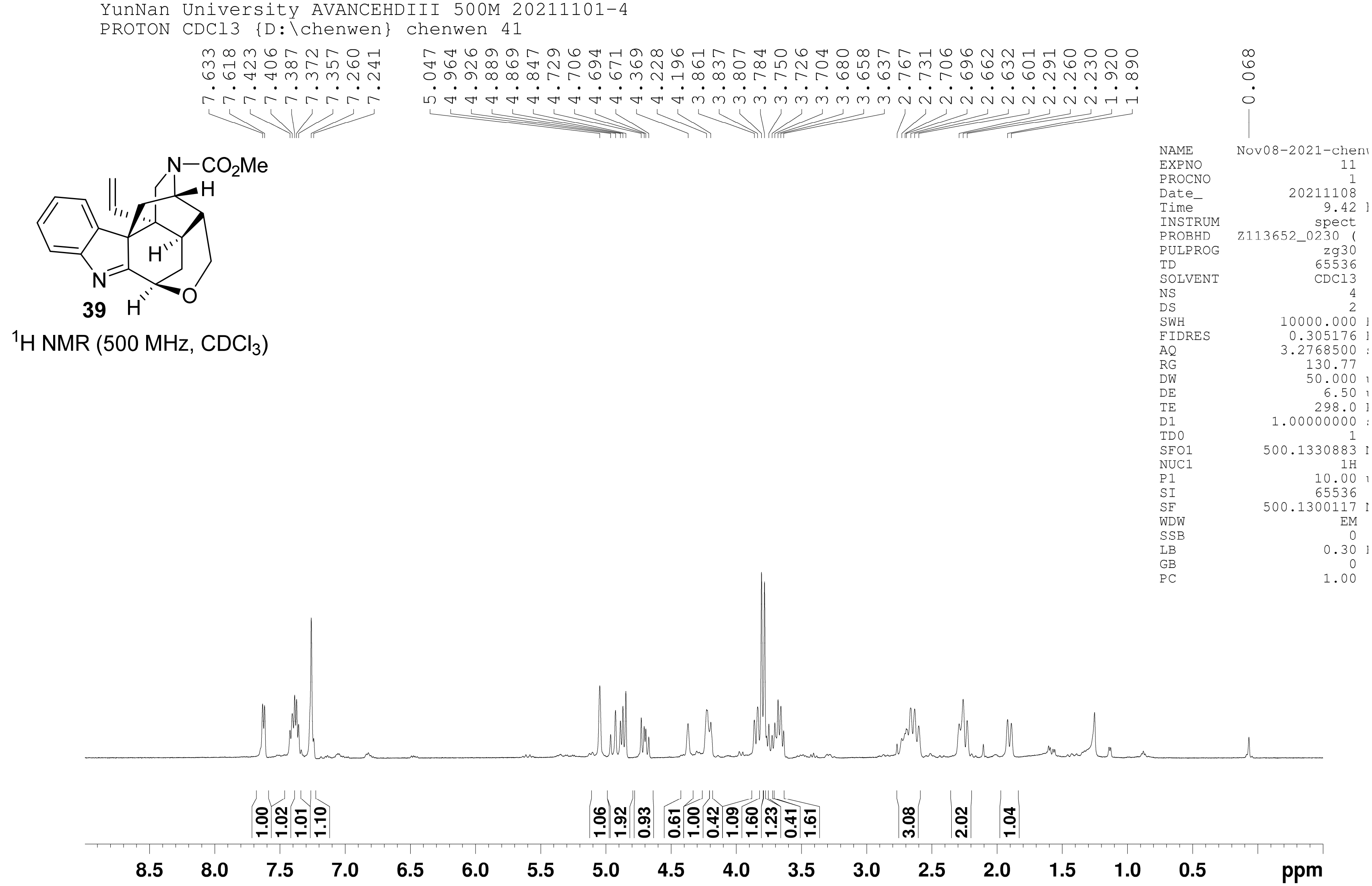

Supplementary Figure 104 ^{1}H-NMR (500 MHz, $CDCl_3$) spectra of **39**

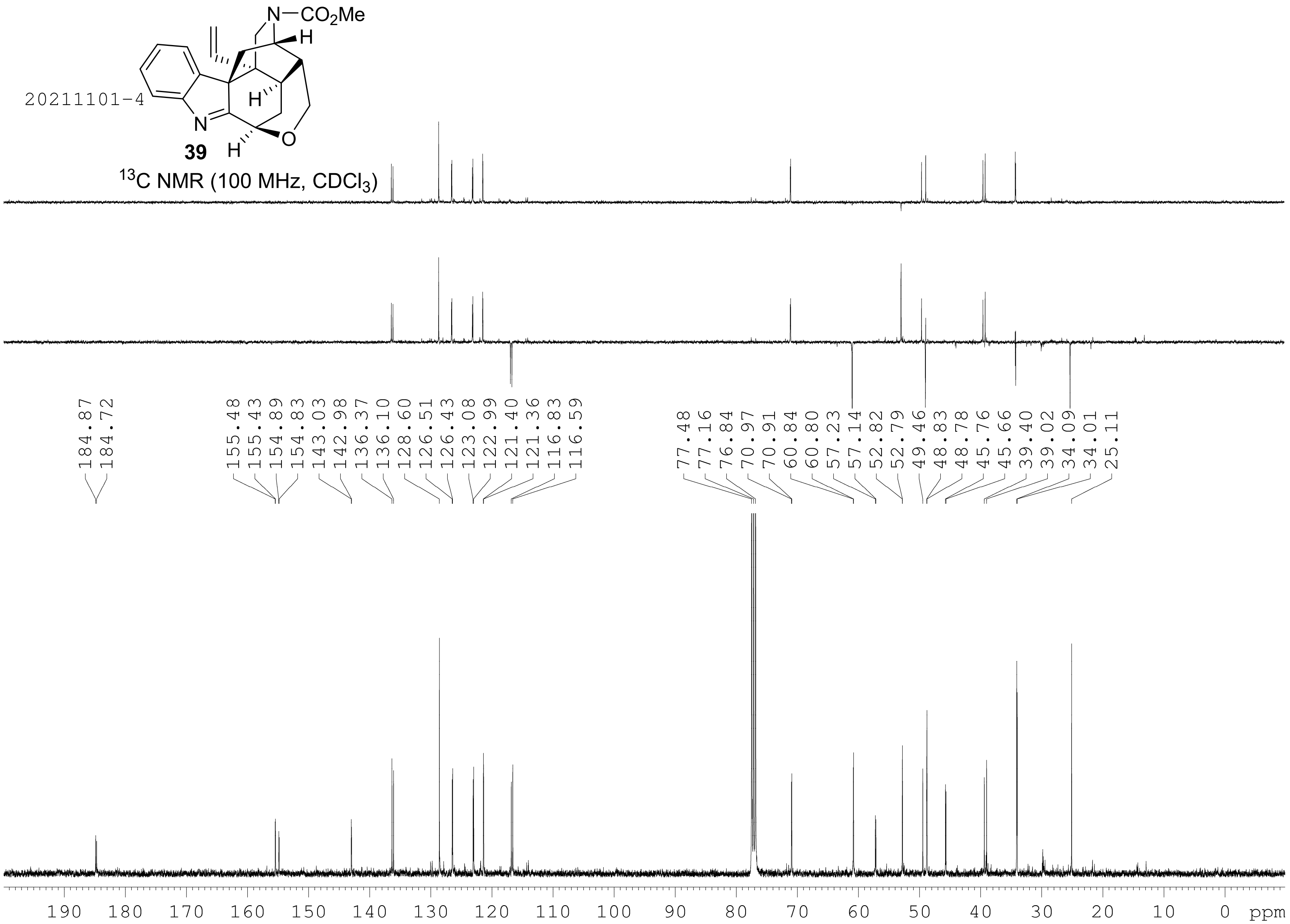

Supplementary Figure 105 ^{13}C-NMR (100 MHz, $CDCl_3$) spectra of **39**

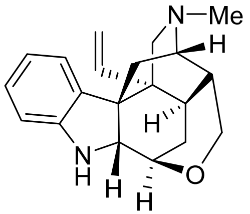

40: Dihydrokoumine

^{1}H NMR (600 MHz, $CDCl_3$)

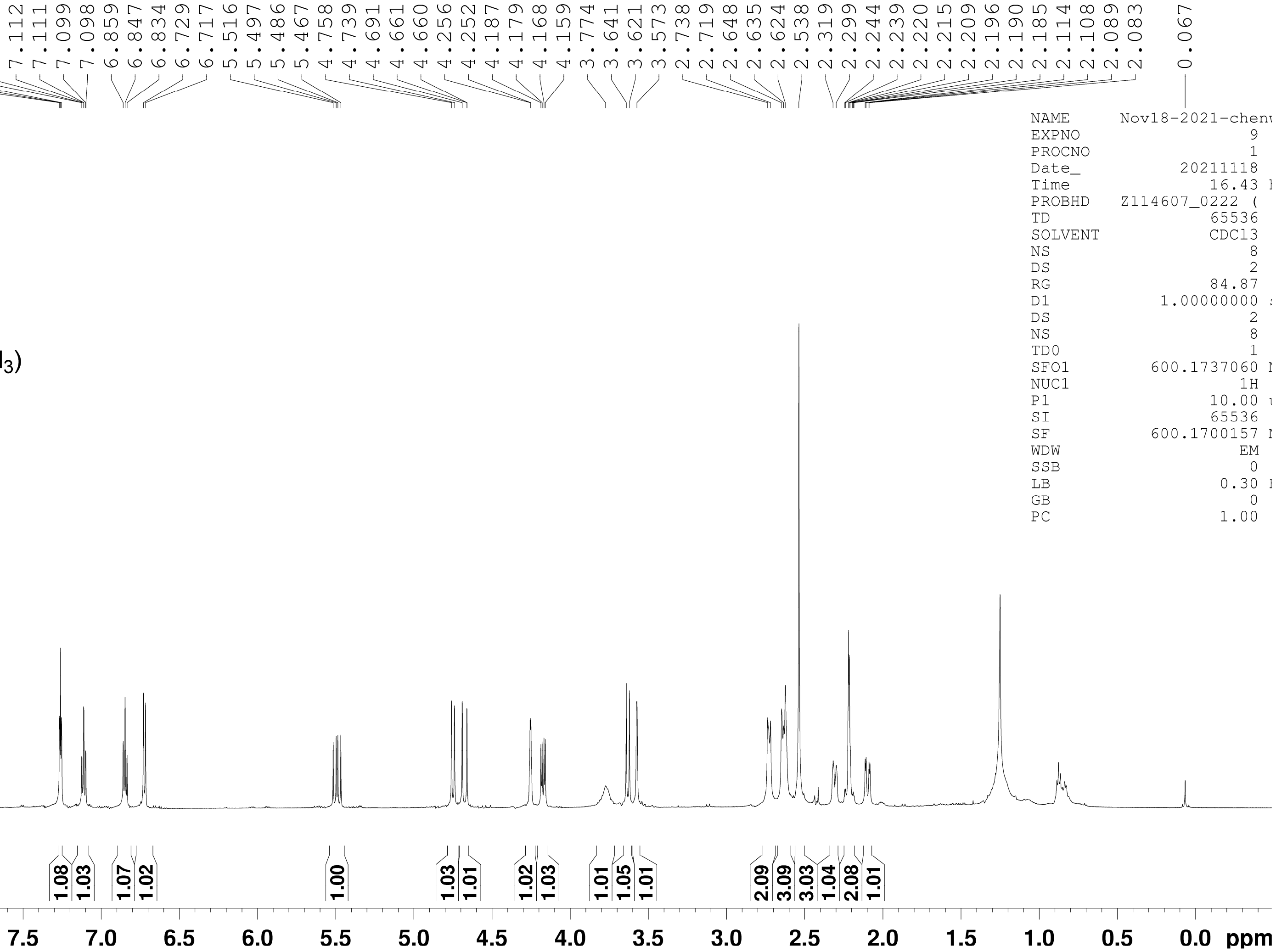

Supplementary Figure 106 ^{1}H-NMR (600 MHz, $CDCl_3$) spectra of Dihydrokoumine (**40**)

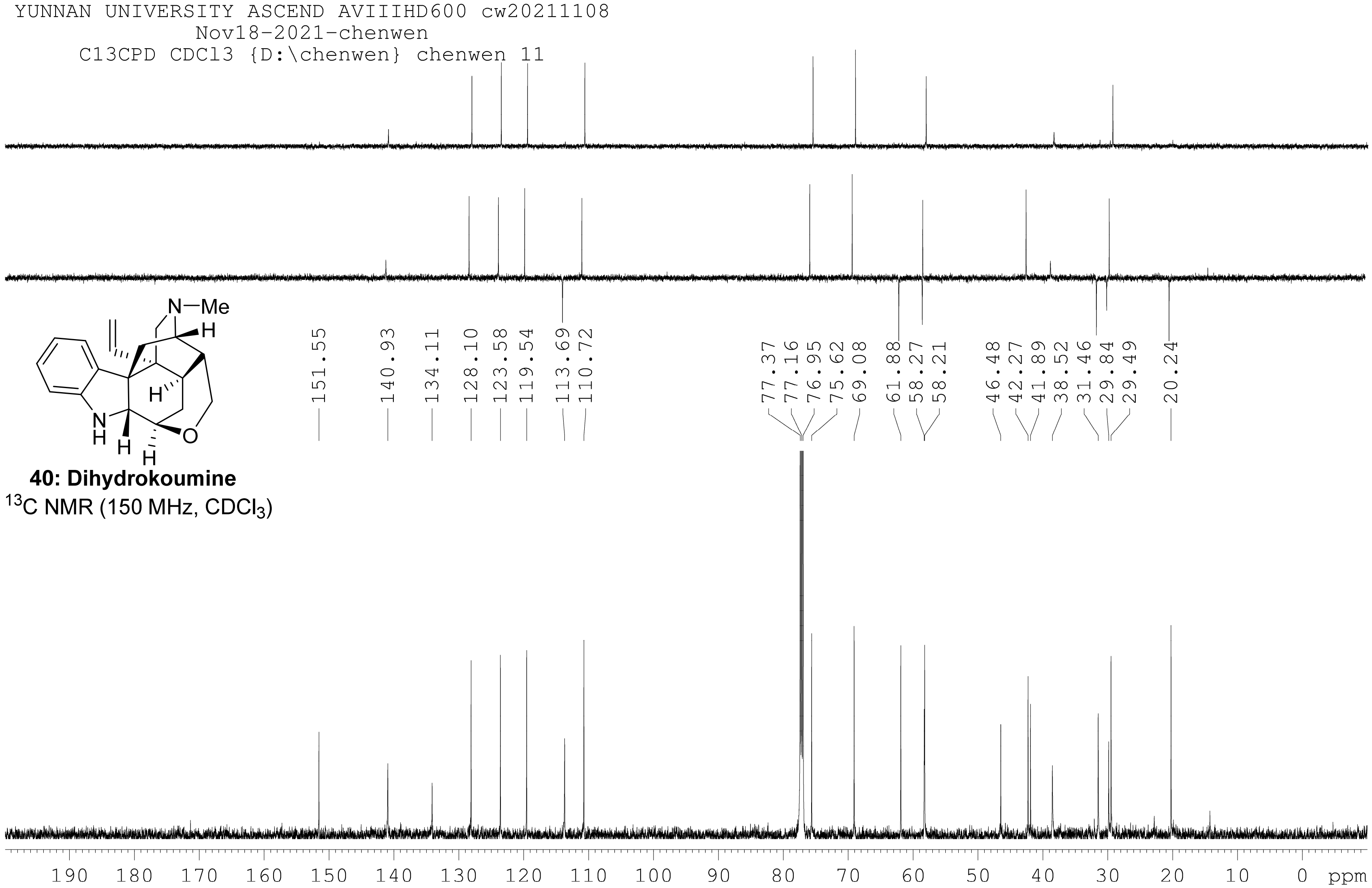

Supplementary Figure 107 ^{13}C-NMR (150 MHz, $CDCl_3$) spectra of Dihydrokoumine (**40**)

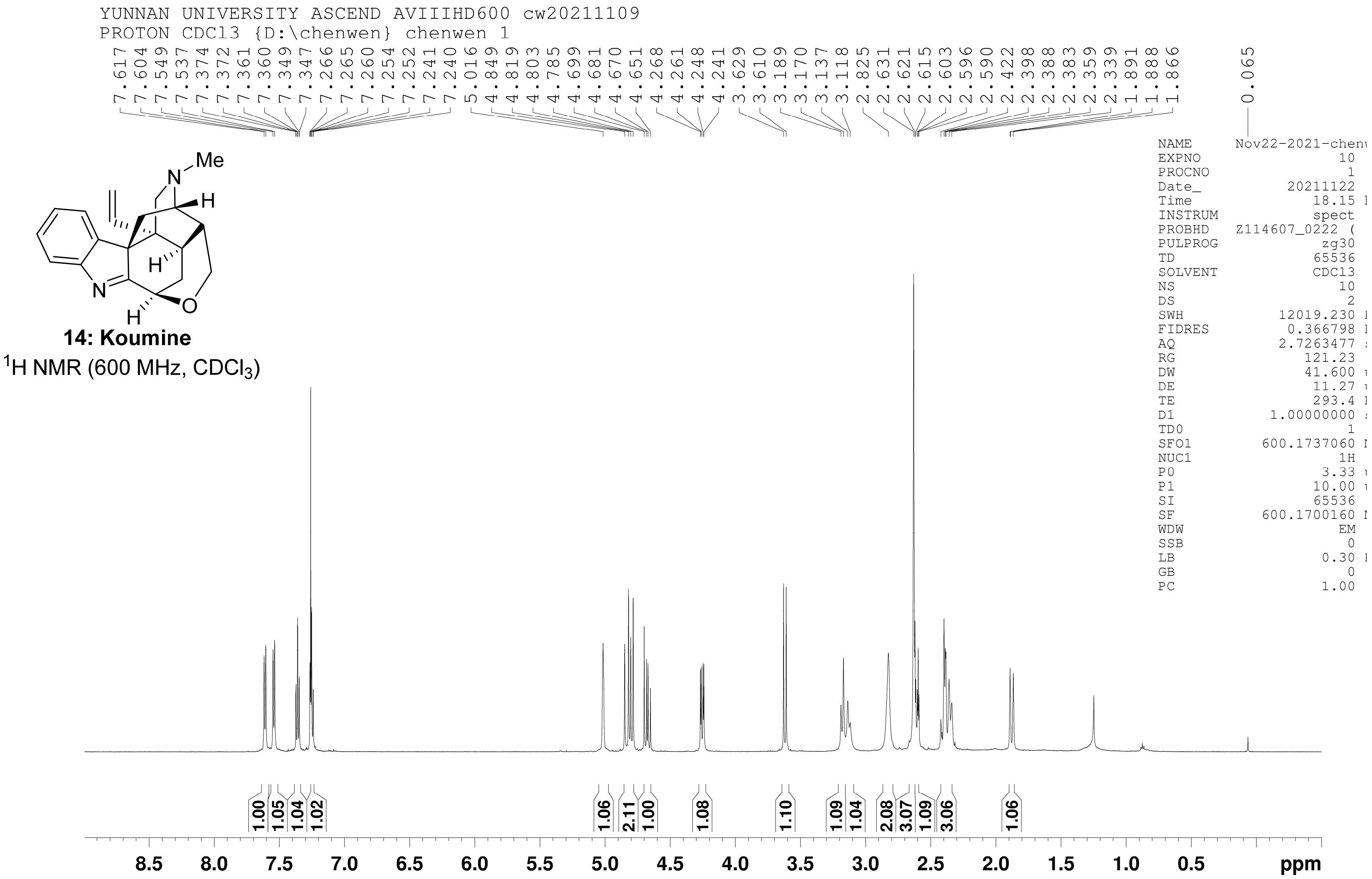

Supplementary Figure 108 ^{1}H-NMR (600 MHz, $CDCl_3$) spectra of Koumine (**14**)

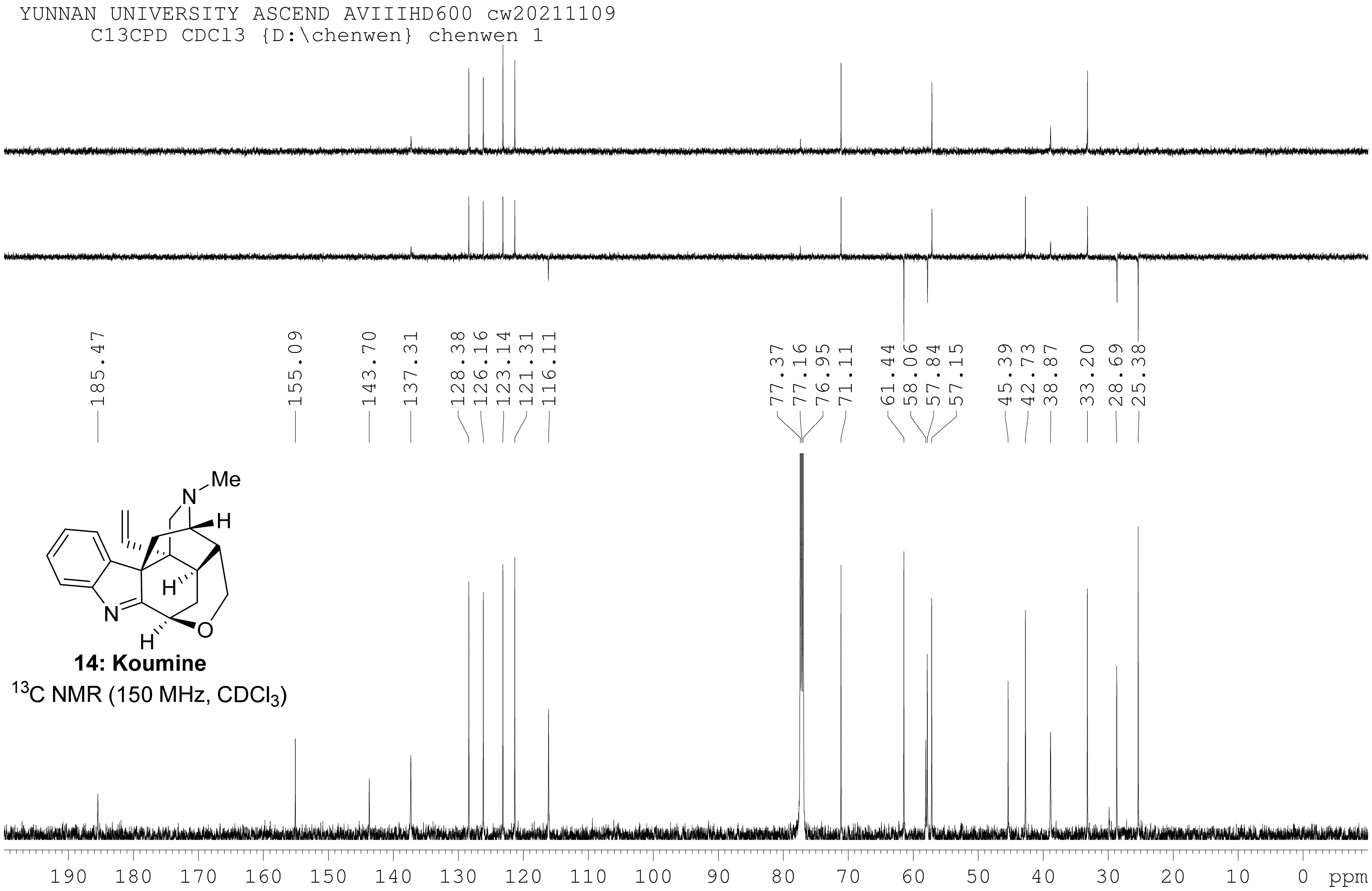

Supplementary Figure 109 ^{13}C-NMR (150 MHz, $CDCl_3$) spectra of Koumine (**14**)

6 X-Ray Single Crystal Diffraction Data

6.1 X-Ray single crystal diffraction data for 16a

Crystal data for **16a**: $C_{31}H_{34}N_2O_6$, M = 530.60, a = 11.1680(3) Å, b = 10.3887(3) Å, c = 12.2200(3) Å, α = 90°, β = 105.5310(10)°, γ = 90°, V = 1366.01(6) Å^3, T = 100.(2) K, space group P1211, Z = 2, μ(Cu Kα) = 0.729 mm^{-1}, 23579 reflections measured, 5373 independent reflections (R_{int} = 0.0409). The final R_1 values were 0.0288 ($I > 2\sigma(I)$). The final $wR(F^2)$ values were 0.0727 ($I > 2\sigma(I)$). The final R_1 values were 0.0289 (all data). The final $wR(F^2)$ values were 0.0728 (all data). The goodness of fit on F^2 was 1.068. Flack parameter = 0.00(4).

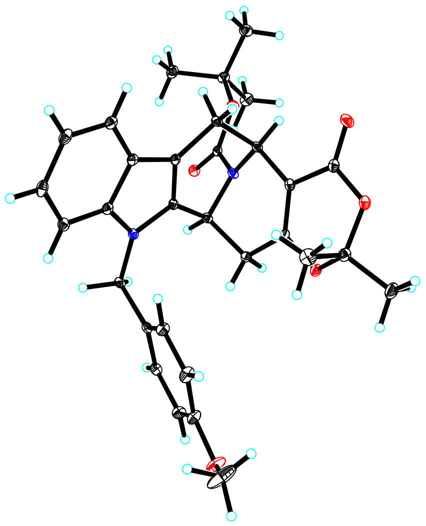

View of a molecule of **16a** with the atom-labelling scheme.

Displacement ellipsoids are drawn at the 30% probability level.

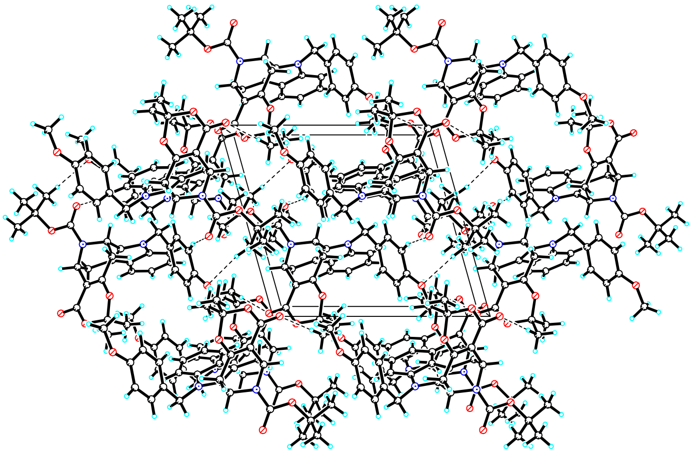

View of the pack drawing of **16a**.

Hydrogen-bonds are shown as dashed lines.

Supplementary Table 21 Crystal data and structure refinement for **16a.**

Identification code	global
Empirical formula	C31 H34 N2 O6
Formula weight	530.60
Temperature	100(2) K
Wavelength	1.54178 Å
Crystal system	Monoclinic
Space group	P 1 21 1
Unit cell dimensions	a = 11.1680(3) Å = 90°.
	b = 10.3887(3) Å =
105.5310(10)°.	
	c = 12.2200(3) Å = 90°.
Volume	1366.01(6) Å^3
Z	2
Density (calculated)	1.290 Mg/m^3
Absorption coefficient	0.729 mm^{-1}
F(000)	564
Crystal size	0.380 x 0.240 x 0.100 mm^3
Theta range for data collection	3.75 to 72.40°.
Index ranges	-12<=h<=13, -12<=k<=12, -15<=l<=15
Reflections collected	23579
Independent reflections	5373 [R(int) = 0.0409]

Completeness to theta = 72.40°	99.8 %
Absorption correction	Semi-empirical from equivalents
Max. and min. transmission	0.93 and 0.73
Refinement method	Full-matrix least-squares on F^2
Data / restraints / parameters	5373 / 1 / 358
Goodness-of-fit on F^2	1.068
Final R indices [I>2sigma(I)]	R1 = 0.0288, wR2 = 0.0727
R indices (all data)	R1 = 0.0289, wR2 = 0.0728
Absolute structure parameter	0.00(4)
Largest diff. peak and hole	0.240 and -0.183 e.Å^{-3}

6.2 X-Ray single crystal diffraction data for 25

Crystal data for **25**: $C_{28}H_{30}N_2O_3$, M = 442.54, a = 9.0083(2) Å, b = 10.4752(3) Å, c = 24.1304(6) Å, α = 90°, β = 90°, γ = 90°, V = 2277.03(10) Å^3, T = 100.(2) K, space group P212121, Z = 4, μ(Cu Kα) = 0.667 mm^{-1}, 37957 reflections measured, 4468 independent reflections (R_{int} = 0.0298). The final R_1 values were 0.0284 ($I > 2\sigma(I)$). The final $wR(F^2)$ values were 0.0742 ($I > 2\sigma(I)$). The final R_1 values were 0.0284 (all data). The final $wR(F^2)$ values were 0.0742 (all data). The goodness of fit on F^2 was 1.056. Flack parameter = 0.00(4).

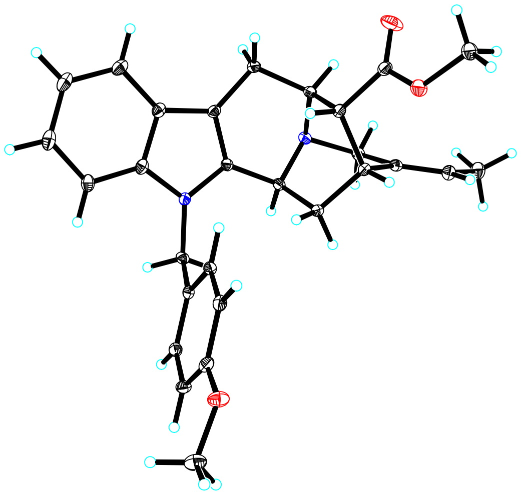

View of a molecule of **25** with the atom-labelling scheme.

Displacement ellipsoids are drawn at the 30% probability level.

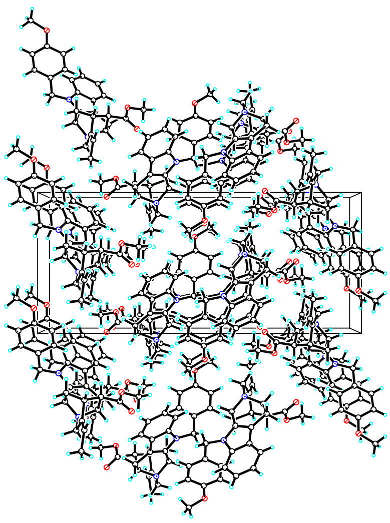

View of the pack drawing of **25**.

Hydrogen-bonds are shown as dashed lines.

Supplementary Table 22 Crystal data and structure refinement for **25.**

Identification code	global	
Empirical formula	C28 H30 N2 O3	
Formula weight	442.54	
Temperature	100(2) K	
Wavelength	1.54178 Å	
Crystal system	Orthorhombic	
Space group	$P2_12_12_1$	
Unit cell dimensions	a = 9.0083(2) Å	= 90°.
	b = 10.4752(3) Å	= 90°.
	c = 24.1304(6) Å	= 90°.
Volume	2277.03(10) Å^3	
Z	4	
Density (calculated)	1.291 Mg/m^3	
Absorption coefficient	0.667 mm^{-1}	
F(000)	944	
Crystal size	0.400 x 0.200 x 0.200 mm^3	
Theta range for data collection	3.66 to 72.33°.	
Index ranges	-11<=h<=11, -12<=k<=12, -29<=l<=29	
Reflections collected	37957	

Independent reflections	4468 [R(int) = 0.0298]
Completeness to theta = 72.33°	99.3 %
Absorption correction	Semi-empirical from equivalents
Max. and min. transmission	0.88 and 0.75
Refinement method	Full-matrix least-squares on F^2
Data / restraints / parameters	4468 / 0 / 301
Goodness-of-fit on F^2	1.056
Final R indices [I>2sigma(I)]	R1 = 0.0284, wR2 = 0.0742
R indices (all data)	R1 = 0.0284, wR2 = 0.0742
Absolute structure parameter	0.00(4)
Largest diff. peak and hole	0.192 and -0.168 e.Å^{-3}

6.3 X-Ray single crystal diffraction data for Akuammidine (1)

Crystal data for **1**: $4(C_{21}H_{24}N_2O_3)\bullet C_4H_8O_2$, M = 1497.79, a = 12.8054(7) Å, b = 22.6423(14) Å, c = 13.3717(8) Å, α = 90°, β = 90.798(3)°, γ = 90°, V = 3876.7(4) Å^3, T = 100.(2) K, space group P1211, Z = 2, μ(Cu Kα) = 0.703 mm^{-1}, 81166 reflections measured, 15270 independent reflections (R_{int} = 0.0861). The final R_1 values were 0.0389 ($I > 2\sigma(I)$). The final $wR(F^2)$ values were 0.0889 ($I > 2\sigma(I)$). The final R_1 values were 0.0449 (all data). The final $wR(F^2)$ values were 0.0929 (all data). The goodness of fit on F^2 was 1.036. Flack parameter = -0.02(7).

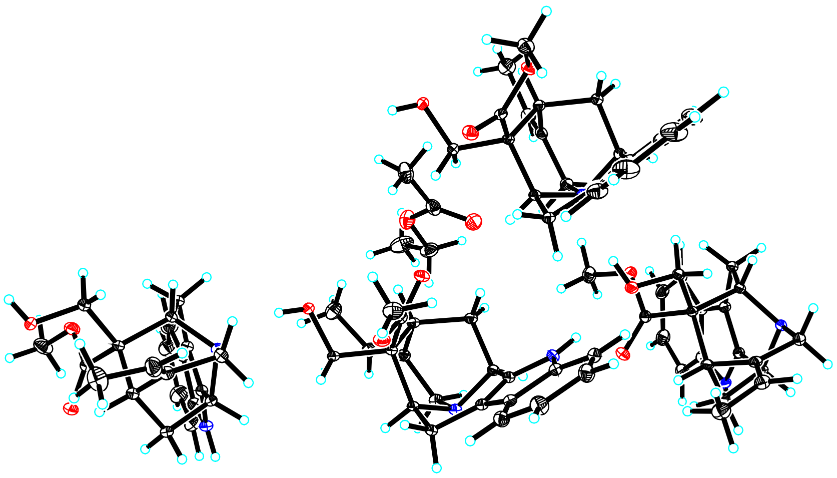

View of the molecules in an asymmetric unit.

Displacement ellipsoids are drawn at the 30% probability level.

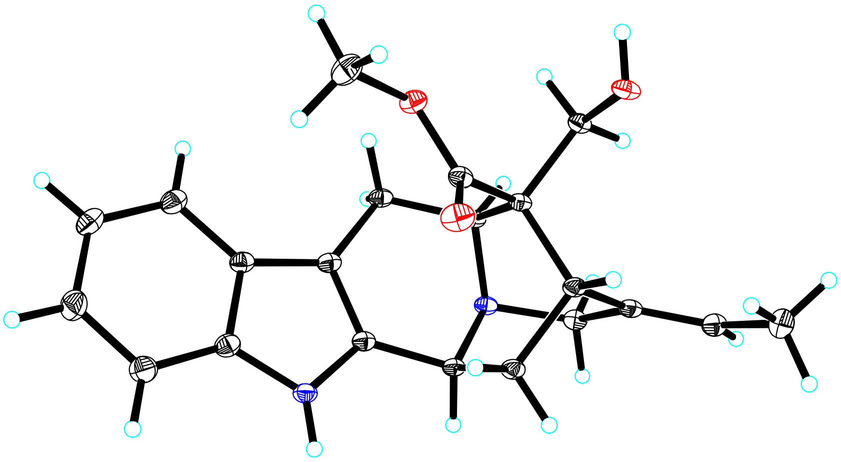

View of a molecule of **1** with the atom-labelling scheme.

Displacement ellipsoids are drawn at the 30% probability level.

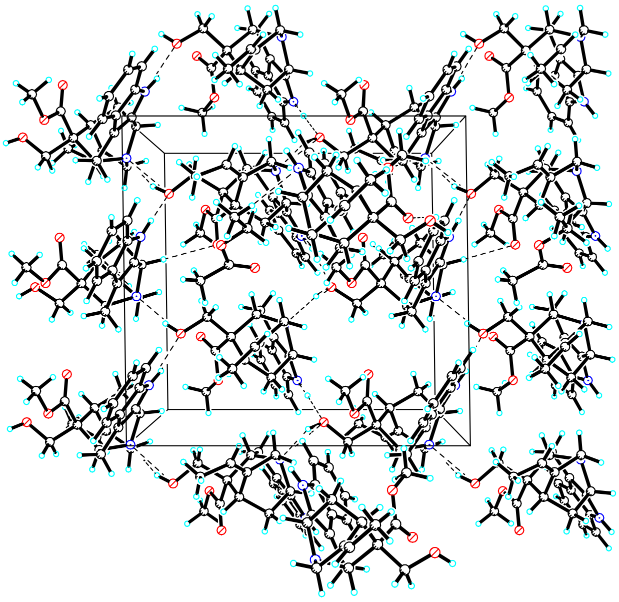

View of the pack drawing of **1**.

Hydrogen-bonds are shown as dashed lines.

Supplementary Table 23 Crystal data and structure refinement for **1.**

Identification code	global	
Empirical formula	C88 H104 N8 O14	
Formula weight	1497.79	
Temperature	100(2) K	
Wavelength	1.54178 Å	
Crystal system	Monoclinic	
Space group	P 1 21 1	
Unit cell dimensions	a = 12.8054(7) Å	= 90°.
	b = 22.6423(14) Å	= 90.798(3)°.
	c = 13.3717(8) Å	= 90°.
Volume	3876.7(4) $Å^3$	
Z	2	
Density (calculated)	1.283 Mg/m^3	
Absorption coefficient	0.703 mm^{-1}	
F(000)	1600	
Crystal size	0.800 x 0.350 x 0.006 mm^3	
Theta range for data collection	3.31 to 72.50°.	
Index ranges	-15<=h<=13, -27<=k<=28, -16<=l<=16	
Reflections collected	81166	
Independent reflections	15270 [R(int) = 0.0861]	
Completeness to theta = 72.50°	99.8 %	
Absorption correction	Semi-empirical from equivalents	
Max. and min. transmission	1.00 and 0.82	
Refinement method	Full-matrix least-squares on F^2	
Data / restraints / parameters	15270 / 1 / 1005	
Goodness-of-fit on F^2	1.036	
Final R indices [I>2sigma(I)]	R1 = 0.0389, wR2 = 0.0889	
R indices (all data)	R1 = 0.0449, wR2 = 0.0929	
Absolute structure parameter	-0.02(7)	
Largest diff. peak and hole	0.200 and -0.232 e.$Å^{-3}$	

6.4 X-Ray single crystal diffraction data for Quebrachidine (5)

Crystal data for **5**: $C_{21}H_{24}N_2O_3$, M = 352.42, a = 8.8914(2) Å, b = 21.3196(5) Å, c = 9.4708(2) Å, α = 90°, β = 97.2780(10)°, γ = 90°, V = 1780.83(7) $Å^3$, T = 100.(2) K, space group P1211, Z = 4, μ(Cu Kα) = 0.710 mm^{-1}, 44904 reflections measured, 6923

independent reflections (R_{int} = 0.0319). The final R_1 values were 0.0290 ($I > 2\sigma(I)$). The final $wR(F^2)$ values were 0.0766 ($I > 2\sigma(I)$). The final R_1 values were 0.0293 (all data). The final $wR(F^2)$ values were 0.0769 (all data). The goodness of fit on F^2 was 1.043. Flack parameter = 0.06(3).

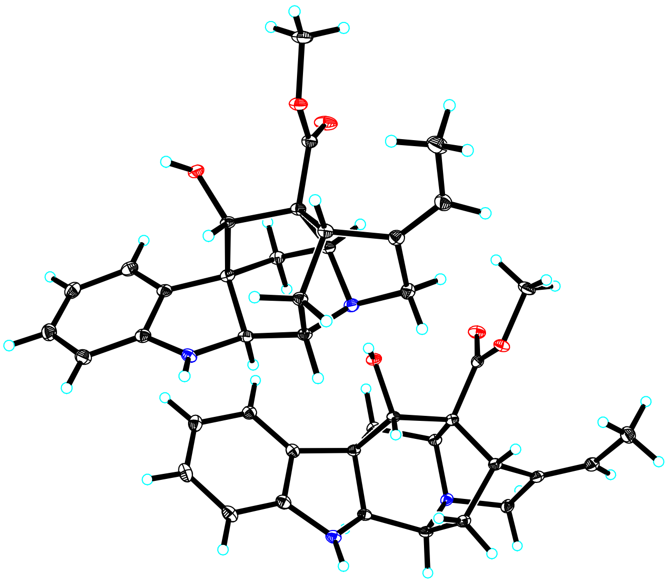

View of the molecules in an asymmetric unit.

Displacement ellipsoids are drawn at the 30% probability level.

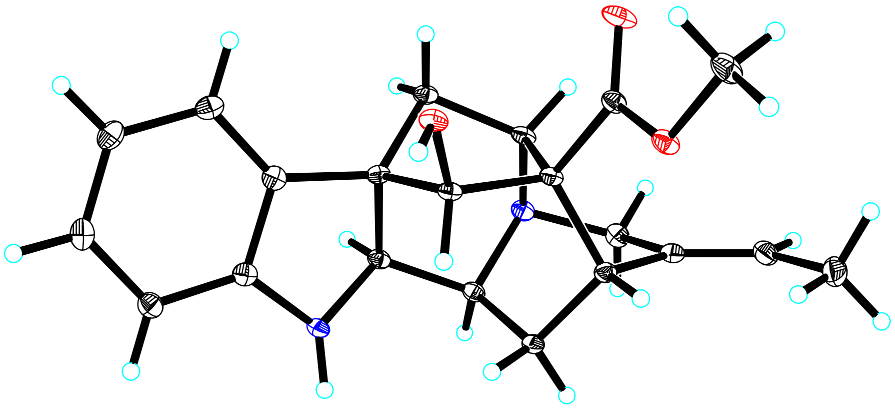

View of a molecule of **5** with the atom-labelling scheme.

Displacement ellipsoids are drawn at the 30% probability level.

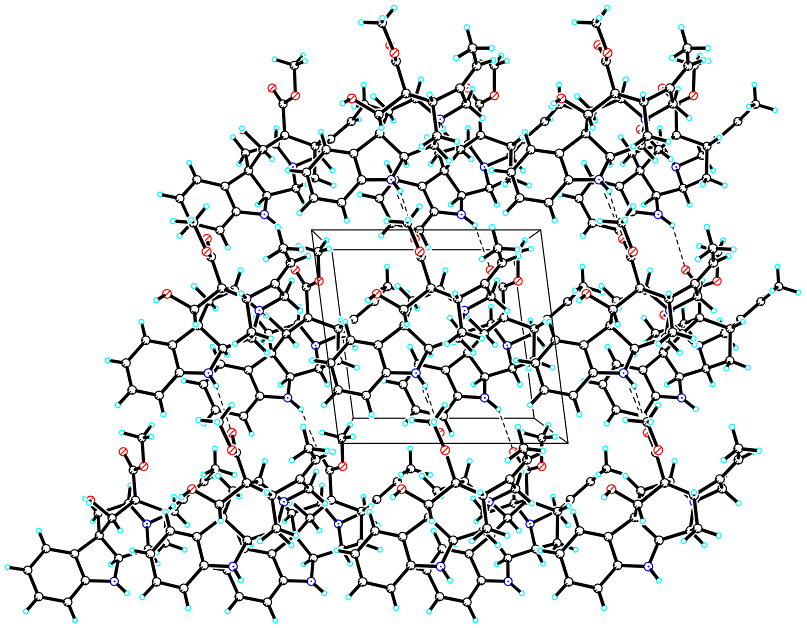

View of the pack drawing of **5**.

Hydrogen-bonds are shown as dashed lines.

Supplementary Table 24 Crystal data and structure refinement for **5.**

Identification code	global	
Empirical formula	C21 H24 N2 O3	
Formula weight	352.42	
Temperature	100(2) K	
Wavelength	1.54178 Å	
Crystal system	Monoclinic	
Space group	P 1 21 1	
Unit cell dimensions	a = 8.8914(2) Å	= 90°.
	b = 21.3196(5) Å	= 97.2780(10)°.
	c = 9.4708(2) Å	= 90°.
Volume	1780.83(7) Å^3	
Z	4	
Density (calculated)	1.314 Mg/m^3	
Absorption coefficient	0.710 mm^{-1}	
F(000)	752	
Crystal size	0.260 x 0.200 x 0.080 mm^3	
Theta range for data collection	4.15 to 72.33°.	

Index ranges	-10<=h<=10, -26<=k<=26, -11<=l<=11
Reflections collected	44904
Independent reflections	6923 [R(int) = 0.0319]
Completeness to theta = 72.33°	99.9 %
Absorption correction	Semi-empirical from equivalents
Max. and min. transmission	0.94 and 0.83
Refinement method	Full-matrix least-squares on F^2
Data / restraints / parameters	6923 / 1 / 475
Goodness-of-fit on F^2	1.043
Final R indices [I>2sigma(I)]	R1 = 0.0290, wR2 = 0.0766
R indices (all data)	R1 = 0.0293, wR2 = 0.0769
Absolute structure parameter	0.06(3)
Largest diff. peak and hole	0.405 and -0.401 e.Å^{-3}

6.5 X-Ray single crystal diffraction data for 25b

Crystal data for **25b**: $C_{28}H_{29}IN_2O_3$, M = 568.43, a = 8.4378(3) Å, b = 11.5538(4) Å, c = 24.6272(8) Å, α = 90°, β = 90°, γ = 90°, V = 2400.87(14) Å^3, T = 100.(2) K, space group P212121, Z = 4, μ(Cu Kα) = 10.745 mm^{-1}, 16791 reflections measured, 4551 independent reflections (R_{int} = 0.0706). The final R_1 values were 0.1041 ($I > 2\sigma(I)$). The final $wR(F^2)$ values were 0.2427 ($I > 2\sigma(I)$). The final R_1 values were 0.1044 (all data). The final $wR(F^2)$ values were 0.2430 (all data). The goodness of fit on F^2 was 1.179. Flack parameter = 0.273(6).

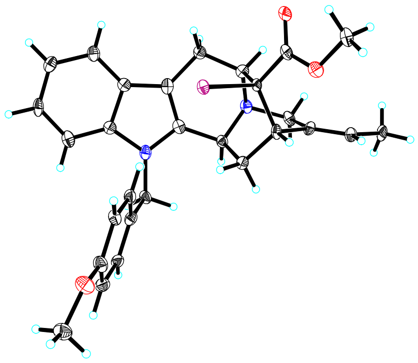

View of a molecule of **25b** with the atom-labelling scheme.

Displacement ellipsoids are drawn at the 30% probability level.

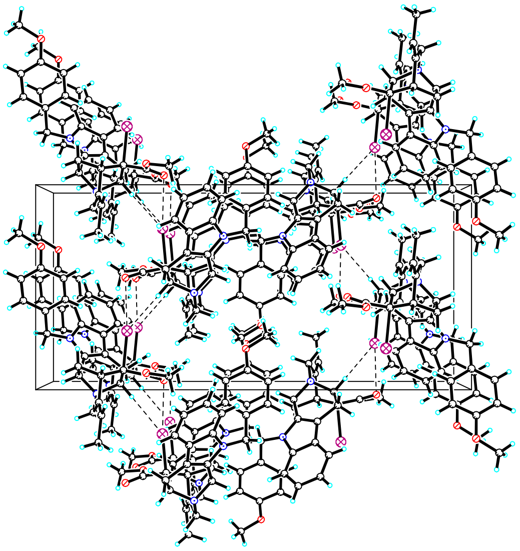

View of the pack drawing of **25b**.

Hydrogen-bonds are shown as dashed lines.

Supplementary Table 25 Crystal data and structure refinement for **25b**.

Identification code	global	
Empirical formula	C28 H29 I N2 O3	
Formula weight	568.43	
Temperature	100(2) K	
Wavelength	1.54178 Å	
Crystal system	Orthorhombic	
Space group	$P2_12_12_1$	
Unit cell dimensions	a = 8.4378(3) Å	α= 90°.
	b = 11.5538(4) Å	β= 90°.
	c = 24.6272(8) Å	γ = 90°.
Volume	2400.87(14) Å^3	
Z	4	
Density (calculated)	1.573 Mg/m^3	
Absorption coefficient	10.745 mm^{-1}	
F(000)	1152	
Crystal size	0.400 x 0.300 x 0.250 mm^3	
Theta range for data collection	3.59 to 72.14°.	
Index ranges	-10<=h<=10, -14<=k<=14, -27<=l<=30	
Reflections collected	16791	
Independent reflections	4551 [R(int) = 0.0706]	
Completeness to theta = 72.14°	99.5 %	

Absorption correction	Semi-empirical from equivalents
Max. and min. transmission	0.17 and 0.01
Refinement method	Full-matrix least-squares on F^2
Data / restraints / parameters	4551 / 0 / 310
Goodness-of-fit on F^2	1.179
Final R indices [I>2sigma(I)]	R1 = 0.1041, wR2 = 0.2427
R indices (all data)	R1 = 0.1044, wR2 = 0.2430
Absolute structure parameter	0.273(6)
Largest diff. peak and hole	5.037 and -2.271 e.Å^{-3}

2. Supplementary References

(1) Davis, F. A., Melamed, J. Y. & Sharik, S. S. Total synthesis of (–)-normalindine via addition of metalated 4-methyl-3-cyanopyridine to an enantiopure sulfinimine. *J. Org. Chem.* **71**, 8761–8766 (2006).

(2) Hashimoto, T., Nakatsu, H., Yamamoto, K. & Maruoka, K. Chiral Brønsted acid-catalyzed asymmetric trisubstituted aziridine synthesis using α-diazoacyl oxazolidinones. *J. Am. Chem. Soc.* **133**, 9730–9733 (2011).

(3) Silvers, S. & Tulinsky, A. The structure of akuammidine. *Tetrahedron Lett.* **3**, 339–343 (1963).

(4) Ponglux, D., Wongseripipatana, S., Subhadhirasakul, S., Takayama, H., Yokota, M., Ogata, K., Phisalaphong, C., Airni, N. & Sakair S. Studies on the indole alkaloids of *Gelsemium elegans* (Thailand): Structure elucidation and proposal of biogenetic route. *Tetrahedron* **44**, 5075–5094 (1988).

(5) Antonaccio, L. D., Pereira, N. A., Gilbert, B., Vorbrueggen, H., Budzikiewicz, H., Wilson, J. M., Durham, L. J. & Djerassi, C. Alkaloid studies. XXXIII. Mass spectrometry in structural and stereochemical problems. VI. polyneuridine, a new alkaloid from *Aspidosperma polyneuron* and some observations on mass spectra of indole alkaloids. *J. Am. Chem. Soc.* **84**, 2161–2169 (1962).

(6) Yuldashev, P. K. & Yunusov S. Y. The structure of vincarine. *Khimiya Prirodnykh Soedinenii* **1**, 110-113 (1965); *Chem. Nat. Compd.* **1**, 85–87 (1965).

(7) Janot, M. M., Le Men, J. & Hammouda, Y. Vincamedine, a (new) alkaloid crystallized from *Vinca difformis*. *Compt. Rend.* **243**, 85–87 (1956).

(8) Patel, M. B., Poisson, J., Pousset, J. L. & Rowson, J. M. Vincamajine, the major alkaloid of leaves of *Rauwolfia mannii* Stapf. *J. Pharm. Pharmacol.* **17**, 323–324 (1965).

(9) Vachnadze, V. Yu., Dzhakeli, É. Z., Robakidze, Z. V., Chkhikvadze, G. V., Mudzhiri, M. M., Abuladze, G. V. & Chuchulashvili, N. A. Chemical composition and pharmacological activity of alkaloids from the common periwinkle cultured in

Georgia. *Pharm. Chem. J.* **35**, 268–270 (2001).

(10) Gosset-Garnier, J., Le Men, J. & Janot, M. M. Periwinkle alkaloids. XXXI. Stereochemistry of vincamedine and vincamajine. *Bulletin de la Societe Chimique de France* **1965**, 676–678 (1965).

(11) Yu, J., Wearing, X. Z. & Cook, J. M. A general strategy for the synthesis of vincamajine-related indole alkaloids: Stereocontrolled total synthesis of (+)-dehydrovoachalotine, (–)-vincamajinine, and (–)-11-methoxy-17-epivincamajine as well as the related quebrachidine diol, vincamajine diol, and vincarinol. *J. Org. Chem.* **70**, 3963–3979 (2005).

(12) Arai, H., Zaima, K., Mitsuta, E., Tamamoto, H., Saito, A., Hirasawa, Y., Rahman, A., Kusumawati, I., Zaini, N. C. & Morita, H. Alstiphyllanines I–O, ajmaline type alkaloids from *Alstonia macrophylla* showing vasorelaxant activity. *Bioorg. Med. Chem.* **20**, 3454–3459 (2012).

(13) Schun, Y.; Cordell, G. A. Revision of the Stereochemistry of Koumidine. *Phytochemistry* **26**, 2875–2876 (1986).

(14) Cao, H.; Yu, J.; Wearing, X. Z.; Zhang, C.; Liu, X.; Deschamps, J.; Cook, J. M. The first enantiospecific synthesis of (–)-koumidine via the intramolecular palladium-catalyzed enolate driven cross coupling reaction. The stereospecific introduction of the 19-(*Z*) ethylidene side chain. *Tetrahedron Lett.* **44**, 8013–8017 (2003).

(15) Takayama, H., Kitajima, M., Wongseripipatana, S. & Sakai S. Partial synthesis and the absolute configuration of two new *Gelsemiurn* Alkaloids, Koumidine and (19*Z*)-Taberpsychine. *J. Chem. Soc., Perkin Trans. 1* **1989**, 1075–1076 (1989).

(16) Zhang, Z. P., Liang, X. T., Sun, F., Lu, Y. & Yang, J. Studies on the indole alkaloids of Gelsemium elegans. *Chin. Chem. Lett.* **2**, 365–368 (1991).

(17) Yang, Z., Tan, Q., Jiang, Y., Yang, J., Su, X., Qiao, Z., Zhou, W., He, L., Qiu, H. & Zhang, M. Asymmetric total synthesis of sarpagine and koumine alkaloids. *Angew. Chem. Int. Ed.* **60**, 13105–13111 (2021).

(18) Creed, S. M., Gutridge, A. M., Argade, M. D., Hennessy, M. R., Friesen, J. B., Pauli, G. F., van Rijn, R. M. & Riley A. P. Isolation and pharmacological

characterization of six opioidergic *Picralima nitida* alkaloids. *J. Nat. Prod.* **84**, 71–80 (2021).
(19) Jokela, R. & Lounasmaa, M. ^{1}H-and ^{13}C-NMR spectral data of five sarpagine-type alkaloids. *Heterocycles* **43**, 1015–1020 (1996).
(20) Yin, W., Kabir, M. S., Wang, Z., Rallapalli, S. K., Ma, J. & Cook, J. M. Enantiospecific total synthesis of the important biogenetic intermediates along the ajmaline pathway, (+)-polyneuridine and (+)-polyneuridine aldehyde, as well as 16-epivellosimine and macusine A. *J. Org. Chem.* **75**, 3339–3349 (2010).
(21) Jokela, R. & Lounasmaa, M. A ^{1}H- and ^{13}C-NMR study of seven ajmaline-type alkaloids. *Planta Med.* **62**, 577–579 (1996).
(22) Lounasmaa, M. & Jokela, R. A 400 MHz ^{1}H NMR study of twelve ajmaline-type alkaloids. *Heterocycles* **23**, 1053–1058 (1985).
(23) Zhukovich, E. N. & Vachnadze, V. Yu. Vincamajinine — A new alkaloid from *Vinca major*. *Chem. Nat. Compd.* **21**, 682 (1985).
(24) Kerkovius, J. K. & Kerr, M. A. Total synthesis of isodihydrokoumine, (19*Z*)-taberpsychine, and (4*R*)-isodihydroukoumine N_4-Oxide. *J. Am. Chem. Soc.* 140, 8415−8419 (2018).
(25) Zhang, W., Zhang, S.-Y., Wang, G.-Y., Li, N.-P., Chen, M.-F., Gu, J.-H., Zhang, D.-M., Wang, L., & Ye, W.-C. Five new koumine-type alkaloids from the roots of *Gelsemium elegans*. *Fitoterapia* **118**, 112–117 (2017).
(26) Liu, C.-T., Wang, Q.-W. & Wang, C.-H. Structure of koumine. *J. Am. Chem. Soc.* **103**, 4634–4635 (1981).
(27) Liu, Z.-J. & Wang, Q.-W. Structure of koumine. *Acta Chim. Sin.* **44**, 157–172 (1986).
